# Generation of Polysubstituted Tetrahydrofurans via Urea-Enabled, Pd-Catalyzed Olefin Heteroannulation

**DOI:** 10.1021/acs.orglett.6c00629

**Published:** 2026-03-05

**Authors:** Shannon T. O’Neil, Owen E. Monteferrante, Brooke R. Stanley, Shauna M. Paradine

**Affiliations:** Department of Chemistry, 6927 University of Rochester, 120 Trustee Road, Rochester, New York 14627, United States

## Abstract

Herein, we report a palladium-catalyzed heteroannulation approach to couple 2-bromoallyl alcohols and 1,3-dienes to generate a broad range of polysubstituted tetrahydrofuran (THF) rings enabled by urea ligands. Under urea-enabled Pd catalysis, a structurally diverse range of ambiphiles and dienes can be engaged to generate THF products with varying substitution patterns. These products can be readily transformed into prevalent core scaffolds in polyketide natural products.

## Graphical abstract

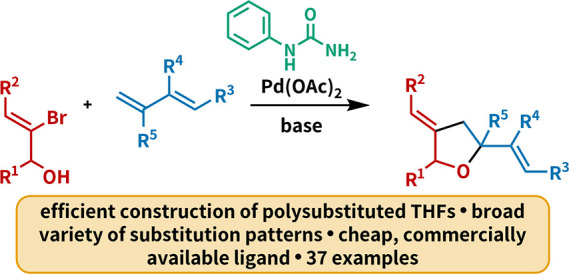

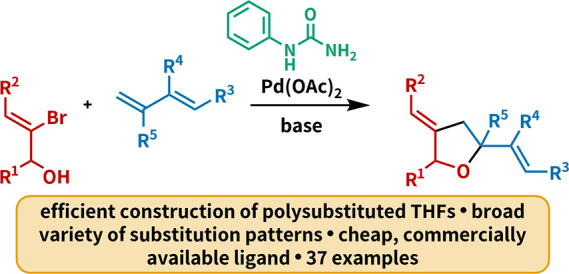

Oxygen-containing heterocycles are ubiquitous structural motifs in both naturally and synthetically derived organic small molecules, being used as pigments, agrochemicals, and therapeutics.^1^ The majority of U.S. Food and Drug Administration (FDA)-approved drugs containing oxygen heterocycles include aliphatic five-membered rings. The tetrahydrofuran (THF) motif is among the most common, appearing in a wide variety of bioactive molecules.^2^ THF-containing polyketide macrolides represent a growing class of bioactive natural products,^3^ many of which have 2,3,5-substitution, including a methylene or alcohol group at the 3 position (Figure 1A).^4^ As a result, the discovery of methods for the construction of substituted THF products has been of longstanding interest in organic synthesis.^5^

**Figure 1:**
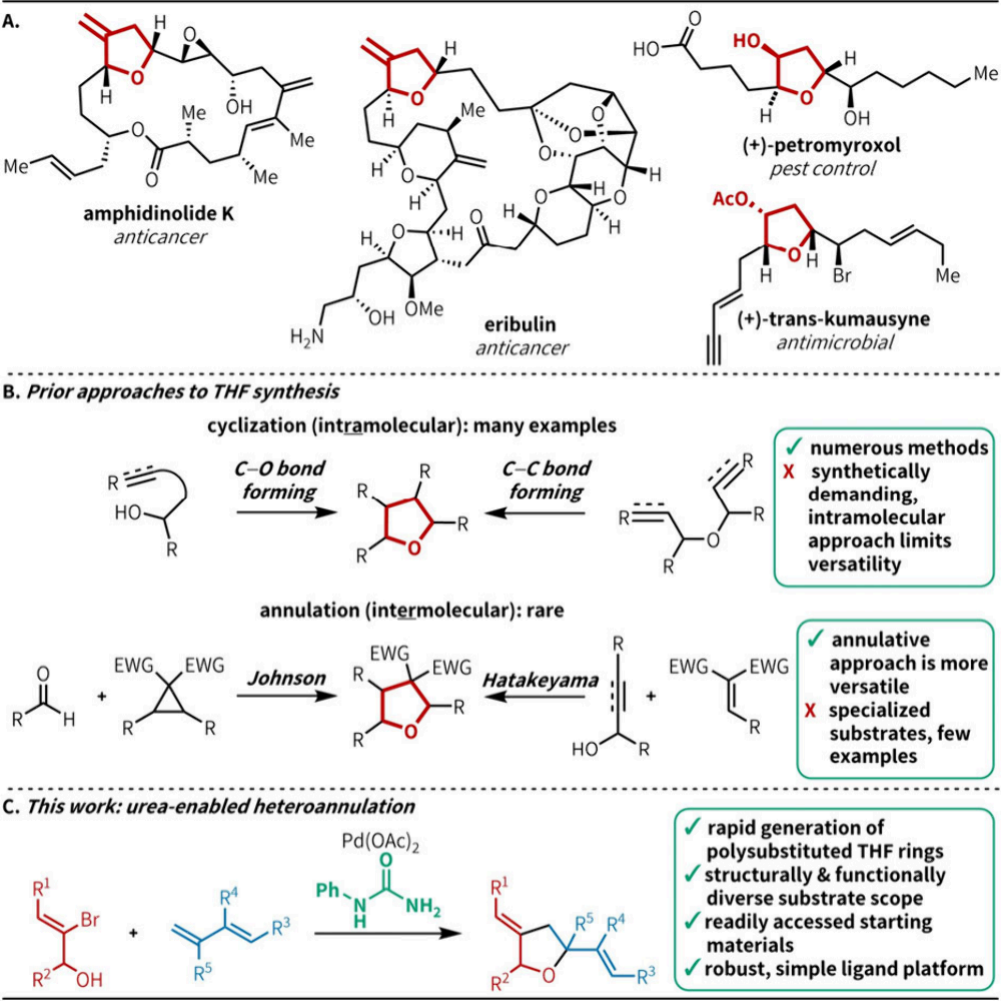
Relevance of polysubstituted THFs. (A) Bioactive organic small molecules containing THFs. (B) Prior THF synthetic strategies. (C) This work: urea-enabled heteroannulation promotes the rapid, annulative construction of polysubstituted THFs.

Common approaches to construct polysubstituted THF rings typically rely on intramolecular C–C or C–O bond formation via cyclization.^6^ While effective, this approach inherently limits the versatility of heterocycle synthesis, as the overall structure must be constructed prior to the key cyclization step. In contrast, an annulative approach, wherein two (or more) fragments are coupled in an intermolecular fashion, allows for the ready diversification of core scaffolds. However, existing annulative methods for THF synthesis are rare, limited in scope, and usually require starting materials with large synthetic overhead (Figure 1B).^7^ A general annulative method in which both coupling partners can be easily varied would provide efficient access to a diverse library of polysubstituted THF products.

Our group has previously reported that urea pro-ligands can promote Pd-catalyzed heteroannulation reactions of 2-bromoanilines and 2-bromophenols with 1,3-dienes to access indoline and dihydrobenzofuran products.^8,9^ More recently, we also demonstrated that ureas could promote chainwalking processes for the rapid synthesis of tetrahydroquinolines and benzazepines from 2-bromoanilines and 1,4- or 1,5-dienes.^10^ Notably, the urea ligands were more effective for these transformations than were traditional ligand platforms for Pd(0)/Pd­(II) catalysis. While these methods provide a general approach to access diversely functionalized heterocyclic scaffolds, they still use aromatic ambiphiles, a limitation shared with other existing olefin heteroannulation methods.^11^ The use of non-aromatic ambiphiles would enable the generation of more sp^3^-rich heterocyclic scaffolds, such as THFs. However, this is not a trivial change: aliphatic alcohols are 10^5^ times less acidic than their phenolic counterparts, and the loss of aromaticity removes a conformational biasing element; together these render the key ring-forming nucleophile addition step more challenging for non-aromatic ambiphiles.^12^ Herein, we show that urea-enabled catalysis can overcome this reactivity challenge in olefin heteroannulation (Figure 1C). Using readily accessible starting materials, we can generate a range of diversely substituted THF products. These products can be easily derivatized and mapped directly onto biologically active polyketide natural products.

Using 3-bromo-4-phenylbut-3-en-2-ol **1a** and 1-phenyl-1,3-butadiene **2a** as model substrates, we investigated the effect of the urea ligand structure on the olefin heteroannulation reaction (Figure 2). Without any ligand present, the reaction afforded desired product **3aa** in 30% yield. Simple urea **L1** exerted a slightly negative ligand effect. Monosubstituted ureas were the most effective, with *N*-phenylurea **L2** affording the product in 77% yield. 1,1-Disubstituted aryl urea **L3** gave results similar to those of **L2**, while 1,3-disubstituted urea **L4** afforded the desired product in a lower yield and was comparable to yields with trisubstituted ureas (**L5** and **L6**). Urea **L6** had, in our past work, been the most effective ligand for the heteroannulation of 2-bromophenol and **2a**,^9^ but in this case, **L6** displayed a minimal beneficial ligand effect (38% yield vs 30% without added ligand). We next explored substituent effects among monosubstituted ureas. Varying the electronic properties on the *N*-phenyl substituent (**L7** and **L8**) led to a slight reduction in product yield, as did introducing *ortho* substituents onto the phenyl group (**L9** and **L10**). Other monosubstituted ureas (**L11**–**L13**) were effective but lower yielding. Ultimately, **L2**, which is commercially available and costs <$0.02/mmol, performed the best of the investigated urea ligands and maintained good reactivity across a wide range of ambiphiles and dienes. We revisited our previous heteroannulation reactions^13^ and found that these reaction conditions with **L2** afforded product yields that were either comparable to or better than what we had previously reported,^9,10^ showing the generality of these reaction conditions for urea-enabled heteroannulation.

**Figure 2:**
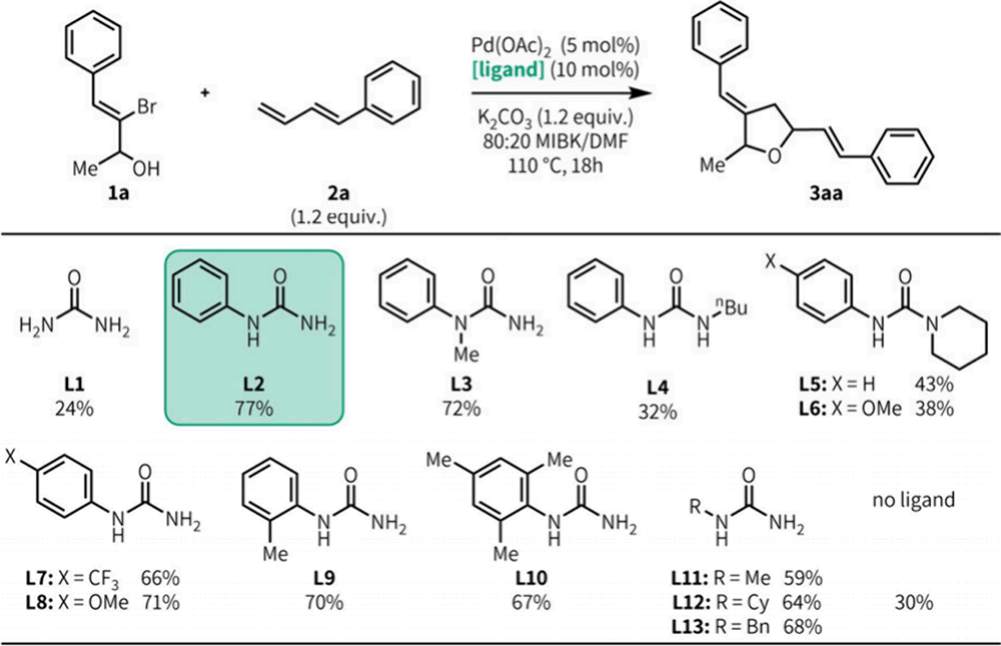
Ligand structure–reactivity relationship studies. Yields were determined by HPLC using nitrobenzene as an internal standard and are an average of three runs. All products have ∼55:45 dr. Reaction conditions: **1a** (0.250 mmol), **2a** (0.300 mmol), Pd­(OAc)_2_ (0.0125 mmol), ligand (0.025 mmol), K_2_CO_3_ (0.300 mmol), and 80:20 MIBK/DMF (0.5 M) at 110 °C for 18 h.

Next, we explored the scope of ambiphiles (Figure 3). Under our optimized conditions, ambiphile **1a** and diene **2a** coupled to provide **3aa** in 72% yield at a 0.5 mmol scale and 66% yield at a 1 g scale (5.0 mmol). The highest product yields were obtained with ambiphiles bearing secondary (**3aa** and **3ba**) and tertiary (**3ea**) alcohols, although excessive steric hindrance adjacent to the alcohol led to poor reactivity (**3ca**, 19%) and the formation of a ketone side product resulting from substrate decomposition. Primary alcohols afforded product in reduced but still reasonable yields (**3da**, 46%). When the vinylic phenyl group was removed, exomethylene-containing products (**3fa** and **3ga**) could be generated directly. While yields were modest for these substrates (29 and 35%, respectively), the ability to engage such unbiased, unactivated substrates is notable; moreover, these products map directly onto a significant class of polyketide natural products.^4^ Other R^2^ substituents were tolerated to varying degrees. Benzodioxolyl (**3ha**) and cyclopropyl (**3ia**) bearing ambiphiles afforded products in reasonable to good yields, while vinyl substitution resulted in poor yields (**3ja**, 17%). Cyclic ambiphiles were less effective than acyclic ambiphiles under our reaction conditions; cyclohexanol substrate **3ka** afforded the corresponding 6,5-bicycle in a modest yield (28%). While aromatic substitution in the R^1^ position was the most effective, alkyl substitution was also tolerated (54 and 37%, **3la** and **3ma**, respectively). The reaction was not very sensitive to the electronic properties of the R^1^ phenyl group (**3na** and **3oa**), and substrates bearing oxygen- and nitrogen-based aromatic heterocycles, including pyridine, could be readily engaged (**3pa** and **3qa**). In all cases with ambiphiles bearing secondary alcohols, the diastereoselectivity was ∼55:45; while poor, this allows for ready access to both THF diastereomers and the future potential for catalyst control over stereochemical outcomes.

**Figure 3:**
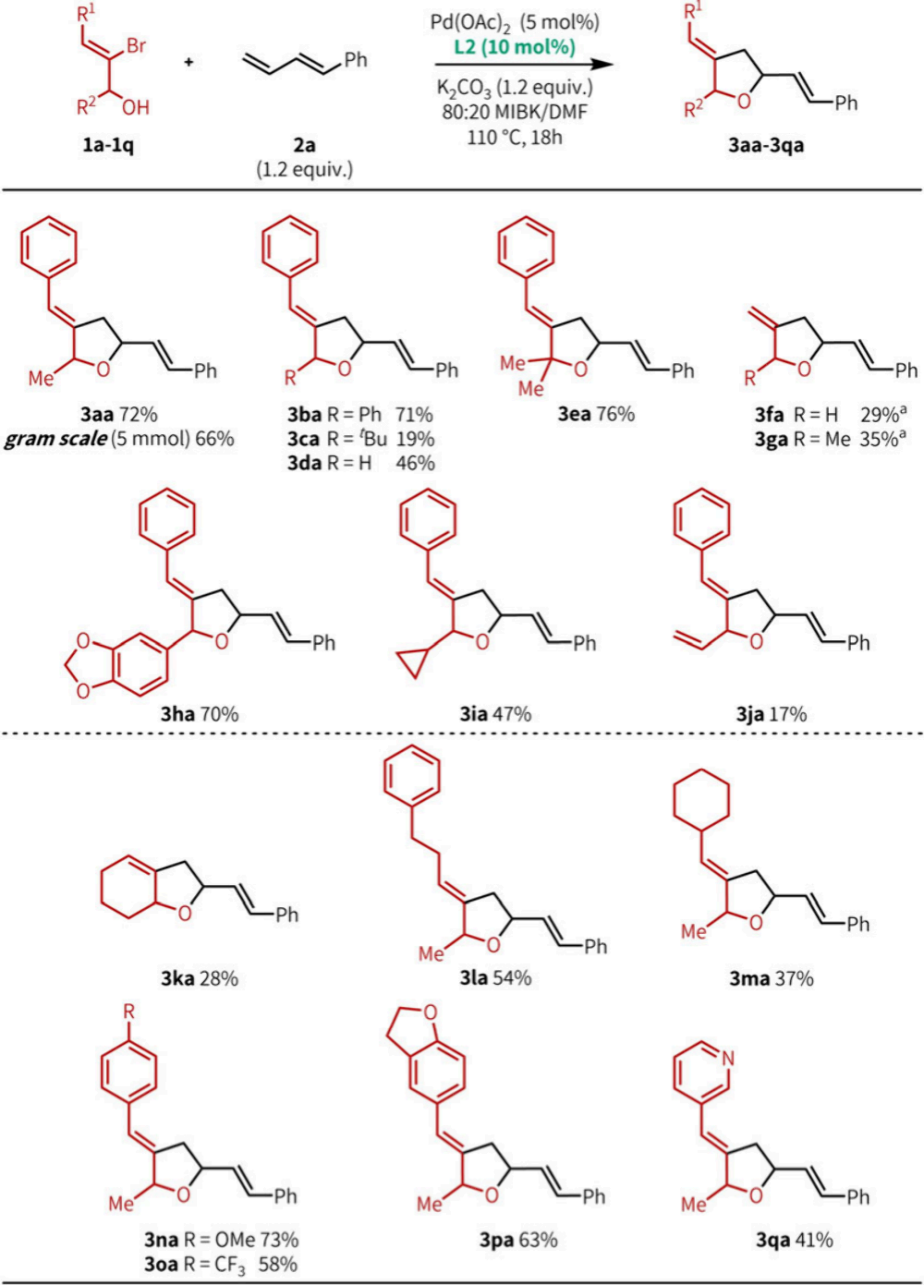
Ambiphile scope. Yields correspond to isolated products and are an average of three runs. All products have ∼55:45 dr. Reaction conditions: **1** (0.500 mmol), **2a** (0.600 mmol), Pd­(OAc)_2_ (0.025 mmol), **L2** (0.050 mmol), K_2_CO_3_ (0.600 mmol), and 80:20 MIBK/DMF (0.5 M) at 110 °C for 18 h. ^*a*^At 80 °C.

As with our previous reports on urea-enabled heteroannulation,^8–10,14^ a structurally and functionally diverse range of diene substrates could be readily engaged in this transformation (Figure 4). Among phenylbutadienes, both electron-donating and electron-withdrawing substitution was well-tolerated (**3ab**–**3ah**), as was *ortho* substitution (**3ai**). Dienes bearing oxygen-, sulfur-, and nitrogen-based aromatic heterocycles were all effective substrates (**3aj**–**3ao**). Aromaticity is not required for good reactivity; alkyl linear dienes (**3ap**, **3aq**, and **3at**) also performed well under the reaction conditions. Similarly, branched and highly substituted dienes, which are typically challenging substrates for diene functionalization reactions,^11b,15^ can be successfully engaged in this reaction (**3ar**–**3au**). 2-Substituted dienes (**3as** and **3at**) allow for the synthesis of fully substituted carbon centers at the 5 position of the THF ring.

**Figure 4:**
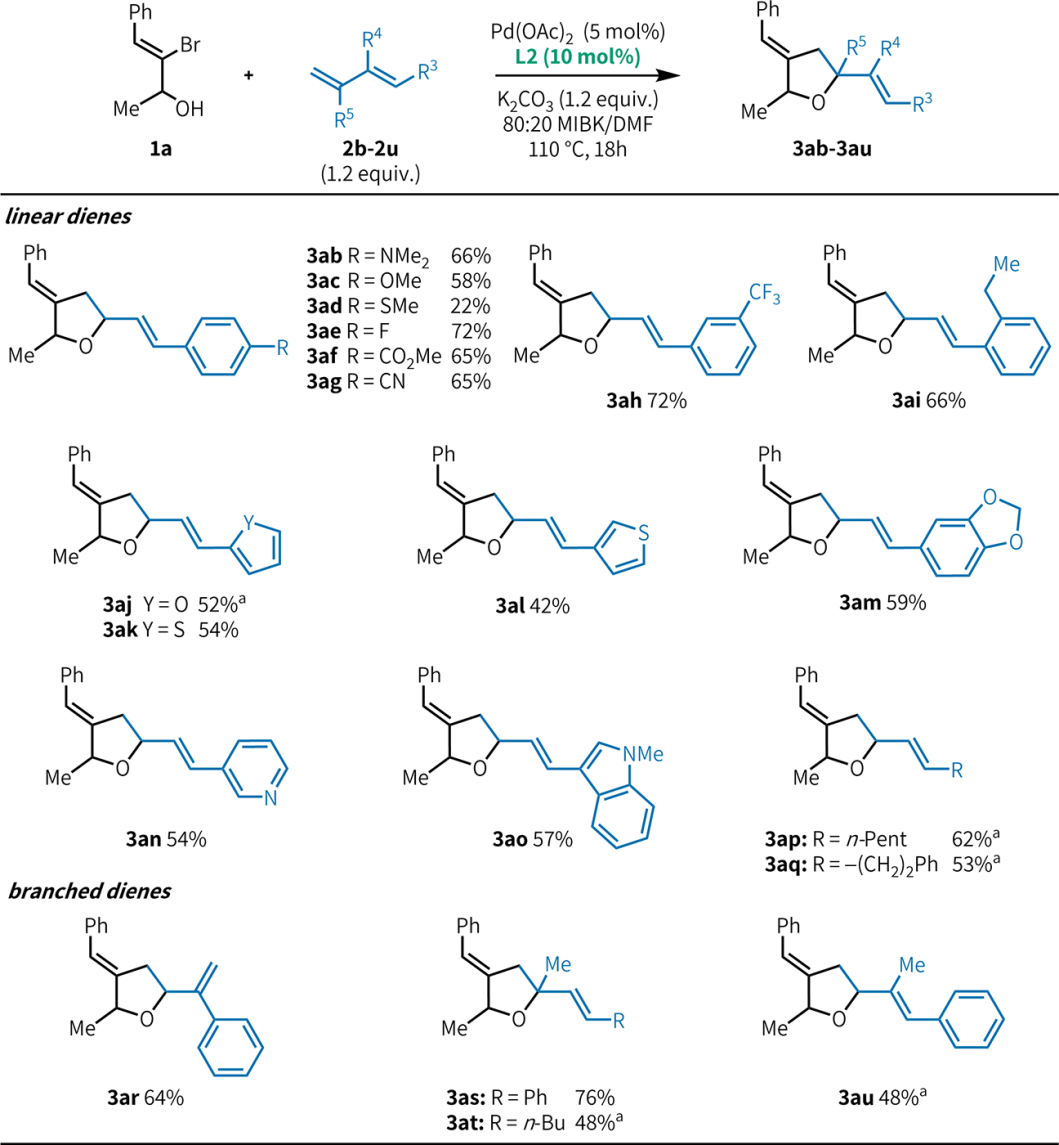
Diene scope. Yields correspond to isolated products and are an average of three runs. All products have ∼55:45 dr. Reaction conditions: **1a** (0.500 mmol), **2** (0.600 mmol), Pd­(OAc)_2_ (0.025 mmol), **L2** (0.050 mmol), K_2_CO_3_ (0.600 mmol), and 80:20 MIBK/DMF (0.5 M) at 110 °C for 18 h. ^*a*^1.5 equiv of diene was used.

Heteroannulation with non-aromatic ambiphile **1a** is sensitive to the stereochemistry of the diene coupling partner;^13^ *Z*-**2a** afforded product in half the yield of *E*-**2a** (35% vs 72% yield; Figure 5A). However, regardless of the starting stereochemistry of the diene, products were isolated as a single alkene stereoisomer (>99:1 *E*/*Z*). On the basis of these findings and support from the literature,^8,11b^ we propose a catalytic cycle involving the intermediacy of a η^3^-allyl species following migratory insertion (Figure 5A). Additionally, likely due to the larger size of **1a** relative to 2-bromophenol, the rate of diene coordination is sensitive to diene stereochemistry, which can cause repulsive steric interactions with the Pd complex. Future mechanistic studies will focus on elucidating the mechanism of nucleophile addition and the specific role of the urea ligand in catalysis.

**Figure 5:**
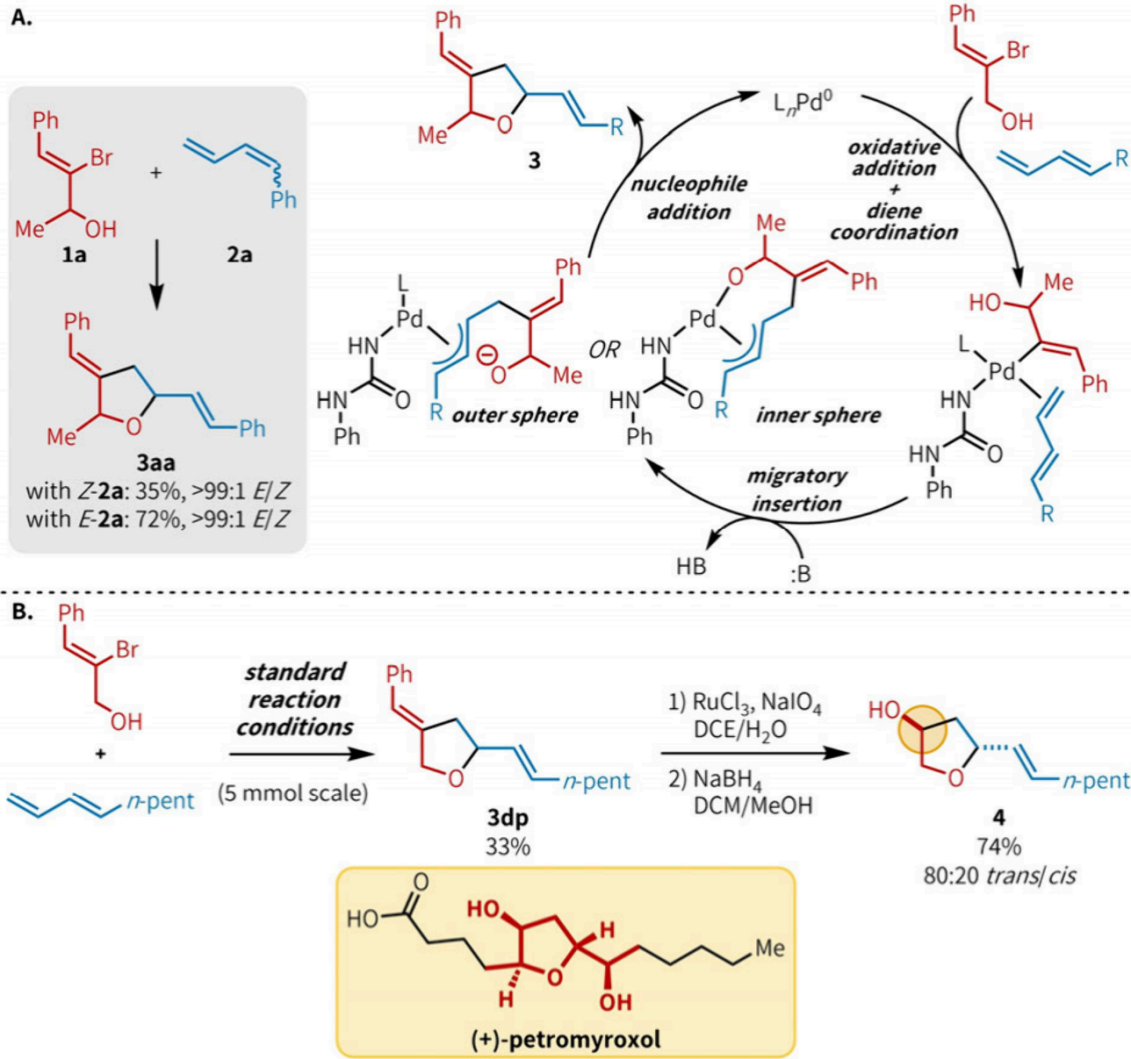
(A) Effect of diene stereochemistry on product yield and plausible catalytic cycle. (B) Rapid generation of hydroxy-THF cores commonly seen in polyketide natural products.

Lastly, we aimed to demonstrate that the polysubstituted THF products obtained through the urea-enabled olefin heteroannulation reaction can be readily transformed into 3-hydroxy-THF cores commonly seen in polyketide natural products, like petromyroxol (Figure 5B).^16,17^ To simplify stereochemical outcomes, we chose to use ambiphile **1d** and diene **2p** under the standard reaction conditions to synthesize 2,5-substituted THF **3dp** in 33% yield. When this heteroannulation product was subjected to a one-pot oxidative cleavage and reduction with NaBH_4_, hydroxy-THF **4** was isolated in 74% yield and 80:20 dr.

We have demonstrated that non-aromatic, acyclic ambiphiles can be engaged in a Pd-catalyzed and urea-enabled heteroannulation approach to synthesize a variety of polysubstituted THF products.^18^ Our method exhibits a broad scope in both ambiphile and diene coupling partners, structurally and functionally, from starting materials that can be easily prepared in 1–3 steps. These products can be easily derivatized and mapped directly onto a variety of THF-containing natural products. Future work will focus on improving the stereocontrol in this reaction and applying our method to the synthesis of valuable bioactive compounds.

## Supplementary Material



## Data Availability

The data underlying this study are available in the published article and its Supporting Information.

## Notes

Additional experimental details, experimental procedures, compound characterization, and NMR spectra for all new compounds (PDF)
