# Supplementary material for: Generation of Polysubstituted Tetrahydrofurans via Urea-Enabled, Pd-Catalyzed Olefin Heteroannulation
Source: Org Lett. 2026 Mar 5;28(11):3589–94. doi: 10.1021/acs.orglett.6c00629 (PMC13010254; doi:10.1021/acs.orglett.6c00629)
Supplement: Supplementary file 1 [file ol6c00629_si_001.pdf]

Generation of Polysubstituted Tetrahydrofurans via Urea-Enabled, Pd-Catalyzed Olefin Heteroannulation

Shannon T. O'Neil, Owen E. Monteferrante, Brooke R. Stanley, Shauna M. Paradine*

Department of Chemistry, University of Rochester, 120 Trustee Road, Rochester, NY 14627

Supporting Information

Table of Contents

General Information 2
  Methods 2
  Materials and Reagents 2
Instrumentation 2
Abbreviations Used 2
Preparation of 2-Bromoallyl Alcohol Ambiphiles 3
Preparation of 1,3-Dienes 18
Preparation of Urea Ligands 31
Reaction Condition Optimization 33
Substrate Scope 54
  2-Bromoallyl Alcohol Scope 54
  1,3-Diene Scope 66
Methodology Limitations 80
Derivatization of THF Products 82
NMR Spectra of New Compounds 83
References 141

General Information

Methods

All reactions were carried out under a N_2 atmosphere in flame–dried glassware with magnetic stir bar unless otherwise specified. Stainless steel gas-tight syringes were used to transfer air- and moisture-sensitive liquids. Reactions were monitored by thin–layer chromatography (TLC) on pre–coated silica gel 60 F254 glass–supported plates from EMD and visualized under UV light (254 nm) or with p–anisaldehyde followed by heating. Flash chromatography was performed using SiliaFlash P60 (230–400 mesh, SiliCycle) using a Combiflash® NextGen 300+ (Teledyne ISCO) or conventional flash columns. Reported product yields were determined based on material isolated after column purification. Room temperature (rt) for the laboratory is 20 °C.

Materials and Reagents

Reagents were used as obtained from commercial suppliers without further purification. Dimethyl sulfoxide (DMSO), dimethylformamide (DMF), methyl isobutyl ketone (MIBK), n-butyl acetate (nBuOAc), benzonitrile, and dichloroethane were purchased from Sigma-Aldrich. Tetrahydrofuran (THF), diethyl ether (Et_2O), methylene chloride (DCM), were purchased from Fisher and dispensed using the Pure Process Technology solvent purification system. ACS grade hexanes, toluene, ethyl acetate, pentane and diethyl ether were used for column chromatography. Thin-layer chromatography (TLC) was performed on precoated silica gel 60 F254 glass-supported plates from EMD, and visualization was performed with a UV lamp followed by staining with p-anisaldehyde followed by heating. Silica gel chromatography purifications were carried out using SiliCycle Silica SiliaFlash P60 230-400 mesh.

Instrumentation

Proton nuclear magnetic resonance (^1H NMR) and proton-decoupled carbon nuclear magnetic resonance (^{13}C, ^1H NMR) spectra were recorded on a Bruker DPX–400 or a JEOL JNM–ECZL S instrument (operating at 400 MHz for ^1H, 100 MHz for ^{13}C) or a Bruker DPX–500 or JEOL JNM–ECZL R instrument (operating at 500 MHz for ^1H, 125 MHz for ^{13}C) at ambient temperature. Proton resonances are referenced to residual protium in the NMR solvent. Carbon resonances are referenced to the carbon resonances of the NMR solvent. Data are represented as follows: chemical shift, multiplicity (br = broad, s = singlet, d = doublet, dd = doublet of doublets, t = triplet, q = quartet, m = multiplet, app = apparent), coupling constants (J) in Hertz (Hz), integration. Mass spectral (MS) data were obtained on a Thermo Fisher Q Exactive Plus spectrometer (University of Rochester Medical Center Mass Spectrometry Resource Laboratory) using an Orbitrap mass analyzer. High-performance liquid chromatography (HPLC) analysis was performed using a Shimadzu Prominence–I LC–2030 Plus system with commercially available Restek Pinnacle DB Cyano column (5 µm, 150x4.6 mm). All samples were eluted with 2% iPrOH/hexanes at an elution rate of 1 mL/min and detected at 254 nm. Total run time was 6 min.

Abbreviations Used

aq. = aqueous, cm–1 = wavenumber, DCM = dichloromethane, DMF = N,N-dimethylformamide, DMSO = dimethyl sulfoxide, d.r. = diastereomeric ratio, equiv. = equivalents, h = hours, nBuOAc = nbutyl acetate, min = m, m/z = mass to charge ratio, NMP = *N*-Methylpyrrolidone, rt = room temperature, sat = saturated, THF = tetrahydrofuran, Tol = toluene

Preparation of 2-Bromoallyl Alcohol Ambiphiles

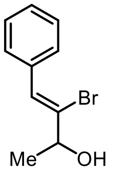

1a

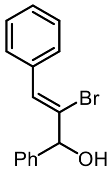

1b

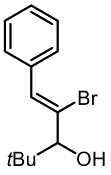

1c

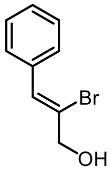

1d

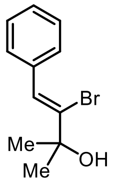

1e

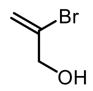

1f

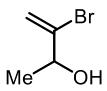

1g

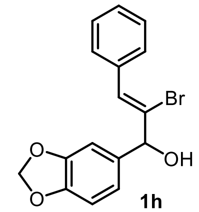

1h

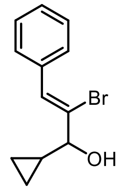

1i

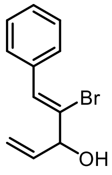

1j

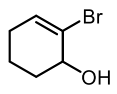

1k

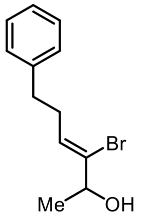

1l

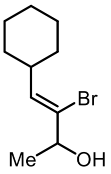

1m

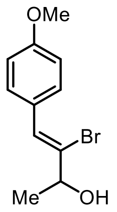

1n

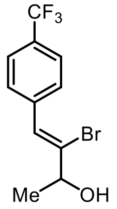

1o

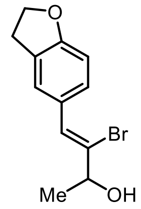

1p

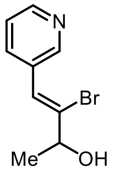

1q

2-Bromoallyl alcohol ambiphile **1f** was purchased from Ambeed and all other were synthesized as described below.

Ambiphile Synthesis Procedure A:

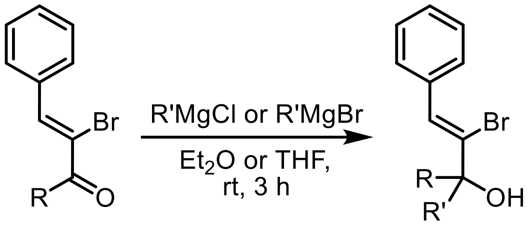

Prepared according to a modified literature procedure.[1] To a solution of bromoaldehyde or ketone (5 mmol, 1.0 equiv.) in 8 mL of dry Et_2O or dry THF, a solution of Grignard reagent in Et_2O/THF (15 mmol, 3 equiv.) at 0 °C was added slowly dropwise under a nitrogen balloon. After stirring 3 h at rt, saturated NH_4Cl was added, and the organic phase was extracted with Et_2O and washed with brine. The combined organic phases were dried over $MgSO_4$. Solvent was removed under reduced pressure and purified by column chromatography on silica gel (10-20% EtOAc in hexanes) to provide product.

(*Z*)-3-bromo-4-phenylbut-3-en-2-ol (1a)

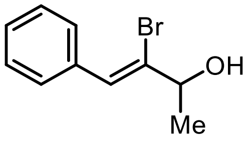

Prepared according to ambiphile synthesis procedure A. (*Z*)-2-bromo-3-phenylacrylaldehyde (1.05 g, 5.00 mmol, 1.00 equiv.) was measured into an oven-dried round bottom flask equipped with a stir bar and septum. The flask was evacuated and refilled with N_2 three times before the addition of dry Et_2O (8 mL, 0.6 M). The solution was then cooled to 0 °C. MeMgBr in Et_2O (3.0 M) was then added (5.00 mL, 15.00 mmol, 3.00 equiv.). The solution was allowed to warm to rt (20 °C) and allowed to stir for 3 h. Upon completion, the solution was quenched with NH_4Cl (20 mL). The organic layer was extracted with Et_2O (3 x 20 mL) and washed with brine (3 X 20 mL), then dried over $MgSO_4$. Solvent was removed under reduced pressure. Crude material was purified via column chromatography on SiO_2 using 10-20% EtOAc in hexanes to afford (**1a**) as a light-yellow oil (95%, 998 mg, 4.75 mmol). Spectral data agree with that reported in the literature.[2]

^{1}H NMR (400 MHz, $CDCl_3$) δ 7.61 (d, J = 7.0 Hz, 2H), 7.40 – 7.29 (m, 3H), 7.08 (s, 1H), 4.50 (q, J = 6.3 Hz, 1H), 2.14 (d, J = 5.8 Hz, 1H), 1.49 (d, J = 6.3 Hz, 3H)

(*Z*)-2-bromo-1,3-diphenylprop-2-en-1-ol (1b)

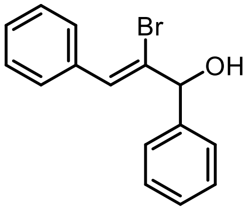

Prepared according to ambiphile synthesis procedure A. (*Z*)-2-bromo-3-phenylacrylaldehyde (1.05 g, 5.00 mmol, 1.00 equiv.) was measured into an oven-dried round bottom flask equipped with a stir bar and septum. The flask was evacuated and refilled with N_2 three times before the addition of dry THF (8 mL, 0.6 M). The solution was then cooled to 0 °C. PhMgBr in THF (1.0 M) was then added (15.00 mL, 15.00 mmol, 3.00 equiv.). The solution was allowed to warm to rt (20 °C) and allowed to stir for 3 h. Upon completion, the solution was quenched with NH_4Cl (20 mL). The organic layer was extracted with Et_2O (3 x 20 mL) and washed with brine (3 X 20 mL), then dried over $MgSO_4$. Solvent was removed under reduced pressure. Crude material was purified via column chromatography on SiO_2 using 10-20% EtOAc in hexanes to afford (**1b**) as a light-yellow oil (67%, 969 mg, 3.35 mmol).

^{1}H NMR (500 MHz, $CDCl_3$) δ 7.66 (d, J = 7.7 Hz, 2H), 7.50 (d, J = 7.7 Hz, 2H), 7.44 – 7.32 (m, 6H), 7.29 (s, 1H), 5.47 (s, 1H), 2.63 (s, 1H)

^{13}C NMR (100 MHz, $CDCl_3$) δ 140.6, 135.1, 129.3, 128.8, 128.7, 128.7, 128.4, 128.4, 128.3, 126.9, 79.3

HRMS (ESI) m/z: $[M + H]^+$ Calcd for $C_{15}H_{13}BrO$ 289.0228; Found 289.0243.

(*Z*)-2-bromo-4,4-dimethyl-1-phenylpent-1-en-3-ol (1c)

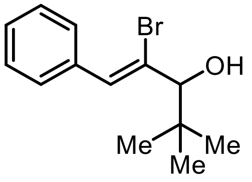

Prepared according to ambiphile synthesis procedure A. (*Z*)-2-bromo-3-phenylacrylaldehyde (1.05 g, 5.00 mmol, 1.00 equiv.) was measured into an oven-dried round bottom flask equipped with a stir bar and septum. The flask was evacuated and refilled with N_2 three times before the addition of dry THF (8 mL, 0.6 M). The solution was then cooled to 0 °C. *t*BuMgCl in THF (1.0 M) was then added (15.00 mL, 15.00 mmol, 3.00 equiv.). The solution was allowed to warm to rt (20 °C) and allowed to stir for 3 h. Upon completion, the solution was quenched with NH_4Cl (20 mL). The organic layer was extracted with Et_2O (3 x 20 mL) and washed with brine (3 X 20 mL), then dried over $MgSO_4$. Solvent was removed under reduced pressure. Crude material was purified via column chromatography on SiO_2 using 10-20% EtOAc in hexanes to afford (**1c**) as a light-yellow oil (35%, 471 mg, 1.75 mmol).

^1H NMR (400 MHz, $CDCl_3$) δ 7.62 (d, J = 7.5 Hz, 2H), 7.39 (t, J = 8.0 Hz, 2H), 7.33 (t, J = 7.5 Hz, 1H), 7.04 (s, 1H), 4.11 (d, J = 5.0 Hz, 1H), 2.36 (d, J = 6.0 Hz, 1H), 1.10 (s, 9H)

^{13}C NMR (100 MHz, $CDCl_3$) δ 135.5, 130.6, 129.3, 128.3, 128.2, 127.6, 84.4, 36.5, 26.9

HRMS (ESI) m/z: $[M + H]^+$ Calcd for $C_{13}H_{17}BrO$ 269.0541; Found 269.0538.

(*Z*)-2-bromo-1-phenylpenta-1,4-dien-3-ol (1j)

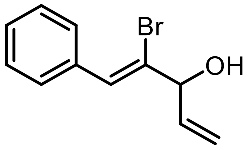

Prepared according to ambiphile synthesis procedure A. (*Z*)-2-bromo-3-phenylacrylaldehyde (1.05 g, 5.00 mmol, 1.00 equiv.) was measured into an oven-dried round bottom flask equipped with a stir bar and septum. The flask was evacuated and refilled with N_2 three times before the addition of dry THF (8 mL, 0.6 M). The solution was then cooled to 0 °C. ViMgBr in THF (1.0 M) was then added (15.00 mL, 15.00 mmol, 3.00 equiv.). The solution was allowed to warm to rt (20 °C) and allowed to stir for 3 h. Upon completion, the solution was quenched with NH_4Cl (20 mL). The organic layer was extracted with Et_2O (3 x 20 mL) and washed with brine (3 X 20 mL), then dried over $MgSO_4$. Solvent was removed under reduced pressure. Crude material was purified via column chromatography on SiO_2 using 10-20% EtOAc in hexanes to afford (**1j**) as a light-yellow oil (59%, 705 mg, 2.95 mmol).

^1H NMR (400 MHz, $CDCl_3$) δ 7.63 (d, J = 7.3 Hz, 2H), 7.40 – 7.29 (m, 3H), 7.14 (s, 1H), 6.06 – 5.97 (m, 1H), 5.49 (d, J = 17.2 Hz, 1H), 5.36 (d, J = 10.3 Hz, 1H), 4.85 (t, J = 5.9 Hz, 1H), 2.31 (d, J = 6.2 Hz, 1H)

^{13}C NMR (100 MHz, $CDCl_3$) δ 137.3, 135.0, 129.3, 128.7, 128.4, 128.3, 128.2, 117.4, 78.2

HRMS (ESI) m/z: $[M + H]^+$ Calcd for $C_{11}H_{11}BrO$ 239.0071; Found 239.0070.

Ambiphile Synthesis Procedure B:

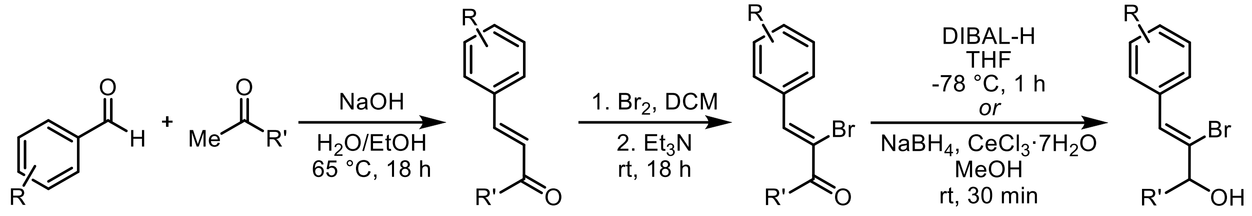

Condensation for Enone Synthesis

Prepared according to a modified literature procedure.[3] Sodium hydroxide (3.00 g, 75 mmol, 5.0 equiv.) added to a mixture of H_2O/EtOH (30 mL/45 mL) and stirred at rt until fully dissolved. Ketone (15 mmol, 1.0 equiv.) followed by aldehyde (15 mmol, 1.0 equiv.) were added to the reaction. This reaction mixture was then stirred and heated to 65 °C in an oil bath and monitored by TLC. After 18 h or when no starting materials remained, the reaction was cooled to rt, quenched with 1M HCl and the organic phase was extracted with EtOAc and washed with brine. The combined organic phases were dried over $MgSO_4$. Solvent was removed under reduced pressure and purified by column chromatography on silica gel (10-20% EtOAc in hexanes) to provide product.

Enone Bromination

Prepared according to a modified literature procedure.[4] Enone (10 mmol, 1.0 equiv.) was dissolved in 30 mL of DCM. This solution was cooled to 0 °C and stirred and Br_2 (615 µL, 12 mmol, 1.2 equiv.) was added slowly dropwise. After stirring 1h at rt, Et_3N (2.4 mL, 17 mmol, 1.7 equiv.) was added to the reaction mixture and 10 mL DCM was added. This mixture then stirred at rt overnight or until no dibrominated intermediate was detected by TLC. Once completed the reaction was quenched with 2M HCl and the organic phase was extracted with DCM and washed with sodium thiosulfate and brine. The combined organic phases were dried over $MgSO_4$. Solvent was removed under reduced pressure and purified by column chromatography on silica gel (10-20% EtOAc in hexanes) to provide product.

Reduction to Alcohol – DIBAL-H

Prepared according to a modified literature procedure.[5] To a solution of brominated ketone or aldehyde (5 mmol, 1.0 equiv.) in 15 mL dry THF, a solution of DIBAL-H reagent in toluene (10 mmol, 2 equiv.) at -78 °C was added slowly dropwise under a nitrogen balloon. After stirring 1 hour at -78 °C or once no enone remained via TLC, saturated NH_4Cl was added, and the organic phase was extracted with Et_2O and washed with brine. The combined organic phases were dried over $MgSO_4$. Solvent was removed under reduced pressure and purified by column chromatography on silica gel (10-20% EtOAc in hexanes) to provide product.

Reduction to Alcohol – $NaBH_4$

Prepared according to a modified literature procedure.[6] A solution of brominated ketone or aldehyde (5 mmol, 1.0 equiv.) and $CeCl_3 \cdot 7H_2O$ (1.66 g, 5.25 mmol, 1.05 equiv.) in 43 mL of MeOH was stirred at rt until all solids were dissolved. Sodium borohydride (227 mg, 6 mmol, 1.2 equiv.) was then added slowly in small portions. After stirring 30 min at rt, the reaction was quenched with 2M HCl. The organic layer was extracted with Et_2O and washed with brine then dried over $MgSO_4$. Solvent was removed under reduced pressure and purified by column chromatography on silica gel (10-20% EtOAc in hexanes) to provide product.

(*Z*)-2-bromo-3-phenylprop-2-en-1-ol (1d)

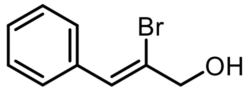

Prepared according to a modified literature procedure.[6] A solution of (*Z*)-2-bromo-3-phenylacrylaldehyde (1.05 g, 5 mmol, 1.00 equiv.) and $CeCl_3 \cdot 7H_2O$ (1.66 g, 5.25 mmol, 1.05 equiv.) in 43 mL of MeOH was stirred at rt until all solids were dissolved. Sodium borohydride (227 mg, 6 mmol, 1.2 equiv.) was then added slowly in small portions. After stirring 30 min at rt, the reaction was quenched with 2M HCl (20 mL). The organic layer was extracted with Et_2O (3 x 20 mL) and washed with brine (3 X 20 mL), then dried over $MgSO_4$. Solvent was removed under reduced pressure. Crude material was purified via column chromatography on SiO_2 using 10-20% EtOAc in hexanes to afford (**1d**) as a light-yellow oil (96%, 1.02 g, 4.80 mmol). Spectral data agree with that reported in the literature.[6]

^{1}H NMR (500 MHz, $CDCl_3$) δ 7.62 (d, J = 7.7 Hz, 2H), 7.40 – 7.35 (m, 2H), 7.32 (t, J = 7.5 Hz, 1H), 7.09 (s, 1H), 4.42 (s, 1H), 2.48 (bs, 1H)

(*Z*)-3-bromo-4-phenylbut-3-en-2-one (S1)

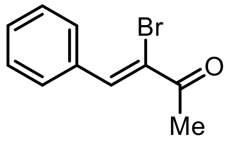

Prepared according to a modified literature procedure.[4] (*E*)-4-Phenylbut-3-en-2-one (1.46 g, 10 mmol, 1.0 equiv.) was dissolved in 30 mL of DCM. This solution was cooled to 0 °C and stirred and Br_2 (615 μL, 12 mmol, 1.2 equiv.) was added slowly dropwise. After stirring 1h at rt, Et_3N (2.4 mL, 17 mmol, 1.7 equiv.) was added to the reaction mixture and 5 mL DCM was added. This mixture then stirred at rt overnight or until no dibrominated intermediate was detected by TLC. Once completed the reaction was quenched with 2M HCl and the organic phase was extracted with DCM and washed with sodium thiosulfate and brine. The combined organic phases were dried over $MgSO_4$. Solvent was removed under reduced to afford (**S1**) as a yellow oil (68%, 1.52 g, 6.76 mmol). Crude material was used without purification for the next step.

(*Z*)-3-bromo-2-methyl-4-phenylbut-3-en-2-ol (1e)

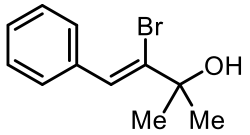

Prepared according to ambiphile synthesis procedure A. (*Z*)-3-Bromo-4-phenylbut-3-en-2-one (**S1**) (1.13 g, 5.00 mmol, 1.00 equiv.) was measured into an oven-dried round bottom flask equipped with a stir bar and septum. The flask was evacuated and refilled with N_2 three times before the addition of dry Et_2O (8 mL, 0.6 M). The solution was then cooled to 0 °C. MeMgBr in Et_2O (3.0 M) was then added (5.00 mL, 15.00 mmol, 3.00 equiv.). The solution was allowed to warm to rt (20 °C) and allowed to stir for 3 h. Upon completion, the solution was quenched with NH_4Cl (20 mL). The organic layer was extracted with Et_2O (3 x 20 mL) and washed with brine (3 X 20 mL), then dried over $MgSO_4$. Solvent was removed under reduced pressure. Crude material was purified via column chromatography on SiO_2 using 10-20% EtOAc in hexanes to afford (**1e**) as a light-yellow oil (85%, 1.03 g, 4.26 mmol).

^{1}H NMR (500 MHz, $CDCl_3$) δ 7.54 (d, J = 7.0 Hz, 2H), 7.36 (t, J = 7.5 Hz, 2H), 7.30 (t, J = 7.0 Hz, 1H), 7.17 (s, 1H), 2.13 (s, 1H), 1.60 (s, 6H)

^{13}C NMR (125 MHz, $CDCl_3$) δ 136.1, 135.9, 129.2, 128.2, 127.9, 125.6, 74.9, 29.3

HRMS (ESI) m/z: $[M + H]^+$ Calcd for $C_{11}H_{13}BrO$ 241.0228; Found 241.0213.

3-bromobut-3-en-2-one (S2)

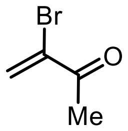

Prepared according to a modified literature procedure.[4] But-3-en-2-one (700 mg, 10 mmol, 1.0 equiv.) was dissolved in 30 mL of DCM. This solution was cooled to 0 °C and stirred and Br_2 (615 µL, 12 mmol, 1.2 equiv.) was added slowly dropwise. After stirring 1h at rt, Et_3N (2.4 mL, 17 mmol, 1.7 equiv.) was added to the reaction mixture and 5 mL DCM was added. This mixture then stirred at rt overnight or until no dibrominated intermediate was detected by TLC. Once completed the reaction was quenched with 2M HCl and the organic phase was extracted with DCM and washed with sodium thiosulfate and brine. The combined organic phases were dried over $MgSO_4$. Solvent was removed under reduced to afford (**S2**) as a yellow oil (77%, 1.15 g, 7.73 mmol). Crude material was used without purification for the next step.

3-bromobut-3-en-2-ol (1g)

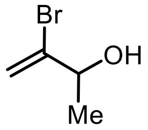

Prepared according to a modified literature procedure.[6] A solution of 3-bromobut-3-en-2-one (**S2**) (745 mg, 5.00 mmol, 1.00 equiv.) and $CeCl_3{\cdot}7H_2O$ (1.66 g, 5.25 mmol, 1.05 equiv.) in 43 mL of MeOH was stirred at rt until all solids were dissolved. Sodium borohydride (227 mg, 6 mmol, 1.2 equiv.) was then added slowly in small portions. After stirring 30 min at rt, the reaction was quenched with 2M HCl (20 mL). The organic layer was extracted with Et_2O (3 x 20 mL) and washed with brine (3 X 20 mL), then dried over $MgSO_4$. Solvent was removed under reduced pressure. Crude material was purified via column chromatography on SiO_2 using 10-20% EtOAc in hexanes to afford (**1g**) as a colorless oil (31%, 234 mg, 1.55 mmol).

^{1}H NMR (500 MHz, $CDCl_3$) δ 5.89 (s, 1H), 5.52 (s, 1H), 4.32 (q, J = 6.44 Hz, 1H), 1.94 (d, J = 5.69 Hz, 1H), 1.39 (d J = 6.29 Hz, 3H)

^{13}C NMR (125 MHz, CDCl3) δ 138.6, 116.0, 72.2, 22.2

HRMS (ESI) m/z: $[M + Na]^+$ Calcd for C_4H_7BrO 172.9570; Found 172.9568.

(*E*)-1-(benzo[d][1,3]dioxol-5-yl)-3-phenylprop-2-en-1-one (S3)

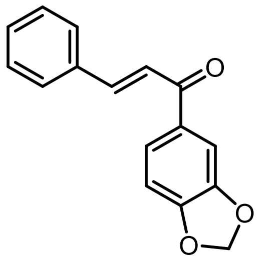

Prepared according to a modified literature procedure.[3] Sodium hydroxide (3.00 g, 75 mmol, 5.0 equiv.) added to a mixture of H_2O/EtOH (30 mL/45 mL) and stirred at rt until fully dissolved. 1-(Benzo[d][1,3]dioxol-5-yl)ethan-1-one (2.46 g, 15 mmol, 1.0 equiv.) followed by benzaldehyde (1.59 g, 15 mmol, 1.0 equiv.) were added to the reaction. This reaction mixture was then stirred and heated to 65 °C in an oil bath and monitored by TLC. After 18 h or when no starting materials remained, the reaction was cooled to rt, quenched with 1M HCl (20 mL). The organic layer was extracted with EtOAc (3 x 20 mL) and washed with brine (3 X 20 mL), then dried over $MgSO_4$. Solvent was removed under reduced pressure. Crude material was purified via column chromatography on SiO_2 using 10-20% EtOAc in hexanes to afford (**S3**) as a yellow oil (84%, 3.18 g, 12.62 mmol). Spectral data agree with that reported in the literature.[7]

^{1}H NMR (500 MHz, $CDCl_3$) δ 7.80 (d, J = 15.5 Hz, 1H), 7.67 – 7.62 (m, 3H), 7.54 (d, J = 1.5 Hz, 1H), 7.50 (d, J = 15.5 Hz, 1H) 7.44 – 7.39 (m, 3H), 6.90 (d, J = 8.0 Hz, 1H), 6.07 (s, 2H)

(*Z*)-1-(benzo[d][1,3]dioxol-5-yl)-2-bromo-3-phenylprop-2-en-1-one (S4)

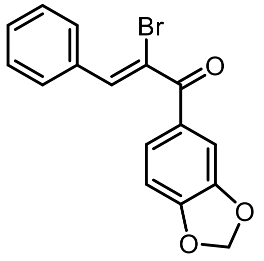

Prepared according to a modified literature procedure.[4] (*E*)-1-(benzo[d][1,3]dioxol-5-yl)-3-phenylprop-2-en-1-one (**S3**) (700 mg, 10 mmol, 1.0 equiv.) was dissolved in 30 mL of DCM. This solution was cooled to 0 °C and stirred and Br_2 (615 µL, 12 mmol, 1.2 equiv.) was added slowly dropwise. After stirring 1h at rt, Et_3N (2.4 mL, 17 mmol, 1.7 equiv.) was added to the reaction mixture and 5 mL DCM was added. This mixture then stirred at rt overnight or until no dibrominated intermediate was detected by TLC. Once completed the reaction was quenched with 2M HCl and the organic phase was extracted with DCM and washed with sodium thiosulfate and brine. The combined organic phases were dried over $MgSO_4$. Solvent was removed under reduced to afford (**S4**) as a yellow oil (72%, 2.37 g, 7.15 mmol). Crude material was used without purification for the next step.

(*Z*)-1-(benzo[d][1,3]dioxol-5-yl)-2-bromo-3-phenylprop-2-en-1-ol (1h)

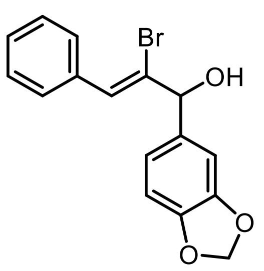

Prepared according to a modified literature procedure.[6] A solution of (*Z*)-1-(benzo[d][1,3]dioxol-5-yl)-2-bromo-3-phenylprop-2-en-1-one (1.66 g, 5.00 mmol, 1.00 equiv.) and $CeCl_3 \cdot 7H_2O$ (1.66 g, 5.25 mmol, 1.05 equiv.) in 43 mL of MeOH was stirred at rt until all solids were dissolved. Sodium borohydride (227 mg, 6 mmol, 1.2 equiv.) was then added slowly in small portions. After stirring 30 min at rt, the reaction was quenched with 2M HCl (20 mL). The organic layer was extracted with Et_2O (3 x 20 mL) and washed with brine (3 X 20 mL), then dried over $MgSO_4$. Solvent was removed under reduced pressure. Crude material was purified via column chromatography on SiO_2 using 10-20% EtOAc in hexanes to afford (**1h**) as a yellow oil (63%, 1.05 g, 3.15 mmol).

^1H NMR (400 MHz, $CDCl_3$) δ 7.65 (d, J = 11.0 Hz, 2H), 7.41 – 7.30 (m, 3H), 7.27 (s, 1H), 6.96 (d, J = 9.6 Hz, 2H), 6.82 (d, J = 7.9 Hz, 1H), 5.98 (s, 2H), 5.37 (d, J = 5.2 Hz,1H), 2.48 (d, J = 5.3 Hz, 1H)

^{13}C NMR (100 MHz, $CDCl_3$) δ 148.0, 147.7, 135.0, 134.5, 129.3, 128.6, 128.5 (2C), 128.4, 120.6, 108.3, 107.3, 101.4, 79.0

HRMS (ESI) m/z: $[M + H]^+$ Calcd for $C_{16}H_{13}BrO_3$ 333.0126; Found 333.0099.

(*E*)-1-cyclopropyl-3-phenylprop-2-en-1-one (S5)

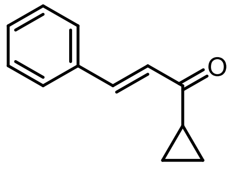

Prepared according to a modified literature procedure.[3] Sodium hydroxide (3.00 g, 75 mmol, 5.0 equiv.) added to a mixture of H_2O/EtOH (30 mL/45 mL) and stirred at rt until fully dissolved. 1-Cyclopropylethan-1-one (1.26 g, 15 mmol, 1.0 equiv.) followed by benzaldehyde (1.59 g, 15 mmol, 1.0 equiv.) were added to the reaction. This reaction mixture was then stirred and heated to 65 °C in an oil bath and monitored by TLC. After 18 h or when no starting materials remained, the reaction was cooled to rt, quenched with 1M HCl (20 mL). The organic layer was extracted with EtOAc (3 x 20 mL) and washed with brine (3 X 20 mL), then dried over $MgSO_4$. Solvent was removed under reduced pressure. Crude material was purified via column chromatography on SiO_2 using 10-20% EtOAc in hexanes to afford (**S5**) as a yellow oil (80%, 2.07 g, 12.03 mmol). Spectral data agree with that reported in the literature.[8]

^1H NMR (400 MHz, $CDCl_3$) δ 7.62 (d, J = 16.4 Hz, 1H), 7.59 – 7.55 (m, 2H), 7.42 – 7.36 (m, 3H), 6.88 (d, J = 16.0 Hz, 1H), 2.30 – 2.21 (m, 1H), 1.20 – 1.13 (m, 2H), 1.02 – 0.94 (m, 2H)

(*Z*)-2-bromo-1-cyclopropyl-3-phenylprop-2-en-1-one (S6)

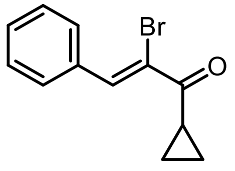

Prepared according to a modified literature procedure.[4] (*E*)-1-cyclopropyl-3-phenylprop-2-en-1-one (**S5**) (1.72 g, 10 mmol, 1.0 equiv.) was dissolved in 30 mL of DCM. This solution was cooled to 0 °C and stirred and Br_2 (615 µL, 12 mmol, 1.2 equiv.) was added slowly dropwise. After stirring 1h at rt, Et_3N (2.4 mL, 17 mmol, 1.7 equiv.) was added to the reaction mixture and 5 mL DCM was added. This mixture then stirred at rt overnight or until no dibrominated intermediate was detected by TLC. Once completed the reaction was quenched with 2M HCl and the organic phase was extracted with DCM and washed with sodium thiosulfate and brine. The combined organic phases were dried over $MgSO_4$. Solvent was removed under reduced to afford (**S6**) as a yellow oil (96%, 2.41 g, 9.58 mmol). Crude material was used without purification for the next step.

(*Z*)-2-bromo-1-cyclopropyl-3-phenylprop-2-en-1-ol (1i)

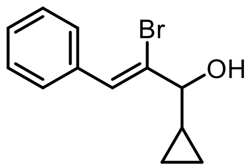

Prepared according to a modified literature procedure.[5] To a solution of (*Z*)-2-bromo-1-cyclopropyl-3-phenylprop-2-en-1-one (**S6**) (1.26 g, 5 mmol, 1.0 equiv.) in 15 mL dry THF, a solution of DIBAL-H reagent in toluene (10 mmol, 2 equiv.) at -78 °C was added slowly dropwise under a nitrogen balloon. After stirring 1 hour at -78 °C or once no enone remained via TLC, saturated NH_4Cl (20mL) was added to quench the reaction. The organic layer was extracted with Et_2O (3 x 20 mL) and washed with brine (3 X 20 mL), then dried over $MgSO_4$. Solvent was removed under reduced pressure. Crude material was purified via column chromatography on SiO_2 using 10-20% EtOAc in hexanes to afford (**1i**) as a colorless oil (69%, 873 mg, 3.45 mmol).

^{1}H NMR (500 MHz, DMSO-d6) δ 7.64 – 7.61 (m, 2H), 7.40 – 7.36 (m, 2H), 7.32 (tt, J = 7.5, 1.3 Hz, 1H), 7.16 (s, 1H), 5.53 (d, J = 4.6 Hz, 1H), 3.62 (dd, J = 7.3, 4.3 Hz, 1H), 1.18 – 1.11 (m, 1H), 0.53 – 0.41 (m, 2H), 0.34 – 0.30 (m, 1H)

^{13}C NMR (100 MHz, DMSO-d6) δ 132.3, 131.6, 128.9, 128.3, 127.9, 126.2, 78.9, 16.6, 3.3, 2.6

HRMS (ESI) m/z: $[M + H]^+$ Calcd for $C_{12}H_{13}BrO$ 253.0028; Found 253.0024.

(*E*)-4-cyclohexylbut-3-en-2-one (S7)

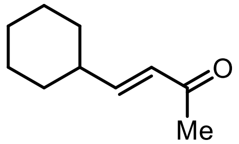

Prepared according to a modified literature procedure.[3] Sodium hydroxide (3.00 g, 75 mmol, 5.0 equiv.) added to a mixture of H_2O/EtOH (30 mL/45 mL) and stirred at rt until fully dissolved. Acetone (1.1 mL, 15 mmol, 1.0 equiv.) followed by cyclohexanecarbaldehyde (1.68 g, 15 mmol, 1.0 equiv.) were added to the reaction. This reaction mixture was then stirred and heated to 65 °C in an oil bath and monitored by TLC. After 18 or when no starting materials remained, the reaction was cooled to rt, quenched with 1M HCl (20 mL). The organic layer was extracted with EtOAc (3 x 20 mL) and washed with brine (3 X 20 mL), then dried over $MgSO_4$. Solvent was removed under reduced pressure. Crude material was purified via column chromatography on SiO_2 using 10-20% EtOAc in hexanes to afford (**S7**) as a light-yellow oil (77%, 1.76 g, 11.55 mmol). Spectral data agree with that reported in the literature.[9]

^{1}H NMR (400 MHz, $CDCl_3$) δ 6.71 (dd, J = 16.0, 6.4 Hz, 1H), 6.00 (d, J = 16.0 Hz, 1H), 2.23 (s, 3H), 2.18 – 2.07 (m, 1H), 1.80 – 1.62 (m, 5H), 1.35 – 1.06 (m, 5H)

(*Z*)-3-bromo-4-cyclohexylbut-3-en-2-one (S8)

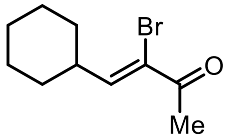

Prepared according to a modified literature procedure.[4] (*E*)-4-cyclohexylbut-3-en-2-one (**S7**) (1.52 g, 10 mmol, 1.0 equiv.) was dissolved in 30 mL of DCM. This solution was cooled to 0 °C and stirred and Br_2 (615 μL, 12 mmol, 1.2 equiv.) was added slowly dropwise. After stirring 1h at rt, Et_3N (2.4 mL, 17 mmol, 1.7 equiv.) was added to the reaction mixture and 5 mL DCM was added. This mixture then stirred at rt overnight or until no dibrominated intermediate was detected by TLC. Once completed the reaction was quenched with 2M HCl and the organic phase was extracted with DCM and washed with sodium thiosulfate and brine. The combined organic phases were dried over $MgSO_4$. Solvent was removed under reduced to afford (**S8**) as a yellow oil (49%, 1.13 g, 4.89 mmol). Crude material was used without purification for the next step.

(*Z*)-3-bromo-4-cyclohexylbut-3-en-2-ol (1m)

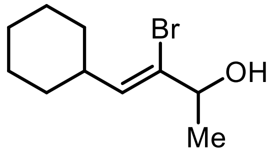

Prepared according to a modified literature procedure.[6] A solution of (*Z*)-3-bromo-4-cyclohexylbut-3-en-2-one (**S8**) (1.16 g, 5.00 mmol, 1.00 equiv.) and $CeCl_3·7H_2O$ (1.66 g, 5.25 mmol, 1.05 equiv.) in 43 mL of MeOH was stirred at rt until all solids were dissolved. Sodium borohydride (227 mg, 6 mmol, 1.2 equiv.) was then added slowly in small portions. After stirring 30 min at rt, the reaction was quenched with 2M HCl (20 mL). The organic layer was extracted with Et_2O (3 x 20 mL) and washed with brine (3 X 20 mL), then dried over $MgSO_4$. Solvent was removed under reduced pressure. Crude material was purified via column chromatography on SiO_2 using 10-20% EtOAc in hexanes to afford (**1m**) as a light-yellow oil (66%, 772 mg, 3.31 mmol).

^{1}H NMR (500 MHz, $CDCl_3$) δ 5.79 (d, J = 8.8 Hz, 1H), 4.26 (q, J = 6.3 Hz, 1H), 2.45 – 2.35 (m, 1H), 1.88 (d, J = 5.8 Hz, 1H), 1.75 – 1.64 (m, 5H), 1.35 (d, J = 6.3 Hz, 3H), 1.32 – 1.25 (m, 2H), 1.21 – 1.14 (m, 1H), 1.13 – 1.03 (m, 2H)

^{13}C NMR (125 MHz, $CDCl_3$) δ 134.3, 130.6, 72.7, 40.0, 31.8, 26.0, 25.7, 22.6

HRMS (ESI) m/z: $[M + Na]^+$ Calcd for $C_{10}H_{17}BrO$ 255.0353; Found 255.0350.

(*E*)-4-(4-methoxyphenyl)but-3-en-2-one (S9)

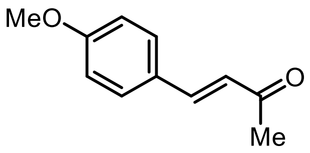

Prepared according to a modified literature procedure.[3] Sodium hydroxide (3.00 g, 75 mmol, 5.0 equiv.) added to a mixture of H_2O/EtOH (30 mL/45 mL) and stirred at rt until fully dissolved. Acetone (1.1 mL, 15 mmol, 1.0 equiv.) followed by 4-methoxybenzaldehyde (2.04 g, 15 mmol, 1.0 equiv.) were added to the reaction. This reaction mixture was then stirred and heated to 65 °C in an oil bath and monitored by TLC. After 18 h or when no starting materials remained, the reaction was cooled to rt, quenched with 1M HCl (20 mL). The organic layer was extracted with EtOAc (3 x 20 mL) and washed with brine (3 X 20 mL), then dried over $MgSO_4$. Solvent was removed under reduced pressure. Crude material was purified via column chromatography on SiO_2 using 10-20% EtOAc in hexanes to afford (**S9**) as a yellow oil (77%, 2.27 g, 12.85 mmol). Spectral data agree with that reported in the literature.[10]

^{1}H NMR (400 MHz, $CDCl_3$) δ 7.53 – 7.45 (m, 3H), 6.95 – 6.90 (m, 2H), 6.60 (d, J = 16.0 Hz, 1H), 3.85 (s, 3H), 2.36 (s, 3H)

(*Z*)-3-bromo-4-(4-methoxyphenyl)but-3-en-2-one (S10)

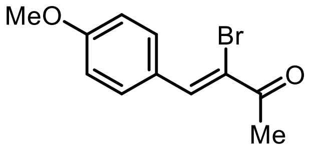

Prepared according to a modified literature procedure.[4] (*E*)-4-(4-methoxyphenyl)but-3-en-2-one (1.76 g, 10 mmol, 1.0 equiv.) was dissolved in 30 mL of DCM. This solution was cooled to 0 °C and stirred and Br_2 (615 µL, 12 mmol, 1.2 equiv.) was added slowly dropwise. After stirring 1h at rt, Et_3N (2.4 mL, 17 mmol, 1.7 equiv.) was added to the reaction mixture and 5 mL DCM was added. This mixture then stirred at rt overnight or until no dibrominated intermediate was detected by TLC. Once completed the reaction was quenched with 2M HCl and the organic phase was extracted with DCM and washed with sodium thiosulfate and brine. The combined organic phases were dried over $MgSO_4$. Solvent was removed under reduced to afford (**S10**) as a yellow oil (82%, 2.10 g, 8.23 mmol). Crude material was used without purification for the next step.

(*Z*)-3-bromo-4-(4-methoxyphenyl)but-3-en-2-ol (1n)

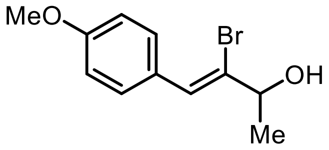

Prepared according to a modified literature procedure.[6] A solution of (*Z*)-3-bromo-4-(4-methoxyphenyl)but-3-en-2-one (**S10**) (1.28 g, 5.00 mmol, 1.00 equiv.) and $CeCl_3 \cdot 7H_2O$ (1.66 g, 5.25 mmol, 1.05 equiv.) in 43 mL of MeOH was stirred at rt until all solids were dissolved. Sodium borohydride (227 mg, 6 mmol, 1.2 equiv.) was then added slowly in small portions. After stirring 30 min at rt, the reaction was quenched with 2M HCl (20 mL). The organic layer was extracted with Et_2O (3 x 20 mL) and washed with brine (3 X 20 mL), then dried over $MgSO_4$. Solvent was removed under reduced pressure. Crude material was purified via column chromatography on SiO_2 using 10-20% EtOAc in hexanes to afford (**1n**) as a white solid (73%, 939 mg, 3.65 mmol).

^1H NMR (500 MHz, $CDCl_3$) δ 7.62 (d, J = 8.9 Hz, 2H), 7.01 (s, 1H), 6.91 (d, J = 8.9 Hz, 2H), 4.50 – 4.42 (m, 1H), 3.83 (s, 3H), 2.55 (d, J = 4.0 Hz, 1H) 1.48 (d, J = 6.3 Hz, 3H)

^{13}C NMR (125 MHz, $CDCl_3$) δ 159.4, 130.7, 129.5, 127.5, 126.5, 113.6, 73.8, 55.3, 22.6

HRMS (ESI) m/z: $[M + H]^+$ Calcd for $C_{11}H_{13}BrO_2$ 257.0177; Found 257.0175.

(*E*)-4-(4-(trifluoromethyl)phenyl)but-3-en-2-one (S11)

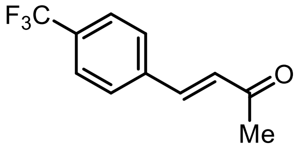

Prepared according to a modified literature procedure.[3] Sodium hydroxide (3.00 g, 75 mmol, 5.0 equiv.) added to a mixture of H_2O/EtOH (30 mL/45 mL) and stirred at rt until fully dissolved. Acetone (1.1 mL, 15 mmol, 1.0 equiv.) followed by 4-(trifluoromethyl)benzaldehyde (2.61 g, 15 mmol, 1.0 equiv.) were added to the reaction. This reaction mixture was then stirred and heated to 65 °C in an oil bath and monitored by TLC. After 18 h or when no starting materials remained, the reaction was cooled to rt, quenched with 1M HCl (20 mL). The organic layer was extracted with EtOAc (3 x 20 mL) and washed with brine (3 X 20 mL), then dried over $MgSO_4$. Solvent was removed under reduced pressure. Crude material was purified via column chromatography on SiO_2 using 10-20% EtOAc in hexanes to afford (**S11**) as a yellow solid (88%, 2.83 g, 13.20 mmol). Spectral data agree with that reported in the literature.[10]

^1H NMR (400 MHz, $CDCl_3$) δ 7.65 (s, 4H), 7.52 (d, J = 16.0 Hz, 1H), 6.78 (d, J = 16.0 Hz, 1H), 2.41 (s, 3H)

(*Z*)-3-bromo-4-(4-(trifluoromethyl)phenyl)but-3-en-2-one (S12)

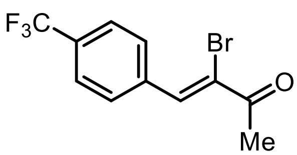

Prepared according to a modified literature procedure.[4] (*E*)-4-(4-(trifluoromethyl)phenyl)but-3-en-2-one (**S11**) (2.14 g, 10 mmol, 1.0 equiv.) was dissolved in 30 mL of DCM. This solution was cooled to 0 °C and stirred and Br_2 (615 µL, 12 mmol, 1.2 equiv.) was added slowly dropwise. After stirring 1h at rt, Et_3N (2.4 mL, 17 mmol, 1.7 equiv.) was added to the reaction mixture and 5 mL DCM was added. This mixture then stirred at rt overnight or until no dibrominated intermediate was detected by TLC. Once completed the reaction was quenched with 2M HCl and the organic phase was extracted with DCM and washed with sodium thiosulfate and brine. The combined organic phases were dried over $MgSO_4$. Solvent was removed under reduced to afford (**S12**) as a yellow oil (79%, 2.33 g, 7.94 mmol). Crude material was used without purification for the next step.

(*Z*)-3-bromo-4-(4-(trifluoromethyl)phenyl)but-3-en-2-ol (1o)

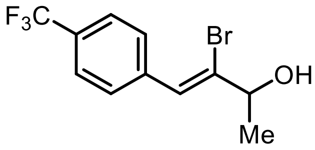

Prepared according to a modified literature procedure.[6] A solution of (*Z*)-3-bromo-4-(4-(trifluoromethyl)phenyl)but-3-en-2-one (**S12**) (1.47 g, 5.00 mmol, 1.00 equiv.) and $CeCl_3 \cdot 7H_2O$ (1.66 g, 5.25 mmol, 1.05 equiv.) in 43 mL of MeOH was stirred at rt until all solids were dissolved. Sodium borohydride (227 mg, 6 mmol, 1.2 equiv.) was then added slowly in small portions. After stirring 30 min at rt, the reaction was quenched with 2M HCl (20 mL). The organic layer was extracted with Et_2O (3 x 20 mL) and washed with brine (3 X 20 mL), then dried over $MgSO_4$. Solvent was removed under reduced pressure. Crude material was purified via column chromatography on SiO_2 using 10-20% EtOAc in hexanes to afford (**1o**) as a light-yellow oil (69%, 1.02 g, 3.45 mmol).

^{1}H NMR (500 MHz, $CDCl_3$) δ 7.68 (d, J = 8.5 Hz, 2H), 7.61 (d, J = 8.5 Hz, 2H), 7.12 (s, 1H), 4.52 (m, 1H), 2.38 (d, J = 5.5 Hz, 1H), 1.50 (d, J = 6.4 Hz, 3H)

^{13}C NMR (125 MHz, $CDCl_3$) δ 138.8, 133.8, 129.9 (q, J = 32.5 Hz), 129.4, 125.8, 125.22 (q, J = 3.8 Hz), 124.15 (q, J = 270.5 Hz), 73.6, 22.6

^{19}F NMR (470 MHz, $CDCl_3$) δ -62.5

HRMS (ESI) m/z: $[M + H]^+$ Calcd for $C_{11}H_{10}BrF_3O$ 294.9945; Found 294.9941.

(*E*)-4-(2,3-dihydrobenzofuran-5-yl)but-3-en-2-one (S13)

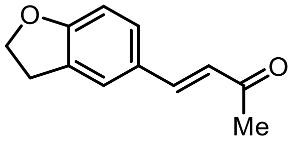

Prepared according to a modified literature procedure.[3] Sodium hydroxide (3.00 g, 75 mmol, 5.0 equiv.) added to a mixture of H_2O/EtOH (30 mL/45 mL) and stirred at rt until fully dissolved. Acetone (1.1 mL, 15 mmol, 1.0 equiv.) followed by 2,3-dihydrobenzofuran-5-carbaldehyde (2.22 g, 15 mmol, 1.0 equiv.) were added to the reaction. This reaction mixture was then stirred and heated to 65 °C in an oil bath and monitored by TLC. After 18 h or when no starting materials remained, the reaction was cooled to rt, quenched with 1M HCl (20 mL). The organic layer was extracted with EtOAc (3 x 20 mL) and washed with brine (3 X 20 mL), then dried over $MgSO_4$. Solvent was removed under reduced pressure. Crude material was purified via column chromatography on SiO_2 using 10-20% EtOAc in hexanes to afford (**S13**) as a white solid (68%, 1.91 g, 10.13 mmol). Spectral data agree with that reported in the literature.[11]

^1H NMR (500 MHz, $CDCl_3$) δ 7.47 (d, J = 16.0 Hz, 1H), 7.43 (s, 1H), 7.32 (d, J = 8.0 Hz, 1H), 6.79 (d, J = 8.0 Hz, 1H), 6.58 (d, J = 16.0 Hz, 1H), 4.63 (t, J = 8.5 Hz, 2H), 3.24 (t, J = 8.5 Hz, 2H), 2.35 (s, 3H)

(*Z*)-3-bromo-4-(2,3-dihydrobenzofuran-5-yl)but-3-en-2-one (S14)

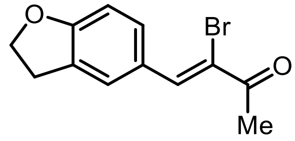

Prepared according to a modified literature procedure.[4] (*E*)-4-(2,3-dihydrobenzofuran-5-yl)but-3-en-2-one (**S13**) (1.88 g, 10 mmol, 1.0 equiv.) was dissolved in 30 mL of DCM. This solution was cooled to 0 °C and stirred and Br_2 (615 µL, 12 mmol, 1.2 equiv.) was added slowly dropwise. After stirring 1h at rt, Et_3N (2.4 mL, 17 mmol, 1.7 equiv.) was added to the reaction mixture and 5 mL DCM was added. This mixture then stirred at rt overnight or until no dibrominated intermediate was detected by TLC. Once completed the reaction was quenched with 2M HCl and the organic phase was extracted with DCM and washed with sodium thiosulfate and brine. The combined organic phases were dried over $MgSO_4$. Solvent was removed under reduced to afford (**S14**) as a yellow solid (46%, 1.23 g, 4.61 mmol). Crude material was used without purification for the next step.

(*Z*)-3-bromo-4-(2,3-dihydrobenzofuran-5-yl)but-3-en-2-ol (1p)

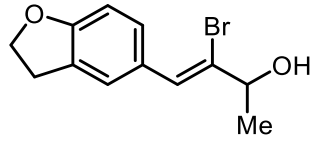

Prepared according to a modified literature procedure.[6] A solution of (*Z*)-3-bromo-4-(2,3-dihydrobenzofuran-5-yl)but-3-en-2-one (**S14**) (1.34 g, 5.00 mmol, 1.00 equiv.) and $CeCl_3 \cdot 7H_2O$ (1.66 g, 5.25 mmol, 1.05 equiv.) in 43 mL of MeOH was stirred at rt until all solids were dissolved. Sodium borohydride (227 mg, 6 mmol, 1.2 equiv.) was then added slowly in small portions. After stirring 30 min at rt, the reaction was quenched with 2M HCl (20 mL). The organic layer was extracted with Et_2O (3 x 20 mL) and washed with brine (3 X 20 mL), then dried over $MgSO_4$. Solvent was removed under reduced pressure. Crude material was purified via column chromatography on SiO_2 using 10-20% EtOAc in hexanes to afford (**1p**) as a yellow oil (83%, 1.11 g, 4.14 mmol).

^1H NMR (500 MHz, $CDCl_3$) δ 7.59 (s, 1H), 7.36 (d, J = 8.3 Hz, 1H), 6.98 (s, 1H), 6.77 (d, J = 8.3 Hz, 1H), 4.60 (t, J = 8.7 Hz, 2H), 4.46 (m, 1H), 3.23 (t, J = 8.7 Hz, 2H), 2.04 (d, J = 5.8 Hz, 1H), 1.46 (d, J = 6.3 Hz, 3H)

^{13}C NMR (125 MHz, $CDCl_3$) δ 160.2, 130.0, 129.0, 127.4, 127.1, 127.0, 125.8, 109.1, 73.9, 71.7, 29.7, 22.7

HRMS (ESI) m/z: $[M + H]^+$ Calcd for $C_{12}H_{13}BrO_2$ 269.0177; Found 269.0162.

Preparation of 2-bromocyclohex-2-en-1-ol (1k)

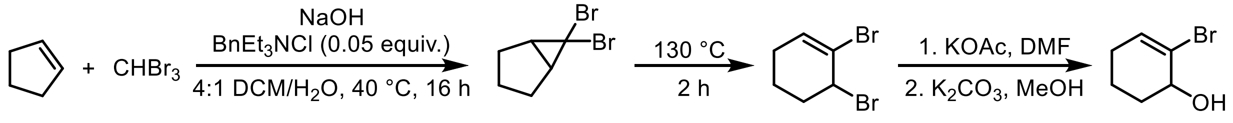

S15 S16 1k

6,6-dibromobicyclo[3.1.0]hexane (S15)

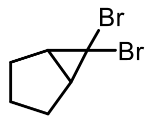

Prepared according to modified literature procedure.[12] Cyclopentene (1.57 g, 23 mmol, 1.15 equiv.) and 14 mL of DCM were combined and stirred at rt. NaOH (480 mg, 12 mmol, 0.6 equiv.) and 3 mL of water were next added at 0 °C and stirred until completely dissolved and the solution was brought back up to rt. Benzyltriethylammonium chloride (228 mg, 1 mmol, 0.05 equiv.) was then added followed by

bromoform (5.05 g, 20 mmol, 1.0 equiv.) to the reaction mixture. The solution was then heated to 40 °C in an oil bath and allowed to stir overnight. Once complete, the reaction was removed from the heat and quenched with 20 mL 1M HCl. The organic layer was extracted with DCM (3 x 20 mL) and washed with brine (3 X 20 mL), then dried over $MgSO_4$. Solvent was removed under reduced pressure. Crude material was purified via column chromatography on SiO_2 using 10-20% EtOAc in hexanes to afford (**S15**) as a brown oil (74%, 3.54 g, 14.76 mmol). Spectral data agree with that reported in the literature.[12]

^{1}H NMR (400 MHz, $CDCl_3$) δ 2.25 – 2.21 (m, 2H), 2.09 – 1.99 (m, 2H), 1.92 – 1.84 (m, 2H), 1.77 – 1.68 (m, 2H)

1,6-dibromocyclohex-1-ene (S16)

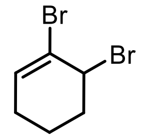

Prepared according to modified literature procedure.[12] 6,6-dibromobicyclo[3.1.0]hexane (**S15**) (2.40 g, 10 mmol, 1.0 equiv.) was added neat or with a small about of hexanes to transfer material to a round bottom flask. A stir bar was added, and the material was heated to 130 °C in an oil bath and stirred for 2 h. After the conversion was complete, the reaction was cooled to rt. Crude material was purified via column chromatography on SiO_2 using 10-20% EtOAc in hexanes to afford (**S16**) as a brown oil (91%, 2.18 g, 9.08 mmol). Spectral data agree with that reported in the literature.[12]

^{1}H NMR (400 MHz, $CDCl_3$) δ 6.21 – 6.17 (m, 1H), 4.80 – 4.76 (m, 1H), 2.36 – 2.20 (m, 3H), 2.20 – 2.09 (m, 1H), 2.07 – 1.93 (m, 1H), 1.80 – 1.70 (m, 1H)

2-bromocyclohex-2-en-1-ol (1k)

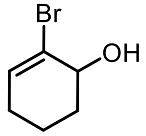

Prepared according to modified literature procedure.[13] 1,6-dibromocyclohex-1-ene (**S16**) (1.20 g, 5 mmol, 1.0 equiv.) was dissolved in 15 mL of DMF. Potassium acetate (1.47 g, 15 mmol, 3 equiv.) was added to the reaction and it was stirred at rt overnight. Once complete, the organic layer was extracted with EtOAc (3 x 20 mL) and washed with brine (3 X 20 mL), then dried over $MgSO_4$. Solvent was removed under reduced pressure. Crude material was redissolved in 50 mL methanol and potassium carbonate (3.46 g, 25 mmol, 5 equiv.) was added to the solution. This solution was stirred at rt for 3 h or until fully converted. Excess base was filtered out of the reaction and solvent was removed under reduced pressure. Crude material was purified via column chromatography on SiO_2 using 10-20% EtOAc in hexanes to afford (**1k**) as a light-brown oil (82%, 729 mg, 4.12 mmol). Spectral data agree with that reported in the literature.[14]

^{1}H NMR (500 MHz, $CDCl_3$) δ 6.19 (t, J = 4.3 Hz, 1H), 4.20 (t, J = 4.99 Hz, 1H), 2.31 (s, 1H), 2.17 – 2.09 (m, 1H), 2.07 – 1.99 (m, 1H), 1.97 – 1.85 (m, 2H), 1.77 – 1.69 (m, 1H), 1.66 – 1.59 (m, 1H)

Ambiphile Synthesis Procedure C:

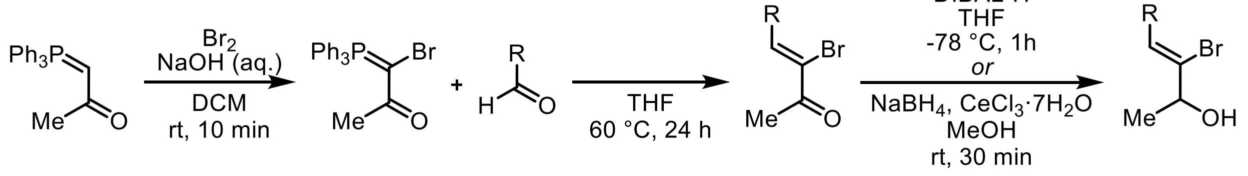

Wittig Reagent Bromination

Prepared according to a modified literature procedure.[15] 1-(Triphenylphosphoranylidene)-2-propanone (4.78 g, 15 mmol, 1.0 equiv.) was added to 100 mL of DCM and was stirred at rt until dissolved. To this solution, bromine (780 μL, 15 mmol, 1.0 equiv.) was added dropwise followed

by an aqueous solution of NaOH (0.25M, 66 mL, 1.1 equiv.). This biphasic mixture was stirred vigorously at rt for 10 min. The phases were separated and the aqueous phase extracted with DCM. The combined organic phases were dried over $MgSO_4$ and concentrated under reduced pressure to give a tan solid (99%, 5.90 g, 14.84 mmol) Crude material was used without purification for the next step.

Bromoenone Synthesis
Prepared according to a modified literature procedure.[16] 1-Bromo-1-(triphenylphosphaneylidene)-2-propanone (5.56 g, 14 mmol, 1.4 equiv.) was dissolved in dry THF (20mL) under nitrogen. This solution was stirred at rt and aldehyde (10 mmol, 1.0 equiv.) was added dropwise. The reaction was heated to 60 °C in an oil bath and left to stir overnight. Once no aldehyde remained via TLC, the reaction was cooled to rt and diluted with hexanes (40 mL). This mixture was than filtered through silica with EtOAc and concentrated under reduced pressure to give bromoenone product.

Reduction to Alcohol – DIBAL-H
Prepared according to a modified literature procedure.[5] To a solution of brominated ketone (5 mmol, 1.0 equiv.) in 15 mL dry THF, a solution of DIBAL-H reagent in toluene (10 mmol, 2 equiv.) at -78 °C was added slowly dropwise under a nitrogen balloon. After stirring 1 hour at -78 °C or once no enone remained via TLC, saturated NH_4Cl was added, and the organic phase was extracted with Et_2O and washed with brine. The combined organic phases were dried over $MgSO_4$. Solvent was removed under reduced pressure and purified by column chromatography on silica gel (10-20% EtOAc in hexanes) to provide product.

Reduction to Alcohol – $NaBH_4$
Prepared according to a modified literature procedure.[6] A solution of brominated ketone (5 mmol, 1.0 equiv.) and $CeCl_3{\cdot}7H_2O$ (1.66 g, 5.25 mmol, 1.05 equiv.) in 43 mL of MeOH was stirred at rt until all solids were dissolved. Sodium borohydride (227 mg, 6 mmol, 1.2 equiv.) was then added slowly in small portions. After stirring 30 min at rt, the reaction was quenched with 2M HCl. The organic layer was extracted with Et_2O and washed with brine then dried over $MgSO_4$. Solvent was removed under reduced pressure and purified by column chromatography on silica gel (10-20% EtOAc in hexanes) to provide product.

(*Z*)-3-bromo-6-phenylhex-3-en-2-one (S17)

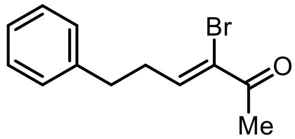

Prepared according to a modified literature procedure.[16] 1-Bromo-1-(triphenylphosphaneylidene)-2-propanone (5.56 g, 14 mmol, 1.4 equiv.) was dissolved in dry THF (20mL) under nitrogen. This solution was stirred at rt and 3-phenylpropanal (1.34 g, 10 mmol, 1.0 equiv.) was added dropwise. The reaction was heated to 60 °C in an oil bath and left to stir overnight. Once no aldehyde remained via TLC, the reaction was cooled to rt and diluted with hexanes (40 mL). This mixture was then filtered through silica with EtOAc (40 mL) and concentrated under reduced pressure to afford (**S17**) as a yellow oil (63%, 1.59 g, 6.29 mmol). Crude material was used without purification for the next step.

(*Z*)-3-bromo-6-phenylhex-3-en-2-ol (1l)

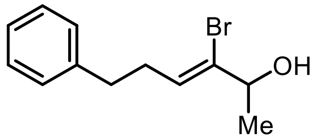

Prepared according to a modified literature procedure.[6] A solution of (*Z*)-3-bromo-6-phenylhex-3-en-2-one (**S17**) (1.27 g, 5.00 mmol, 1.00 equiv.) and $CeCl_3{\cdot}7H_2O$ (1.66 g, 5.25 mmol, 1.05 equiv.) in 43 mL of MeOH was stirred at rt until all solids were dissolved. Sodium borohydride (227 mg, 6 mmol, 1.2 equiv.) was then added slowly in small portions. After stirring 30 min at rt, the reaction was quenched with 2M HCl (20 mL). The organic layer was extracted with Et_2O (3 x 20 mL) and washed with brine (3 X 20 mL), then dried

over $MgSO_4$. Solvent was removed under reduced pressure. Crude material was purified via column chromatography on SiO_2 using 10-20% EtOAc in hexanes to afford (**1l**) as a yellow oil (84%, 1.07 g, 4.19 mmol).

^{1}H NMR (500 MHz, $CDCl_3$) δ 7.20 (t, J = 7.20 Hz, 2H), 7.24 – 7.18 (m, 3H), 6.02 (t, J = 6.89 Hz, 1H), 4.30 (q, 6.14 Hz, 1H), 2.73 (t, 7.73 Hz, 2H), 2.52 (q, J = 7.14 Hz, 2H), 1.89 (d, J = 5.69 Hz, 1H), 1.36 (d, J = 6.29 Hz, 3H)

^{13}C NMR (125 MHz, $CDCl_3$) δ 141.2, 133.4, 128.6 (2C), 128.0, 126.2, 72.6, 34.4, 32.5, 22.5

HRMS (ESI) m/z: $[M + H]^+$ Calcd for $C_{12}H_{15}BrO$ 255.0228; Found 255.0225.

(*Z*)-3-bromo-4-(pyridin-3-yl)but-3-en-2-one (S18)

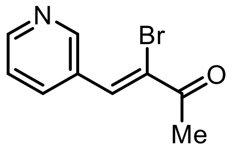

Prepared according to a modified literature procedure.[16] 1-Bromo-1-(triphenylphosphaneylidene)-2-propanone (5.56 g, 14 mmol, 1.4 equiv.) was dissolved in dry THF (20mL) under nitrogen. This solution was stirred at rt and nicotinaldehyde (1.07 g, 10 mmol, 1.0 equiv.) was added dropwise. The reaction was heated to 60 °C in an oil bath and left to stir overnight. Once no aldehyde remained via TLC, the reaction was cooled to rt and diluted with hexanes (40 mL). This mixture was then filtered through silica with EtOAc and concentrated under reduced pressure to afford (**S18**) as a yellow oil (53%, 1.21 g, 5.33 mmol). Crude material was used without purification for the next step.

(*Z*)-3-bromo-4-(pyridin-3-yl)but-3-en-2-ol (1q)

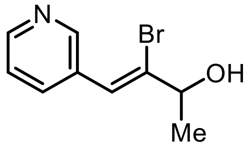

Prepared according to a modified literature procedure.[5] To a solution of (*Z*)-3-bromo-4-(pyridin-3-yl)but-3-en-2-one (**S18**) (1.13 g, 5 mmol, 1.0 equiv.) in 15 mL dry THF, a solution of DIBAL-H reagent in toluene (10 mmol, 2 equiv.) at -78 °C was added slowly dropwise under a nitrogen balloon. After stirring 1 hour at -78 °C or once no enone remained via TLC, saturated NH_4Cl (20mL) was added to quench the reaction. The organic layer was extracted with Et_2O (3 x 20 mL) and washed with brine (3 X 20 mL), then dried over $MgSO_4$. Solvent was removed under reduced pressure. Crude material was purified via column chromatography on SiO_2 using 10-20% EtOAc in hexanes to afford (**1q**) as a white solid (58%, 657 mg, 2.88 mmol).

^{1}H NMR (500 MHz, $CDCl_3$) δ 8.61 (d, J = 2.45 Hz, 1H), 8.45 (dd, J = 4.85, 1.55 Hz, 1H), 8.02 (dt, J = 8.00, 2.00 Hz, 1H), 7.28 (dd, J = 7.90, 4.85 Hz, 1H), 4.61 (s, 1H), 4.53- 4.48 (m, 1H), 1.47 (d, J = 6.30, 3H)

^{13}C NMR (125 MHz, $CDCl_3$) δ 150.0, 148.4, 136.3, 135.4, 131.8, 123.3, 122.9, 73.2, 22.5

HRMS (ESI) m/z: $[M + H]^+$ Calcd for $C_9H_{10}BrNO$ 228.0024; Found 228.0016.

Preparation of 1,3-Dienes

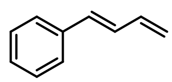
2a

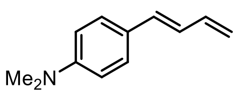

2b

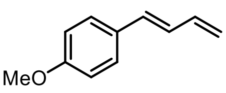

2c

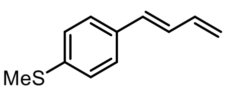

2d

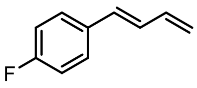

2e

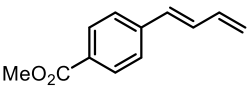

2f

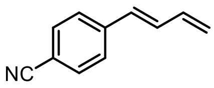

2g

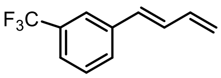

2h

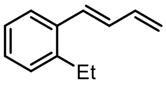

2i

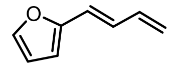

2j

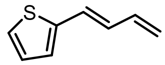

2k

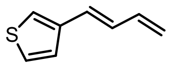

2l

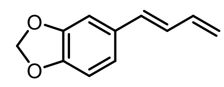

2m

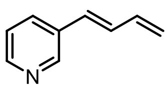

2n

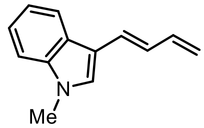

2o

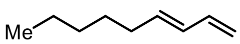

2p

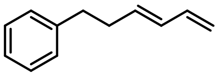
2q

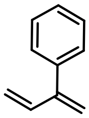
2r

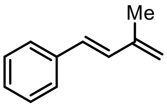

2s

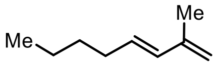

2t

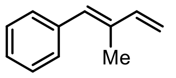

2u

Diene Synthesis Procedure A:

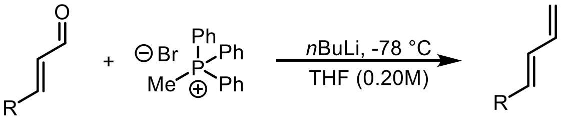

According to a modified literature procedure.[17] Methyltriphenylphosphonium bromide (1.00 equiv.) oven-dried round bottom flask equipped with a stir bar and septum. The flask was evacuated and refilled with N_2 three times before the addition of dry THF (0.2 M). The solution was then cooled to −78 °C. 2.5 M *n*BuLi in hexanes was then added (1.1 equiv.). The solution was allowed to warm to rt (20 °C) over 1 h. Aldehyde (1.1 equiv.) was then added to the solution and the reaction was

allowed to stir for 12 h. Upon completion, the solution was quenched with NH_4Cl (3 x 40 mL). The organic layer was extracted with Et_2O (100 mL) and washed with brine (3 X 40 mL), then dried over $MgSO_4$. Solvent was removed under reduced pressure. Crude material was purified via column chromatography on SiO_2.

(*E*)-buta-1,3-dien-1-ylbenzene (2a)

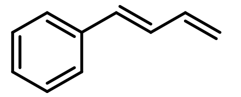

Prepared according to diene synthesis procedure A. Methyltriphenylphosphonium bromide (1.61 g, 4.50 mmol, 1.00 equiv.) was measured into an oven-dried round bottom flask equipped with a stir bar and septum. The flask was evacuated and refilled with N_2 three times before the addition of dry THF (30 mL, 0.2 M). The solution was then cooled to −78 °C. 2.5 M *n*BuLi in hexanes was then added (1.98 mL, 4.95 mmol, 1.1 equiv.). The solution was allowed to warm to rt (20 °C) over 1 h. Cinnamaldehyde (0.654 g, 4.95 mmol, 1.1 equiv.) was then added to the solution and the reaction was allowed to stir for 12 h. Upon completion, the solution was quenched with NH_4Cl (3 x 40 mL). The organic layer was extracted with Et_2O (100 mL) and washed with brine (3 X 40 mL), then dried over $MgSO_4$. Solvent was removed under reduced pressure. Crude material was purified via column chromatography on SiO_2 using 100% hexanes to afford (**2a**) as a colorless oil (83%, 486 mg, 3.74 mmol). Material was stored at −20 °C and used promptly. Spectral data agree with that reported in the literature.[17]

^{1}H NMR (500 MHz, $CDCl_3$): δ 7.42 – 7.40 (m, 2H), 7.34 – 7.31 (m, 2H), 7.25 – 7.22 (m, 1H), 6.81 – 6.76 (dd, J = 11.0, 11.0 Hz, 1H), 6.58 (d, J = 15.5 Hz, 1H), 6.53 – 6.49 (m, 1H), 5.34 (d, J = 17.2 Hz, 1H), 5.18 (d, J = 10.4 Hz, 1H)

(*E*)-4-(buta-1,3-dien-1-yl)-N,N-dimethylaniline (2b)

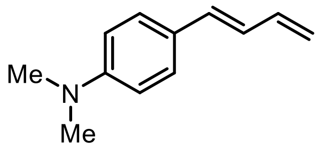

Prepared according to diene synthesis procedure A. Methyltriphenylphosphonium bromide (1.61 g, 4.50 mmol, 1.00 equiv.) was measured into an oven-dried round bottom flask equipped with a stir bar and septum. The flask was evacuated and refilled with N_2 three times before the addition of dry THF (30 mL, 0.2 M). The solution was then cooled to −78 °C. 2.5 M *n*BuLi in hexanes was then added (1.98 mL, 4.95 mmol, 1.1 equiv.). The solution was allowed to warm to rt (20 °C) over 1 h. 4-(dimethylamino)cinnamaldehyde (0.867 g, 4.95 mmol, 1.1 equiv.) was then added to the solution and the reaction was allowed to stir for 12 h. Upon completion, the solution was quenched with NH_4Cl (3 x 40 mL). The organic layer was extracted with Et_2O (100 mL) and washed with brine (3 X 40 mL), then dried over $MgSO_4$. Solvent was removed under reduced pressure. Crude material was purified via column chromatography on SiO_2 using 100% hexanes to afford (**2b**) as a colorless oil (78%, 608 mg, 3.51 mmol) Material was stored at −20 °C and used promptly. Spectral data agree with that reported in the literature.[18]

^{1}H NMR (400 MHz, $CDCl_3$) δ 7.30 (d, J = 8.9 Hz, 2H), 6.68 – 6.58 (m, 3H), 6.52 – 6.43 (m, 2H), 5.22 (dd, J = 17.5, 1.7 Hz, 1H), 5.03 (dd, J = 9.8, 1.6 Hz, 1H), 2.96 (s, 6H)

(*E*)-1-(buta-1,3-dien-1-yl)-4-methoxybenzene (2c)

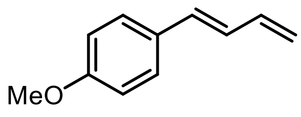

Prepared according to diene synthesis procedure A. Methyltriphenylphosphonium bromide (1.61 g, 4.50 mmol, 1.00 equiv.) was measured into an oven-dried round bottom flask equipped with a stir bar and septum. The flask was evacuated and refilled with N_2 three times before the addition of dry THF (30 mL, 0.2 M). The solution was then cooled to −78 °C. 2.5 M *n*BuLi in hexanes was then added (1.98 mL, 4.95 mmol, 1.1 equiv.). The solution was allowed to warm to rt (20 °C) over 1 h. *trans*-p-methoxycinnamaldehyde (0.802 g, 4.95 mmol, 1.1 equiv.) was then added to the solution and the reaction was allowed to stir for 12 h. Upon completion,

the solution was quenched with NH_4Cl (3 x 40 mL). The organic layer was extracted with Et_2O (100 mL) and washed with brine (3 X 40 mL), then dried over $MgSO_4$. Solvent was removed under reduced pressure. Crude material was purified via column chromatography on SiO_2 using 100% hexanes to afford (**2c**) as a white solid (94%, 678 mg, 4.23 mmol) Material was stored at −20 °C and used promptly. Spectral data agree with that reported in the literature.[17]

^{1}H NMR (500 MHz, $CDCl_3$): δ 7.34 (d, J = 8.6 Hz, 2H), 6.86 (d, J = 8.6 Hz, 2H), 6.67 (dd, J = 10.4, 15.2 Hz, 1H), 6.52 (d, J = 10.4 Hz, 1H), 6.50 – 6.44 (m, 1H), 5.28 (d, J = 16.0 Hz, 1H), 5.11 (d, J = 9.2 Hz, 1H), 3.81 (s, 3H)

(*E*)-1-(buta-1,3-dien-1-yl)-4-flourobenzene (2e)

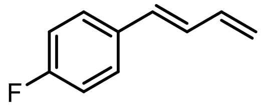

Prepared according to diene synthesis procedure A. Methyltriphenylphosphonium bromide (1.61 g, 4.50 mmol, 1.00 equiv.) was measured into an oven-dried round bottom flask equipped with a stir bar and septum. The flask was evacuated and refilled with N_2 three times before the addition of dry THF (30 mL, 0.2 M). The solution was then cooled to −78 °C. 2.5 M *n*BuLi in hexanes was then added (1.98 mL, 4.95 mmol, 1.1 equiv.). The solution was allowed to warm to rt (20 °C) over 1 h. *trans*-4-fluorocinnamaldehyde (0.802 g, 4.95 mmol, 1.1 equiv.) was then added to the solution and the reaction was allowed to stir for 12 h. Upon completion, the solution was quenched with NH_4Cl (3 x 40 mL). The organic layer was extracted with Et_2O (100 mL) and washed with brine (3 X 40 mL), then dried over $MgSO_4$. Solvent was removed under reduced pressure. Crude material was purified via column chromatography on SiO_2 using 100% hexanes to afford (**2e**) as a colorless oil (86%, 573 mg, 3.87 mmol). Material was stored at −20 °C and used promptly. Spectral data agree with that reported in the literature.[18]

^{1}H NMR (400 MHz, $CDCl_3$) δ 7.38 – 7.33 (m, 2H), 7.03 – 6.97 (m, 2H), 6.69 (dd, J = 15.6, 10.5 Hz, 1H), 6.54 – 6.44 (m, 2H), 5.32 (d, J = 15.6 Hz, 1H), 5.17 (d, J = 10.5 Hz, 1H)

(*E*)-2-(buta-1,3-dien-1-yl)furan (2j)

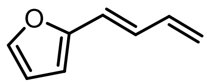

Prepared according to diene synthesis procedure A. Methyltriphenylphosphonium bromide (1.61 g, 4.50 mmol, 1.00 equiv.) was measured into an oven-dried round bottom flask equipped with a stir bar and septum. The flask was evacuated and refilled with N_2 three times before the addition of dry THF (30 mL, 0.2 M). The solution was then cooled to −78 °C. 2.5 M *n*BuLi in hexanes was then added (1.98 mL, 4.95 mmol, 1.1 equiv.). The solution was allowed to warm to rt (20 °C) over 1 h. (*E*)-3-(Furan-2-yl)acrylaldehyde (0.604 g, 4.95 mmol, 1.1 equiv.) was then added to the solution and the reaction was allowed to stir for 12 h. Upon completion, the solution was quenched with NH_4Cl (3 x 40 mL). The organic layer was extracted with Et_2O (100 mL) and washed with brine (3 X 40 mL), then dried over $MgSO_4$. Solvent was removed under reduced pressure. Crude material was purified via column chromatography on SiO_2 using 100% hexanes to afford (**2j**) as a colorless oil (68%, 368 mg, 3.06 mmol). Material was stored at −20 °C and used promptly. Spectral data agree with that reported in the literature.[18]

^{1}H NMR (400 MHz, $CDCl_3$) δ 7.36 (d, J = 1.6 Hz, 1H), 6.70 (dd, J = 12.5, 8.7 Hz, 1H), 6.43 (dt, J = 13.5, 8.5 Hz, 1H), 6.39 – 6.33 (m, 2H), 6.26 (d, J = 2.8 Hz, 1H), 5.32 (d, J = 13.5 Hz, 1H), 5.15 (d, J = 8.0 Hz, 1H)

(*E*)-1,3-nonadiene (2p)

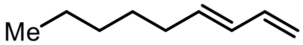

Prepared according to diene synthesis procedure A. Methyltriphenylphosphonium bromide (1.61 g, 4.50 mmol, 1.00 equiv.) was measured into an oven-dried round bottom flask equipped with a stir bar and septum. The

flask was evacuated and refilled with N_2 three times before the addition of dry THF (30 mL, 0.2 M). The solution was then cooled to −78 °C. 2.5 M *n*BuLi in hexanes was then added (1.98 mL, 4.95 mmol, 1.1 equiv.). The solution was allowed to warm to rt (20 °C) over 1 h. Trans 2-octenal (0.568 g, 4.95 mmol, 1.1 equiv.) was then added to the solution and the reaction was allowed to stir for 12 h. Upon completion, the solution was quenched with NH_4Cl (3 x 40 mL). The organic layer was extracted with Et_2O (100 mL) and washed with brine (3 X 40 mL), then dried over $MgSO_4$. Solvent was removed under reduced pressure. Crude material was purified via column chromatography on SiO_2 using 100% hexanes to afford (**2p**) as a colorless oil (63%, 352 mg, 2.84 mmol). Material was stored at −20 °C and used promptly. Spectral data agree with that reported in the literature.[19]

^{1}H NMR (400 MHz, $CDCl_3$) δ 6.30 (dt, J = 16.9, 10.2 Hz, 1H), 6.07 – 6.00 (m, 1H), 5.70 (dt, J = 15.1, 7.7 Hz, 1H), 5.10 – 5.05 (m, 1H), 4.95 – 4.92 (m, 1H), 2.06 (q, J = 8.7 Hz, 2H), 1.41 – 1.22 (m, 6H), 0.88 (t, J = 7.0 Hz, 3H)

(*E*)-(3-methylbuta-1,3-dien-1-yl)benzene (2s)

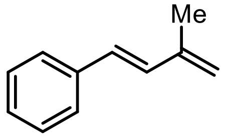

Prepared according to diene synthesis procedure A. Methyltriphenylphosphonium bromide (1.61 g, 4.50 mmol, 1.00 equiv.) was measured into an oven-dried round bottom flask equipped with a stir bar and septum. The flask was evacuated and refilled with N_2 three times before the addition of dry THF (30 mL, 0.2 M). The solution was then cooled to −78 °C. 2.5 M *n*BuLi in hexanes was then added (1.98 mL, 4.95 mmol, 1.1 equiv.). The solution was allowed to warm to rt (20 °C) over 1 h. (*E*)-4-phenylbut-3-en-2-one (0.724 g, 4.95 mmol, 1.1 equiv.) was then added to the solution and the reaction was allowed to stir for 12 h. Upon completion, the solution was quenched with NH_4Cl (3 x 40 mL). The organic layer was extracted with Et_2O (100 mL) and washed with brine (3 X 40 mL), then dried over $MgSO_4$. Solvent was removed under reduced pressure. Crude material was purified via column chromatography on SiO_2 using 100% hexanes to afford (**2s**) as a colorless oil (47%, 306 mg, 2.12 mmol). Material was stored at −20 °C and used promptly. Spectral data agree with that reported in the literature.[20]

^{1}H NMR (400 MHz, $CDCl_3$) δ 7.43 (dt, J = 7.1, 1.8 Hz, 2H), 7.32 (dt, J = 7.1, 1.4 Hz, 2H), 7.22 (tt, J = 7.4 Hz, 1.3 Hz, 1H), 6.88 (d, J = 16.2 Hz, 1H), 6.53 (d, J = 16.2 Hz, 1H), 5.11 (s, 1H), 5.07 (s, 1H), 1.97 (s, 3H)

(*E*)-2-methylocta-1,3-diene (2t)

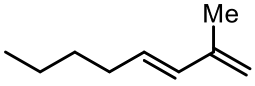

Prepared according to diene synthesis procedure A. Methyltriphenylphosphonium bromide (1.61 g, 4.50 mmol, 1.00 equiv.) was measured into an oven-dried round bottom flask equipped with a stir bar and septum. The flask was evacuated and refilled with N_2 three times before the addition of dry THF (30 mL, 0.2 M). The solution was then cooled to −78 °C. 2.5 M *n*BuLi in hexanes was then added (1.98 mL, 4.95 mmol, 1.1 equiv.). The solution was allowed to warm to rt (20 °C) over 1 h. (*E*)-oct-3-en-2-one (0.625 g, 4.95 mmol, 1.1 equiv.) was then added to the solution and the reaction was allowed to stir for 12 h. Upon completion, the solution was quenched with NH_4Cl (3 x 40 mL). The organic layer was extracted with Et_2O (100 mL) and washed with brine (3 X 40 mL), then dried over $MgSO_4$. Solvent was removed under reduced pressure. Crude material was purified via column chromatography on SiO_2 using 100% hexanes to afford (**2t**) as a colorless oil (33%, 188 mg, 1.51 mmol). Material was stored at −20 °C and used promptly. Spectral data agree with that reported in the literature.[17]

^{1}H NMR (500 MHz, $CDCl_3$): δ 6.14 (d, J = 15.6 Hz, 1H), 5.66 (dt, J = 15.5, 7.0 Hz, 1H), 4.86 (s, 2H), 2.11 (q, J = 6.8 Hz, 2H), 1.83 (s, 3H), 1.44 – 1.25 (m, 4H), 0.91 (t, J = 7.1 Hz, 3H)

(*E*)-(2-methylbuta-1,3-dien-1-yl)benzene (2u)

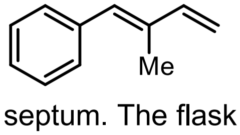

Prepared according to diene synthesis procedure A. Methyltriphenylphosphonium bromide (1.61 g, 4.50 mmol, 1.00 equiv.) was measured into an oven-dried round bottom flask equipped with a stir bar and septum. The flask was evacuated and refilled with N_2 three times before the addition of dry THF (30 mL, 0.2 M). The solution was then cooled to −78 °C. 2.5 M *n*BuLi in hexanes was then added (1.98 mL, 4.95 mmol, 1.1 equiv.). The solution was allowed to warm to rt (20 °C) over 1 h. (*E*)-2-methyl-3-phenylacrylaldehyde (0.802 g, 4.95 mmol, 1.1 equiv.) was then added to the solution and the reaction was allowed to stir for 12 h. Upon completion, the solution was quenched with NH_4Cl (3 x 40 mL). The organic layer was extracted with Et_2O (100 mL) and washed with brine (3 X 40 mL), then dried over $MgSO_4$. Solvent was removed under reduced pressure. Crude material was purified via column chromatography on SiO_2 using 100% hexanes to afford (**2u**) as a colorless oil (56%, 363 mg, 2.52 mmol) Material was stored at −20 °C and used promptly. Spectral data agree with that reported in the literature.[17]

^{1}H NMR (500 MHz, $CDCl_3$): δ 7.49 – 7.33 (m, 5H), 6.70 (m, 2H), 5.45 (d, J = 17.6 Hz, 1H), 5.28 (d, J = 10.4 Hz, 1H), 2.15 (s, 3H)

Diene Synthesis Procedure B:

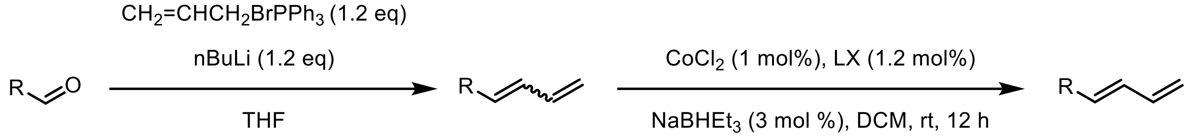

Diene Formation

Prepared according to a modified literature procedure.[17] To a flame dried flask equipped with a stir bar was added allyl triphenylphosphonium bromide (5.4 mmol, 2.07 g, 1.2 eq). Dry THF (50 mL) was then added to the flask. The reaction was then cooled to -78 °C. After cooling, *n*BuLi (1.2 eq, 2.16 mL, 1.2 eq) was added. The solution was then allowed to return to room temperature and stirred for 30 minutes. Carbonyl containing compounds (4.5 mmol, 1 eq) were then added slowly. The solution was then allowed to stir overnight. Afterwards, a saturated ammonium chloride solution (10 mL) was then added to quench the reaction. After an aqueous workup (NH_4Cl 10mL x 3), crude material was filtered through a silica plug to generate crude diene.

Diene Isomerization

Prepared according to a modified literature procedure.[21] To a 10 mL flame-dried Schlenk flask cooled under argon, $CoCl_2$ (0.025 mmol, 1 mol %), (**S20**) (0.03 mmol, 1.2 mol %), DCM (5 mL, 0.5 M), and a mixture of (*Z*)- and (*E*)-diene (2.5 mmol) were added in sequence. The mixture was stirred at room temperature for 5 min and added with $NaBHEt_3$ (75 µL, 0.075 mmol, 3 mol %) dropwise. After stirring at room temperature for 12 h, the resulting solution was purified by flash column chromatography to give the (*E*)-isomer as the product.

Preparation of S20

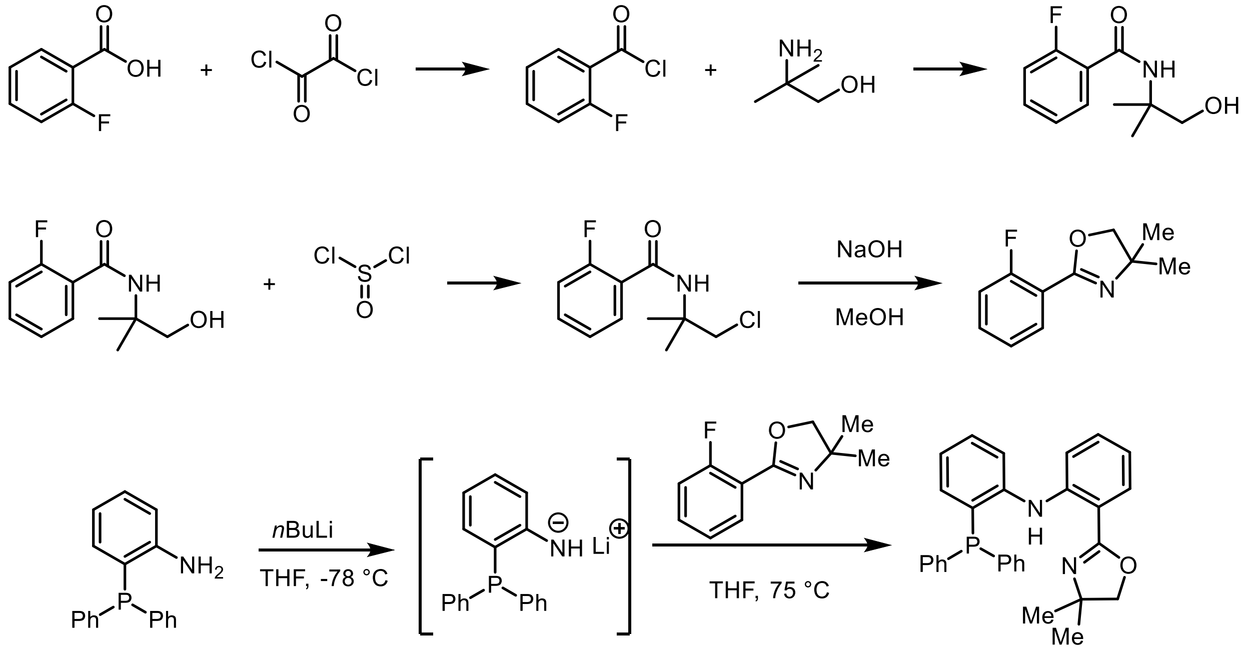

2-(2-fluorophenyl)-4,4-dimethyl-4,5-dihydrooxazole (S19)

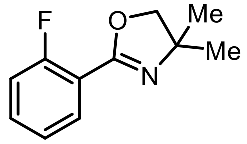

Prepared according to a modified literature procedure.[22] To a 100 mL oven-dried round-bottom flask was added the benzoic acid (10 mmol, 1.0 equiv.) and CH_2Cl_2 (20 mL, 0.5 M). The solution was cooled to 0 °C, oxalyl chloride (15 mmol, 1.5 equiv.) was then added, followed by slow addition of DMF (1.0 mmol, 0.1 equiv.). The reaction was slowly warmed to room temperature and stirred overnight. The reaction was diluted with CH_2Cl_2 (300 mL), the organic layer was washed by 5% $NaHCO_3$(200 mL x 2) and dried over anhydrous Na_2SO_4, filtered, and concentrated under reduced pressure. The crude 2-flourobenzoyl chloride was carried through to the next step without further purification. To a 100 mL oven-dried round-bottom flask was added commercial 2-amino-2-methyl-1-propanol (10 mmol, 1.0 eq), Et_3N (20 mmol, 2.0 equiv.) and 1,4 dioxane (14 mL). The solution was cooled to 0 °C, and the 2-flourobenzoyl chloride from the previous step in 12 mL of 1,4 dioxane was added slowly. The reaction was allowed to warm up to room temperature and stirred for another 1 hour. The reaction mixture was filtered through a silica plug with ethyl acetate and concentrated under reduced pressure. Crude 2-fluoro-N-(1-hydroxy-2-methylpropan-2-yl)benzamide was carried through to the next step without further purification. An oven-dried 300 mL round-bottom flask equipped with condenser was added the crude amide and toluene (70 mL). After cooled to 0 °C, thionyl chloride (2.2 mL, 30 mmol, 3.0 eq.), was added dropwise and the reaction was refluxed overnight in an oil bath. The crude solution was co-rotary evaporated with ethyl acetate The crude product was carried through without further purification. To the same round-bottom flask was added NaOH (15 mmol, 1.5 equiv.) and MeOH (80 mL), and the mixture was heated to reflux in an oil bath open to air for 1 hour. After cooling to room temperature, diethyl ether (200 mL) was added. The resulting solution was washed 3 times with brine. The organic layer was dried over anhydrous $MgSO_4$, filtered, and concentrated under reduced pressure. The crude mixture was purified via flash column chromatography (0% - 10% acetone in hexanes) to afford 2-(2-fluorophenyl)-4,4-dimethyl-4,5-dihydrooxazole (38%, 3.81 mmol, 736 mg).

2-(4,4-dimethyl-4,5-dihydrooxazol-2-yl)-N-(2-(diphenylphosphaneyl)phenyl)aniline (S20)

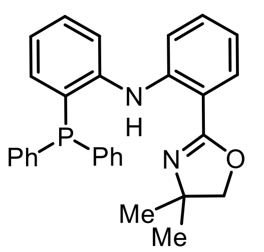

Prepared according to a modified literature procedure.[22] A solution of *n*BuLi (1.26 mL, 3.17 mmol, 2.5 M in hexane, 1.1 equiv.) was added dropwise over 10 min to a solution of 2-(diphenylphosphino)benzenamine (800 mg, 2.88 mmol, 1.0 equiv.) in 10 mL dry THF at −78 °C. After stirring for 4 h at room temperature, the lithium solution was transferred to the solution of 2-(2-fluorophenyl)-4,4-dimethyl-4,5-dihydrooxazole (613 mg, 3.17 mmol) in 10 mL of dry THF at −78 °C. The reaction mixture was slowly warmed to room temperature and then stirred for 48 h at 70 °C in an oil bath. The solvent was removed in vacuum and water (100mL) was added. The aqueous phases were extracted with EtOAc (4 × 100 mL), and the organic phases were dried over anhydrous Na_2SO_4. The residue was purified by passing through a silica gel column with hexane/EtOAc as eluent to give the desired (**S20**) as a yellow oil (45%,1.30 mmol, 584 mg).

(4-(buta-1,3-dien-1-yl)phenyl)(methyl)sulfane (S21)

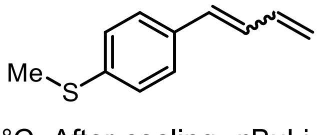

Prepared according to a modified literature procedure.[17] To a flame dried flask equipped with a stir bar was added allyl triphenylphosphonium bromide (5.4 mmol, 2.07 g, 1.2 eq). Dry THF (50 mL) was then added to the flask. The reaction was then cooled to -78 °C. After cooling, *n*BuLi (5.4 eq, 2.16 mL, 1.2 eq) was added. The solution was then allowed to return to room temperature and stirred for 30 minutes. 4-(Methylthio)benzaldehyde (685 mg, 4.5 mmol, 1 eq) were then added slowly. The solution was then allowed to stir overnight. Afterwards, a saturated ammonium chloride solution (10 mL) was then added to quench the reaction. After an aqueous workup (NH_4Cl 10mL x 3), crude material was filtered through a silica plug and solvent was removed to collect diene (3.92 mmol, 691 mg, 87%). Crude material was used without purification for the next step.

(*E*)-(4-(buta-1,3-dien-1-yl)phenyl)(methyl)sulfane (2d)

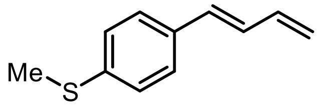

Prepared according to a modified literature procedure.[21] To a 10 mL flame-dried Schlenk flask cooled under argon, $CoCl_2$ (3.25mg, 0.025 mmol, 1 mol %), (**S20**) (11.2 mg, 0.03 mmol, 1.2 mol %), DCM (5 mL, 0.5 M), and a mixture of (**S21**) (*Z*)- and (*E*)-diene (441 mg, 2.5 mmol, 1 eq) were added in sequence. The mixture was stirred at room temperature for 5 min and added with $NaBHEt_3$ (75 µL, 0.075 mmol, 3 mol %) dropwise. After stirring at room temperature for 12 h, the resulting solution was purified by flash column chromatography to give the (E)-(4-(buta-1,3-dien-1-yl)phenyl)(methyl)sulfane as a colorless oil (96%, 426 mg, 2.41 mmol). Spectral data agree with that reported in the literature.[21]

^{1}H NMR (400 MHz, $CDCl_3$) δ 7.32 (d, J = 8.4 Hz, 2H), 7.20 (d, J = 8.5 Hz, 2H), 6.76 (dd, J = 15.1, 11.0 Hz, 1H), 6.55 – 6.45 (m, 2H), 5.33 (d, J = 17.1 Hz, 1H), 5.16 (d, J = 9.3 Hz, 1H)

methyl 4-(buta-1,3-dien-1-yl)benzoate (S22)

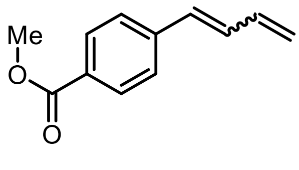

Prepared according to a modified literature procedure.[17] To a flame dried flask equipped with a stir bar was added allyl triphenylphosphonium bromide (5.4 mmol, 2.07 g, 1.2 eq). Dry THF (50 mL) was then added to the flask. The reaction was then cooled to -78 °C. After cooling, *n*BuLi (5.4 eq, 2.16 mL, 1.2 eq) was added. The solution was then allowed to return to room temperature and stirred for 30 minutes. Methyl 4-formylbenzoate (738 mg, 4.5 mmol, 1 eq) were then added slowly. The solution was then allowed to stir overnight. Afterwards, a saturated ammonium chloride solution (10 mL) was then added to quench the reaction. After an aqueous workup (NH_4Cl 10mL x 3), crude material was filtered through a silica plug and solvent

was removed to collect diene (3.81 mmol, 717 mg, 85%). Crude material was used without purification for the next step.

methyl-(E)-4-(buta-1,3-dien-1-yl)benzoate (2f)

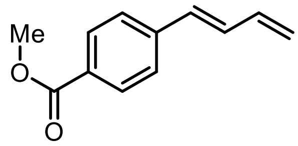

Prepared according to a modified literature procedure.[21] To a 10 mL flame-dried Schlenk flask cooled under argon, $CoCl_2$ (3.25mg, 0.025 mmol, 1 mol %), (**S20**) (11.2 mg, 0.03 mmol, 1.2 mol %), DCM (5 mL, 0.5 M), and a mixture of (**S22**) (*Z*)- and (*E*)-diene (471 mg, 2.5 mmol, 1 eq) were added in sequence. The mixture was stirred at room temperature for 5 min and added with $NaBHEt_3$ (75 µL, 0.075 mmol, 3 mol %) dropwise. After stirring at room temperature for 12 h, the resulting solution was purified by flash column chromatography to give the methyl-(*E*)-4-(buta-1,3-dien-1-yl)benzoate as a colorless oil (89%, 421 mg, 2.23 mmol, (90/10 *E/Z*). Spectral data agree with that reported in the literature.[21]

^{1}H NMR (400 MHz, $CDCl_3$) δ 7.99 (d, J = 8.5 Hz, 2H), 7.36 (d, J = 8.5 Hz, 2H), 6.88 – 6.79 (m, 1H), 6.45 (d, J = 11.7 Hz, 1H), 6.36 – 6.30 (m, 1H), 5.45 – 5.40 (m, 1H), 5.30 – 5.26 (m, 1H), 3.91 (s, 3H)

4-(buta-1,3-dien-1-yl)benzonitrile (S23)

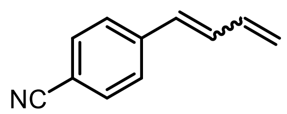

Prepared according to a modified literature procedure.[17] To a flame dried flask equipped with a stir bar was added allyl triphenylphosphonium bromide (5.4 mmol, 2.07 g, 1.2 eq). Dry THF (50 mL) was then added to the flask. The reaction was then cooled to -78 °C. After cooling, *n*BuLi (5.4 mmol, 2.16 mL, 1.2 eq) was added. The solution was then allowed to return to room temperature and stirred for 30 minutes. 4-formylbenzonitrile (590 mg, 4.5 mmol, 1 eq) were then added slowly. The solution was then allowed to stir overnight. Afterwards, a saturated ammonium chloride solution (10 mL) was then added to quench the reaction. After an aqueous workup (NH_4Cl 10mL x 3), crude material was filtered through a silica plug and solvent was removed to collect diene (2.63 mmol, 408 mg, 58%). Crude material was used without purification for the next step.

(*E*)-4-(buta-1,3-dien-1-yl)benzonitrile (2g)

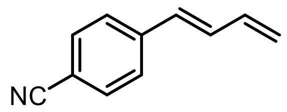

Prepared according to a modified literature procedure.[21] To a 10 mL flame-dried Schlenk flask cooled under argon, $CoCl_2$ (3.25mg, 0.025 mmol, 1 mol %), (**S20**) (11.2 mg, 0.03 mmol, 1.2 mol %), DCM (5 mL, 0.5 M), and a mixture of (**S23**) (*Z*)- and (*E*)-diene (388 mg, 2.5 mmol, 1 eq) were added in sequence. The mixture was stirred at room temperature for 5 min and added with $NaBHEt_3$ (75 µL, 0.075 mmol, 3 mol %) dropwise. After stirring at room temperature for 12 h, the resulting solution was purified by flash column chromatography to give the (*E*)-4-(buta-1,3-dien-1-yl)benzonitrile as a colorless oil (91%, 354 mg, 2.28 mmol, less than 5% *Z*-isomer). Spectral data agree with that reported in the literature.[23]

^{1}H NMR (400 MHz, $CDCl_3$) δ 7.57 (d, J = 8.4 Hz, 2H), 7.45 (d, J = 8.4 Hz, 2H), 6.86 (dd, J = 15.6, 10.7 Hz, 1H), 6.55 – 6.44 (m, 2H), 5.43 (d, J = 16.8 Hz, 1H), 5.30 (d, J = 10.4 Hz, 1H)

1-(buta-1,3-dien-1-yl)-3-(trifluoromethyl)benzene (S24)

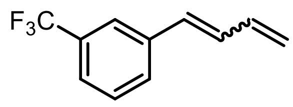

Prepared according to a modified literature procedure.[17] To a flame dried flask equipped with a stir bar was added allyl triphenylphosphonium bromide (5.4 mmol, 2.07 g, 1.2 eq). Dry THF (50 mL) was then added to the flask. The reaction was then cooled to -78 °C. After cooling, *n*BuLi (5.4 mmol, 2.16 mL, 1.2 eq) was added. The solution was then allowed to return to room temperature and stirred for 30 minutes. 3-(trifluoromethyl)benzaldehyde (4.5 mmol, 783 mg, 1 eq)

were then added slowly. The solution was then allowed to stir overnight. Afterwards, a saturated ammonium chloride solution (10 mL) was then added to quench the reaction. After an aqueous workup (NH_4Cl 10mL x 3), crude material was filtered through a silica plug and solvent was removed to collect diene (2.78 mmol, 550 mg, 62%). Crude material was used without purification for the next step.

(*E*)-1-(buta-1,3-dien-1-yl)-3-(trifluoromethyl)benzene (2h)

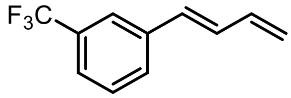

Prepared according to a modified literature procedure.[21] To a 10 mL flame-dried Schlenk flask cooled under argon, $CoCl_2$ (3.25mg, 0.025 mmol, 1 mol %), (**S20**) (11.2 mg, 0.03 mmol, 1.2 mol %), DCM (5 mL, 0.5 M), and a mixture of (**S24**) (*Z*)- and (*E*)-diene (495 mg, 2.5 mmol, 1 eq) were added in sequence. The mixture was stirred at room temperature for 5 min and added with $NaBHEt_3$ (75 µL, 0.075 mmol, 3 mol %) dropwise. After stirring at room temperature for 12 h, the resulting solution was purified by flash column chromatography to give the (*E*)-1-(buta-1,3-dien-1-yl)-3-(trifluoromethyl)benzene as a colorless solid (94%, 465 mg, 2.35 mmol). Spectral data agree with that reported in the literature.[23]

^{1}H NMR (400 MHz, $CDCl_3$) δ 7.63 (d, J = 1.4 Hz, 1H), 7.54 (d, J = 7.7 Hz, 1H), 7.47 – 7.38 (m, 2H), 6.83 (m, 1H), 6.59 – 6.45 (m, 2H), 5.42 – 5.37 (m, 1H), 5.26 – 5.23 (m, 1H)

1-(buta-1,3-dien-1-yl)-2-ethylbenzene (S25)

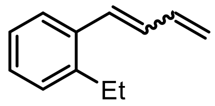

Prepared according to a modified literature procedure.[17] To a flame dried flask equipped with a stir bar was added allyl triphenylphosphonium bromide (5.4 mmol, 2.07 g, 1.2 eq). Dry THF (50 mL) was then added to the flask. The reaction was then cooled to -78 °C. After cooling, *n*BuLi (5.4 eq, 2.16 mL, 1.2 eq) was added. The solution was then allowed to return to room temperature and stirred for 30 minutes. *o*-ethylbenzaldehyde (604 mg, 4.5 mmol, 1 eq) were then added slowly. The solution was then allowed to stir overnight. Afterwards, a saturated ammonium chloride solution (10 mL) was then added to quench the reaction. After an aqueous workup (NH_4Cl 10mL x 3), crude material was filtered through a silica plug and solvent was removed to collect diene (3.69 mmol, 584 mg, 82%). Crude material was used without purification for the next step.

(*E*)-1-(buta-1,3-dien-1-yl)-2-ethylbenzene (2i)

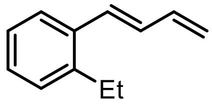

Prepared according to a modified literature procedure.[21] To a 10 mL flame-dried Schlenk flask cooled under argon, $CoCl_2$ (3.25mg, 0.025 mmol, 1 mol %), (**S20**) (11.2 mg, 0.03 mmol, 1.2 mol %), DCM (5 mL, 0.5 M), and a mixture of (**S25**) (*Z*)- and (*E*)-diene (396 mg, 2.5 mmol, 1 eq) were added in sequence. The mixture was stirred at room temperature for 5 min and added with $NaBHEt_3$ (75 µL, 0.075 mmol, 3 mol %) dropwise. After stirring at room temperature for 12 h, the resulting solution was purified by flash column chromatography to give (*E*)-1-(buta-1,3-dien-1-yl)-2-ethylbenzene as a colorless oil (97%, 383 mg, 2.43 mmol). Spectral data agree with that reported in the literature.[24]

^{1}H NMR (400 MHz, $CDCl_3$) δ 7.53 – 7.49 (m, 1H), 7.19 – 7.15 (m, 3H), 6.83 (d, J = 15.5 Hz, 1H), 6.70 (dd, J = 15.4, 10.2 Hz, 1H), 6.55 (dt, J = 16.8, 10.2 Hz, 1H), 5.35 – 5.31 (m, 1H), 5.18 – 5.15 (m, 1H), 2.71 (q, J = 7.6 Hz, 2H), 1.20 (t, J = 7.6 Hz, 3H).

2-(buta-1,3-dien-1-yl)thiophene (S26)

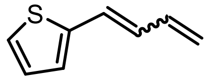

Prepared according to a modified literature procedure.[17] To a flame dried flask equipped with a stir bar was added allyl triphenylphosphonium bromide (5.4 mmol, 2.07 g, 1.2 eq). Dry THF (50 mL) was then added to the flask. The reaction was then cooled to -78 °C. After cooling, *n*BuLi (5.4 mmol, 2.16 mL,

1.2 eq) was added. The solution was then allowed to return to room temperature and stirred for 30 minutes. Thiophene-2-carbaldehyde (505 mg, 4.5 mmol, 1 eq) were then added slowly. The solution was then allowed to stir overnight. Afterwards, a saturated ammonium chloride solution (10 mL) was then added to quench the reaction. After an aqueous workup (NH_4Cl 10mL x 3), crude material was filtered through a silica plug and solvent was removed to collect diene (4.08 mmol, 556 mg, 91%). Crude material was used without purification for the next step.

(*E*)-2-(buta-1,3-dien-1-yl)thiophene (2k)

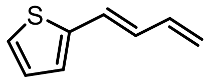

Prepared according to a modified literature procedure.[21] To a 10 mL flame-dried Schlenk flask cooled under argon, $CoCl_2$ (3.25mg, 0.025 mmol, 1 mol %), (**S20**) (11.2 mg, 0.03 mmol, 1.2 mol %), DCM (5 mL, 0.5 M), and a mixture of (**S26**) (*Z*)- and (*E*)-diene (341 mg, 2.5 mmol, 1 eq) were added in sequence. The mixture was stirred at room temperature for 5 min and added with $NaBHEt_3$ (75 µL, 0.075 mmol, 3 mol %) dropwise. After stirring at room temperature for 12 h, the resulting solution was purified by flash column chromatography to give (*E*)-2-(buta-1,3-dien-1-yl)thiophene as a colorless oil (93%, 318 mg, 2.33 mmol). Spectral data agree with that reported in the literature.[25]

^{1}H NMR (400 MHz, $CDCl_3$) δ 7.19 – 7.14 (m, 1H), 6.99 – 6.94 (m, 2H), 6.75 – 6.57 (m, 2H), 6.44 (d, J = 16.8 hz,1H), 5.31 (ddt, J = 16.9, 1.6, 0.7 Hz, 1H), 5.15 (ddt, J = 10.1, 1.5, 0.7 Hz, 1H),

3-(buta-1,3-dien-1-yl)thiophene (S27)

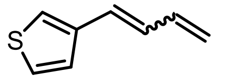

Prepared according to a modified literature procedure.[17] To a flame dried flask equipped with a stir bar was added allyl triphenylphosphonium bromide (5.4 mmol, 2.07 g, 1.2 eq). Dry THF (50 mL) was then added to the flask. The reaction was then cooled to -78 °C. After cooling, *n*BuLi (5.4 eq, 2.16 mL, 1.2 eq) was added. The solution was then allowed to return to room temperature and stirred for 30 minutes. thiophene-3-carbaldehyde (505 mg, 4.5 mmol, 1 eq) were then added slowly. The solution was then allowed to stir overnight. Afterwards, a saturated ammonium chloride solution (10 mL) was then added to quench the reaction. After an aqueous workup (NH_4Cl 10mL x 3), crude material was filtered through a silica plug and solvent was removed to collect diene (3.99 mmol, 543 mg, 89%). Crude material was used without purification for the next step.

(*E*)-3-(buta-1,3-dien-1-yl)thiophene (2l)

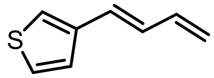

Prepared according to a modified literature procedure.[21] To a 10 mL flame-dried Schlenk flask cooled under argon, $CoCl_2$ (3.25mg, 0.025 mmol, 1 mol %), (**S20**) (11.2 mg, 0.03 mmol, 1.2 mol %), DCM (5 mL, 0.5 M), and a mixture of (**S27**) (*Z*)- and (*E*)-diene (341 mg, 2.5 mmol, 1 eq) were added in sequence. The mixture was stirred at room temperature for 5 min and added with $NaBHEt_3$ (75 µL, 0.075 mmol, 3 mol %) dropwise. After stirring at room temperature for 12 h, the resulting solution was purified by flash column chromatography to give (*E*)-3-(buta-1,3-dien-1-yl)thiophene as a colorless oil (97%, 331 mg, 2.43 mmol). Spectral data agree with that reported in the literature.[26]

^{1}H NMR (400 MHz, $CDCl_3$) δ 7.30 – 7.22 (m , 2H), 7.17 – 7.16 (m, 1H), 6.66 – 6.55 (m, 2H), 6.46 (dt, J = 16.9, 10.0 Hz, 1H), 5.30 (dd, J = 16.8, 1.6 Hz, 1H), 5.14 (dd, J = 10, 1.8 Hz, 1H)

5-(buta-1,3-dien-1-yl)benzo[d][1,3]dioxole (S28)

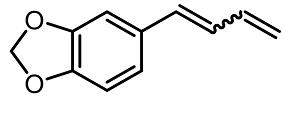

Prepared according to a modified literature procedure.[17] To a flame dried flask equipped with a stir bar was added allyl triphenylphosphonium bromide (5.4 mmol, 2.07 g, 1.2 eq). Dry THF (50 mL) was then added to the flask. The reaction was then cooled to -78 °C. After cooling, *n*BuLi (5.4 mmol, 2.16 mL, 1.2 eq) was added. The solution was then allowed to return to room temperature

and stirred for 30 minutes. benzo[d][1,3]dioxole-5-carbaldehyde (676 mg, 4.5 mmol, 1 eq) were then added slowly. The solution was then allowed to stir overnight. Afterwards, a saturated ammonium chloride solution (10 mL) was then added to quench the reaction. After an aqueous workup (NH_4Cl 10mL x 3), crude material was filtered through a silica plug and solvent was removed to collect diene (4.12 mmol, 717 mg, 92%). Crude material was used without purification for the next step.

(*E*)-5-(buta-1,3-dien-1-yl)benzo[d][1,3]dioxole (2m)

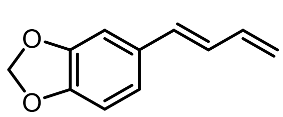

Prepared according to a modified literature procedure.[21] To a 10 mL flame-dried Schlenk flask cooled under argon, $CoCl_2$ (3.25mg, 0.025 mmol, 1 mol %), (**S20**) (11.2 mg, 0.03 mmol, 1.2 mol %), DCM (5 mL, 0.5 M), and a mixture of (**S28**) (*Z*)- and (*E*)-diene (435 mg, 2.5 mmol, 1 eq) were added in sequence. The mixture was stirred at room temperature for 5 min and added with $NaBHEt_3$ (75 μL, 0.075 mmol, 3 mol %) dropwise. After stirring at room temperature for 12 h, the resulting solution was purified by flash column chromatography to give the (*E*)-5-(buta-1,3-dien-1-yl)benzo[d][1,3]dioxole as a colorless oil (98%, 427 mg, 2.45 mmol, less than 5% *Z*-isomer). Spectral data agree with that reported in the literature.[21]

^{1}H NMR (400 MHz, $CDCl_3$) δ 6.95 (s, 1H), 6.83 (d, J = 8.1 Hz, 1H), 6.75 (d, J = 8.1 Hz, 1H), 6.63 (dd, J = 14.8, 9.6 Hz, 1H), 6.52 – 6.42 (m, 2H), 5.95 (s, 2H), 5.29 (d, J = 17.3 Hz, 1H), 5.13 (d, J = 9.9 Hz, 1H)

3-(buta-1,3-dien-1-yl)pyridine (S29)

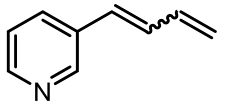

Prepared according to a modified literature procedure.[17] To a flame dried flask equipped with a stir bar was added allyl triphenylphosphonium bromide (5.4 mmol, 2.07 g, 1.2 eq). Dry THF (50 mL) was then added to the flask. The reaction was then cooled to -78 °C. After cooling, *n*BuLi (1.2 eq, 2.16 mL, 1.2 eq) was added. The solution was then allowed to return to room temperature and stirred for 30 minutes. Nicotinaldehyde (482 mg, 4.5 mmol, 1 eq) were then added slowly. The solution was then allowed to stir overnight. Afterwards, a saturated ammonium chloride solution (10 mL) was then added to quench the reaction. After an aqueous workup (NH_4Cl 10mL x 3), crude material was filtered through a silica plug and solvent was removed to collect diene (2.73 mmol, 358 mg, 61%). Crude material was used without purification for the next step.

(*E*)-3-(buta-1,3-dien-1-yl)pyridine (2n)

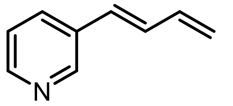

Prepared according to a modified literature procedure.[21] To a 10 mL flame-dried Schlenk flask cooled under argon, $CoCl_2$ (3.25mg, 0.025 mmol, 1 mol %), (**S20**) (11.2 mg, 0.03 mmol, 1.2 mol %), DCM (5 mL, 0.5 M), and 3-(buta-1,3-dien-1-yl)pyridine (**S29**) (327 mg, 2.5 mmol, 1 eq) were added in sequence. The mixture was stirred at room temperature for 5 min and added with $NaBHEt_3$ (75 μL, 0.075 mmol, 3 mol %) dropwise. After stirring at room temperature for 12 h, the resulting solution was purified by flash column chromatography to give the (*E*)-3-(buta-1,3-dien-1-yl)pyridine as a yellow oil (88%, 288 mg, 2.20 mmol). Spectral data agree with that reported in the literature.[27]

^{1}H NMR (400 MHz, $CDCl_3$) δ 8.61 (s, 1H), 8.45 (s, 1H), 7.70 (d, J = 8.0 Hz, 1H), 7.24 – 7.21 (m, 1H), 6.82 (dd, J = 15.3, 10.9 Hz, 1H), 6.55 – 6.45 (m, 2H), 5.38 (d, J = 17.1 Hz, 1H), 5.24 (d, J =9.1 Hz, 1H)

3-(buta-1,3-dien-1-yl)-1-methyl-1H-indole (S30)

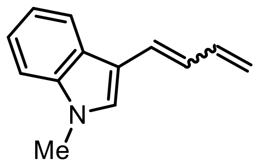

Prepared according to a modified literature procedure.[17] To a flame dried flask equipped with a stir bar was added allyl triphenylphosphonium bromide (5.4 mmol, 2.07 g, 1.2 eq). Dry THF (50 mL) was then added to the flask. The reaction was then cooled to -78 °C. After cooling, *n*BuLi (5.4 mmol, 2.16 mL, 1.2 eq) was added. The solution was then allowed to return to room temperature and stirred for 30 minutes. 1-methyl-1H-indole-3-carbaldehyde (716 mg, 4.5 mmol, 1 eq) were then added slowly. The solution was then allowed to stir overnight. Afterwards, a saturated ammonium chloride solution (10 mL) was then added to quench the reaction. After an aqueous workup (NH_4Cl 10mL x 3), crude material was filtered through a silica plug and solvent was removed to collect diene (3.24 mmol, 594 mg, 72%). Crude material was used without purification for the next step.

(*E*)-3-(buta-1,3-dien-1-yl)-1-methyl-1H-indole (2o)

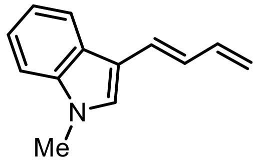

Prepared according to a modified literature procedure.[21] To a 10 mL flame-dried Schlenk flask cooled under argon, $CoCl_2$ (3.25mg, 0.025 mmol, 1 mol %), (**S20**) (11.2 mg, 0.03 mmol, 1.2 mol %), DCM (5 mL, 0.5 M), and a mixture of (**S30**) (*Z*)- and (*E*)-diene (458 mg, 2.5 mmol, 1 eq) were added in sequence. The mixture was stirred at room temperature for 5 min and added with $NaBHEt_3$ (75 μL, 0.075 mmol, 3 mol %) dropwise. After stirring at room temperature for 12 h, the resulting solution was purified by flash column chromatography to give the (*E*)-3-(buta-1,3-dien-1-yl)-1-methyl-1H-indole as a yellow oil (2.4 mmol, 448 mg, 98% yield). Spectral data agree with that reported in the literature.[17]

^{1}H NMR (400 MHz, $CDCl_3$) δ 7.67 (d, J = 7.9 Hz, 1H), 7.35 – 7.24 (m, 2H), 7.17 (dd, J = 15.6, 8.5 Hz, 2H), 6.98 (tt, J = 19.9, 10.1 Hz, 1H), 6.62 (d, J = 11.2 Hz, 1H), 6.23 (t, J = 11.2 Hz, 1H), 5.37 (d, J = 16.9 Hz, 1H), 5.18 (d, J = 10.1 Hz, 1H), 3.81 (s, 3H).

Preparation of (*E*)-hexa-3,5-dien-1-ylbenzene

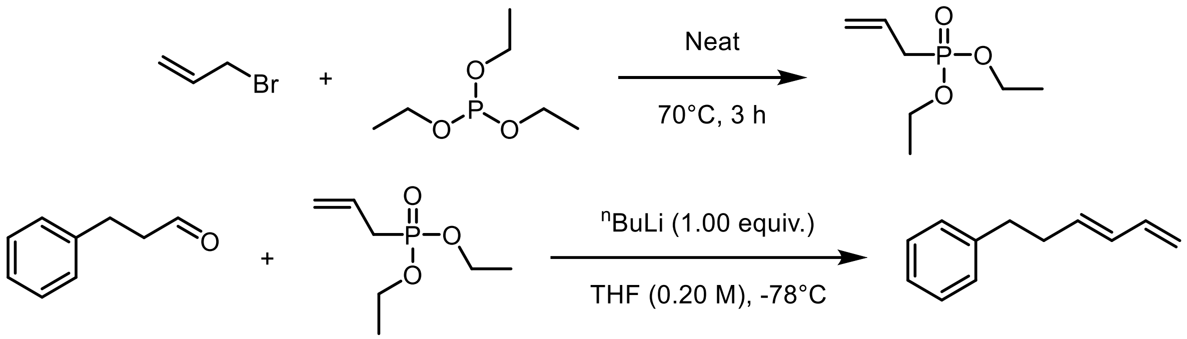

Preparation of diethyl allylphosphonate (S31)

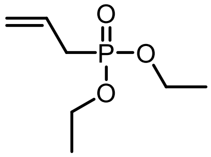

Prepared following a modified literature procedure.[28] To a round-bottom flask equipped with a reflux condenser and under a nitrogen atmosphere was added triethyl phosphite (3.42 mL, 20 mmol, 1 equiv.) and allyl bromide (1.90 mol, 22 mmol, 1.2 equiv.) was added. The mixture was heated to 70 °C in an oil bath for 3 h. After 3 h, the reaction was heated to 120 °C in an oil bath until allyl bromide was no longer detected (2 h). Diethyl allylphosphonate (**S31**) was recovered (96%, 3.42 g, 19.2 mmol) and used without further purification.

Preparation of (*E*)-hexa-3,5-dien-1-ylbenzene (2q)

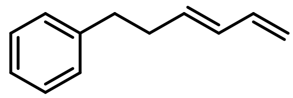

Prepared according to a modified literature procedure.[17] To a flame dried flask equipped with a stir bar was added diethyl allylphosphonate (**S31**) (20 mmol, 3.56 g, 2.0 eq). Dry THF (50 mL) was then added to the flask. The reaction was then cooled to -78 °C. After cooling, *n*BuLi (5.4 eq, 2.16 mL, 1.2 eq) was added. The solution was then allowed to return to room temperature and stirred for 30 minutes. 3-phenylpropanal (1.34 g, 10 mmol, 1 eq) were then added slowly. The solution was then allowed to stir overnight. Afterwards, a saturated ammonium chloride solution (10 mL) was then added to quench the reaction. After an aqueous workup (NH_4Cl 10mL x 3), crude material was filtered through a silica plug and solvent was removed to collect crude diene. The crude diene was purified by flash column chromatography to give (*E*)-hexa-3,5-dien-1-ylbenzene as a colorless oil (35%, 550 mg, 3.48 mmol). Spectra agrees with reported literature values.[29]

^{1}H NMR (400 MHz, $CDCl_3$) δ 7.35 – 7.13 (m, 5H), 6.68 – 6.23 (m, 1H), 6.15 – 5.97 (m, 1H), 5.82 – 5.43 (m, 1H), 5.24 – 4.94 (m, 2H), 3.76 – 2.65 (m, 2H), 2.57 – 2.35 (m, 2H)

Preparation of Buta-1,3-dien-2-ylbenzene

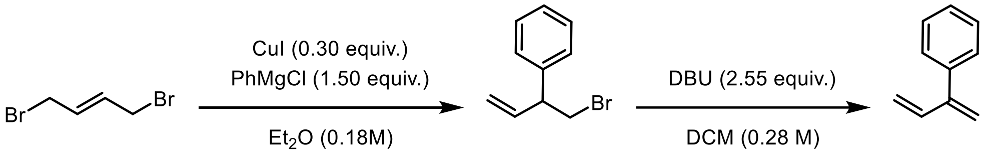

(1-bromobut-3-en-2-yl)benzene (S32)

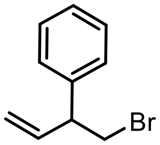

Prepared according to modified literature procedure.[30] Copper iodide (404.6 mg, 2.12 mmol, 0.30 equiv.) and 1,4-dibromo-2-butene (1.50 g, 7.01 mmol, 1.00 equiv.) was taken up in diethyl ether S 4 (40 mL, 0.18 M) in a round bottomed flask equipped with stir bar and cooled to -10 °C. Phenyl magnesium chloride (5.25 mL, 10.5 mmol, 1.5 equiv.) was added dropwise over 5 min to the reaction mixture which then was left to stir at room temperature overnight. The reaction mixture was diluted with sat. aq. NH4Cl (15 mL) extracted with Et2O (3 x 10 mL), combined organic layers washed with brine (3 x 5 mL) three times and dried over MgSO4. Crude (1-bromobut-3-en-2-yl)benzene (**S32**) (52%, 768 mg, 3.64 mmol) was used without purification.

Buta-1,3-dien-2-ylbenzene (2r)

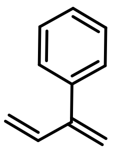

Prepared according to a modified literature procedure.[30] (1-bromobut-3-en-2-yl)benzene (**S32**) (1.16 g, 5.50 mmol, 1.00 equiv.) was taken up in DCM (20 mL, 0.28 M), DBU (2.08 mL, 14.0 mmol, 2.55 equiv.) was added to the reaction mixture which then was heated up to 40 °C in an oil bath for 12h. The reaction mixture was diluted with water (10 mL), extracted with DCM (3 x 5 mL), dried over MgSO4, filtered, and concentrated under reduced pressure. Crude material was purified via column chromatography on SiO2 using pentane. Pure (**2r**) product was isolated as colorless oil (28%, 255.0 mg, 1.96 mmol). Spectral data agree with that reported in the literature.[30]

^{1}H NMR (400MHz, CDCl3) δ 7.35 – 7.26 (m, 5H), 6.66 – 6.57 (m, 1H), 5.29(d, J = 1.7 Hz, 1H), 5.22 – 5.16 (m, 3H).

Preparation of Urea Ligands

General procedure for preparation of monosubstituted and 1,1-disubstituted ureas from amines: Prepared according to literature procedure.[17] To a solution of corresponding primary or secondary amine (1.0 equiv.) in water/glacial acetic acid (1:1, ca. 0.25 M) was added potassium cyanate (1.5 or 3.0 equiv.). The resulting suspension was stirred for 18 h and then quenched with ice. The resulting precipitate was collected by filtration and washed with water to afford the crude product. The crude product was recrystallized in refluxing ethanol/hexanes to give the desired urea.

1-Methyl-1-phenylurea (L3)

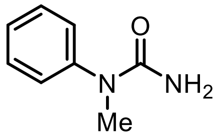

Prepared according to general procedure. N-Methylaniline (1.99 g, 18.6 mmol, 1.0 equiv.), water/glacial acetic acid (1:1, 75 mL, 0.25M), and potassium cyanate (4.53 g, 55.8 mmol, 3.0 equiv.) were used. The recrystallized product was obtained as an off-white crystalline solid (2.40 g, 16.0 mmol, 86%). Spectral data agree with that reported in the literature.[17]

^{1}H NMR (400 MHz, $CDCl_3$) δ 7.41 (t, J = 7.6 Hz, 2H), 7.19 – 7.35 (m, 3H), 5.11 (s, 2H), 3.23 (s, 3H).

1-(4-Methoxyphenyl)urea (L7)

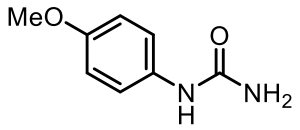

Prepared according to general procedure. 4-Methoxyaniline (1.00 g, 8.10 mmol, 1.0 equiv.), water/glacial acetic acid (1:1, 30.0 mL, 0.27 M), and potassium cyanate (1.97 g, 24.3 mmol, 3.0 equiv.) were used. The recrystallized product was obtained as an off-white solid (713 mg, 4.29 mmol, 53%). Spectral data agree with that reported in the literature.[17]

^{1}H NMR (400 MHz, DMSO-d6) δ 7.21 (d, J = 8.8 Hz, 2H), 6.82 (d, J = 8.8 Hz, 2H), 3.73 (s, 3H).

1-(*o*-tolyl)urea (L9)

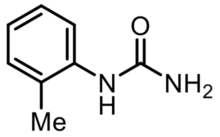

Prepared according to general procedure. o-Toluidine (2.02 g, 18.8 mmol, 1.0 equiv.), water/glacial acetic acid (1:1, 75 mL, 0.25M), and potassium cyanate (4.56 g, 56.4 mmol, 3.0 equiv.) were used. The recrystallized product was obtained as an off-white solid (875 mg, 5.83 mmol, 31%). Spectral data agree with that reported in the literature.[17]

^{1}H NMR (400 MHz, DMSO-d6) δ 7.76 (m, 1H), 7.70 (s, 1H), 7.11 – 7.03 (m, 2H), 6.87 (m, 1H), 6.01 (s, 2H), 2.17 (s, 3H).

1-Mesitylurea (L10)

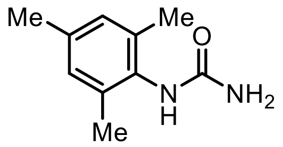

Prepared according to general procedure. 2,4,6-Trimethylaniline (1.00 g, 7.41 mmol, 1.0 equiv.), water/glacial acetic acid (1:1, 30 mL, 0.25M), and potassium cyanate (1.80 g, 22.2 mmol, 3.0 equiv.) were used. The recrystallized product was obtained as a white solid (910 g, 5.11 mmol, 69%). Spectral data agree with that reported in the literature.[17]

^{1}H NMR (500 MHz, DMSO-d6) δ 7.39 (br, 1H), 6.83 (s, 2H), 5.61 (br, 2H), 2.20 (s, 3H), 2.11 (s, 6H).

1-Benzylurea (L13)

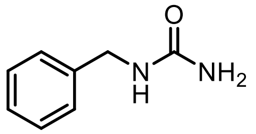

Prepared according to general procedure. Benzylamine (1.80 g, 16.8 mmol, 1.0 equiv.), water/glacial acetic acid (1:1, 24 mL, 0.70M), and potassium cyanate (2.04 g, 25.2 mmol, 1.5 equiv.) were used. The recrystallized product was obtained as a white crystalline solid (990 mg, 6.59 mmol, 39%). Spectral data agree with that reported in the literature.[17]

^{1}H NMR (400 MHz, $CDCl_3$) δ 7.33 – 7.14 (m, 5H), 6.40 (s, 1H), 5.22 (s, 2H), 4.17 (s, 2H).

General procedure for preparation of 1,3 disubstituted and 1,3 trisubstituted ureas from amines: Prepared according to literature procedure.[17] To an oven-dried round bottom flask was added hexanes (50.0 mL) at rt. Phenyl isocyanate (1.00 equiv.) and relevant amine (1.00 equiv.) were added dropwise while stirring. Upon completion (~20 min), the resulting urea crashed out in solution, which was filtered and rinsed with hexanes resulting in the pure ligand as a powder. Column chromatography was performed where noted.

1-Butyl-3-phenylurea (L4)

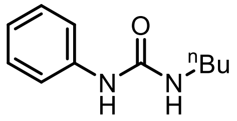

Prepared according to general procedure. To a solution of phenyl isocyanate (1.00 mL, 9.16 mmol, 1.0 equiv.) in hexanes (40.0 mL, 0.23 M), *n*–butylamine (0.910 mL, 9.16 mmol, 1.0 equiv.). The immediately formed white suspension was stirred vigorously for 10 minutes, filtered, and washed with hexanes (2 X 100 mL) to afford the product as a white solid (1.62 g, 8.42 mmol, 92%). Spectral data agree with that reported in the literature.[17]

^{1}H NMR (400 MHz, DMSO-d6) δ 8.36 (s, 1H), 7.38 (d, J = 7.6 Hz, 2H), 7.20 (dd, J = 7.6, 7.2 Hz, 2H), 6.87 (t, J = 7.2 Hz, 1H), 6.01 (t, J = 5.6 Hz, 1H), 3.08 (q, J = 5.6 Hz, 2H), 1.44 – 1.26 (m, 4H), 0.89 (t, J = 7.2 Hz, 3H).

***N*-Phenylpiperidine-1-carboxamide (L5)**

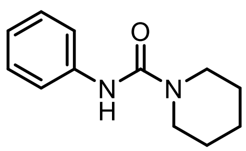

Prepared according to the general procedure. Phenyl isocyanate (0.842 mL, 7.70 mmol, 1.0 equiv.) was added to hexanes (50.0 mL, 0.15 M). Piperidine (2.30 mL, 23.1 mmol, 3.0 equiv.) was then added dropwise. The reaction was allowed to stir for 20 minutes, and the desired urea precipitated from solution. The product was filtered and rinsed with hexanes to afford the product as a white solid in quantitative yield. No further purification was required. Spectral data agree with that reported in the literature.[31]

^{1}H NMR (400 MHz, DMSO-d6) δ 8.45 (s, 1H), 7.49 (dd, J = 8.8 Hz, 8 Hz, 2H), 7.25 (t, J = 7.6 Hz, 2H), 6.95 (m, 1H), 3.45 (m, 4H), 1.60 (m, 2H), 1.52 (m, 4H).

***N*-(*p*-tolyl)piperidine-1-carboxamide (L6)**

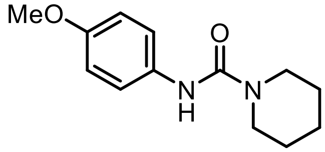

Prepared according to the general procedure. 4-methoxyphenyl isocyanate (1.00 mL, 7.70 mmol, 1.0 equiv.) was added to hexanes (50.0 mL, 0.15 M). Piperidine (2.30 mL, 23.1 mmol, 3.0 equiv.) was then added dropwise. The reaction was allowed to stir for 20 minutes, and the desired urea precipitated from solution. The product was filtered and rinsed with hexanes to afford the product as a white solid in quantitative yield. No further purification was required. Spectral data agree with that reported in the literature.[31]

^{1}H NMR (400 MHz, DMSO-d6) δ 8.28 (s, 1H), 7.36 (d, *J* = 9.1 Hz, 2H), 6.84 (d, *J* = 9.1 Hz, 2H), 3.74 (s, 3H), 3.42 (t, *J* = 5.4 Hz, 4H), 1.60 (m, 2H), 1.51 (m, 4H).

Reaction Condition Optimization

Base Screening

Entry #	Base	3aa Yield	d.r.
1	Li_2CO_3	<5%	45:55
2	Na_2CO_3	10%	44:56
3	K_2CO_3	72%	47:53
4	Rb_2CO_3	56%	45:55
5	Cs_2CO_3	26%	43:57
6	K_3PO_4	58%	-
7	KCN	41%	-
8	KOAc	82%	-
9	KOH	13%	-
10	KO^{t}Bu	<5%	-
11	Pyridine	<5%	-
12	Et_3N	<5%	-
13	No Base	<5%	-

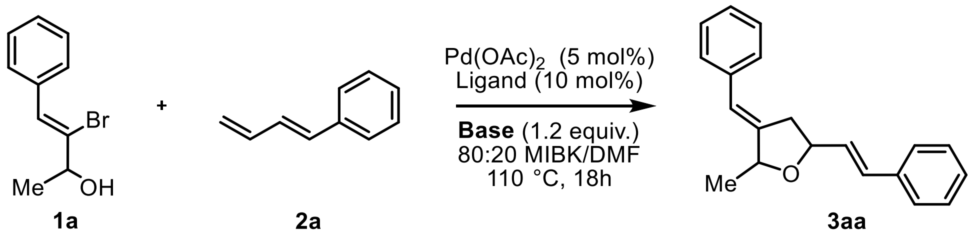

General Procedure for Base Optimization Studies (General Optimization Procedure 1): (*Z*)-3-bromo-4-phenylbut-3-en-2-ol **1a** (113.6 mg, 0.50 mmol, 1.0 equiv.), N-phenyl urea (6.8 mg, 0.050 mmol, 0.1 equiv.), nitrobenzene (25 μL, 0.243 mmol, 0.49 equiv.), and (*E*)-1-phenyl-1,3-butadiene **2a** (84 μL, 0.60 mmol, 1.2 equiv.) were weighed/measured out in the above-mentioned order into a flame-dried 1-dram vial equipped with a small football-shaped stir bar and a cap with a silicone septum. 1.0 mL stock solution was prepped (in a flame-dried 1-dram vial equipped with a small football-shaped stir bar and a cap with a silicone septum) with solvent (0.2 mL DMF, 0.8 mL MIBK) and palladium acetate (5.6 mg, 0.025 mmol, 0.05 equiv.) and left to stir for 30 minutes. Then, the stock solution was added to the first vial and left to stir for several minutes at room temperature. Base (0.60 mmol, 1.2 equiv.) was added to the reaction mixture and a fresh septum was placed on the vial. The reaction mixture was then stirred at 110 °C in an aluminum block for 18 h at 650 rpm. Lastly, a 10 μL aliquot of solution was passed through a plug of celite and analyzed via HPLC. Yield of **3aa** was then determined by quantitative HPLC using nitrobenzene as an internal standard.

Entry 1 – Lithium Carbonate:

General optimization procedure 1 was followed: 1.0 mL stock solution $Pd(OAc)_2$ (5.6 mg, 0.025 mmol, 0.05 equiv.), N-phenyl urea (6.8 mg, 0.050 mmol, 0.1 equiv.), Bromoallyl alcohol **1a** (113.6 mg, 0.50 mmol, 1.00 equiv.), Li_2CO_3 (44.3 mg, 0.60 mmol, 1.2 equiv.) and diene **2a** (84 μL, 0.60

mmol, 1.2 equiv.). Yield determined by quantitative HPLC with nitrobenzene standard. cis/trans (45:55)

Run 1: (<5%)
Run 2: (<5%)
Average: <5% yield

Entry 2 – Sodium Carbonate:

General optimization procedure 1 was followed: 1.0 mL stock solution $Pd(OAc)_2$ (5.6 mg, 0.025 mmol, 0.05 equiv.), N-phenyl urea (6.8 mg, 0.050 mmol, 0.1 equiv.), Bromoallyl alcohol **1a** (113.6 mg, 0.50 mmol, 1.00 equiv.), Na_2CO_3 (63.6 mg, 0.60 mmol, 1.2 equiv.) and diene **2a** (84 µL, 0.60 mmol, 1.2 equiv.). Yield determined by quantitative HPLC with nitrobenzene standard. cis/trans (44:56)

Run 1: (0.057 mmol, 11%)
Run 2: (0.039 mmol, 8%)
Average: 10% yield

Entry 3 – Potassium Carbonate:

General optimization procedure 1 was followed: 1.0 mL stock solution $Pd(OAc)_2$ (5.6 mg, 0.025 mmol, 0.05 equiv.), N-phenyl urea (6.8 mg, 0.050 mmol, 0.1 equiv.), Bromoallyl alcohol **1a** (113.6 mg, 0.50 mmol, 1.00 equiv.), K_2CO_3 (83.0 mg, 0.60 mmol, 1.2 equiv.) and diene **2a** (84 µL, 0.60 mmol, 1.2 equiv.). Yield determined by quantitative HPLC with nitrobenzene standard. cis/trans (47:53)

Run 1: (0.348 mmol, 70%)
Run 2: (0.372 mmol, 74%)
Average: 72% yield

Entry 4 – Rubidium Carbonate:

General optimization procedure 1 was followed: 1.0 mL stock solution $Pd(OAc)_2$ (5.6 mg, 0.025 mmol, 0.05 equiv.), N-phenyl urea (6.8 mg, 0.050 mmol, 0.1 equiv.), Bromoallyl alcohol **1a** (113.6 mg, 0.50 mmol, 1.00 equiv.), Rb_2CO_3 (138.6 mg, 0.60 mmol, 1.2 equiv.) and diene **2a** (84 µL, 0.60 mmol, 1.2 equiv.). Yield determined by quantitative HPLC with nitrobenzene standard. cis/trans (45:55)

Run 1: (0.285 mmol, 57%)
Run 2: (0.273 mmol, 55%)
Average: 56% yield

Entry 5 – Cesium Carbonate:

General optimization procedure 1 was followed: 1.0 mL stock solution $Pd(OAc)_2$ (5.6 mg, 0.025 mmol, 0.05 equiv.), N-phenyl urea (6.8 mg, 0.050 mmol, 0.1 equiv.), Bromoallyl alcohol **1a** (113.6 mg, 0.50 mmol, 1.00 equiv.), Cs_2CO_3 (195.5 mg, 0.60 mmol, 1.2 equiv.) and diene **2a** (84 µL, 0.60 mmol, 1.2 equiv.). Yield determined by quantitative HPLC with nitrobenzene standard. cis/trans (43:57)

Run 1: (0.125 mmol, 25%)
Run 2: (0.131 mmol, 26%)
Average: 26% yield

Entry 6 – Potassium Phosphate Tribasic:

General optimization procedure 1 was followed: 1.0 mL stock solution $Pd(OAc)_2$ (5.6 mg, 0.025 mmol, 0.05 equiv.), N-phenyl urea (6.8 mg, 0.050 mmol, 0.1 equiv.), Bromoallyl alcohol **1a** (113.6 mg, 0.50 mmol, 1.00 equiv.), K_3PO_4 (127.4 mg, 0.60 mmol, 1.2 equiv.) and diene **2a** (84 µL, 0.60 mmol, 1.2 equiv.). Yield determined by quantitative HPLC with nitrobenzene standard.

Run 1: (0.290 mmol, 58%)
Run 2: (0.283 mmol, 57%)
Average: 58% yield

Entry 7 – Potassium Cyanide:

General optimization procedure 1 was followed: 1.0 mL stock solution $Pd(OAc)_2$ (5.6 mg, 0.025 mmol, 0.05 equiv.), N-phenyl urea (6.8 mg, 0.050 mmol, 0.1 equiv.), Bromoallyl alcohol **1a** (113.6 mg, 0.50 mmol, 1.00 equiv.), KCN (39.0 mg, 0.60 mmol, 1.2 equiv.) and diene **2a** (84 µL, 0.60 mmol, 1.2 equiv.). Yield determined by quantitative HPLC with nitrobenzene standard.

Run 1: (0.208 mmol, 42%)
Run 2: (0.199 mmol, 40%)
Average: 41% yield

Entry 8 – Potassium Acetate:

General optimization procedure 1 was followed: 1.0 mL stock solution $Pd(OAc)_2$ (5.6 mg, 0.025 mmol, 0.05 equiv.), N-phenyl urea (6.8 mg, 0.050 mmol, 0.1 equiv.), Bromoallyl alcohol **1a** (113.6 mg, 0.50 mmol, 1.00 equiv.), KOAc (59.0 mg, 0.60 mmol, 1.2 equiv.) and diene **2a** (84 µL, 0.60 mmol, 1.2 equiv.). Yield determined by quantitative HPLC with nitrobenzene standard.

Run 1: (0.415 mmol, 83%)
Run 2: (0.407 mmol, 81%)
Average: 82% yield

Entry 9 – Potassium Hydroxide:

General optimization procedure 1 was followed: 1.0 mL stock solution $Pd(OAc)_2$ (5.6 mg, 0.025 mmol, 0.05 equiv.), N-phenyl urea (6.8 mg, 0.050 mmol, 0.1 equiv.), Bromoallyl alcohol **1a** (113.6 mg, 0.50 mmol, 1.00 equiv.), KOH (33.7 mg, 0.60 mmol, 1.2 equiv.) and diene **2a** (84 µL, 0.60 mmol, 1.2 equiv.). Yield determined by quantitative HPLC with nitrobenzene standard.

Run 1: (0.053 mmol, 11%)
Run 2: (0.072 mmol, 14%)
Average: 13% yield

Entry 10 – Potassium tert-Butoxide:

General optimization procedure 1 was followed: 1.0 mL stock solution Pd(OAc)$_2$ (5.6 mg, 0.025 mmol, 0.05 equiv.), N-phenyl urea (6.8 mg, 0.050 mmol, 0.1 equiv.), Bromoallyl alcohol **1a** (113.6 mg, 0.50 mmol, 1.00 equiv.) KO^{t}Bu (67.3 mg, 0.60 mmol, 1.2 equiv.) and diene **2a** (84 µL, 0.60 mmol, 1.2 equiv.). Yield determined by quantitative HPLC with nitrobenzene standard.

Run 1: (<5%)
Run 2: (<5%)
Average: <5% yield

Entry 11 – Pyridine:

General optimization procedure 1 was followed: 1.0 mL stock solution Pd(OAc)$_2$ (5.6 mg, 0.025 mmol, 0.05 equiv.), N-phenyl urea (6.8 mg, 0.050 mmol, 0.1 equiv.), Bromoallyl alcohol **1a** (113.6 mg, 0.50 mmol, 1.00 equiv.), Pyridine (47.5 mg, 0.60 mmol, 1.2 equiv.) and diene **2a** (84 µL, 0.60 mmol, 1.2 equiv.). Yield determined by quantitative HPLC with nitrobenzene standard.

Run 1: (<5%)
Run 2: (<5%)
Average: <5% yield

Entry 12 – Triethylamine:

General optimization procedure 1 was followed: 1.0 mL stock solution Pd(OAc)$_2$ (5.6 mg, 0.025 mmol, 0.05 equiv.), N-phenyl urea (6.8 mg, 0.050 mmol, 0.1 equiv.), Bromoallyl alcohol **1a** (113.6 mg, 0.50 mmol, 1.00 equiv.), Et$_3$N (60.7 mg, 0.60 mmol, 1.2 equiv.) and diene **2a** (84 µL, 0.60 mmol, 1.2 equiv.). Yield determined by quantitative HPLC with nitrobenzene standard.

Run 1: (<5%)
Run 2: (<5%)
Average: <5% yield

Entry 13 – No Base:

General optimization procedure 1 was followed: 1.0 mL stock solution Pd(OAc)$_2$ (5.6 mg, 0.025 mmol, 0.05 equiv.), N-phenyl urea (6.8 mg, 0.050 mmol, 0.1 equiv.), Bromoallyl alcohol **1a** (113.6 mg, 0.50 mmol, 1.00 equiv.), and diene **2a** (84 µL, 0.60 mmol, 1.2 equiv.). Yield determined by quantitative HPLC with nitrobenzene standard.

Run 1: (<5%)
Run 2: (<5%)
Average: <5% yield

Solvent Screening

Entry #	Solvent	3aa Yield
1	MIBK	19%
2	1:9 DMF/MIBK	41%
3	1:6 DMF/MIBK	63%
4	1:4 DMF/MIBK	72%
5	(1M) 1:4 DMF/MIBK	65%
6	1:3 DMF/MIBK	64%
7	1:2 DMF/MIBK	61%
8	1:1 DMF/MIBK	51%
9	3:1 DMF/MIBK	39%
10	DMF	47%
11	1:1 DMF/nBuOAc	43%
12	nBuOAc	7%
13	DMSO	65%
14	1:4 DMSO/MIBK	55%
15	Dioxane	<5%
16	Toluene	<5%

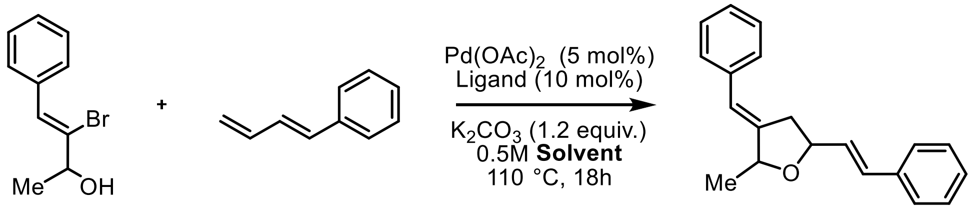

1a **2a** **3aa**

General Procedure for Solvent Optimization Studies (General Optimization Procedure 2): (*Z*)-3-bromo-4-phenylbut-3-en-2-ol **1a** (113.6 mg, 0.50 mmol, 1.0 equiv.), N-phenyl urea (6.8 mg, 0.050 mmol, 0.1 equiv.), nitrobenzene (25 μL, 0.243 mmol, 0.49 equiv.), and (*E*)-1-phenyl-1,3-butadiene **2a** (84 μL, 0.60 mmol, 1.2 equiv.) were weighed/measured out in the above-mentioned order into a flame-dried 1-dram vial equipped with a small football-shaped stir bar and a cap with a silicone septum. 1.0 mL stock solution was prepped (in a flame-dried 1-dram vial equipped with a small football-shaped stir bar and a cap with a silicone septum) with solvent (1 mL) and palladium acetate (5.6 mg, 0.025 mmol, 0.05 equiv.) and left to stir for 30 minutes. Then, the stock solution was added to the first vial and left to stir for several minutes at room temperature. Base (0.60 mmol, 1.2 equiv.) was added to the reaction mixture and a fresh septum was placed on the vial. The reaction mixture was then stirred at 110 °C in an aluminum block for 18 h at 650 rpm. Lastly, a 10 μL aliquot of solution was passed through a plug of celite and analyzed via HPLC. Yield of **3aa** was then determined by quantitative HPLC using nitrobenzene as an internal standard.

Entry 1 – MIBK:

General optimization procedure 2 was followed: 1.0 mL stock solution $Pd(OAc)_2$ (5.6 mg, 0.025 mmol, 0.05 equiv.), N-phenyl urea (6.8 mg, 0.050 mmol, 0.1 equiv.), Bromoallyl alcohol **1a** (113.6 mg, 0.50 mmol, 1.00 equiv.), K_2CO_3 (83.0 mg, 0.60 mmol, 1.2 equiv.) and diene **2a** (84 μL, 0.60 mmol, 1.2 equiv.). Yield determined by quantitative HPLC with nitrobenzene standard.

<u>Run 1:</u> (0.091 mmol, 18%)

Run 2: (0.102 mmol, 20%)
Average: 19% yield

Entry 2 – 1:9 DMF/MIBK:

General optimization procedure 2 was followed: 1.0 mL stock solution $Pd(OAc)_2$ (5.6 mg, 0.025 mmol, 0.05 equiv.), N-phenyl urea (6.8 mg, 0.050 mmol, 0.1 equiv.), Bromoallyl alcohol **1a** (113.6 mg, 0.50 mmol, 1.00 equiv.), K_2CO_3 (83.0 mg, 0.60 mmol, 1.2 equiv.) and diene **2a** (84 µL, 0.60 mmol, 1.2 equiv.). Yield determined by quantitative HPLC with nitrobenzene standard.

Run 1: (0.193 mmol, 39%)
Run 2: (0.208 mmol, 42%)
Average: 41% yield

Entry 3 – 1:6 DMF/MIBK:

General optimization procedure 2 was followed: 1.0 mL stock solution $Pd(OAc)_2$ (5.6 mg, 0.025 mmol, 0.05 equiv.), N-phenyl urea (6.8 mg, 0.050 mmol, 0.1 equiv.), Bromoallyl alcohol **1a** (113.6 mg, 0.50 mmol, 1.00 equiv.), K_2CO_3 (83.0 mg, 0.60 mmol, 1.2 equiv.) and diene **2a** (84 µL, 0.60 mmol, 1.2 equiv.). Yield determined by quantitative HPLC with nitrobenzene standard.

Run 1: (0.311 mmol, 62%)
Run 2: (0.315 mmol, 63%)
Average: 63% yield

Entry 4 – 1:4 DMF/MIBK:

General optimization procedure 2 was followed: 1.0 mL stock solution $Pd(OAc)_2$ (5.6 mg, 0.025 mmol, 0.05 equiv.), N-phenyl urea (6.8 mg, 0.050 mmol, 0.1 equiv.), Bromoallyl alcohol **1a** (113.6 mg, 0.50 mmol, 1.00 equiv.), K_2CO_3 (83.0 mg, 0.60 mmol, 1.2 equiv.) and diene **2a** (84 µL, 0.60 mmol, 1.2 equiv.). Yield determined by quantitative HPLC with nitrobenzene standard.

Run 1: (0.357 mmol, 71%)
Run 2: (0.366 mmol, 73%)
Average: 72% yield

Entry 5 – (1M) 1:4 DMF/MIBK:

General optimization procedure 2 was followed: 0.5 mL stock solution ($Pd(OAc)_2$ (5.6 mg, 0.025 mmol, 0.05 equiv.), N-phenyl urea (6.8 mg, 0.050 mmol, 0.1 equiv.), Bromoallyl alcohol **1a** (113.6 mg, 0.50 mmol, 1.00 equiv.), K_2CO_3 (83.0 mg, 0.60 mmol, 1.2 equiv.) and diene **2a** (84 µL, 0.60 mmol, 1.2 equiv.). Yield determined by quantitative HPLC with nitrobenzene standard.

Run 1: (0.318 mmol, 64%)
Run 2: (0.324 mmol, 65%)
Average: 65% yield

Entry 6 – 1:3 DMF/MIBK:

General optimization procedure 2 was followed: 1.0 mL stock solution $Pd(OAc)_2$ (5.6 mg, 0.025 mmol, 0.05 equiv.), N-phenyl urea (6.8 mg, 0.050 mmol, 0.1 equiv.), Bromoallyl alcohol **1a** (113.6 mg, 0.50 mmol, 1.00 equiv.), K_2CO_3 (83.0 mg, 0.60 mmol, 1.2 equiv.) and diene **2a** (84 µL, 0.60 mmol, 1.2 equiv.). Yield determined by quantitative HPLC with nitrobenzene standard.

Run 1: (0.309 mmol, 62%)
Run 2: (0.331 mmol, 66%)
Average: 64% yield

Entry 7 – 1:2 DMF/MIBK:

General optimization procedure 2 was followed: 1.0 mL stock solution $Pd(OAc)_2$ (5.6 mg, 0.025 mmol, 0.05 equiv.), N-phenyl urea (6.8 mg, 0.050 mmol, 0.1 equiv.), Bromoallyl alcohol **1a** (113.6 mg, 0.50 mmol, 1.00 equiv.), K_2CO_3 (83.0 mg, 0.60 mmol, 1.2 equiv.) and diene **2a** (84 µL, 0.60 mmol, 1.2 equiv.). Yield determined by quantitative HPLC with nitrobenzene standard.

Run 1: (0.294 mmol, 59%)
Run 2: (0.311 mmol, 62%)
Average: 61% yield

Entry 8 – 1:1 DMF/MIBK:

General optimization procedure 2 was followed: 1.0 mL stock solution $Pd(OAc)_2$ (5.6 mg, 0.025 mmol, 0.05 equiv.), N-phenyl urea (6.8 mg, 0.050 mmol, 0.1 equiv.), Bromoallyl alcohol **1a** (113.6 mg, 0.50 mmol, 1.00 equiv.), K_2CO_3 (83.0 mg, 0.60 mmol, 1.2 equiv.) and diene **2a** (84 µL, 0.60 mmol, 1.2 equiv.). Yield determined by quantitative HPLC with nitrobenzene standard.

Run 1: (0.256 mmol, 51%)
Run 2: (0.251 mmol, 50%)
Average: 51% yield

Entry 9 – 3:1 DMF/MIBK:

General optimization procedure 2 was followed: 1.0 mL stock solution $Pd(OAc)_2$ (5.6 mg, 0.025 mmol, 0.05 equiv.), N-phenyl urea (6.8 mg, 0.050 mmol, 0.1 equiv.), Bromoallyl alcohol **1a** (113.6 mg, 0.50 mmol, 1.00 equiv.), K_2CO_3 (83.0 mg, 0.60 mmol, 1.2 equiv.) and diene **2a** (84 µL, 0.60 mmol, 1.2 equiv.). Yield determined by quantitative HPLC with nitrobenzene standard.

Run 1: (0.202 mmol, 40%)
Run 2: (0.188 mmol, 38%)
Average: 39% yield

Entry 10 – DMF:

General optimization procedure 2 was followed: 1.0 mL stock solution $Pd(OAc)_2$ (5.6 mg, 0.025 mmol, 0.05 equiv.), N-phenyl urea (6.8 mg, 0.050 mmol, 0.1 equiv.), Bromoallyl alcohol **1a** (113.6 mg, 0.50 mmol, 1.00 equiv.), K_2CO_3 (83.0 mg, 0.60 mmol, 1.2 equiv.) and diene **2a** (84 µL, 0.60 mmol, 1.2 equiv.). Yield determined by quantitative HPLC with nitrobenzene standard.

Run 1: (0.236 mmol, 47%)

Run 2: (0.233 mmol, 47%)
Average: 47% yield

Entry 11 – 1:1 DMF/nBuOAc:

General optimization procedure 2 was followed: 1.0 mL stock solution $Pd(OAc)_2$ (5.6 mg, 0.025 mmol, 0.05 equiv.), N-phenyl urea (6.8 mg, 0.050 mmol, 0.1 equiv.), Bromoallyl alcohol **1a** (113.6 mg, 0.50 mmol, 1.00 equiv.), K_2CO_3 (83.0 mg, 0.60 mmol, 1.2 equiv.) and diene **2a** (84 µL, 0.60 mmol, 1.2 equiv.). Yield determined by quantitative HPLC with nitrobenzene standard.

Run 1: (0.206 mmol, 41%)
Run 2: (0.218 mmol, 44%)
Average: 43% yield

Entry 12 – nBuOAc:

General optimization procedure 2 was followed: 1.0 mL stock solution $Pd(OAc)_2$ (5.6 mg, 0.025 mmol, 0.05 equiv.), N-phenyl urea (6.8 mg, 0.050 mmol, 0.1 equiv.), Bromoallyl alcohol **1a** (113.6 mg, 0.50 mmol, 1.00 equiv.), K_2CO_3 (83.0 mg, 0.60 mmol, 1.2 equiv.) and diene **2a** (84 µL, 0.60 mmol, 1.2 equiv.). Yield determined by quantitative HPLC with nitrobenzene standard.

Run 1: (0.029 mmol, 6%)
Run 2: (0.041 mmol, 8%)
Average: 7% yield

Entry 13 – DMSO:

General optimization procedure 2 was followed: 1.0 mL stock solution $Pd(OAc)_2$ (5.6 mg, 0.025 mmol, 0.05 equiv.), N-phenyl urea (6.8 mg, 0.050 mmol, 0.1 equiv.), Bromoallyl alcohol **1a** (113.6 mg, 0.50 mmol, 1.00 equiv.), K_2CO_3 (83.0 mg, 0.60 mmol, 1.2 equiv.) and diene **2a** (84 µL, 0.60 mmol, 1.2 equiv.). Yield determined by quantitative HPLC with nitrobenzene standard.

Run 1: (0.318 mmol, 64%)
Run 2: (0.331 mmol, 66%)
Average: 65% yield

Entry 14 – 1:4 DMSO/MIBK:

General optimization procedure 2 was followed: 1.0 mL stock solution $Pd(OAc)_2$ (5.6 mg, 0.025 mmol, 0.05 equiv.), N-phenyl urea (6.8 mg, 0.050 mmol, 0.1 equiv.), Bromoallyl alcohol **1a** (113.6 mg, 0.50 mmol, 1.00 equiv.), K_2CO_3 (83.0 mg, 0.60 mmol, 1.2 equiv.) and diene **2a** (84 µL, 0.60 mmol, 1.2 equiv.). Yield determined by quantitative HPLC with nitrobenzene standard.

Run 1: (0.270 mmol, 54%)
Run 2: (0.276 mmol, 55%)
Average: 55% yield

Entry 15 – Dioxane:

General optimization procedure 2 was followed: 1.0 mL stock solution $Pd(OAc)_2$ (5.6 mg, 0.025 mmol, 0.05 equiv.), N-phenyl urea (6.8 mg, 0.050 mmol, 0.1 equiv.), Bromoallyl alcohol **1a** (113.6 mg, 0.50 mmol, 1.00 equiv.), K_2CO_3 (83.0 mg, 0.60 mmol, 1.2 equiv.) and diene **2a** (84 µL, 0.60 mmol, 1.2 equiv.). Yield determined by quantitative HPLC with nitrobenzene standard.

Run 1: (<5%)
Run 2: (<5%)
Average: <5% yield

Entry 16 – Toluene:

General optimization procedure 2 was followed: 1.0 mL stock solution $Pd(OAc)_2$ (5.6 mg, 0.025 mmol, 0.05 equiv.), N-phenyl urea (6.8 mg, 0.050 mmol, 0.1 equiv.), Bromoallyl alcohol **1a** (113.6 mg, 0.50 mmol, 1.00 equiv.), K_2CO_3 (83.0 mg, 0.60 mmol, 1.2 equiv.) and diene **2a** (84 µL, 0.60 mmol, 1.2 equiv.). Yield determined by quantitative HPLC with nitrobenzene standard.

Run 1: (<5%)
Run 2: (<5%)
Average: <5% yield

Additive Screen

Entry #	Reaction Tolerance + Additives	3aa Yield
1	1 equiv. H_2O	61%
2	5 equiv. H_2O	69%
3	Under N_2	57%
4	1 equiv. TBAI	11%
5	1 equiv. TBABr	<5%
6	1 equiv. TBACl	<5%
7	1 equiv. LiCl	<5%

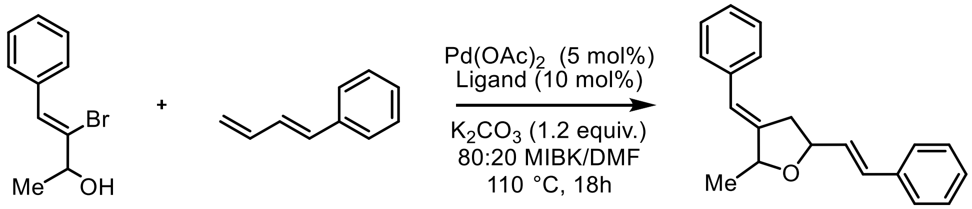

General Procedure for Additive Optimization Studies (General Optimization Procedure 3): (*Z*)-3-bromo-4-phenylbut-3-en-2-ol **1a** (113.6 mg, 0.50 mmol, 1.0 equiv.), N-phenyl urea (6.8 mg, 0.050 mmol, 0.1 equiv.), nitrobenzene (25 µL, 0.243 mmol, 0.49 equiv.), and (*E*)-1-phenyl-1,3-butadiene **2a** (84 µL, 0.60 mmol, 1.2 equiv.) were weighed/measured out in the above-mentioned order into a flame-dried 1-dram vial equipped with a small football-shaped stir bar and a cap with a silicone septum. 1.0 mL stock solution was prepped (in a flame-dried 1-dram vial equipped with a small football-shaped stir bar and a cap with a silicone septum) with solvent (1 mL) and palladium acetate (5.6 mg, 0.025 mmol, 0.05 equiv.) and left to stir for 30 minutes. Then, the stock solution was added to the first vial and left to stir for several minutes at room temperature. Potassium carbonate (83.0 mg, 0.60 mmol, 1.2 equiv.) was added to the reaction mixture and a fresh septum was placed on the vial. The reaction mixture was then stirred at 110 °C in an

aluminum block for 18 h at 650 rpm. Lastly, a 10 µL aliquot of solution was passed through a plug of celite and analyzed via HPLC. Yield of **3aa** was then determined by quantitative HPLC using nitrobenzene as an internal standard.

Entry 1 – 1 equiv. H_2O:

General optimization procedure 3 was followed: 1.0 mL stock solution $Pd(OAc)_2$ (5.6 mg, 0.025 mmol, 0.05 equiv.), N-phenyl urea (6.8 mg, 0.050 mmol, 0.1 equiv.), Bromoallyl alcohol **1a** (113.6 mg, 0.50 mmol, 1.00 equiv.), K_2CO_3 (83.0 mg, 0.60 mmol, 1.2 equiv.), diene **2a** (84 µL, 0.60 mmol, 1.2 equiv.) and H_2O (9 µL, 0.50 mmol, 1.00 equiv.). Yield determined by quantitative HPLC with nitrobenzene standard.

<u>Run 1:</u> (0.313 mmol, 63%)
<u>Run 2:</u> (0.290 mmol, 58%)
Average: 61% yield

Entry 2 – 5 equiv. H_2O:

General optimization procedure 3 was followed: 1.0 mL stock solution $Pd(OAc)_2$ (5.6 mg, 0.025 mmol, 0.05 equiv.), N-phenyl urea (6.8 mg, 0.050 mmol, 0.1 equiv.), Bromoallyl alcohol **1a** (113.6 mg, 0.50 mmol, 1.00 equiv.), K_2CO_3 (83.0 mg, 0.60 mmol, 1.2 equiv.), diene **2a** (84 µL, 0.60 mmol, 1.2 equiv.) and H_2O (45 µL, 2.50 mmol, 5.00 equiv.). Yield determined by quantitative HPLC with nitrobenzene standard.

<u>Run 1:</u> (0.331, 67%)
<u>Run 2:</u> (0.352, 70%)
Average: 69% yield

Entry 3 – Under N_2:

General optimization procedure 3 was followed: 1.0 mL stock solution $Pd(OAc)_2$ (5.6 mg, 0.025 mmol, 0.05 equiv.), N-phenyl urea (6.8 mg, 0.050 mmol, 0.1 equiv.), Bromoallyl alcohol **1a** (113.6 mg, 0.50 mmol, 1.00 equiv.), K_2CO_3 (83.0 mg, 0.60 mmol, 1.2 equiv.), diene **2a** (84 µL, 0.60 mmol, 1.2 equiv.) All added under a balloon of nitrogen and vial sealed with electrical tape. Yield determined by quantitative HPLC with nitrobenzene standard.

<u>Run 1:</u> (0.274, 55%)
<u>Run 2:</u> (0.294, 59%)
Average: 57% yield

Entry 4 – 1 equiv. TBAI:

General optimization procedure 3 was followed: 1.0 mL stock solution $Pd(OAc)_2$ (5.6 mg, 0.025 mmol, 0.05 equiv.), N-phenyl urea (6.8 mg, 0.050 mmol, 0.1 equiv.), Bromoallyl alcohol **1a** (113.6 mg, 0.50 mmol, 1.00 equiv.), K_2CO_3 (83.0 mg, 0.60 mmol, 1.2 equiv.), diene **2a** (84 µL, 0.60 mmol, 1.2 equiv.) and TBAI (184.7 mg, 0.50 mmol, 1.00 equiv.). Yield determined by quantitative HPLC with nitrobenzene standard.

<u>Run 1:</u> (0.048, 10%)
<u>Run 2:</u> (0.051, 12%)
Average: 11% yield

Entry 5 – 1 equiv. TBABr:

General optimization procedure 3 was followed: 1.0 mL stock solution $Pd(OAc)_2$ (5.6 mg, 0.025 mmol, 0.05 equiv.), N-phenyl urea (6.8 mg, 0.050 mmol, 0.1 equiv.), Bromoallyl alcohol **1a** (113.6 mg, 0.50 mmol, 1.00 equiv.), K_2CO_3 (83.0 mg, 0.60 mmol, 1.2 equiv.), diene **2a** (84 µL, 0.60 mmol, 1.2 equiv.) and TBABr (161.2 mg, 0.50 mmol, 1.00 equiv.). Yield determined by quantitative HPLC with nitrobenzene standard.

Run 1: (<5%)
Run 2: (<5%)
Average: <5% yield

Entry 6 – 1 equiv. TBACl:

General optimization procedure 3 was followed: 1.0 mL stock solution $Pd(OAc)_2$ (5.6 mg, 0.025 mmol, 0.05 equiv.), N-phenyl urea (6.8 mg, 0.050 mmol, 0.1 equiv.), Bromoallyl alcohol **1a** (113.6 mg, 0.50 mmol, 1.00 equiv.), K_2CO_3 (83.0 mg, 0.60 mmol, 1.2 equiv.), diene **2a** (84 µL, 0.60 mmol, 1.2 equiv.) and TBACl (139.0 mg, 0.50 mmol, 1.00 equiv.). Yield determined by quantitative HPLC with nitrobenzene standard.

Run 1: (<5%)
Run 2: (<5%)
Average: <5% yield

Entry 7 – 1 equiv. LiCl:

General optimization procedure 3 was followed: 1.0 mL stock solution $Pd(OAc)_2$ (5.6 mg, 0.025 mmol, 0.05 equiv.), N-phenyl urea (6.8 mg, 0.050 mmol, 0.1 equiv.), Bromoallyl alcohol **1a** (113.6 mg, 0.50 mmol, 1.00 equiv.), K_2CO_3 (83.0 mg, 0.60 mmol, 1.2 equiv.), diene **2a** (84 µL, 0.60 mmol, 1.2 equiv.) and LiCl (21.2 mg, 0.50 mmol, 1.00 equiv.). Yield determined by quantitative HPLC with nitrobenzene standard.

Run 1: (<5%)
Run 2: (<5%)
Average: <5% yield

Temperature Screen

Entry #	Temperature (°C)	3aa Yield
1	80	58%
2	90	59%
3	100	67%
4	110	72%
5	120	61%

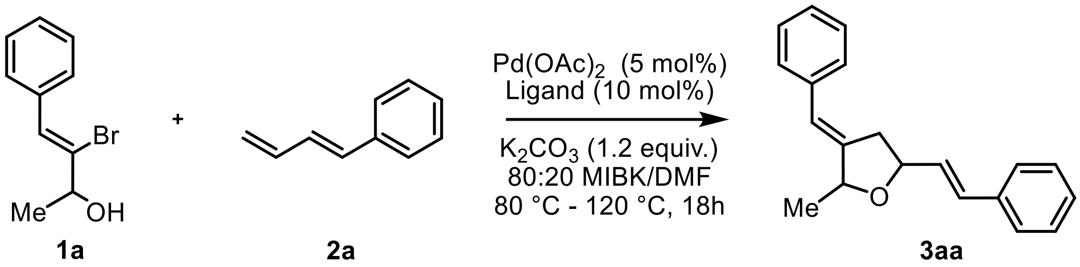

General Procedure for Temperature Optimization Studies (General Optimization Procedure 4):

(*Z*)-3-bromo-4-phenylbut-3-en-2-ol **1a** (113.6 mg, 0.50 mmol, 1.0 equiv.), N-phenyl urea (6.8 mg, 0.050 mmol, 0.1 equiv.), nitrobenzene (25 µL, 0.243 mmol, 0.49 equiv.), and (*E*)-1-phenyl-1,3-butadiene **2a** (84 µL, 0.60 mmol, 1.2 equiv.) were weighed/measured out in the above-mentioned order into a flame-dried 1-dram vial equipped with a small football-shaped stir bar and a cap with a silicone septum. 1.0 mL stock solution was prepped (in a flame-dried 1-dram vial equipped with a small football-shaped stir bar and a cap with a silicone septum) with solvent (1 mL) and palladium acetate (5.6 mg, 0.025 mmol, 0.05 equiv.) and left to stir for 30 minutes. Then, the stock solution was added to the first vial and left to stir for several minutes at room temperature. Potassium carbonate (83.0 mg, 0.60 mmol, 1.2 equiv.) was added to the reaction mixture and a fresh septum was placed on the vial. The reaction mixture was then stirred and heated to 110 °C in an oil bath for 18 h at 650 rpm. Lastly, a 10 µL aliquot of solution was passed through a plug of celite and analyzed via HPLC. Yield of **3aa** was then determined by quantitative HPLC using nitrobenzene as an internal standard.

Entry 1 – 80 °C:

General optimization procedure 4 was followed: 1.0 mL stock solution $Pd(OAc)_2$ (5.6 mg, 0.025 mmol, 0.05 equiv.), N-phenyl urea (6.8 mg, 0.050 mmol, 0.1 equiv.), Bromoallyl alcohol **1a** (113.6 mg, 0.50 mmol, 1.00 equiv.), K_2CO_3 (83.0 mg, 0.60 mmol, 1.2 equiv.), diene **2a** (84 µL, 0.60 mmol, 1.2 equiv.) and H_2O (9 µL, 0.50 mmol, 1.00 equiv.). Yield determined by quantitative HPLC with nitrobenzene standard.

Run 1: (0.286 mmol, 57%)
Run 2: (0.297 mmol, 59%)
Average: 58% yield

Entry 2 – 90 °C:

General optimization procedure 4 was followed: 1.0 mL stock solution $Pd(OAc)_2$ (5.6 mg, 0.025 mmol, 0.05 equiv.), N-phenyl urea (6.8 mg, 0.050 mmol, 0.1 equiv.), Bromoallyl alcohol **1a** (113.6 mg, 0.50 mmol, 1.00 equiv.), K_2CO_3 (83.0 mg, 0.60 mmol, 1.2 equiv.), diene **2a** (84 µL, 0.60 mmol, 1.2 equiv.) and H_2O (9 µL, 0.50 mmol, 1.00 equiv.). Yield determined by quantitative HPLC with nitrobenzene standard.

Run 1: (0.295 mmol, 59%)
Run 2: (0.288 mmol, 58%)
Average: 59% yield

Entry 3 – 100 °C:

General optimization procedure 4 was followed: 1.0 mL stock solution $Pd(OAc)_2$ (5.6 mg, 0.025 mmol, 0.05 equiv.), N-phenyl urea (6.8 mg, 0.050 mmol, 0.1 equiv.), Bromoallyl alcohol **1a** (113.6 mg, 0.50 mmol, 1.00 equiv.), K_2CO_3 (83.0 mg, 0.60 mmol, 1.2 equiv.), diene **2a** (84 µL, 0.60 mmol, 1.2 equiv.) and H_2O (9 µL, 0.50 mmol, 1.00 equiv.). Yield determined by quantitative HPLC with nitrobenzene standard.

Run 1: (0.333 mmol, 67%)
Run 2: (0.336 mmol, 67%)
Average: 67% yield

Entry 4 – 110 °C:

General optimization procedure 4 was followed: 1.0 mL stock solution $Pd(OAc)_2$ (5.6 mg, 0.025 mmol, 0.05 equiv.), N-phenyl urea (6.8 mg, 0.050 mmol, 0.1 equiv.), Bromoallyl alcohol **1a** (113.6 mg, 0.50 mmol, 1.00 equiv.), K_2CO_3 (83.0 mg, 0.60 mmol, 1.2 equiv.), diene **2a** (84 µL, 0.60 mmol, 1.2 equiv.) and H_2O (9 µL, 0.50 mmol, 1.00 equiv.). Yield determined by quantitative HPLC with nitrobenzene standard.

Run 1: (0.348 mmol, 70%)
Run 2: (0.369 mmol, 74%)
Average: 72% yield

Entry 5 – 120 °C:

General optimization procedure 4 was followed: 1.0 mL stock solution $Pd(OAc)_2$ (5.6 mg, 0.025 mmol, 0.05 equiv.), N-phenyl urea (6.8 mg, 0.050 mmol, 0.1 equiv.), Bromoallyl alcohol **1a** (113.6 mg, 0.50 mmol, 1.00 equiv.), K_2CO_3 (83.0 mg, 0.60 mmol, 1.2 equiv.), diene **2a** (84 µL, 0.60 mmol, 1.2 equiv.) and H_2O (9 µL, 0.50 mmol, 1.00 equiv.). Yield determined by quantitative HPLC with nitrobenzene standard.

Run 1: (0.314 mmol, 63%)
Run 2: (0.294 mmol, 59%)
Average: 61% yield

Palladium Source/Loading Screen

Entry #	Palladium Source/ Pd:Ligand Loading	3aa Yield
1	$PdCl_2$	38%
2	$Pd(dba)_2$	<5%
3	No Pd/10 mol% ligand	<5%
4	2.5/5 mol%	63%
5	5/5 mol%	60%
6	2.5/10 mol%	61%

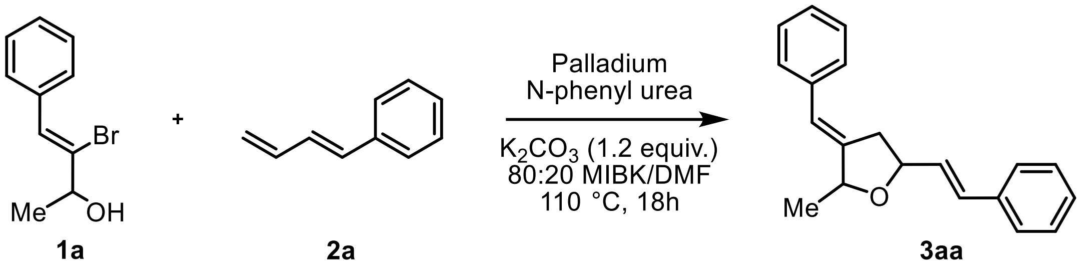

General Procedure for Palladium Source/Loading Screen Optimization Studies (General Optimization Procedure 5):
(*Z*)-3-bromo-4-phenylbut-3-en-2-ol **1a** (113.6 mg, 0.50 mmol, 1.0 equiv.), N-phenyl urea (6.8 mg, 0.050 mmol, 0.1 equiv.), nitrobenzene (25 µL, 0.243 mmol, 0.49 equiv.), and (*E*)-1-phenyl-1,3-butadiene **2a** (84 µL, 0.60 mmol, 1.2 equiv.) were weighed/measured out in the above-mentioned order into a flame-dried 1-dram vial equipped with a small football-shaped stir bar and a cap with a silicone septum. 1.0 mL stock solution was prepped (in a flame-dried 1-dram vial equipped with a small football-shaped stir bar and a cap with a silicone septum) with solvent (1 mL) and palladium acetate (5.6 mg, 0.025 mmol, 0.05 equiv.) and left to stir for 30 minutes. Then, the stock solution was added to the first vial and left to stir for several minutes at room temperature. Potassium carbonate (83.0 mg, 0.60 mmol, 1.2 equiv.) was added to the reaction mixture and a fresh septum was placed on the vial. The reaction mixture was then stirred and heated to 110 °C in an oil bath for 18 h at 650 rpm. Lastly, a 10 µL aliquot of solution was passed through a plug of celite and analyzed via HPLC. Yield of **3aa** was then determined by quantitative HPLC using nitrobenzene as an internal standard.

Entry 1 – $PdCl_2$:

General optimization procedure 5 was followed: 1.0 mL stock solution $PdCl_2$ (4.4 mg, 0.025 mmol, 0.05 equiv.), N-phenyl urea (6.8 mg, 0.050 mmol, 0.1 equiv.), Bromoallyl alcohol **1a** (113.6 mg, 0.50 mmol, 1.00 equiv.), K_2CO_3 (83.0 mg, 0.60 mmol, 1.2 equiv.), and diene **2a** (84 µL, 0.60 mmol, 1.2 equiv. Yield determined by quantitative HPLC with nitrobenzene standard.

Run 1: (0.193, 39%)
Run 2: (0.184, 37%)
Average: 38% yield

Entry 2 – $Pd(dba)_2$:

General optimization procedure 5 was followed: 1.0 mL stock solution $Pd(dba)_2$ (14.4 mg, 0.025 mmol, 0.05 equiv.), N-phenyl urea (6.8 mg, 0.050 mmol, 0.1 equiv.), Bromoallyl alcohol **1a** (113.6 mg, 0.50 mmol, 1.00 equiv.), K_2CO_3 (83.0 mg, 0.60 mmol, 1.2 equiv.), and diene **2a** (84 µL, 0.60 mmol, 1.2 equiv.). Yield determined by quantitative HPLC with nitrobenzene standard.

Run 1: (<5%)
Run 2: (<5%)
Average: <5% yield

Entry 3 – No Pd/10 mol% ligand:

General optimization procedure 5 was followed: 1.0 mL solvent (0.2 mL DMF, 0.8 mL MIBK), N-phenyl urea (6.8 mg, 0.050 mmol, 0.1 equiv.), Bromoallyl alcohol **1a** (113.6 mg, 0.50 mmol, 1.00

equiv.), K_2CO_3 (83.0 mg, 0.60 mmol, 1.2 equiv.), and diene **2a** (84 µL, 0.60 mmol, 1.2 equiv.). Yield determined by quantitative HPLC with nitrobenzene standard.

Run 1: (<5%)
Run 2: (<5%)
Average: <5% yield

Entry 4 – 2.5/5 mol%:

General optimization procedure 5 was followed: 1.0 mL stock solution $Pd(OAc)_2$ (2.8 mg, 0.0125 mmol, 0.025 equiv.), N-phenyl urea (3.4 mg, 0.025 mmol, 0.05 equiv.), Bromoallyl alcohol **1a** (113.6 mg, 0.50 mmol, 1.00 equiv.), K_2CO_3 (83.0 mg, 0.60 mmol, 1.2 equiv.), and diene **2a** (84 µL, 0.60 mmol, 1.2 equiv.). Yield determined by quantitative HPLC with nitrobenzene standard.

Run 1: (0.321, 64%)
Run 2: (0.310, 62%)
Average: 63% yield

Entry 5 – 5/5 mol%:

General optimization procedure 5 was followed: 1.0 mL stock solution $Pd(OAc)_2$ (5.6 mg, 0.025 mmol, 0.050 equiv.), N-phenyl urea (3.4 mg, 0.025 mmol, 0.05 equiv.), Bromoallyl alcohol **1a** (113.6 mg, 0.50 mmol, 1.00 equiv.), K_2CO_3 (83.0 mg, 0.60 mmol, 1.2 equiv.), and diene **2a** (84 µL, 0.60 mmol, 1.2 equiv.). Yield determined by quantitative HPLC with nitrobenzene standard.

Run 1: (0.301, 60%)
Run 2: (0.298, 60%)
Average: 60% yield

Entry 6 – 2.5/10 mol%:

General optimization procedure 5 was followed: 1.0 mL stock solution $Pd(OAc)_2$ (2.8 mg, 0.0125 mmol, 0.025 equiv.), N-phenyl urea (6.8 mg, 0.050 mmol, 0.1 equiv.), Bromoallyl alcohol **1a** (113.6 mg, 0.50 mmol, 1.00 equiv.), K_2CO_3 (83.0 mg, 0.60 mmol, 1.2 equiv.), and diene **2a** (84 µL, 0.60 mmol, 1.2 equiv.). Yield determined by quantitative HPLC with nitrobenzene standard.

Run 1: (0.294, 59%)
Run 2: (0.311, 62%)
Average: 61% yield

Ligand Screen

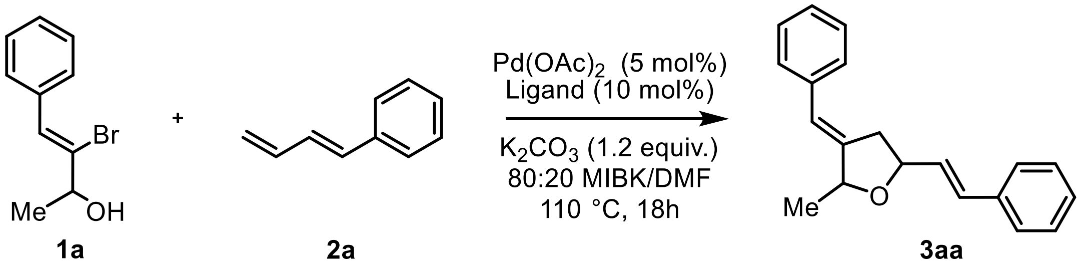

1a **2a** **3aa**

General Procedure for Ligand Optimization Studies (General Optimization Procedure 6): (*Z*)-3-bromo-4-phenylbut-3-en-2-ol **1a** (56.8 mg, 0.25 mmol, 1.0 equiv.), ligand (0.025 mmol, 0.1 equiv.), nitrobenzene (15 µL, 0.146 mmol, 0.58 equiv.), and (*E*)-1-phenyl-1,3-butadiene **2a** (42 µL, 0.30 mmol, 1.2 equiv.) were weighed/measured out in the above-mentioned order into a flame-dried 1-dram vial equipped with a small football-shaped stir bar and a cap with a silicone septum. 0.5 mL stock solution was prepped (in a flame-dried 1-dram vial equipped with a small football-shaped stir bar and a cap with a silicone septum) with solvent (0.1 mL DMF, 0.4 mL MIBK) and palladium acetate (2.8 mg, 0.0125 mmol, 0.05 equiv.) and left to stir for 30 minutes. Then, the stock solution was added to the first vial and left to stir for several minutes at room temperature. Potassium carbonate base (41.5 mg, 0.30 mmol, 1.2 equiv.) was added to the reaction mixture and a fresh septum was placed on the vial. The reaction mixture was then stirred at 110 °C in an aluminum block for 18 h at 650 rpm. Lastly, a 10 µL aliquot of solution was passed through a plug of celite and analyzed via HPLC. Yield of **3aa** was then determined by quantitative HPLC using nitrobenzene as an internal standard.

L1 – Urea:

General optimization procedure 6 was followed: 0.5 mL stock solution $Pd(OAc)_2$ (2.8 mg, 0.0125 mmol, 0.05 equiv.), urea (L1) (1.5 mg, 0.025 mmol, 0.1 equiv.)), Bromoallyl alcohol **1a** (56.8 mg, 0.25 mmol, 1.00 equiv.), K_2CO_3 (41.5 mg, 0.30 mmol, 1.2 equiv.) and diene **2a** (42 µL, 0.30 mmol, 1.2 equiv.). Yield determined by quantitative HPLC with nitrobenzene standard. cis/trans (42:58)

Run 1: (0.055 mmol, 22%)
Run 2: (0.059 mmol, 24%)
Run 3: (0.064 mmol, 26%)
Average: 24% yield

L2 – 1-Phenylurea:

General optimization procedure 6 was followed: 0.5 mL stock solution $Pd(OAc)_2$ (2.8 mg, 0.0125 mmol, 0.05 equiv.), 1-Phenylurea (L2) (3.4 mg, 0.025 mmol, 0.1 equiv.)), Bromoallyl alcohol **1a** (56.8 mg, 0.25 mmol, 1.00 equiv.), K_2CO_3 (41.5 mg, 0.30 mmol, 1.2 equiv.) and diene **2a** (42 µL, 0.30 mmol, 1.2 equiv.). Yield determined by quantitative HPLC with nitrobenzene standard. cis/trans (47:53)

Run 1: (0.197 mmol, 79%)
Run 2: (0.190 mmol, 76%)
Run 3: (0.188 mmol, 75%)
Average: 77% yield

L3 – 1-Methyl-1-phenylurea:

General optimization procedure 6 was followed: 0.5 mL stock solution $Pd(OAc)_2$ (2.8 mg, 0.0125 mmol, 0.05 equiv.), 1-Methyl-1-phenylurea (L3) (3.8 mg, 0.025 mmol, 0.1 equiv.)), Bromoallyl alcohol **1a** (56.8 mg, 0.25 mmol, 1.00 equiv.), K_2CO_3 (41.5 mg, 0.30 mmol, 1.2 equiv.) and diene **2a** (42 µL, 0.30 mmol, 1.2 equiv.). Yield determined by quantitative HPLC with nitrobenzene standard. cis/trans (46:54)

Run 1: (0.181 mmol, 72%)
Run 2: (0.178 mmol, 71%)
Run 3: (0.181 mmol, 72%)

Average: 72% yield

L4 – 1-Butyl-3-phenylurea:

General optimization procedure 6 was followed: 0.5 mL stock solution $Pd(OAc)_2$ (2.8 mg, 0.0125 mmol, 0.05 equiv.), 1-Butyl-3-phenylurea (L4) (4.8 mg, 0.025 mmol, 0.1 equiv.)), Bromoallyl alcohol **1a** (56.8 mg, 0.25 mmol, 1.00 equiv.), K_2CO_3 (41.5 mg, 0.30 mmol, 1.2 equiv.) and diene **2a** (42 µL, 0.30 mmol, 1.2 equiv.). Yield determined by quantitative HPLC with nitrobenzene standard. cis/trans (46:54)

Run 1: (0.077 mmol, 31%)
Run 2: (0.085 mmol, 34%)
Run 3: (0.079 mmol, 31%)
Average: 32% yield

L5 – *N*-Phenylpiperidine-1-carboxamide:

General optimization procedure 6 was followed: 0.5 mL stock solution $Pd(OAc)_2$ (2.8 mg, 0.0125 mmol, 0.05 equiv.), *N*-Phenylpiperidine-1-carboxamide (L5) (5.1 mg, 0.025 mmol, 0.1 equiv.)), Bromoallyl alcohol **1a** (56.8 mg, 0.25 mmol, 1.00 equiv.), K_2CO_3 (41.5 mg, 0.30 mmol, 1.2 equiv.) and diene **2a** (42 µL, 0.30 mmol, 1.2 equiv.). Yield determined by quantitative HPLC with nitrobenzene standard. cis/trans (46:54)

Run 1: (0.101 mmol, 40%)
Run 2: (0.112 mmol, 45%)
Run 3: (0.108 mmol, 43%)
Average: 43% yield

L6 – *N*-(4-methoxyphenyl)piperidine-1-carboxamide:

General optimization procedure 6 was followed: 0.5 mL stock solution $Pd(OAc)_2$ (2.8 mg, 0.0125 mmol, 0.05 equiv.), *N*-(4-methoxyphenyl)piperidine-1-carboxamide (L6) (5.9 mg, 0.025 mmol, 0.1 equiv.)), Bromoallyl alcohol **1a** (56.8 mg, 0.25 mmol, 1.00 equiv.), K_2CO_3 (41.5 mg, 0.30 mmol, 1.2 equiv.) and diene **2a** (42 µL, 0.30 mmol, 1.2 equiv.). Yield determined by quantitative HPLC with nitrobenzene standard. cis/trans (43:57)

Run 1: (0.090 mmol, 36%)
Run 2: (0.100 mmol, 40%)
Run 3: (0.098 mmol, 39%)
Average: 38% yield

L7 – 1-(4-(Trifluoromethyl)phenyl)urea:

General optimization procedure 6 was followed: 0.5 mL stock solution $Pd(OAc)_2$ (2.8 mg, 0.0125 mmol, 0.05 equiv.), 1-(4-(Trifluoromethyl)phenyl)urea (L7) (5.1 mg, 0.025 mmol, 0.1 equiv.)), Bromoallyl alcohol **1a** (56.8 mg, 0.25 mmol, 1.00 equiv.), K_2CO_3 (41.5 mg, 0.30 mmol, 1.2 equiv.) and diene **2a** (42 µL, 0.30 mmol, 1.2 equiv.). Yield determined by quantitative HPLC with nitrobenzene standard. cis/trans (45:55)

Run 1: (0.157 mmol, 63%)
Run 2: (0.166 mmol, 67%)

Run 3: (0.166 mmol, 67%)
Average: 66% yield

L8 – 1-(4-Methoxyphenyl)urea:

General optimization procedure 6 was followed: 0.5 mL stock solution $Pd(OAc)_2$ (2.8 mg, 0.0125 mmol, 0.05 equiv.), 1-(4-Methoxyphenyl)urea (L8) (4.2 mg, 0.025 mmol, 0.1 equiv.)), Bromoallyl alcohol **1a** (56.8 mg, 0.25 mmol, 1.00 equiv.), K_2CO_3 (41.5 mg, 0.30 mmol, 1.2 equiv.) and diene **2a** (42 µL, 0.30 mmol, 1.2 equiv.). Yield determined by quantitative HPLC with nitrobenzene standard. cis/trans (45:55)

Run 1: (0.179 mmol, 72%)
Run 2: (0.171 mmol, 68%)
Run 3: (0.180 mmol, 72%)
Average: 71% yield

L9 – 1-(*o*-tolyl)urea:

General optimization procedure 6 was followed: 0.5 mL stock solution $Pd(OAc)_2$ (2.8 mg, 0.0125 mmol, 0.05 equiv.), 1-(*o*-tolyl)urea (L9) (3.8 mg, 0.025 mmol, 0.1 equiv.)), Bromoallyl alcohol **1a** (56.8 mg, 0.25 mmol, 1.00 equiv.), K_2CO_3 (41.5 mg, 0.30 mmol, 1.2 equiv.) and diene **2a** (42 µL, 0.30 mmol, 1.2 equiv.). Yield determined by quantitative HPLC with nitrobenzene standard. cis/trans (44:56)

Run 1: (0.177 mmol, 71%)
Run 2: (0.178 mmol, 71%)
Run 3: (0.168 mmol, 67%)
Average: 70% yield

L10 – 1-Mesitylurea:

General optimization procedure 6 was followed: 0.5 mL stock solution $Pd(OAc)_2$ (2.8 mg, 0.0125 mmol, 0.05 equiv.), 1-Mesitylurea (L10) (4.5 mg, 0.025 mmol, 0.1 equiv.)), Bromoallyl alcohol **1a** (56.8 mg, 0.25 mmol, 1.00 equiv.), K_2CO_3 (41.5 mg, 0.30 mmol, 1.2 equiv.) and diene **2a** (42 µL, 0.30 mmol, 1.2 equiv.). Yield determined by quantitative HPLC with nitrobenzene standard. cis/trans (43:57)

Run 1: (0.168 mmol, 67%)
Run 2: (0.171 mmol, 68%)
Run 3: (0.167 mmol, 67%)
Average: 67% yield

L11 – 1-Methylurea:

General optimization procedure 6 was followed: 0.5 mL stock solution $Pd(OAc)_2$ (2.8 mg, 0.0125 mmol, 0.05 equiv.), 1-Methylurea (L11) (1.9 mg, 0.025 mmol, 0.1 equiv.)), Bromoallyl alcohol **1a** (56.8 mg, 0.25 mmol, 1.00 equiv.), K_2CO_3 (41.5 mg, 0.30 mmol, 1.2 equiv.) and diene **2a** (42 µL, 0.30 mmol, 1.2 equiv.). Yield determined by quantitative HPLC with nitrobenzene standard. cis/trans (43:57)

Run 1: (0.146 mmol, 58%)

Run 2: (0.147 mmol, 59%)
Run 3: (0.148 mmol, 59%)
Average: 59% yield

L12 – 1-Cyclohexylurea:

General optimization procedure 6 was followed: 0.5 mL stock solution $Pd(OAc)_2$ (2.8 mg, 0.0125 mmol, 0.05 equiv.), 1-Cyclohexylurea (L12) (3.6 mg, 0.025 mmol, 0.1 equiv.)), Bromoallyl alcohol **1a** (56.8 mg, 0.25 mmol, 1.00 equiv.), K_2CO_3 (41.5 mg, 0.30 mmol, 1.2 equiv.) and diene **2a** (42 μL, 0.30 mmol, 1.2 equiv.). Yield determined by quantitative HPLC with nitrobenzene standard. cis/trans (43:57)

Run 1: (0.164 mmol, 66%)
Run 2: (0.162 mmol, 65%)
Run 3: (0.152 mmol, 61%)
Average: 64% yield

L13 – 1-Benzylurea:

General optimization procedure 6 was followed: 0.5 mL stock solution $Pd(OAc)_2$ (2.8 mg, 0.0125 mmol, 0.05 equiv.), 1-Benzylurea (L13) (3.8 mg, 0.025 mmol, 0.1 equiv.)), Bromoallyl alcohol **1a** (56.8 mg, 0.25 mmol, 1.00 equiv.), K_2CO_3 (41.5 mg, 0.30 mmol, 1.2 equiv.) and diene **2a** (42 μL, 0.30 mmol, 1.2 equiv.). Yield determined by quantitative HPLC with nitrobenzene standard. cis/trans (43:57)

Run 1: (0.169 mmol, 68%)
Run 2: (0.178 mmol, 71%)
Run 3: (0.164 mmol, 66%)
Average: 68% yield

No Ligand:

General optimization procedure 6 was followed: 0.5 mL stock solution $Pd(OAc)_2$ (2.8 mg, 0.0125 mmol, 0.05 equiv.), bromoallyl alcohol **1a** (56.8 mg, 0.25 mmol, 1.00 equiv.), K_2CO_3 (41.5 mg, 0.30 mmol, 1.2 equiv.) and diene **2a** (42 μL, 0.30 mmol, 1.2 equiv.). Yield determined by quantitative HPLC with nitrobenzene standard. cis/trans (43:57)

Run 1: (0.074 mmol, 30%)
Run 2: (0.075 mmol, 30%)
Run 3: (0.074 mmol, 30%)
Average: 30% yield

Optimized Reaction Conditions with Aromatic Ambiphiles

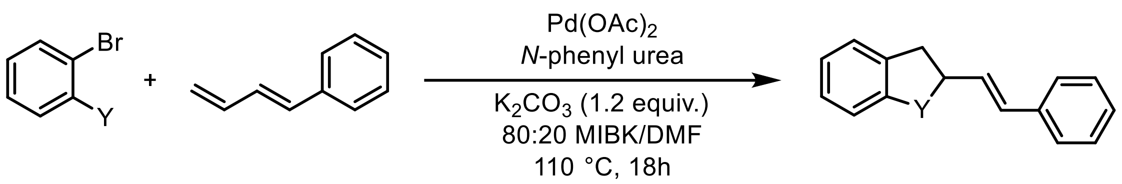

General Procedure for Aromatic Ambiphiles (General Optimization Procedure 7):
Ambiphile **1** (0.50 mmol, 1.0 equiv.), N-phenyl urea (6.8 mg, 0.050 mmol, 0.1 equiv.), and (*E*)-1-phenyl-1,3-butadiene **2a** (84 µL, 0.60 mmol, 1.2 equiv.) were weighed/measured out in the above-mentioned order into a flame-dried 1-dram vial equipped with a small football-shaped stir bar and a cap with a silicone septum. 1.0 mL stock solution was prepared (in a flame-dried 1-dram vial equipped with a small football-shaped stir bar and a cap with a silicone septum) with solvent (0.2 mL DMF, 0.8 mL MIBK) and palladium acetate (5.6 mg, 0.025 mmol, 0.05 equiv.) and left to stir for 30 min. Then, the stock solution was added to the first vial and left to stir for several minutes at room temperature. Potassium carbonate base (83 mg, 0.60 mmol, 1.2 equiv.) was added to the reaction mixture and a fresh septum was placed on the vial. The reaction mixture was then stirred at 110 °C in an aluminum block for 18 h at 650 rpm. After cooling to rt, the crude material was filtered through a plug of celite using ethyl acetate. The crude material was purified by flash column chromatography to determine isolated yield.

(*E*)-2-styryl-2,3-dihydrobenzofuran (S33)

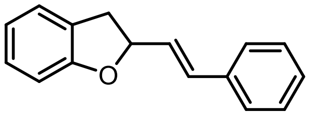

General Optimization Procedure 7 was followed. 2-bromophenol (86.5 mg, 0.50 mmol, 1.0 equiv.), N-phenyl urea (6.8 mg, 0.050 mmol, 0.1 equiv.), (*E*)-1-phenyl-1,3-butadiene **2a** (84 µL, 0.60 mmol, 1.2 equiv.), K_2CO_3 (83 mg, 0.60 mmol, 1.2 equiv.), and 1 mL of stock solution was used. The crude material was purified as a yellow solid using flash chromatography (silica, 100% hexanes - 95/5 hexanes/EtOAc) Spectral data agree with that reported in the literature.[31]

^{1}H NMR (400 MHz, $CDCl_3$) δ 7.51 (m, 2H), 7.43 (m, 2H), 7.37 (m, 1H), 7.32 – 7.22 (m, 2H), 7.04 – 6.94 (m, 2H), 6.81 (d, *J* = 15.8 Hz, 1H), 6.47 (dd, *J* = 15.8, 7.3 Hz, 1H), 5.50 – 5.39 (dt, J = 15.8 Hz, 9.1 Hz, 1H), 3.51 (dd, *J* = 15.6, 9.2 Hz, 1H), 3.17 (dd, *J* = 15.6, 7.8 Hz, 1H).

<u>2.5/5 mol% Pd/urea loading:</u>

Run 1: (97.0 mg, 0.436 mmol, 87%)
Run 2: (91.4 mg, 0.411 mmol, 82%)
Run 3: (92.6 mg, 0.417 mmol, 83%)
Average: 84% yield vs 74%[31]

5/10 mol% Pd/urea loading:

Run 1: (93.1 mg, 0.419 mmol, 84%)
Run 2: (90.0 mg, 0.405 mmol, 81%)
Run 3: (87.4 mg, 0.393 mmol, 79%)
Average: 81% yield

(*E*)-2-styryl-1-tosylindoline (S34)

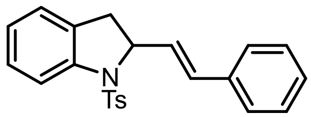

General scope procedure was followed. (2-bromophenyl)(tosyl)-λ^2-azane (163.1 mg, 0.50 mmol, 1.0 equiv.), N-phenyl urea (6.8 mg, 0.050 mmol, 0.1 equiv.), (*E*)-1-phenyl-1,3-butadiene **2a** (84 μL, 0.60 mmol, 1.2 equiv.), K_2CO_3 (83 mg, 0.60 mmol, 1.2 equiv.), and 1 mL of stock solution was used. The crude material was purified as a white foam using flash chromatography (silica, 100% hexanes - 95/5 hexanes/EtOAc) Spectral data agree with that reported in the literature. [17]

^{1}H NMR (400 MHz, $CDCl_3$) δ 7.68 (d, J = 8.0 Hz, 1H), 7.62 (d, J = 8.2 Hz, 2H), 7.33 – 7.28 (m, 4H), 7.23 (d, J = 7.0 Hz, 2H), 7.15 (d, J = 8.1 Hz, 2H), 7.09 – 7.01 (m, 2H), 6.69 (d, J = 15.8 Hz, 1H), 6.16 (dd, J = 15.8, 7.2 Hz, 1H), 4.97 (td, J = 6.9 Hz, 2.6 Hz, 1H), 3.08 (dd, J = 16.0, 9.6 Hz, 1H), 2.73 (dd, J = 16.0, 2.6 Hz, 1H), 2.37 (d, J = 19.7 Hz, 3H).

2.5/5 mol% Pd/urea loading:

Run 1: (158.7 mg, 0.423 mmol, 85%)
Run 2: (157.2 mg, 0.419 mmol, 84%)
Run 3: (149.5 mg, 0.398 mmol, 80%)
Average: 83% yield vs. 89%[17]

5/10 mol% Pd/urea loading:

Run 1: (156.1 mg, 0.416 mmol, 83%)
Run 2: (152.3 mg, 0.406 mmol, 81%)
Run 3: (153.2 mg, 0.408 mmol, 82%)
Average: 82% yield

Substrate Scope
2-Bromoallyl Alcohol Scope

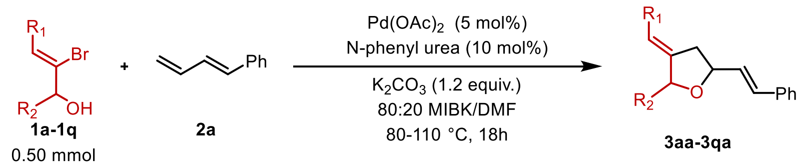

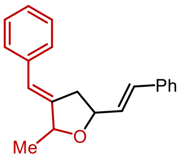

72%
3aa

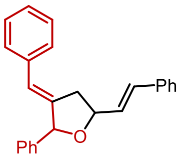

71%
3ba

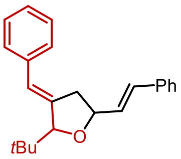

19%
3ca

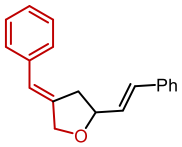

46%
3da

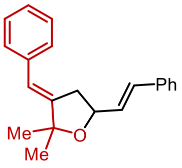

76%
3ea

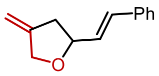

29%
3fa

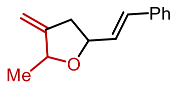

35%
3ga

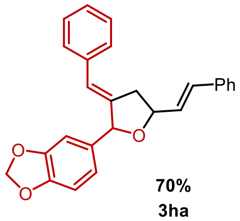

70%
3ha

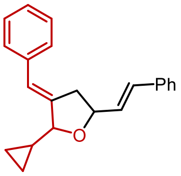

47%
3ia

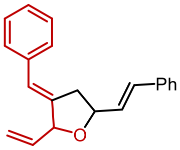

17%
3ja

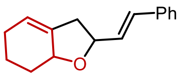

28%
3ka

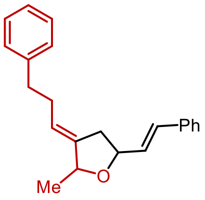

54%
3la

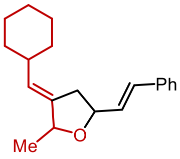

37%
3ma

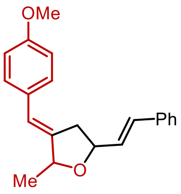

73%
3na

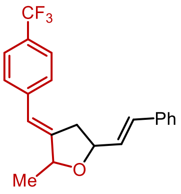

58%
3oa

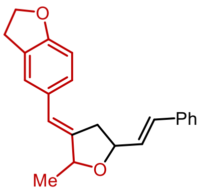

63%
3pa

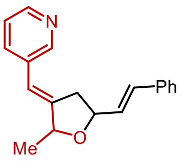

41%
3qa

General Procedure for 2-Bromoallyl Alcohol Scope (General Scope Procedure):
Bromoallyl alcohol **1a-1r** (0.50 mmol, 1.0 equiv.), N-phenyl urea (6.8 mg, 0.050 mmol, 0.1 equiv.), and (*E*)-1-phenyl-1,3-butadiene **2a** (84 µL, 0.60 mmol, 1.2 equiv.) were weighed/measured out in the above-mentioned order into a flame-dried 1-dram vial equipped with a small football-shaped stir bar and a cap with a silicone septum. 1.0 mL stock solution was prepared (in a flame-dried 1-dram vial equipped with a small football-shaped stir bar and a cap with a silicone septum) with solvent (0.2 mL DMF, 0.8 mL MIBK) and palladium acetate (5.6 mg, 0.025 mmol, 0.05 equiv.) and left to stir for 30 min. Then, the stock solution was added to the first vial and left to stir for several minutes at room temperature. Potassium carbonate base (83 mg, 0.60 mmol, 1.2 equiv.) was added to the reaction mixture and a fresh septum was placed on the vial. The reaction mixture was then stirred at 110 °C in an aluminum block for 18 h at 650 rpm. After cooling to rt, the crude material was filtered through a plug of celite using ethyl acetate. The crude material was then analyzed via NMR to determine the d.r. The crude material was purified by flash column chromatography to determine isolated yield.

3-((*E*)-benzylidene)-2-methyl-5-((*E*)-styryl)tetrahydrofuran (3aa)

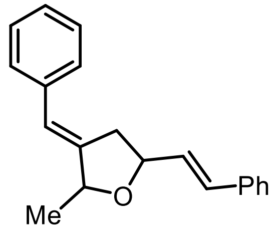

General scope procedure was followed. (*Z*)-3-bromo-4-phenylbut-3-en-2-ol **1a** (113.6 mg, 0.50 mmol, 1.0 equiv.), N-phenyl urea (6.8 mg, 0.050 mmol, 0.1 equiv.), (*E*)-1-phenyl-1,3-butadiene **2a** (84 µL, 0.60 mmol, 1.2 equiv.), K_2CO_3 (83 mg, 0.60 mmol, 1.2 equiv.), and 1 mL of stock solution was used. The crude material was purified as a yellow oil using flash chromatography (silica, 100% hexanes - 95/5 hexanes/EtOAc) cis/trans (47:53)

<u>Run 1:</u> (99.1 mg, 0.359 mmol, 72%)
<u>Run 2:</u> (96.2 mg, 0.348 mmol, 70%)
<u>Run 3:</u> (102.9 mg, 0.372 mmol, 74%)
Average: 72% yield

^{1}H NMR (400 MHz, $CDCl_3$) trans δ 7.47 – 7.29 (m, 10H), 6.72 (d, J = 16.0 Hz, 1H), 6.39 – 6.26 (m, 2H), 4.67 – 4.60 (m, 1H), 4.58 – 4.51 (m, 1H), 3.14 – 3.08 (m, 1H), 2.78 – 2.71 (m, 1H), 1.54 (d, J = 6.2 Hz, 3H)

^{1}H NMR (400 MHz, $CDCl_3$) cis δ 7.47 – 7.29 (m, 10H), 6.68 (d, J = 16.0 Hz, 1H) 6.39 – 6.26 (m, 2H), 4.90 – 4.80 (m, 2H), 3.21 – 3.14 (m, 1H), 2.84 – 2.78 (m, 1H), 1.48 (d, J = 6.4 Hz, 3H)

^{13}C NMR (100 MHz, $CDCl_3$) trans δ 145.7, 137.7, 136.6, 132.3, 129.3, 128.6 (2C), 128.5 (2C), 128.2 (2C), 127.9, 126.7, 126.7 (2C), 120.1, 79.5, 78.7, 38.6, 20.7

^{13}C NMR (100 MHz, $CDCl_3$) cis δ 145.4, 137.7, 136.7, 131.3, 129.7, 128.6 (2C), 128.5 (2C), 128.2 (2C), 127.8, 126.7, 126.6 (2C), 120.4, 78.2, 77.9, 37.6, 21.2

HRMS (ESI) m/z: $[M + H]^+$ Calcd for $C_{20}H_{20}O$ 277.1592; Found 277.1588.

3-((*E*)-benzylidene)-2-phenyl-5-((*E*)-styryl)tetrahydrofuran (3ba)

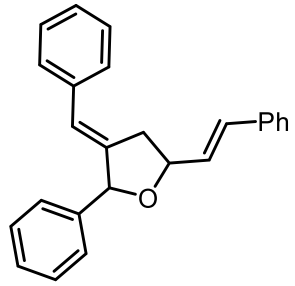

General scope procedure was followed. (*Z*)-2-bromo-1,3-diphenylprop-2-en-1-ol **1b** (144.6 mg, 0.50 mmol, 1.0 equiv.), N-phenyl urea (6.8 mg, 0.050 mmol, 0.1 equiv.), (*E*)-1-phenyl-1,3-butadiene **2a** (84 µL, 0.60 mmol, 1.2 equiv.), K_2CO_3 (83 mg, 0.60 mmol, 1.2 equiv.), and 1 mL of stock solution was used. The crude material was purified as a yellow oil using flash chromatography (silica, 100% hexanes - 95/5 hexanes/EtOAc) cis/trans (47:53)

Run 1: (123.2 mg, 0.364 mmol, 73%)
Run 2: (120.0 mg, 0.355 mmol, 71%)
Run 3: (119.0 mg, 0.352 mmol, 70%)
Average: 71% yield

^{1}H NMR (500 MHz, $CDCl_3$) trans δ 7.50 – 7.19 (m, 15H), 6.77 (d, J = 16.0 Hz, 1H), 6.45 (dd, J = 12.7, 7.0 Hz, 1H), 6.09 (s, 1H), 5.48 (s, 1H), 4.75 – 4.69 (m, 1H), 3.34– 3.22 (m, 1H), 2.97 – 2.86 (m, 1H)

^{1}H NMR (500 MHz, $CDCl_3$) cis δ 7.50 – 7.19 (m, 15H), 6.69 (d, J = 16.0 Hz, 1H), 6.34 (dd, J = 12.7, 6.5 Hz, 1H), 6.24 (s, 1H), 5.69 (s, 1H), 5.01 (q, J = 6.5 Hz, 1H), 3.34 – 3.22 (m, 1H), 2.97 – 2.86 (m, 1H)

^{13}C NMR (125 MHz, $CDCl_3$) trans δ 144.7, 141.9, 137.4, 136.6, 132.3, 129.1, 128.7 (2C), 128.6 (2C), 128.5 (2C), 128.4 (2C), 128.0, 128.0, 128.0 (2C), 127.0, 126.7 (2C), 123.0, 85.5, 84.3, 38.9

^{13}C NMR (125 MHz, $CDCl_3$) cis δ 143.4, 141.5, 137.4, 136.6, 131.5, 129.6, 128.7 (2C), 128.6 (2C), 128.5 (2C), 128.2 (2C), 128.0, 127.9, 127.5 (2C), 126.9, 126.7 (2C), 123.5, 80.1, 79.4, 37.6

HRMS (ESI) m/z: $[M + H]^+$ Calcd for $C_{25}H_{22}O$ 339.1749; Found 339.1750.

3-((*E*)-benzylidene)-2-(tert-butyl)-5-((*E*)-styryl)tetrahydrofuran (3ca)

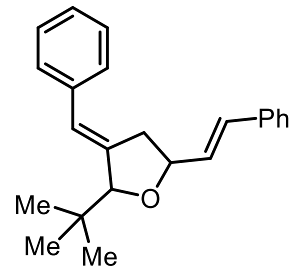

General scope procedure was followed. (*Z*)-2-bromo-4,4-dimethyl-1-phenylpent-1-en-3-ol **1c** (134.6 mg, 0.50 mmol, 1.0 equiv.), N-phenyl urea (6.8 mg, 0.050 mmol, 0.1 equiv.), (*E*)-1-phenyl-1,3-butadiene **2a** (84 µL, 0.60 mmol, 1.2 equiv.), K_2CO_3 (83 mg, 0.60 mmol, 1.2 equiv.), and 1 mL of stock solution was used. The crude material was redissolved in 2mL of MeOH and $NaBH_4$ (50 mg, 1.32 mmol) was added to the above mixture was stirred at rt for 15 min to remove unwanted enone side product. It was then quenched with aq. HCl (2M solution, 10 mL) and extracted with EtOAc (3 x 10 mL), dried over MgSO4, and solvent removed under reduced pressure. The crude material was purified as a yellow oil using flash chromatography (silica, 100% hexanes - 95/5 hexanes/EtOAc) cis/trans cis/trans (46:54)

Run 1: (26.6 mg, 0.084 mmol, 17%)
Run 2: (35.5 mg, 0.111 mmol, 22%)
Run 3: (28.4 mg, 0.089 mmol, 18%)
Average: 19% yield

^{1}H NMR (500 MHz, $CDCl_3$) trans δ 7.43 – 7.14 (m, 10H), 6.66 (d, J = 12.7 Hz, 1H), 6.41 (s, 1H), 6.33 (dd, J = 15.0, 7.0 Hz, 1H), 4.45 – 4.39 (m, 1H), 4.26 (s, 1H), 2.76 (d, J = 15.7 Hz, 1H), 2.63 – 2.54 (m, 1H), 1.03 (s, 9H)

^{1}H NMR (500 MHz, $CDCl_3$) cis δ 7.43 – 7.14 (m, 10H), 6.51 (d, J = 12.6 Hz, 1H), 6.47 (s, 1H), 6.09 (dd, J = 15.5, 7.0 Hz, 1H), 5.00 – 4.95 (m, 1H), 4.34 (s, 1H), 3.15 – 3.07 (m, 1H), 2.96 (dd, J = 14.5, 5.0 Hz, 1H), 1.02 (s, 9H)

^{13}C NMR (125 MHz, $CDCl_3$) trans δ 142.8, 137.7, 136.8, 131.7, 129.7, 128.7 (2C), 128.7 (2C), 128.4 (2C), 127.8, 126.8, 126.7 (2C), 123.6, 90.7, 79.9, 39.2, 36.7, 26.2 (3C)

^{13}C NMR (125 MHz, $CDCl_3$) cis δ 141.3, 137.7, 136.8, 131.0, 130.3, 128.7 (2C), 128.6 (2C), 128.4 (2C), 127.6, 126.8, 126.7 (2C), 124.2, 90.6, 79.7, 37.6, 36.1, 26.2 (3C)

HRMS (ESI) m/z: $[M + H]^+$ Calcd for $C_{23}H_{26}O$ 319.2063; Found 319.2059.

4-((*E*)-benzylidene)-2-((*E*)-styryl)tetrahydrofuran (3da)

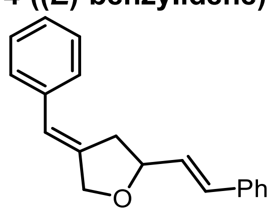

General scope procedure was followed. (*Z*)-2-bromo-3-phenylprop-2-en-1-ol **1d** (106.5 mg, 0.50 mmol, 1.0 equiv.), N-phenyl urea (6.8 mg, 0.050 mmol, 0.1 equiv.), (*E*)-1-phenyl-1,3-butadiene **2a** (84 μL, 0.60 mmol, 1.2 equiv.), K_2CO_3 (83 mg, 0.60 mmol, 1.2 equiv.), and 1 mL of stock solution was used. The crude material was purified as a yellow oil using flash chromatography (silica, 100% hexanes - 95/5 hexanes/EtOAc)

<u>Run 1:</u> (58.9 mg, 0.225 mmol, 45%)
<u>Run 2:</u> (63.6 mg, 0.242 mmol, 48%)
<u>Run 3:</u> (60.2 mg, 0.229 mmol, 46%)
Average: 46% yield

^{1}H NMR (500 MHz, $CDCl_3$) δ 7.45 – 7.35 (m, 10H), 6.69 (d, J = 16.0 Hz, 1H), 6.40 (t, J = 2.4 Hz, 1H), 6.33 (dd, J = 15.8 Hz, 6.8 Hz, 1H), 4.73 – 4.63 (m, 2H), 4.58 – 4.53 (m, 1H), 3.10 (dd, J = 16.2 Hz, 6.4 Hz, 1H), 2.72 (dd, J = 16.2 Hz, 8.0 Hz, 1H)

^{13}C NMR (125 MHz, $CDCl_3$) δ 140.8, 137.6, 136.7, 131.8, 129.2, 128.7 (2C), 128.5 (2C), 128.1 (2C), 127.9, 126.8, 126.7 (2C), 120.0, 81.0, 77.8, 37.9

HRMS (ESI) m/z: $[M + H]^+$ Calcd for $C_{19}H_{18}O$ 263.1437; Found 263.1430.

3-((*E*)-benzylidene)-2,2-dimethyl-5-((*E*)-styryl)tetrahydrofuran (3ea)

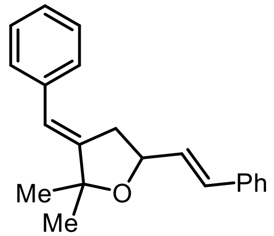

General scope procedure was followed. (*Z*)-3-bromo-2-methyl-4-phenylbut-3-en-2-ol **1e** (120.6 mg, 0.50 mmol, 1.0 equiv.), N-phenyl urea (6.8 mg, 0.050 mmol, 0.1 equiv.), (*E*)-1-phenyl-1,3-butadiene **2a** (84 μL, 0.60 mmol, 1.2 equiv.), K_2CO_3 (83 mg, 0.60 mmol, 1.2 equiv.), and 1 mL of stock solution was used. The crude material was purified as a yellow oil using flash chromatography (silica, 100% hexanes - 95/5 hexanes/EtOAc)

<u>Run 1:</u> (111.6 mg, 0.384 mmol, 77%)
<u>Run 2:</u> (108.6 mg, 0.374 mmol, 75%)
<u>Run 3:</u> (112.2 mg, 0.386 mmol, 77%)
Average: 76% yield

^{1}H NMR (400 MHz, $CDCl_3$) δ 7.45 (d, J= 7.5 Hz, 1H), 7.41 – 7.32 (m, 6H), 7.30 – 7.22 (m, 2H), 6.71 (d, J = 15.9 Hz, 1H), 6.34 (dd, J = 15.8 Hz, 7.1 Hz, 1H), 6.29 (t, J = 2.8 Hz, 1H), 4.66 (q, J = 6.4 Hz, 1H), 3.18 (dd, J = 15.9 Hz, 6.0 Hz, 1H), 2.84 (ddd, J = 16.3 Hz, 9.3 Hz, 3.1 Hz, 1H), 1.58 (s, 3H), 1.50 (s, 3H)

^{13}C NMR (100 MHz, $CDCl_3$) δ 149.1, 137.8, 136.7, 132.0, 129.9, 128.6 (2C), 128.5 (2C), 128.2 (2C), 127.8, 126.7 (3C), 119.7, 83.5, 77.2, 38.5, 29.4, 27.6

HRMS (ESI) m/z: $[M + H]^+$ Calcd for $C_{21}H_{22}O$ 291.1750; Found 291.1728.

(*E*)-4-methylene-2-styryltetrahydrofuran (3fa)

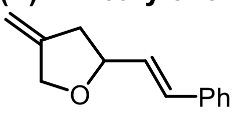

General scope procedure was followed at 80 °C. 2-bromoprop-2-en-1-ol **1f** (68.5 mg, 0.50 mmol, 1.0 equiv.), N-phenyl urea (6.8 mg, 0.050 mmol, 0.1 equiv.), (*E*)-1-phenyl-1,3-butadiene **2a** (84 μL, 0.60 mmol, 1.2 equiv.), K_2CO_3 (83 mg, 0.60 mmol, 1.2 equiv.), and 1 mL of stock solution was used. The crude material was purified as a yellow oil using flash chromatography (silica, 100% hexanes - 95/5 hexanes/EtOAc)

Run 1: (27.9 mg, 0.150 mmol, 30%)
Run 2: (25.2 mg, 0.135 mmol, 27%)
Run 3: (28.6 mg, 0.154 mmol, 31%)
Average: 29% yield

^{1}H NMR (400 MHz, $CDCl_3$) δ 7.43 (d, J = 7.04 Hz, 2H), 7.35 (t, J = 7.16 Hz, 2H), 7.28 (d, J = 6.16 Hz, 1H), 6.67 (d, J = 15.88 Hz, 1H), 6.28 (dd, J = 15.88, 6.72 Hz, 1H), 5.07 (t, J = 2.32 Hz, 1H), 4.99 (t, J = 2.36 Hz, 1H), 4.62 (q, J = 6.76 Hz, 1H), 4.52 (d, J = 13.00 Hz, 1H), 4.37 (d, J = 13.04 Hz, 1H), 2.82 (dd, J = 6.24, 4.68 Hz, 1H), 2.50 (dd, J = 15.6, 8.16 Hz, 1H).

^{13}C NMR (100 MHz, $CDCl_3$) δ 147.9, 136.7, 131.6, 129.3, 128.7 (2C), 127.7, 126.7 (2C), 104.6, 80.3, 71.0, 39.4

HRMS (ESI) m/z: $[M + H]^+$ Calcd for $C_{13}H_{14}O$ 187.1124; Found 187.1119.

(*E*)-2-methyl-3-methylene-5-styryltetrahydrofuran (3ga)

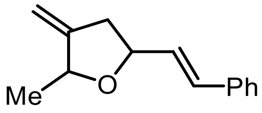

General scope procedure was followed at 80 °C. 3-bromobut-3-en-2-ol **1g** (75.5 mg, 0.50 mmol, 1.0 equiv.), N-phenyl urea (6.8 mg, 0.050 mmol, 0.1 equiv.), (*E*)-1-phenyl-1,3-butadiene **2a** (84 μL, 0.60 mmol, 1.2 equiv.), K_2CO_3 (83 mg, 0.60 mmol, 1.2 equiv.), and 1 mL of stock solution was used. The crude material was purified as a yellow oil using flash chromatography (silica, 100% hexanes - 95/5 hexanes/EtOAc) cis/trans (47:53)

Run 1: (35.2 mg, 0.176 mmol, 35%)
Run 2: (33.2 mg, 0.166 mmol, 33%)
Run 3: (36.8 mg, 0.184 mmol, 37%)
Average: 35% yield

^{1}H NMR (400 MHz, $CDCl_3$) trans δ 7.43 – 7.36 (m, 2H) 7.34 – 7.28 (m, 2H), 7.27 – 7.20 (m, 1H), 6.61(d, J = 12.0 Hz, 1H), 6.30 (m, 1H), 5.07 (d, 2.1 Hz, 1H), 4.78 – 4.65 (m, 1H), 4.56 – 4.44 (m, 1H), 2.94 – 2.80 (m, 1H), 2.63 – 2.48 (m, 1H), 1.39 (d, J = 6.4 Hz, 3H)

^{1}H NMR (400 MHz, $CDCl_3$) cis δ 7.43 – 7.36 (m, 2H) 7.34 – 7.28 (m, 2H), 7.27 – 7.20 (m, 1H), 6.65 (d, J = 11.6 Hz, 1H), 6.30 (m, 1H), 5.04 (d, 2.0 Hz, 1H), 4.95 – 4.89 (m, 2H), 2.94 – 2.80 (m, 1H), 2.63 – 2.48 (m, 1H), 1.34 (d, J = 6.2 Hz, 3H)

^{13}C NMR (100 MHz, $CDCl_3$) trans δ 152.9, 136.8, 132.1, 129.5, 128.6 (2C), 127.8, 126.7 (2C), 104.3, 78.9, 77.6, 39.7, 20.5

^{13}C NMR (100 MHz, $CDCl_3$) cis δ 152.7, 136.8, 131.2, 129.9, 128.6 (2C), 127.7, 126.6 (2C), 104.8, 77.3, 76.2, 39.5, 21.0

HRMS (ESI) m/z: $[M + H]^+$ Calcd for $C_{14}H_{16}O$ 201.1279; Found 201.1276.

5-(3-((*E*)-benzylidene)-5-styryltetrahydrofuran-2-yl)benzo[*d*][1,3]dioxole (3ha)

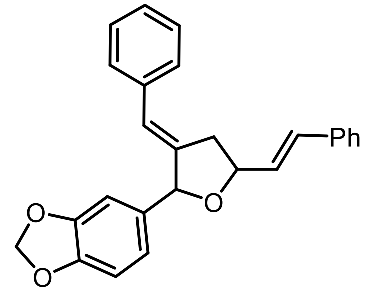

General scope procedure was followed. (*Z*)-1-(benzo[d][1,3]dioxol-5-yl)-2-bromo-3-phenylprop-2-en-1-ol **1h** (166.6 mg, 0.50 mmol, 1.0 equiv.), N-phenyl urea (6.8 mg, 0.050 mmol, 0.1 equiv.), (*E*)-1-phenyl-1,3-butadiene **2a** (84 µL, 0.60 mmol, 1.2 equiv.), K_2CO_3 (83 mg, 0.60 mmol, 1.2 equiv.), and 1 mL of stock solution was used. The crude material was purified as a yellow oil using flash chromatography (silica, 100% hexanes - 95/5 hexanes/EtOAc) cis/trans (49:51)

Run 1: (131.1 mg, 0.343 mmol, 69%)
Run 2: (133.7 mg, 0.350 mmol, 70%)
Run 3: (136.7 mg, 0.357 mmol, 71%)
Average: 70% yield

^{1}H NMR (500 MHz, $CDCl_3$) trans δ 7.48 – 7.24 (m, 10H), 6.98 – 6.95 (m, 2H), 6.88 – 6.84 (m, 1H), 6.78 (d, J = 12.8 Hz, 1H), 6.45 (dd, J = 12.7, 5.6 Hz, 1H), 6.10 (s, 1H), 5.98 (s, 2H), 5.40 (s, 1H), 4.71 – 4.65 (m, 1H), 3.32 – 3.22 (m, 1H), 2.95 – 2.85 (m, 1H)

^{1}H NMR (500 MHz, $CDCl_3$) cis δ 7.48 – 7.24 (m, 10H), 6.98 – 6.95 (m, 2H), 6.88 – 6.84 (m, 1H), 6.70 (d, J = 12.7 Hz, 1H), 6.34 (dd, J = 12.7, 5.2 Hz, 1H), 6.24 (s, 1H), 5.97 (s, 2H), 5.61 (s, 1H), 4.99 (q, J = 5.3 Hz, 1H), 3.32 – 3.22 (m, 1H), 2.95 – 2.85 (m, 1H)

^{13}C NMR (125 MHz, $CDCl_3$) trans δ 148.0, 147.6, 144.6, 137.4, 136.6, 135.9, 132.2, 129.0, 128.7 (2C), 128.5 (2C), 128.4 (2C), 127.9, 127.0, 126.7 (2C), 123.0, 121.8, 108.2, 108.1, 101.1, 85.3, 84.1, 38.9

^{13}C NMR (125 MHz, $CDCl_3$) cis δ 147.9, 147.4, 143.4, 137.3, 136.6, 135.4, 131.5, 129.5, 128.6 (2C), 128.4 (2C), 128.2 (2C), 127.8, 126.9, 126.7 (2C), 123.5, 121.3, 108.1, 107.9, 101.1, 79.9, 79.1, 37.5

HRMS (ESI) m/z: $[M + H]^+$ Calcd for $C_{26}H_{22}O_3$ 383.1648; Found 383.1670.

3-((*E*)-benzylidene)-2-cyclopropyl-5-((*E*)-styryl)tetrahydrofuran (3ia)

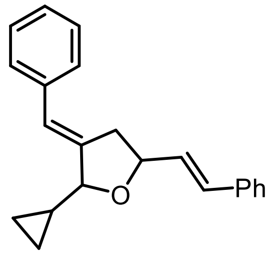

General scope procedure was followed. (*Z*)-2-bromo-1-cyclopropyl-3-phenylprop-2-en-1-ol **1i** (126.6 mg, 0.50 mmol, 1.0 equiv.), N-phenyl urea (6.8 mg, 0.050 mmol, 0.1 equiv.), (*E*)-1-phenyl-1,3-butadiene **2a** (84 µL, 0.60 mmol, 1.2 equiv.), K_2CO_3 (83 mg, 0.60 mmol, 1.2 equiv.), and 1 mL of stock solution was used. The crude material was purified as a yellow oil using flash chromatography (silica, 100% hexanes - 95/5 hexanes/EtOAc) cis/trans (47:53)

Run 1: (73.7 mg, 0.244 mmol, 49%)
Run 2: (68.3 mg, 0.226 mmol, 45%)
Run 3: (71.7 mg, 0.237 mmol, 47%)
Average: 47% yield

^{1}H NMR (500 MHz, $CDCl_3$) trans δ 7.43 -7.22 (m, 10H), 6.69 (d, J = 12.8 Hz, 1H), 6.60 – 6.57 (m, 1H), 6.35 (dd, J = 12.7, 5.8 Hz, 1H), 4.52 – 4.47 (m, 1H), 3.80 (d, J = 6.9 Hz, 1H), 3.11 (dd, J = 13.0, 4.8 Hz, 1H), 2.82 – 2.75 (m, 1H), 1.20 – 1.13 (m, 1H), 0.81 – 0.63 (m, 2H), 0.57 – 0.49 (m, 2H)

^{1}H NMR (500 MHz, $CDCl_3$) cis δ 7.43 -7.22 (m, 10H), 6.63 (d, J = 12.7 Hz, 1H), 6.60 – 6.57 (m, 1H), 6.32 (dd, J = 12.7, 5.5 Hz, 1H), 4.88 – 4.83 (m, 1H), 4.04 (d, J = 6.8 Hz, 1H), 3.20 (dd, J = 13.0, 5.6 Hz, 1H), 2.82 – 2.75 (m, 1H), 1.20 – 1.13 (m, 1H), 0.81 – 0.63 (m, 2H), 0.57 – 0.49 (m, 2H)

^{13}C NMR (125 MHz, $CDCl_3$) trans δ 144.1, 137.6, 136.6, 132.4, 129.4, 128.6 (2C), 128.5 (2C), 128.4 (2C), 127.9, 126.8, 126.7 (2C), 120.9, 87.6, 86.4, 38.9, 15.5, 2.2, 2.2

^{13}C NMR (125 MHz, $CDCl_3$) cis δ 143.6, 137.7, 136.6, 131.4, 129.8, 128.6 (2C), 128.5 (2C), 128.3 (2C), 127.8, 126.8, 126.6 (2C), 121.3, 79.4, 78.7, 37.8, 15.9, 2.8, 2.7

HRMS (ESI) m/z: $[M + H]^+$ Calcd for $C_{22}H_{22}O$ 303.1750; Found 303.1735.

3-((*E*)-benzylidene)-5-((*E*)-styryl)-2-vinyltetrahydrofuran (3ja)

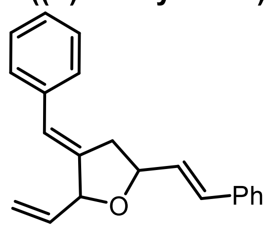

General scope procedure was followed. (*Z*)-2-bromo-1-phenylpenta-1,4-dien-3-ol **1j** (119.6 mg, 0.50 mmol, 1.0 equiv.), N-phenyl urea (6.8 mg, 0.050 mmol, 0.1 equiv.), (*E*)-1-phenyl-1,3-butadiene **2a** (84 µL, 0.60 mmol, 1.2 equiv.), K_2CO_3 (83 mg, 0.60 mmol, 1.2 equiv.), and 1 mL of stock solution was used. The crude material was purified as a yellow oil using flash chromatography (silica, 100% hexanes - 95/5 hexanes/EtOAc) cis/trans (50:50)

Run 1: (24.0 mg, 0.083 mmol, 17%)
Run 2: (24.7 mg, 0.086 mmol, 17%)
Run 3: (23.0 mg, 0.080 mmol, 16%)
Average: 17% yield

^{1}H NMR (400 MHz, $CDCl_3$) trans δ 7.42 – 7.21 (m, 10H), 6.68 (t, J = 16.0 Hz, 1H), 6.36 – 6.24 (m, 2H), 5.97 – 5.87 (m, 1H), 5.47 – 5.27 (m, 2H), 4.89 (d, J = 7.5 Hz, 1H), 4.60 (q, J= 6.2 Hz, 1H), 3.18 – 3.10 (m, 1H), 2.83 – 2.70 (m, 1H)

^{1}H NMR (400 MHz, $CDCl_3$) cis δ 7.42 – 7.21 (m, 10H), 6.68 (t, J = 16.0 Hz, 1H), 6.36 – 6.24 (m, 2H), 5.97 – 5.87 (m, 1H), 5.47 – 5.27 (m, 2H), 5.09 (d, J = 7.0 Hz, 1H), 4.82 (q, J = 6.8 Hz, 1H), 3.18 – 3.10 (m, 1H), 2.83 – 2.70 (m, 1H)

^{13}C NMR (100 MHz, $CDCl_3$) trans δ 142.9, 137.5, 137.4 136.6, 132.4, 129.2, 128.7 (2C), 128.6 (2C), 128.3 (2C), 128.0, 127.0, 126.7 (2C), 122.0, 117.1, 84.8, 83.6, 38.3

^{13}C NMR (100 MHz, $CDCl_3$) cis δ 142.4, 137.5, 137.5, 136.6, 131.6, 129.5, 128.7 (2C), 128.6 (2C), 128.3 (2C), 127.9, 126.9, 126.7 (2C), 122.5, 118.6, 80.0, 79.1, 37.5

HRMS (ESI) m/z: $[M + H]^+$ Calcd for $C_{21}H_{20}O$ 289.1593; Found 289.1580.

(*E*)-2-styryl-2,3,5,6,7,7a-hexahydrobenzofuran (3ka)

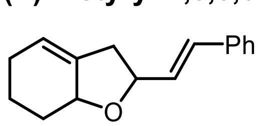

General scope procedure was followed. 2-bromocyclohex-2-en-1-ol **1k** (88.5 mg, 0.50 mmol, 1.0 equiv.), N-phenyl urea (6.8 mg, 0.050 mmol, 0.1 equiv.), (*E*)-1-phenyl-1,3-butadiene **2a** (84 μL, 0.60 mmol, 1.2 equiv.), K_2CO_3 (83 mg, 0.60 mmol, 1.2 equiv.), and 1 mL of stock solution was used. The crude material was purified as a yellow oil using flash chromatography (silica, 100% hexanes - 95/5 hexanes/EtOAc) cis/trans (45:55)

Run 1: (30.6 mg, 0.135 mmol, 27%)
Run 2: (34.5 mg, 0.152 mmol, 30%)
Run 3: (32.2 mg, 0.142 mmol, 28%)
Average: 28% yield

^{1}H NMR (500 MHz, $CDCl_3$) trans δ 7.41 – 7.36 (m, 2H), 7.33 – 7.28 (m, 2H), 7.25 – 7.20 (m, 1H), 6.60 (d, J = 3.4 Hz, 1H), 6.28 (dd, J = 15.8, 7.0 Hz, 1H), 5.52 (s, 1H), 4.36 – 4.29 (m, 1H), 4.16 – 4.10 (m, 1H), 2.69 (dd, J = 14.2, 6.3 Hz, 1H), 2.52 – 2.43 (m, 1H), 2.27 – 2.15 (m, 1H), 2.11 – 2.03 (m, 2H), 1.90 – 1.81 (m, 1H), 1.57 – 1.44 (m, 1H), 1.39 – 1.23 (m, 1H)

^{1}H NMR (500 MHz, $CDCl_3$) cis δ 7.41 – 7.36 (m, 2H), 7.33 – 7.28 (m, 2H), 7.25 – 7.20 (m, 1H), 6.63 (d, J = 3.3 Hz, 1H), 6.20 (dd, J = 15.8, 7.2 Hz, 1H), 5.49 (s, 1H), 4.60 – 4.49 (m, 2H), 2.89 – 2.80 (m, 1H), 2.37 – 2.29 (m, 1H), 2.27 – 2.21 (m, 1H), 2.11 – 2.03 (m, 2H), 1.90 – 1.81 (m, 1H), 1.57 – 1.44 (m, 1H), 1.39 – 1.23 (m, 1H)

^{13}C NMR (125 MHz, DMSO-d6) trans δ 139.8, 136.5, 131.5, 129.7, 128.6 (2C), 127.6, 126.4 (2C), 118.2, 77.3, 74.7, 37.5, 28.8, 24.4, 19.3

^{13}C NMR (125 MHz, DMSO-d6) cis δ 139.4, 136.4, 131.0, 129.9, 128.6 (2C), 127.6, 126.4 (2C), 118.2, 77.0, 76.3, 36.6, 28.4, 24.6, 19.3

HRMS (ESI) m/z: $[M + H]^+$ Calcd for $C_{16}H_{18}O$ 227.1436; Found 227.1428.

(*E*)-2-methyl-3-(3-phenylpropylidene)-5-((*E*)-styryl)tetrahydrofuran (3la)

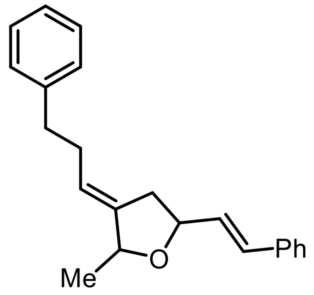

General scope procedure was followed. (*Z*)-3-bromo-6-phenylhex-3-en-2-ol **1l** (127.6 mg, 0.50 mmol, 1.0 equiv.), N-phenyl urea (6.8 mg, 0.050 mmol, 0.1 equiv.), (*E*)-1-phenyl-1,3-butadiene **2a** (84 µL, 0.60 mmol, 1.2 equiv.), K_2CO_3 (83 mg, 0.60 mmol, 1.2 equiv.), and 1 mL of stock solution was used. The crude material was purified as a yellow oil using flash chromatography (silica, 100% hexanes - 95/5 hexanes/EtOAc) cis/trans (47:53)

<u>Run 1:</u> (79.3 mg, 0.260 mmol, 52%)
<u>Run 2:</u> (80.0 mg, 0.263 mmol, 53%)
<u>Run 3:</u> (85.5 mg, 0.281 mmol, 56%)
Average: 54% yield

^{1}H NMR (400 MHz, $CDCl_3$) trans δ 7.44 – 7.20 (m, 10H), 6.63 (t, J = 16.20, 1H), 6.26 (dd, J = 15.8, 7.2 Hz, 1H), 5.31 – 5.23 (m, 1H), 4.45 – 4.38 (m, 2H), 2.74 (t, J = 8.0 Hz, 2H), 2.71 – 2.60 (m, 1H), 2.38 (t, J = 6.8 Hz, 2H), 2.33 – 2.19 (m, 1H), 1.38 (d, J = 6.2 Hz, 3H)

^{1}H NMR (400 MHz, $CDCl_3$) cis δ 7.44 – 7.20 (m, 10H), 6.63 (t, J = 16.2, 1H), 6.21 (dd, J = 15.8, 6.7 Hz, 1H), 5.31 – 5.23 (m, 1H), 4.68 – 4.59 (m, 2H), 2.74 (t, J = 8.0 Hz, 2H), 2.71 – 2.60 (m, 1H), 2.38 (t, J = 6.8 Hz, 2H), 2.33 – 2.19 (m, 1H), 1.33 (d, J = 6.3 Hz, 3H)

^{13}C NMR (100 MHz, $CDCl_3$) trans δ 144.2, 141.9, 136.7, 131.9, 129.6, 128.6 (2C), 128.6 (2C), 128.4 (2C), 127.8, 126.6 (2C), 125.9, 118.7, 78.9, 77.2, 36.4, 35.7, 31.9, 20.4

^{13}C NMR (100 MHz, $CDCl_3$) cis δ 144.0, 141.9, 136.8, 130.9, 130.1, 128.6 (2C), 128.6 (2C), 128.4 (2C), 127.7, 126.6 (2C), 125.9, 119.1, 77.5, 76.3, 35.8, 35.6, 31.9, 21.2

HRMS (ESI) m/z: $[M + H]^+$ Calcd for $C_{22}H_{24}O$ 305.1906; Found 305.1895.

(*E*)-3-(cyclohexylmethylene)-2-methyl-5-((*E*)-styryl)tetrahydrofuran (3ma)

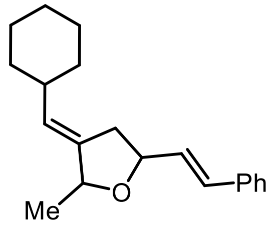

General scope procedure was followed. (*Z*)-3-bromo-4-cyclohexylbut-3-en-2-ol **1m** (116.6 mg, 0.50 mmol, 1.0 equiv.), N-phenyl urea (6.8 mg, 0.050 mmol, 0.1 equiv.), (*E*)-1-phenyl-1,3-butadiene **2a** (84 µL, 0.60 mmol, 1.2 equiv.), K_2CO_3 (83 mg, 0.60 mmol, 1.2 equiv.), and 1 mL of stock solution was used. The crude material was purified as a yellow oil using flash chromatography (silica, 100% hexanes - 95/5 hexanes/EtOAc) cis/trans (47:53)

<u>Run 1:</u> (52.6 mg, 0.186 mmol, 37%)
<u>Run 2:</u> (52.5 mg, 0.186 mmol, 37%)
<u>Run 3:</u> (51.2 mg, 0.181 mmol, 36%)
Average: 37% yield

^{1}H NMR (500 MHz, $CDCl_3$) trans δ 7.43 – 7.38 (m, 2H), 7.35 – 7.30 (m, 2H), 7.27 – 7.22 (m, 1H), 6.65 (d, J = 16.0 Hz, 1H), 6.31 – 6.21 (m, 1H), 5.09 – 5.01 (m, 1H), 4.48 – 4.42 (m, 1H), 4.39 – 4.34 (m, 1H), 2.86 – 2.73 (m, 1H), 2.46 – 2.33 (m, 1H), 2.09 – 2.00 (m, 1H), 1.76 – 1.61 (m, 6H), 1.34 (d, J = 6.2 Hz, 3H) 1.27 – 1.03 (m, 4H)

^1H NMR (500 MHz, $CDCl_3$) cis δ 7.43 – 7.38 (m, 2H), 7.35 – 7.30 (m, 2H), 7.27 – 7.22 (m, 1H), 6.61 (d, J = 16.0 Hz, 1H), 6.31 – 6.21 (m, 1H), 5.09 – 5.01 (m, 1H), 4.70 (q, J = 6.5 Hz, 1H), 4.59 (q, J = 6.5 Hz, 1H), 2.86 – 2.73 (m, 1H), 2.46 – 2.33 (m, 1H), 2.09 – 2.00 (m, 1H), 1.76 – 1.61 (m, 6H), 1.29 (d, J = 6.4 Hz, 3H) 1.27 – 1.03 (m, 4H)

^{13}C NMR (125 MHz, $CDCl_3$) trans δ 141.3, 136.8, 131.8, 129.9, 128.6 (2C), 127.8, 126.7 (2C), 125.9, 79.0, 77.7, 39.2, 36.4, 33.2, 33.0, 26.1 (2C), 26.1, 20.4

^{13}C NMR (125 MHz, $CDCl_3$) cis δ 141.1, 136.9, 130.9, 130.3, 128.6 (2C), 127.7, 126.6 (2C), 126.3, 77.3, 76.3, 39.3, 35.5, 33.1, 33.0, 26.2 (2C), 26.1, 21.2

HRMS (ESI) m/z: $[M + H]^+$ Calcd for $C_{20}H_{26}O$ 283.2063; Found 283.2056.

3-((*E*)-4-methoxybenzylidene)-2-methyl-5-((*E*)-styryl)tetrahydrofuran (3na)

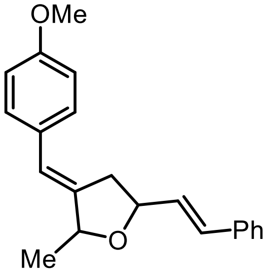

General scope procedure was followed. (*Z*)-3-bromo-4-(p-tolyl)but-3-en-2-ol **1n** (128.9 mg, 0.50 mmol, 1.0 equiv.), N-phenyl urea (6.8 mg, 0.050 mmol, 0.1 equiv.), (*E*)-1-phenyl-1,3-butadiene **2a** (84 μL, 0.60 mmol, 1.2 equiv.), K_2CO_3 (83 mg, 0.60 mmol, 1.2 equiv.), and 1 mL of stock solution was used. The crude material was purified as a yellow oil using flash chromatography (silica, 100% hexanes - 95/5 hexanes/EtOAc) cis/trans (47:53)

Run 1: (112.1 mg, 0.366 mmol, 73%)
Run 2: (110.1 mg, 0.359 mmol, 72%)
Run 3: (111.8 mg, 0.365 mmol, 73%)
Average: 73% yield

^1H NMR (400 MHz, $CDCl_3$) trans δ 7.48 – 7.23 (m, 7H), 6.94 (d, J = 8.8 Hz, 2H), 6.73 (d, J = 16.0 Hz, 1H), 6.41 – 6.21 (m, 2H), 4.67 – 4.59 (m, 2H), 4.59 – 4.51 (m, 2H), 3.84 (s, 3H), 3.21 – 3.04 (m, 1H), 2.83 – 2.68 (m, 1H), 1.54 (d, J = 6.2 Hz, 3H)

^1H NMR (400 MHz, $CDCl_3$) cis δ 7.48 – 7.23 (m, 7H), 6.94 (d, J = 8.8 Hz, 2H), 6.68 (d, J = 16.0 Hz, 1H), 6.41 – 6.21 (m, 2H), 4.90 – 4.80 (m, 2H), 3.84 (s, 3H), 3.21 – 3.04 (m, 1H), 2.83 – 2.68 (m 1H), 1.48 (d, J = 6.3 Hz, 3H)

^{13}C NMR (100 MHz, $CDCl_3$) trans δ 158.4, 143.3, 136.7, 132.2, 130.5, 129.4, 129.4 (2C), 129.3 (2C) 128.6 (2C), 127.8, 126.7 (2C), 119.4, 113.9, 79.5, 78.7, 55.3, 38.5, 20.7

^{13}C NMR (100 MHz, $CDCl_3$) cis δ 158.4, 143.1, 136.6, 131.2, 130.4, 129.9, 129.4 (2C), 129.3 (2C), 128.6 (2C), 127.7. 126.6 (2C), 119.8, 113.9, 78.2, 77.9, 55.3, 37.4, 21.2

HRMS (ESI) m/z: $[M + H]^+$ Calcd for $C_{21}H_{22}O_2$ 307.1699; Found 307.1685.

2-methyl-5-((*E*)-styryl)-3-((*E*)-4-(trifluoromethyl)benzylidene)tetrahydrofuran (3oa)

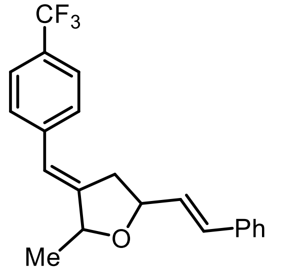

General scope procedure was followed. (*Z*)-3-bromo-4-(4-(trifluoromethyl)phenyl)but-3-en-2-ol **1o** (147.6 mg, 0.50 mmol, 1.0 equiv.), N-phenyl urea (6.8 mg, 0.050 mmol, 0.1 equiv.), (*E*)-1-phenyl-1,3-butadiene **2a** (84 µL, 0.60 mmol, 1.2 equiv.), K_2CO_3 (83 mg, 0.60 mmol, 1.2 equiv.), and 1 mL of stock solution was used. The crude material was purified as a yellow oil using flash chromatography (silica, 100% hexanes - 95/5 hexanes/EtOAc) cis/trans (47:53)

Run 1: (68.4 mg, 0.292 mmol, 58%)
Run 2: (68.3 mg, 0.292 mmol, 58%)
Run 3: (71.7 mg, 0.286 mmol, 57%)
Average: 58% yield

^{1}H NMR (400 MHz, $CDCl_3$) trans δ 7.62 (d, J = 8.0 Hz, 2H), 7.46 – 7.37 (m, 4H), 7.37 – 7.30 (m, 2H), 7.30 – 7.23 (m, 1H), 6.72 (d, J = 16.0 Hz, 1H), 6.39 – 6.22 (m, 2H), 4.68 – 4.59 (m, 1H), 4.59 – 4.50 (m, 1H), 3.21 – 3.03 (m, 1H), 2.84 – 2.69 (m, 1H), 1.53 (d, J = 6.2 Hz, 3H)

^{1}H NMR (400 MHz, $CDCl_3$) cis δ 7.62 (d, J = 8.0 Hz, 2H), 7.46 – 7.37 (m, 4H), 7.37 – 7.30 (m, 2H), 7.30 – 7.23 (m, 1H), 6.67 (d, J = 16.0 Hz, 1H), 6.39 – 6.22 (m, 2H), 4.92 – 4.79 (m, 2H), 3.21 – 3.03 (m, 1H), 2.84 – 2.69 (m, 1H), 1.48 (d, J = 6.3 Hz, 3H)

^{13}C NMR (100 MHz, $CDCl_3$) trans δ 148.7, 141.2, 136.6, 132.5, 129.0, 128.7 (3C), 128.3 (2C), 128.0, 127.1, 126.7 (2C), 125.4 (2C), 119.0, 79.5, 78.7, 38.7, 20.5

^{13}C NMR (100 MHz, $CDCl_3$) cis δ 148.4, 141.2, 136.5, 131.5, 129.4, 128.7 (3C), 128.4 (2C), 127.9, 127.1, 126.7 (2C), 125.4 (2C), 119.4, 78.2, 78.0, 37.7, 21.1

^{19}F NMR (100 MHz, $CDCl_3$) trans δ -62.2

^{19}F NMR (100 MHz, $CDCl_3$) cis δ -62.2

HRMS (ESI) m/z: $[M + H]^+$ Calcd for $C_{21}H_{19}F_3O$ 345.1467; Found 345.1460.

5-((*E*)-(2-methyl-5-((*E*)-styryl)dihydrofuran-3(2H)-ylidene)methyl)-2,3-dihydrobenzofuran (3pa)

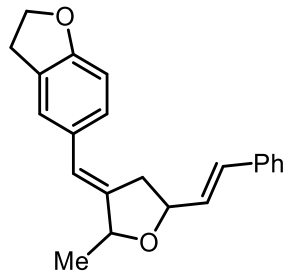

General scope procedure was followed. (*Z*)-3-bromo-4-(2,3-dihydrobenzofuran-5-yl)but-3-en-2-ol **1p** (134.6 mg, 0.50 mmol, 1.0 equiv.), N-phenyl urea (6.8 mg, 0.050 mmol, 0.1 equiv.), (*E*)-1-phenyl-1,3-butadiene **2a** (84 µL, 0.60 mmol, 1.2 equiv.), K_2CO_3 (83 mg, 0.60 mmol, 1.2 equiv.), and 1 mL of stock solution was used. The crude material was purified as a yellow oil using flash chromatography (silica, 100% hexanes - 95/5 hexanes/EtOAc) cis/trans (47:53)

Run 1: (100.6 mg, 0.316 mmol, 63%)
Run 2: (95.9 mg, 0.301 mmol, 60%)
Run 3: (103.0 mg, 0.323 mmol, 65%)
Average: 63% yield

^{1}H NMR (400 MHz, $CDCl_3$) trans δ 7.48 – 7.40 (m, 2H), 7.40 – 7.32 (m, 2H), 7.32 – 7.26 (m, 1H), 7.25 – 7.21 (m, 1H), 7.15 – 7.10 (m, 1H), 6.83 (d, J = 8.5 Hz, 1H), 6.73 (d, J = 16.0 Hz, 1H), 6.41 – 6.21 (m, 2H), 4.61 (t, J = 8.5 Hz, 2H), 4.66 – 4.52 (m, 2H), 3.24 (t, J = 8.5 Hz, 2H), 3.20 – 3.05 (m, 1H), 2.83 – 2.70 (m, 1H), 1.53 (d, J = 6.2 Hz, 3H)

^{1}H NMR (400 MHz, $CDCl_3$) cis δ 7.48 – 7.40 (m, 2H), 7.40 – 7.32 (m, 2H), 7.32 – 7.26 (m, 1H), 7.25 – 7.21 (m, 1H), 7.15 – 7.10 (m, 1H), 6.83 (d, J = 8.5 Hz, 1H), 6.68 (d, J = 16.0 Hz, 1H), 6.41 – 6.21 (m, 2H), 4.89 – 4.80 (m, 2H), 4.61 (t, J = 8.5 Hz, 2H), 3.24 (t, J = 8.5 Hz, 2H), 3.20 – 3.05 (m, 1H), 2.83 – 2.70 (m, 1H), 1.48 (d, J = 6.3 Hz, 3H)

^{13}C NMR (100 MHz, $CDCl_3$) trans δ 159.0, 142.6, 136.6, 132.2, 130.5, 129.5, 128.6 (2C), 128.4, 127.9, 127.3, 126.7 (2C), 124.6, 119.8, 109.2, 79.5, 78.7, 71.5, 38.6, 29.8, 20.8

^{13}C NMR (100 MHz, $CDCl_3$) cis δ 159.0, 142.4, 136.7, 131.2, 130.5, 129.9, 128.6 (2C), 128.5, 127.7, 127.3, 126.6 (2C), 124.7, 120.2, 109.2, 78.2, 77.9, 71.5, 37.6, 29.8, 21.3

HRMS (ESI) m/z: $[M + H]^+$ Calcd for $C_{22}H_{22}O_2$ 319.1699; Found 319.1695.

3-((1*E*)-(2-methyl-5-styryldihydrofuran-3(2H)-ylidene)methyl)pyridine (3qa)

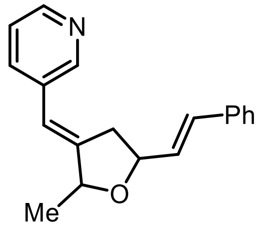

General scope procedure was followed. (*Z*)-3-bromo-4-(pyridin-3-yl)but-3-en-2-ol **1q** (114.0 mg, 0.50 mmol, 1.0 equiv.), N-phenyl urea (6.8 mg, 0.050 mmol, 0.1 equiv.), (*E*)-1-phenyl-1,3-butadiene **2a** (84 µL, 0.60 mmol, 1.2 equiv.), K_2CO_3 (83 mg, 0.60 mmol, 1.2 equiv.), and 1 mL of stock solution was used. The crude material was purified as a yellow oil fusing flash chromatography (silica, 100% hexanes - 95/5 hexanes/EtOAc) cis/trans (43:57)

Run 1: (58.4 mg, 0.211 mmol, 42%)
Run 2: (53.9 mg, 0.194 mmol, 39%)
Run 3: (56.6 mg, 0.204 mmol, 41%)
Average: 41% yield

^{1}H NMR (500 MHz, $CDCl_3$) trans δ 8.59 (s, 1H), 8.45 (s, 1H), 7.64 (t, J = 7.0 Hz, 1H), 7.45 – 7.37 (m, 2H), 7.37 – 7.22 (m, 4H), 6.71 (d, J = 16.0 Hz, 1H), 6.37 – 6.21 (m, 2H), 4.67 – 4.60 (m, 1H), 4.59 – 4.51 (m, 1H), 3.19 – 3.03 (m, 1H), 2.83 – 2.68 (m, 1H), 1.53 (d, J = 6.2 Hz, 3H)

^{1}H NMR (500 MHz, $CDCl_3$) cis δ 8.59 (s, 1H), 8.45 (s, 1H), 7.64 (t, J = 7.0 Hz, 1H), 7.45 – 7.37 (m, 2H), 7.37 – 7.22 (m, 4H), 6.66 (d, J = 16.0 Hz, 1H), 6.37 – 6.21 (m, 2H), 4.91 – 4.81 (m, 2H), 3.19 – 3.03 (m, 1H), 2.83 – 2.68 (m, 1H), 1.47 (d, J = 6.3 Hz, 3H)

^{13}C NMR (125 MHz, $CDCl_3$) trans δ 149.7, 148.5, 147.6, 136.4, 134.6, 133.3, 132.6, 128.8, 128.6, 128.0, 126.7 (2C), 123.3, 116.6, 79.4, 78.6, 38.6, 20.5

^{13}C NMR (125 MHz, $CDCl_3$) cis δ 149.7, 148.2, 147.6, 136.5, 134.7, 133.3, 131.5, 129.3, 128.6, 127.8, 126.6 (2C), 123.3, 117.0, 78.1, 77.8, 37.5, 21.1

HRMS (ESI) m/z: $[M + H]^+$ Calcd for $C_{19}H_{19}NO$ 278.1546; Found 278.1544.

1,3-Diene Scope

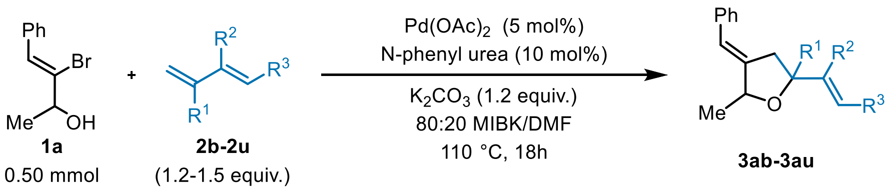

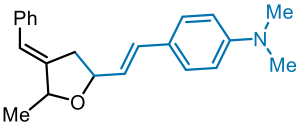

66%
3ab

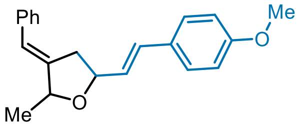

58%
3ac

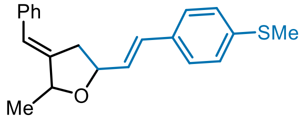

22%
3ad

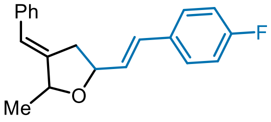

72%
3ae

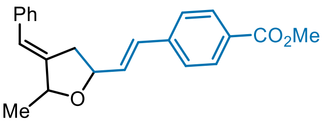

65%
3af

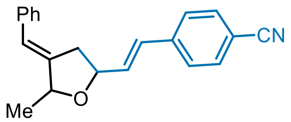

65%
3ag

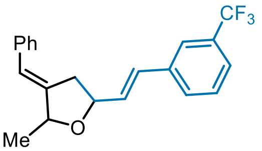

72%
3ah

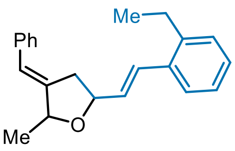

66%
3ai

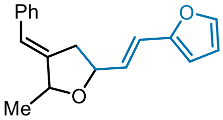

52%
3aj

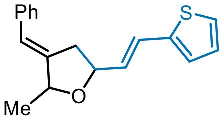

54%
3ak

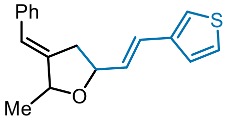

42%
3al

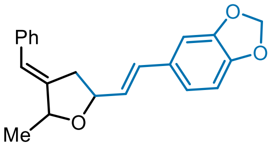

59%
3am

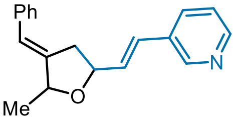

54%
3an

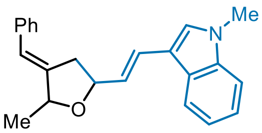

57%
3ao

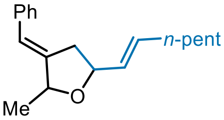

62%
3ap

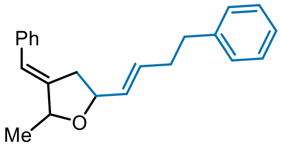

53%
3aq

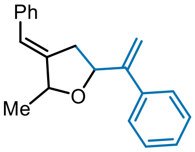

64%
3ar

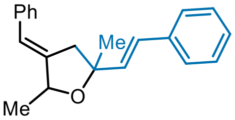

76%
3as

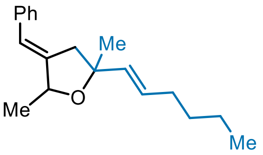

48%
3at

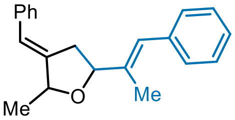

48%
3au

General Procedure for Diene Scope (General Scope Procedure):
Bromoallyl alcohol **1a** (113.6 mg, 0.50 mmol, 1.0 equiv.), N-phenyl urea (6.8 mg, 0.050 mmol, 0.1 equiv.), and diene **2b-2u** (0.60-0.75 mmol, 1.2-1.5 equiv.) were weighed/measured out in the above-mentioned order into a flame-dried 1-dram vial equipped with a small football-shaped stir bar and a cap with a silicone septum. 1.0 mL stock solution was prepared (in a flame-dried 1-dram vial equipped with a small football-shaped stir bar and a cap with a silicone septum) with solvent (0.2 mL DMF, 0.8 mL MIBK) and palladium acetate (5.6 mg, 0.025 mmol, 0.05 equiv.) and left to stir for 30 min. Then, the stock solution was added to the first vial and left to stir for several minutes at room temperature. Potassium carbonate base (83 mg, 0.60 mmol, 1.2 equiv.) was added to the reaction mixture and a fresh septum was placed on the vial. The reaction mixture was then stirred at 110 °C in an aluminum block for 18 h at 650 rpm. After cooling to rt, the crude material was filtered through a plug of celite using ethyl acetate. The crude material was then analyzed via NMR to determine the d.r. The crude material was purified by flash column chromatography to determine isolated yield.

4-((*E*)-2-(4-((*E*)-benzylidene)-5-methyltetrahydrofuran-2-yl)vinyl)-N,N-dimethylaniline (3ab)

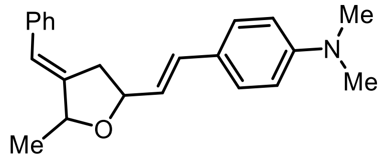

General scope procedure was followed. (*Z*)-3-bromo-4-phenylbut-3-en-2-ol **1a** (113.6 mg, 0.50 mmol, 1.0 equiv.), N-phenyl urea (6.8 mg, 0.050 mmol, 0.1 equiv.), (*E*)-4-(buta-1,3-dien-1-yl)-N,N-dimethylaniline **2b** (104 mg, 0.60 mmol, 1.2 equiv.), K_2CO_3 (83 mg, 0.60 mmol, 1.2 equiv.), and 1 mL of stock solution was used. The crude material was purified as a yellow oil using flash chromatography (silica, 100% hexanes - 95/5 hexanes/EtOAc) cis/trans (45:55)

<u>Run 1:</u> (108.9 mg, 0.341 mmol, 68%)
<u>Run 2:</u> (101.2 mg, 0.317 mmol, 64%)
<u>Run 3:</u> (104.8 mg, 0.327 mmol, 65%)
Average: 66% yield

^{1}H NMR (400 MHz, $CDCl_3$) trans δ 7.37 – 7.20 (m, 7H), 6.70 – 6.63 (m, 2H), 6.60 (d, J = 16.0 Hz, 1H), 6.16 – 6.01 (m, 1H), 4.63 – 4.53 (m, 1H), 4.53 – 4.43 (m, 1H), 3.18 – 3.02 (m, 1H), 2.96 (s, 6H), 2.82 – 2.66 (m, 1H), 1.49 (d, J = 6.2 Hz, 3H)

^{1}H NMR (400 MHz, $CDCl_3$) cis δ 7.37 – 7.20 (m, 7H), 6.70 – 6.63 (m, 2H), 6.54 (d, J = 16.0 Hz, 1H), 6.16 – 6.01 (m, 1H), 4.87 – 4.71 (m, 2H), 3.18 – 3.02 (m, 1H), 2.95 (s, 6H), 2.82 – 2.66 (m, 1H), 1.43 (d, J = 6.4 Hz, 3H)

^{13}C NMR (100 MHz, $CDCl_3$) trans δ 150.3, 146.2 137.8, 132.9, 128.5 (2C), 128.2 (2C), 127.8 (2C), 126.7, 125.1, 124.7, 119.9, 112.4 (2C), 80.2, 78.8, 40.6 (2C), 38.9, 20.7

^{13}C NMR (100 MHz, $CDCl_3$) cis δ 150.3, 146.0, 137.8, 131.8, 128.5 (2C), 128.3 (2C), 127.7 (2C), 126.7, 125.2, 125.0, 120.2, 112.4 (2C), 78.6, 77.8, 40.6 (2C), 37.9, 21.2

HRMS (ESI) m/z: $[M + H]^+$ Calcd for $C_{23}H_{28}NO$ 320.2015; Found 320.2005.

3-((*E*)-benzylidene)-5-((*E*)-4-methoxystyryl)-2-methyltetrahydrofuran (3ac)

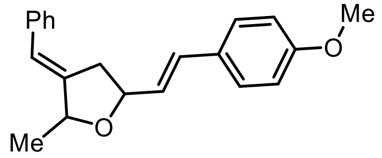

General scope procedure was followed. (*Z*)-3-bromo-4-phenylbut-3-en-2-ol **1a** (113.6 mg, 0.50 mmol, 1.0 equiv.), N-phenyl urea (6.8 mg, 0.050 mmol, 0.1 equiv.), (*E*)-1-(buta-1,3-dien-1-yl)-4-methoxybenzene **2c** (96 mg, 0.60 mmol, 1.2 equiv.), K_2CO_3 (83 mg, 0.60 mmol, 1.2 equiv.), and 1 mL of stock solution was used. The crude material was purified as a yellow oil using flash chromatography (silica, 100% hexanes - 95/5 hexanes/EtOAc) cis/tans (47:53)

<u>Run 1:</u> (90.1 mg, 0.294 mmol, 59%)
<u>Run 2:</u> (92.5 mg, 0.302 mmol, 60%)
<u>Run 3:</u> (85.8 mg, 0.280 mmol, 56%)
Average: 58% yield

^{1}H NMR (400 MHz, $CDCl_3$) trans δ 7.38 – 7.27 (m, 7H), 7.25 – 7.16 (m, 1H), 6.89 – 6.81 (m, 2H), 6.63 (d, J = 16.0 Hz, 1H), 6.25 (s, 1H), 6.23 – 6.08 (m, 1H), 4.64 – 4.55 (m, 1H), 4.49 (q, J = 7.2 Hz, 1H), 3.80 (s, 3H), 3.19 – 3.02 (m, 1H), 2.82 – 2.66 (m, 1H), 1.50 (d, J = 6.2 Hz, 3H)

^{1}H NMR (400 MHz, $CDCl_3$) cis δ 7.38 – 7.27 (m, 7H), 7.25 – 7.16 (m, 1H), 6.89 – 6.81 (m, 2H), 6.58 (d, J = 16.0 Hz, 1H), 6.29 (s, 1H), 6.23 – 6.08 (m, 1H), 4.87 – 4.73 (m, 2H), 3.79 (s, 3H), 3.19 – 3.02 (m, H), 2.82 – 2.66 (m, 1H), 1.44 (d, J = 6.3 Hz, 3H)

^{13}C NMR (100 MHz, $CDCl_3$) trans δ 159.4, 145.8, 137.7, 132.0, 129.4, 128.5 (2C), 128.2 (2C), 127.9 (2C), 127.0, 126.7 (2C), 120.0, 114.0, 79.8, 78.6, 55.3, 38.7, 20.7

^{13}C NMR (100 MHz, $CDCl_3$) cis δ 159.4, 145.6, 137.7, 131.0, 129.4, 128.5 (2C), 128.2 (2C), 127.8 (2C), 127.4, 126.7 (2C), 120.3, 114.0, 78.4, 77.8, 55.3, 37.7, 21.2

HRMS (ESI) m/z: $[M + H]^+$ Calcd for $C_{22}H_{25}O_2$ 307.1699; Found 307.1690.

3-((*E*)-benzylidene)-2-methyl-5-((*E*)-4-(methylthio)styryl)tetrahydrofuran (3ad)

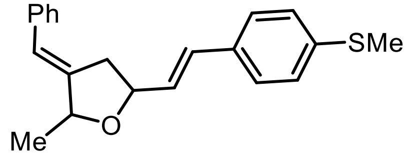

General scope procedure was followed. (*Z*)-3-bromo-4-phenylbut-3-en-2-ol **1a** (113.6 mg, 0.50 mmol, 1.0 equiv.), N-phenyl urea (6.8 mg, 0.050 mmol, 0.1 equiv.), (*E*)-(4-(buta-1,3-dien-1-yl)phenyl)(methyl)sulfane **2d** (106 mg, 0.60 mmol, 1.2 equiv.), K_2CO_3 (83 mg, 0.60 mmol, 1.2 equiv.), and 1 mL of stock solution was used. The crude material was purified as a yellow oil using flash chromatography (silica, 100% hexanes - 95/5 hexanes/EtOAc) cis/trans (47:53)

<u>Run 1:</u> (37.4 mg, 0.116 mmol, 23%)
<u>Run 2:</u> (31.9 mg, 0.099 mmol, 20%)
<u>Run 3:</u> (34.1 mg, 0.106 mmol, 21%)
Average: 22% yield

^{1}H NMR (400 MHz, $CDCl_3$) trans δ 7.39 – 7.15 (m, 9H), 6.62 (d, J = 16.0 Hz, 1H), 6.31 – 6.16 (m, 2H), 4.64 – 4.55 (m, 1H), 4.54 – 4.44 (m, 1H), 3.18 – 3.03 (m, 1H), 2.80 – 2.66 (m, 1H), 2.47 (s, 3H), 1.48 (d, J = 6.3 Hz, 3H)

^{1}H NMR (400 MHz, $CDCl_3$) cis δ 7.39 – 7.15 (m, 9H), 6.57 (d, J = 16.0 Hz, 1H), 6.31 – 6.16 (m, 2H), 4.86 – 4.75 (m, 2H), 3.18 – 3.03 (m, 1H), 2.80 – 2.66 (m, 1H), 2.46 (s, 3H), 1.43 (d, J = 6.3 Hz, 3H)

^{13}C NMR (100 MHz, $CDCl_3$) trans δ 145.7, 138.1, 137.7, 133.6, 131.8, 128.7, 128.5 (2C), 128.2 (2C), 127.1 (2C), 126.8, 126.6 (2C), 120.0, 79.6, 78.7, 38.7, 20.7, 15.8

^{13}C NMR (100 MHz, $CDCl_3$) cis δ 145.4, 138.0, 137.7, 133.7, 130.8, 129.1, 128.5 (2C), 128.3 (2C), 127.1 (2C), 126.8, 126.6 (2C), 120.5, 78.3, 78.0, 37.6, 21.3, 15.9

HRMS (ESI) m/z: $[M + H]^+$ Calcd for $C_{22}H_{25}OS$ 323.1470; Found 323.1460.

3-((*E*)-benzylidene)-5-((*E*)-4-fluorostyryl)-2-methyltetrahydrofuran (3ae)

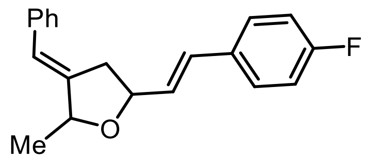

General scope procedure was followed. (*Z*)-3-bromo-4-phenylbut-3-en-2-ol **1a** (113.6 mg, 0.50 mmol, 1.0 equiv.), N-phenyl urea (6.8 mg, 0.050 mmol, 0.1 equiv.), (*E*)-1-(buta-1,3-dien-1-yl)-4-fluorobenzene **2e** (89.0 mg, 0.60 mmol, 1.2 equiv.), K_2CO_3 (83 mg, 0.60 mmol, 1.2 equiv.), and 1 mL of stock solution was used. The crude material was purified as a yellow oil using flash chromatography (silica, 100% hexanes - 95/5 hexanes/EtOAc) cis/trans (48:52)

Run 1: (107.9 mg, 0.368 mmol, 74%)
Run 2: (103.3 mg, 0.351 mmol, 70%)
Run 3: (107.5 mg, 0.365 mmol, 73%)
Average: 72% yield

^{1}H NMR (400 MHz, $CDCl_3$) trans δ 7.40 – 7.31 (m, 6H), 7.27 – 7.21 (m, 1H), 7.05 – 6.96 (m, 2H), 6.66 (d, J = 16.0 Hz, 1H), 6.33 – 6.14 (m, 2H), 4.65 – 4.57 (m, 1H), 4.54 – 4.46 (m, 1H), 3.20 – 3.04 (m, 1H), 2.82 – 2.67 (m, 1H), 1.51 (d, J = 6.0 Hz, 3H)

^{1}H NMR (400 MHz, $CDCl_3$) cis δ 7.40 – 7.31 (m, 6H), 7.27 – 7.21 (m, 1H), 7.05 – 6.96 (m, 2H), 6.60 (d, J = 16.0 Hz, 1H), 6.33 – 6.14 (m, 2H), 4.88 – 4.75 (m, 2H), 3.20 – 3.04 (m, 1H), 2.82 – 2.67 (m, 1H), 1.45 (d, J = 6.4 Hz, 3H)

^{13}C NMR (100 MHz, $CDCl_3$) trans δ 163.7, 145.5, 137.7, 132.8, 131.1, 129.6, 129.5, 128.5, (2C) 128.2 (2C), 128.2 (2C), 126.7, 120.1, 115.6, 79.4, 78.7, 38.6, 20.8

^{13}C NMR (100 MHz, $CDCl_3$) cis δ 161.2, 145.3, 137.6, 132.8, 130.1, 129.5, 129.1, 128.5, (2C) 128.2 (2C), 128.2 (2C), 128.1, 120.5, 115.4, 78.1, 77.9, 37.5, 21.2

^{19}F NMR (375 MHz, $CDCl_3$) trans δ -114.0

^{19}F NMR (375 MHz, $CDCl_3$) cis δ -114.2

HRMS (ESI) m/z: $[M + H]^+$ Calcd for $C_{21}H_{22}FO$ 295.1499; Found 295.1491.

Methyl 4-((*E*)-2-(4-((*E*)-benzylidene)-5-methyltetrahydrofuran-2-yl)vinyl)benzoate (3af)

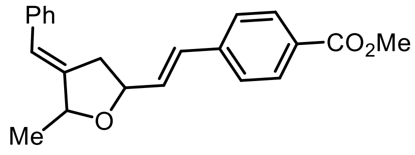

General scope procedure was followed. (*Z*)-3-bromo-4-phenylbut-3-en-2-ol **1a** (113.6 mg, 0.50 mmol, 1.0 equiv.), N-phenyl urea (6.8 mg, 0.050 mmol, 0.1 equiv.), methyl (*E*)-4-(buta-1,3-dien-1-yl)benzoate **2f** (113 mg, 0.60 mmol, 1.2 equiv.), K_2CO_3 (83 mg, 0.60 mmol, 1.2 equiv.), and 1 mL of stock solution was used. The crude material was purified as a yellow oil using flash chromatography (silica, 100% hexanes - 95/5 hexanes/EtOAc) cis/trans (49:51)

<u>Run 1:</u> (106.7 mg, 0.319 mmol, 64%)
<u>Run 2:</u> (109.3 mg, 0.328 mmol, 66%)
<u>Run 3:</u> (111.2 mg, 0.332 mmol, 66%)
Average: 65% yield

^{1}H NMR (400 MHz, $CDCl_3$) trans δ 7.95 – 7.81 (m, 2H), 7.38 (d, J = 8.4 Hz, 2H), 7.31 – 7.22 (m, 3H), 7.20 – 7.11 (m, 2H), 6.64 (d, J = 16.0 Hz, 1H), 6.36 (dd, J = 16.0, 7.0 Hz, 1H), 6.25 – 6.21 (m, 1H), 4.58 – 4.50 (m, 1H), 4.50 – 4.42 (m, 1H), 3.38 (s, 3H), 3.15 – 3.98 (m, 1H), 2.78 – 2.60 (m, 1H), 1.42 (d, J = 6.3 Hz, 3H)

^{1}H NMR (400 MHz, $CDCl_3$) cis δ 7.95 – 7.81 (m, 2H), 7.35 (d, J = 8.4 Hz, 2H), 7.31 – 7.22 (m, 3H), 7.20 – 7.11 (m, 2H), 6.59 (d, J = 16.0 Hz, 1H), 6.29 (dd, J = 16.0 Hz, 6.3 Hz, 1H), 6.21 – 6.17 (m, 1H), 4.80 – 4.71 (m, 2H), 3.38 (s, 3H), 3.15 – 3.98 (m, 1H), 2.78 – 2.60 (m, 1H), 1.37 (d, J = 6.4 Hz, 3H)

^{13}C NMR (100 MHz, $CDCl_3$) trans δ 167.0, 145.3, 141.2, 137.6, 132.6, 131.1, 130.0 (2C), 128.5 (2C), 128.2 (2C), 126.8, 126.6 (2C), 126.0, 120.3, 79.2, 78.8, 52.2, 38.5, 20.7

^{13}C NMR (100 MHz, $CDCl_3$) cis δ 167.0, 145.0, 141.2, 137.6, 132.1, 130.1, 130.0 (2C), 128.5 (2C), 128.3 (2C), 126.8, 126.5 (2C), 126.0, 120.7, 78.1, 78.0, 52.2, 37.4, 21.3

HRMS (ESI) m/z: $[M + H]^+$ Calcd for $C_{23}H_{25}O_3$ 335.1648; Found 335.1639.

4-((*E*)-2-(4-((*E*)-benzylidene)-5-methyltetrahydrofuran-2-yl)vinyl)benzonitrile (3ag)

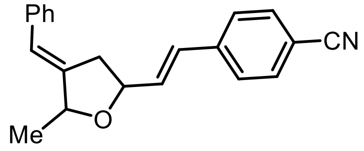

General scope procedure was followed. (*Z*)-3-bromo-4-phenylbut-3-en-2-ol **1a** (113.6 mg, 0.50 mmol, 1.0 equiv.), N-phenyl urea (6.8 mg, 0.050 mmol, 0.1 equiv.), (*E*)-4-(buta-1,3-dien-1-yl)benzonitrile **2g** (93.1 mg, 0.60 mmol, 1.2 equiv.), K_2CO_3 (83 mg, 0.60 mmol, 1.2 equiv.), and 1 mL of stock solution was used. The crude material was purified as a yellow oil using flash chromatography (silica, 100% hexanes - 95/5 hexanes/EtOAc) cis/trans (48:52)

<u>Run 1:</u> (100.8 mg, 0.334 mmol, 67%)
<u>Run 2:</u> (97.1 mg , 0.322 mmol, 64%)
<u>Run 3:</u> (96.7 mg, 0.321 mmol, 64%)
Average: 65% yield

^{1}H NMR (400 MHz, $CDCl_3$) trans δ 7.61 – 7.55 (m, 2H), 7.47 – 7.41 (m, 2H), 7.37 – 7.27 (m, 4H), 7.24 – 7.19 (m, 1H), 6.68 (d, J = 16.0 Hz, 1H), 6.44 (dd, J = 16.0, 6.8 Hz, 1H), 6.28 – 6.23 (m, 1H), 4.65 – 4.57 (m, 1H), 4.57 – 4.49 (m, 1H), 3.22 – 3.05 (m, 1H), 2.81 – 2.65 (m, 1H), 1.49 (d, J = 6.2 Hz, 3H)

^{1}H NMR (400 MHz, $CDCl_3$) cis δ 7.61 – 7.55 (m, 2H), 7.47 – 7.41 (m, 2H), 7.37 – 7.27 (m, 4H), 7.24 – 7.19 (m, 1H), 6.63 (d, J = 16.0 Hz, 1H), 6.37 (dd, J = 16.0, 6.2 Hz, 1H), 6.31 – 6.28 (m, 1H), 4.85 – 4.78 (m, 2H), 3.22 – 3.05 (m, 1H), 2.81 – 2.65 (m, 1H), 1.43 (d, J = 6.4 Hz, 3H)

^{13}C NMR (100 MHz, $CDCl_3$) trans δ 145.0, 141.3, 137.6, 133.5, 132.6 (2C), 130.1, 128.6 (2C), 128.2 (2C), 127.2 (2C), 126.9, 120.4, 119.1, 111.1, 78.9, 78.9, 38.4, 20.7

^{13}C NMR (100 MHz, $CDCl_3$) cis δ 144.7, 141.2, 137.5, 133.9, 132.5 (2C), 129.3, 128.6 (2C), 128.3 (2C), 127.1 (2C), 126.9, 120.8, 119.1, 111.1, 78.2, 77.8, 37.4, 21.3

HRMS (ESI) m/z: $[M + H]^+$ Calcd for $C_{22}H_{22}NO$ 302.1546; Found 302.1537.

3-((*E*)-benzylidene)-2-methyl-5-((*E*)-3-(trifluoromethyl)styryl)tetrahydrofuran (3ah)

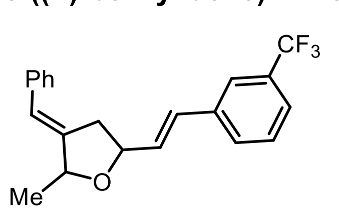

General scope procedure was followed. (*Z*)-3-bromo-4-phenylbut-3-en-2-ol **1a** (113.6 mg, 0.50 mmol, 1.0 equiv.), N-phenyl urea (6.8 mg, 0.050 mmol, 0.1 equiv.), (*E*)-1-(buta-1,3-dien-1-yl)-3-(trifluoromethyl)benzene **2i** (119 µL, 0.60 mmol, 1.2 equiv.), K_2CO_3 (83 mg, 0.60 mmol, 1.2 equiv.), and 1 mL of stock solution was used. The crude material was purified as a yellow oil using flash chromatography (silica, 100% hexanes - 95/5 hexanes/EtOAc) cis/trans (50:50)

Run 1: (128.1 mg, 0.372 mmol, 74%)
Run 2: (122.3 mg, 0.355 mmol, 71%)
Run 3: (123.8 mg, 0.359 mmol, 72%)
Average: 72% yield

^{1}H NMR (400 MHz, $CDCl_3$) trans δ 7.63 (d, J = 14.8 Hz, 1H), 7.59 – 7.29 (m, 7H), 7.26 – 7.20 (m, 1H), 6.72 (d, J = 16.0 Hz, 1H), 6.40 (dd, J = 16.0, 6.9 Hz, 1H), 6.28 – 6.24 (m, 1H), 4.66 – 4.58 (m, 1H), 4.58 – 4.50 (m, 1H), 3.22 – 3.06 (m, 1H), 2.83 – 2.68 (m, 1H), 1.50 (d, J = 6.3 Hz, 1H)

^{1}H NMR (400 MHz, $CDCl_3$) cis δ 7.63 (d, J = 14.8 Hz, 1H), 7.59 – 7.29 (m, 7H), 7.26 – 7.20 (m, 1H), 6.66 (d, J = 16.0 Hz, 1H), 6.33 (dd, J = 16.0, 6.4 Hz, 1H), 6.32 – 6.29 (m, 1H), 4.88 – 4.79 (m, 2H), 3.22 – 3.06 (m, 1H), 2.83 – 2.68 (m, 1H), 1.45 (d, J = 6.4 Hz, 1H)

^{13}C NMR (100 MHz, $CDCl_3$) trans δ 145.3, 137.6, 137.5, 131.9, 131.2, 130.6, 129.8, 129.7, 129.2, 128.6 (2C), 128.2 (2C), 126.9, 125.6, 124.4 – 124.3 (m, 1C), 120.3, 79.1, 78.9, 38.6, 20.7

^{13}C NMR (100 MHz, $CDCl_3$) cis δ 145.0, 137.7, 137.6, 131.4, 131.9, 130.6, 129.8, 129.1, 128.6 (2C), 128.3 (2C), 126.8, 123.5 – 123.1 (m, 1C), 122.9, 120.7, 78.1, 77.9, 37.5, 21.3

^{19}F NMR (100 MHz, $CDCl_3$) trans δ -62.7

^{19}F NMR (100 MHz, $CDCl_3$) cis δ -62.7

HRMS (ESI) m/z: $[M + H]^+$ Calcd for $C_{22}H_{22}F_3O$ 345.1467; Found 345.1464.

3-((*E*)-benzylidene)-5-((*E*)-2-ethylstyryl)-2-methyltetrahydrofuran (3ai)

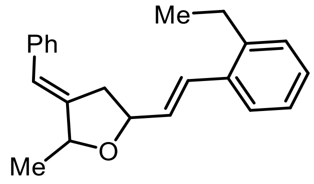

General scope procedure was followed. (*Z*)-3-bromo-4-phenylbut-3-en-2-ol **1a** (113.6 mg, 0.50 mmol, 1.0 equiv.), N-phenyl urea (6.8 mg, 0.050 mmol, 0.1 equiv.), (*E*)-1-(buta-1,3-dien-1-yl)-2-ethylbenzene **2h** (94.9 mg, 0.60 mmol, 1.2 equiv.), K_2CO_3 (83 mg, 0.60 mmol, 1.2 equiv.), and 1 mL of stock solution was used. The crude material was purified as a yellow oil using flash chromatography (silica, 100% hexanes - 95/5 hexanes/EtOAc) cis/trans (47:53)

Run 1: (101.7 mg, 0.334 mmol, 67%)
Run 2: (101.4 mg, 0.333 mmol, 67%)
Run 3: (97.4 mg, 0.320 mmol, 64%)
Average: 66% yield

^{1}H NMR (400 MHz, $CDCl_3$) trans δ 7.56 (d, J = 7.9 Hz, 1H), 7.45 – 7.34 (m, 4H), 7.32 – 7.15 (m, 4H), 7.01 (d, J = 16.0 Hz, 1H), 6.31 (m, 1H), 6.25 (dd, J = 16.0, 7.3 Hz, 1H), 4.71 – 4.63 (m, 1H), 4.63 – 4.53 (m, 1H), 3.23 – 3.08 (m, 1H), 2.87 – 2.70 (m, 3H), 1.56 (d, J = 6.3 Hz, 3H) 1.30 – 1.21 (m, 3H)

^{1}H NMR (400 MHz, $CDCl_3$) cis δ 7.50 (d, J = 7.2 Hz, 1H), 7.45 – 7.34 (m, 4H), 7.32 – 7.15 (m, 4H), 6.95 (d, J = 16.0 Hz, 1H), 6.35 (m, 1H), 6.19 (dd, J = 16.0, 6.8 Hz, 1H), 4.92 – 4.85 (m, 2H), 3.23 – 3.08 (m, 1H), 2.87 – 2.70 (m, 3H), 1.51 (d, J = 6.4 Hz, 3H), 1.30 – 1.21 (m, 3H)

^{13}C NMR (100 MHz, $CDCl_3$) trans δ 145.7, 141.7, 137.7, 135.0, 130.8, 129.8, 129.8, 128.7 128.5 (2C), 128.2 (2C), 127.0, 126.2, 126.1, 120.0, 79.8, 78.7, 38.7, 26.4, 20.7, 15.4

^{13}C NMR (100 MHz, $CDCl_3$) cis δ 145.4, 141.7, 137.6, 135.1, 131.2, 128.8, 128.8, 128.7, 128.4 (2C), 128.2 (2C), 126.9, 126.1, 126.1, 120.4, 78.4, 77.9, 37.6, 26.4, 21.3, 15.4

HRMS (ESI) m/z: $[M + H]^+$ Calcd for $C_{23}H_{27}O$ 305.1906; Found 305.1895.

2-((*E*)-2-(4-((*E*)-benzylidene)-5-methyltetrahydrofuran-2-yl)vinyl)furan (3aj)

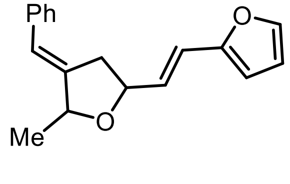

General scope procedure was followed. (*Z*)-3-bromo-4-phenylbut-3-en-2-ol **1a** (113.6 mg, 0.50 mmol, 1.0 equiv.), N-phenyl urea (6.8 mg, 0.050 mmol, 0.1 equiv.), (*E*)-2-(buta-1,3-dien-1-yl)furan **2j** (90.1 mg, 0.75 mmol, 1.5 equiv.), K_2CO_3 (83 mg, 0.60 mmol, 1.2 equiv.), and 1 mL of stock solution was used. The crude material was purified as a yellow oil using flash chromatography (silica, 100% hexanes - 95/5 hexanes/EtOAc) cis/trans (44:56)

Run 1: (69.8 mg, 0.262 mmol, 52%)
Run 2: (72.1 mg, 0.271 mmol, 54%)
Run 3: (66.3 mg, 0.249 mmol, 50%)
Average: 52% yield

^{1}H NMR (400 MHz, $CDCl_3$) trans δ 7.39 – 7.28 (m, 5H), 7.27 – 7.18 (m, 1H), 6.51 (d, J = 16.0 Hz, 1H), 6.40 – 6.14 (m, 4H), 4.66 – 4.55 (m, 1H), 4.52 – 4.42 (m, 1H), 3.18 – 3.01 (m, 1H), 2.81 – 2.63 (m, 1H), 1.48 (d, J = 6.3 Hz, 3H)

^{1}H NMR (400 MHz, $CDCl_3$) cis δ 7.39 – 7.28 (m, 5H), 7.27 – 7.18 (m, 1H), 6.45 (d, J = 16.0 Hz, 1H), 6.40 – 6.14 (m, 4H), 4.87 – 4.73 (m, 2H), 3.18 – 3.01 (m, 1H), 2.81 – 2.63 (m, 1H), 1.43 (d, J = 6.4 Hz, 3H)

^{13}C NMR (100 MHz, $CDCl_3$) trans δ 152.3, 145.6, 142.2, 137.7, 128.5 (2C), 128.2 (2C), 127.9, 126.7, 120.2, 120.1, 111.4, 108.4, 79.0, 78.7, 38.6, 20.8

^{13}C NMR (100 MHz, $CDCl_3$) cis δ 152.4, 145.3, 142.1, 137.7, 128.5 (2C), 128.4, 128.2 (2C), 126.7, 120.5, 119.3, 111.4, 108.3, 77.8, 77.8, 37.5, 21.2

HRMS (ESI) m/z: $[M + H]^+$ Calcd for $C_{19}H_{21}O_2$ 267.1386; Found 267.1377.

3-((*E*)-benzylidene)-2-methyl-5-((*E*)-2-(thiophen-2-yl)vinyl)tetrahydrofuran (3ak)

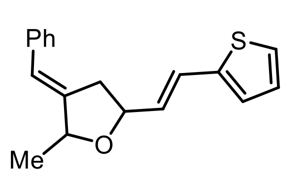

General scope procedure was followed. (*Z*)-3-bromo-4-phenylbut-3-en-2-ol **1a** (113.6 mg, 0.50 mmol, 1.0 equiv.), N-phenyl urea (6.8 mg, 0.050 mmol, 0.1 equiv.), (*E*)-2-(buta-1,3-dien-1-yl)thiophene **2k** (81.7 µL, 0.60 mmol, 1.2 equiv.), K_2CO_3 (83 mg, 0.60 mmol, 1.2 equiv.), and 1 mL of stock solution was used. The crude material was purified as a yellow oil using flash chromatography (silica, 100% hexanes - 95/5 hexanes/EtOAc) cis/trans (45:55)

<u>Run 1:</u> (73.8 mg, 0.261 mmol, 52%)
<u>Run 2:</u> (78.7 mg, 0.279 mmol, 56%)
<u>Run 3:</u> (74.9 mg, 0.265 mmol, 53%)
Average: 54% yield

^{1}H NMR (400 MHz, $CDCl_3$) trans δ 7.39 – 7.28 (m, 4H), 7.27 – 7.19 (m, 1H), 7.19 – 7.11 (m, 1H), 6.96 – 6.92 (m, 2H), 6.81 (d, J = 16.0 Hz, 1H), 6.24 (s, 1H), 6.20 – 6.04 (m, 1H), 4.64 – 4.54 (m, 1H), 4.52 – 4.42 (m, 1H), 3.19 – 3.02 (m, 1H), 2.81 – 2.65 (m, 1H), 1.48 (d, J = 6.3 Hz, 3H)

^{1}H NMR (400 MHz, $CDCl_3$) cis δ 7.39 – 7.28 (m, 4H), 7.27 – 7.19 (m, 1H), 7.19 – 7.11 (m, 1H), 6.99 – 6.96 (m, 2H), 6.75 (d, J = 16.0 Hz, 1H), 6.28 (s, 1H), 6.20 – 6.04 (m, 1H), 4.86 – 4.72 (m, 2H), 3.19 – 3.02 (m, 1H), 2.81 – 2.65 (m, 1H), 1.43 (d, J = 6.3 Hz, 3H)

^{13}C NMR (100 MHz, $CDCl_3$) trans δ 145.6, 141.9, 137.7, 128.9, 128.6 (2C), 128.2 (2C), 127.5, 126.8, 126.3, 124.7, 124.6, 120.2, 79.2, 78.8, 38.6, 20.7

^{13}C NMR (100 MHz, $CDCl_3$) cis δ 145.3, 141.8, 137.7, 129.4, 128.6 (2C), 128.3 (2C), 127.5, 126.8, 126.1, 125.4, 124.4, 120.5, 77.9, 77.9, 37.5, 21.2

HRMS (ESI) m/z: $[M + H]^+$ Calcd for $C_{19}H_{21}OS$ 283.1157; Found 283.1146.

3-((*E*)-benzylidene)-2-methyl-5-((*E*)-2-(thiophen-3-yl)vinyl)tetrahydrofuran (3al)

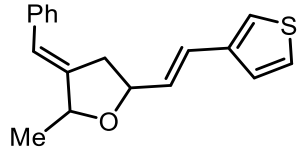

General scope procedure was followed. (*Z*)-3-bromo-4-phenylbut-3-en-2-ol **1a** (113.6 mg, 0.50 mmol, 1.0 equiv.), N-phenyl urea (6.8 mg, 0.050 mmol, 0.1 equiv.), (*E*)-3-(buta-1,3-dien-1-yl)thiophene **2l** (81.7 mg, 0.60 mmol, 1.2 equiv.), K_2CO_3 (83 mg, 0.60 mmol, 1.2 equiv.), and 1 mL of stock solution was used. The crude material was purified as a yellow oil using flash chromatography (silica, 100% hexanes - 95/5 hexanes/EtOAc) cis/trans (27:73)

Run 1: (61.8 mg, 0.219 mmol, 44%)
Run 2: (61.3 mg, 0.217 mmol, 43%)
Run 3: (55.9 mg, 0.198 mmol, 40%)
Average: 42% yield

^{1}H NMR (400 MHz, $CDCl_3$) trans δ 7.37 – 7.12 (m, 8H), 6.68 (d, J = 16.0 Hz, 1H), 6.26 – 6.21 (m, 1H), 6.16 (dd, J = 16.0, 7.2 Hz, 1H), 4.62 – 4.54 (m, 1H), 4.51 – 4.42 (m, 1H), 3.17 – 3.02 (m, 1H), 2.80 – 2.65 (m, 1H), 1.48 (d, J = 6.3 Hz, 3H)

^{1}H NMR (400 MHz, $CDCl_3$) cis δ 7.37 – 7.12 (m, 8H), 6.62 (d, J = 16.0 Hz, 1H), 6.29 – 6.26 (m, 1H), 6.09 (dd, J = 16.0, 6.6 Hz, 1H), 4.85 – 4.71 (m, 2H), 3.17 – 3.02 (m, 1H), 2.80 – 2.65 (m, 1H), 1.42 (d, J = 6.4 Hz, 3H)

^{13}C NMR (100 MHz, $CDCl_3$) trans δ 145.7, 139.3, 137.7, 129.6, 128.6 (2C), 128.2 (2C), 126.8, 126.6, 126.2, 125.2, 122.8, 120.1, 79.5, 78.7, 38.7, 20.7

^{13}C NMR (100 MHz, $CDCl_3$) cis δ 145.5, 139.4, 137.7, 129.2, 128.6 (2C), 128.3, 128.2 (2C), 126.6, 126.2, 125.5, 122.6, 120.5, 78.2, 77.9, 37.6, 21.3

HRMS (ESI) m/z: $[M + H]^+$ Calcd for $C_{19}H_{21}OS$ 283.1157; Found 283.1140.

5-((*E*)-2-(4-((*E*)-benzylidene)-5-methyltetrahydrofuran-2-yl)vinyl)benzo[*d*][1,3]dioxole (3am)

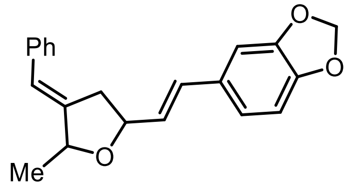

General scope procedure was followed. (*Z*)-3-bromo-4-phenylbut-3-en-2-ol **1a** (113.6 mg, 0.50 mmol, 1.0 equiv.), N-phenyl urea (6.8 mg, 0.050 mmol, 0.1 equiv.), (*E*)-5-(buta-1,3-dien-1-yl)benzo[d][1,3]dioxole **2m** (105 mg, 0.60 mmol, 1.2 equiv.), K_2CO_3 (83 mg, 0.60 mmol, 1.2 equiv.), and 1 mL of stock solution was used. The crude material was purified as a yellow oil using flash chromatography (silica, 100% hexanes - 95/5 hexanes/EtOAc)

cis/trans (47:53)

Run 1: (94.7 mg, 0.296 mmol, 59%)
Run 2: (96.2 mg, 0.300 mmol, 60%)
Run 3: (92.0 mg, 0.288 mmol, 58%)
Average: 59% yield

^{1}H NMR (400 MHz, $CDCl_3$) trans δ 7.39 – 7.29 (m, 4H), 7.26 – 7.19 (m, 1H), 6.95 (s, 1H), 6.85 – 6.78 (m, 1H), 6.76 (d, J = 8.0 Hz, 1H), 6.59 (d, J = 16.0 Hz, 1H), 6.26 – 6.22 (m, 1H), 6.14 (dd, J =16.0, 7.3 Hz, 1H), 5.94 (s, 2H), 4.63 – 4.55 (m, 1H), 4.51 – 4.43 (m, 1H), 3.18 – 3.01 (m, 1H), 2.80 – 2.64 (m, 1H), 1.49 (d, J = 6.3 Hz, 3H)

^{1}H NMR (400 MHz, $CDCl_3$) cis δ 7.39 – 7.29 (m, 4H), 7.26 – 7.19 (m, 1H), 6.92 (s, 1H), 6.85 – 6.78 (m, 1H), 6.74 (d, J = 8.0 Hz, 1H), 6.53 (d, J = 16.0 Hz, 1H), 6.30 – 6.26 (m, 1H), 6.08 (dd, J = 16.0, 6.9 Hz, 1H), 5.93 (s, 2H), 4.86 – 4.71 (m, 2H), 3.18 – 3.01 (m, 1H), 2.80 – 2.64 (m, 1H), 1.43 (d, J = 6.2 Hz, 3H)

^{13}C NMR (100 MHz, $CDCl_3$) trans δ 148.1, 147.5, 145.7, 137.7, 132.1, 131.1, 128.5 (2C), 128.2 (2C), 127.5, 126.7, 121.5, 120.0, 108.4, 105.9, 101.2, 79.6, 78.7, 38.7, 20.7

^{13}C NMR (100 MHz, $CDCl_3$) cis δ 148.1, 147.4, 145.5, 137.7, 131.2, 131.1, 128.5 (2C), 128.2 (2C), 127.9, 126.7, 121.4, 120.4, 108.4, 105.9, 101.2, 78.3, 77.9, 37.7, 21.2

HRMS (ESI) m/z: $[M + H]^+$ Calcd for $C_{21}H_{21}O_3$ 321.1491; Found 321.1485.

3-((*E*)-2-(4-((*E*)-benzylidene)-5-methyltetrahydrofuran-2-yl)vinyl)pyridine (3an)

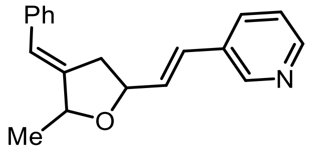

General scope procedure was followed. (*Z*)-3-bromo-4-phenylbut-3-en-2-ol **1a** (113.6 mg, 0.50 mmol, 1.0 equiv.), N-phenyl urea (6.8 mg, 0.050 mmol, 0.1 equiv.), (*E*)-3-(buta-1,3-dien-1-yl)pyridine **2n** (78.7 mg, 0.60 mmol, 1.2 equiv.), K_2CO_3 (83 mg, 0.60 mmol, 1.2 equiv.), and 1 mL of stock solution was used. The crude material was purified as a yellow oil using flash chromatography (silica, 100% hexanes - 95/5 hexanes/EtOAc) cis/trans (48:52)

<u>Run 1:</u> (71.7 mg, 0.259 mmol, 52%)
<u>Run 2:</u> (74.1 mg, 0.267 mmol, 53%)
<u>Run 3:</u> (76.5 mg, 0.276 mmol, 55%)
Average: 54% yield

^1H NMR (400 MHz, $CDCl_3$) trans δ 8.60 (s, 1H), 8.49 – 8.38 (m, 1H), 7.68 (dt, J = 14.9, 1.9 Hz, 1H), 7.36 – 7.26 (m, 4H), 7.24 – 7.16 (m, 2H), 6.64 (d, J = 16.0 Hz, 1H), 6.41 – 6.21 (m, 2H), 4.63 – 4.54 (m, 1H), 4.54 – 4.46 (m, 1H), 3.19 – 3.02 (m, 1H), 2.79 – 2.64 (m, 1H), 1.47 (d, J = 6.3 Hz, 3H)

^1H NMR (400 MHz, $CDCl_3$) cis δ 8.57 (s, 1H), 8.49 – 8.38 (m, 1H), 7.66 (dt, J = 14.8, 2.1 Hz, 1H), 7.36 – 7.26 (m, 4H), 7.24 – 7.16 (m, 2H), 6.59 (d, J = 16.0 Hz, 1H), 6.41 – 6.21 (m, 2H), 4.85 – 4.74 (m, 2H), 3.19 – 3.02 (m, 1H), 2.79 – 2.64 (m, 1H), 1.42 (d, J = 6.3 Hz, 3H)

^{13}C NMR (100 MHz, $CDCl_3$) trans δ 148.9, 148.5, 145.1, 137.5, 133.0, 132.2, 131.7, 128.5 (2C), 128.4, 128.1 (2C), 126.7, 123.5, 120.2, 79.0, 78.7, 38.4, 20.6

^{13}C NMR (100 MHz, $CDCl_3$) cis δ 148.8, 148.5, 144.9, 137.5, 133.0, 132.3 132.1, 128.5 (2C), 128.2 (2C), 127.4, 126.8, 123.5, 120.6, 78.0, 77.8, 37.3, 21.2

HRMS (ESI) m/z: $[M + H]^+$ Calcd for $C_{19}H_{20}NO$ 278.1545; Found 278.1548.

3-((*E*)-2-(4-((*E*)-benzylidene)-5-methyltetrahydrofuran-2-yl)vinyl)-1-methyl-1H-indole (3ao)

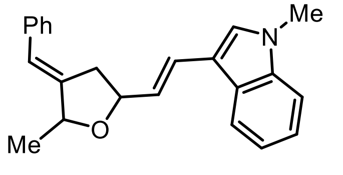

General scope procedure was followed. (*Z*)-3-bromo-4-phenylbut-3-en-2-ol **1a** (113.6 mg, 0.50 mmol, 1.0 equiv.), N-phenyl urea (6.8 mg, 0.050 mmol, 0.1 equiv.), (*E*)-3-(buta-1,3-dien-1-yl)-1-methyl-1H-indole **2o** (110 mg, 0.60 mmol, 1.2 equiv.), K_2CO_3 (83 mg, 0.60 mmol, 1.2 equiv.), and 1 mL of stock solution was used. The crude material was purified as a yellow/orange oil using flash chromatography (silica, 100% hexanes - 95/5 hexanes/EtOAc) cis/trans (43:57)

<u>Run 1:</u> (93.6 mg, 0.284 mmol, 57%)
<u>Run 2:</u> (93.2 mg, 0.283 mmol, 57%)
<u>Run 3:</u> (96.9 mg, 0.294 mmol, 59%)
Average: 57% yield

^{1}H NMR (400 MHz, $CDCl_3$) trans δ 7.91 (d, J = 8.0 Hz, 1H), 7.40 – 7.15 (m, 8H), 7.11 (s, 1H), 6.87 (d, J = 16.0 Hz, 1H), 6.34 – 6.20 (m, 2H), 4.67 – 4.58 (m, 1H), 4.57 – 4.48 (m, 1H), 3.74 (s, 3H), 3.23 – 3.07 (m, 1H), 2.88 – 2.73 (m, 1H), 1.53 (d, J = 6.4 Hz, 3H)

^{1}H NMR (400 MHz, $CDCl_3$) cis δ 7.88 (d, J = 8.0 Hz, 1H), 7.40 – 7.15 (m, 8H), 7.09 (s, 1H), 6.82 (d, J = 16.0 Hz, 1H), 6.34 – 6.20 (m, 2H), 4.92 – 4.77 (m, 2H), 3.73 (s, 3H), 3.23 – 3.07 (m, 1H), 2.88 – 2.73 (m, 1H), 1.48 (d, J = 6.4 Hz, 3H)

^{13}C NMR (100 MHz, $CDCl_3$) trans δ 146.3, 137.8, 137.6, 128.7, 128.5 (2C), 128.2 (2C), 126.6, 126.2, 125.6, 124.9, 122.2, 120.3, 120.0, 119.8, 113.0, 109.5, 80.8, 79.3, 39.1, 32.9, 20.7

^{13}C NMR (100 MHz, $CDCl_3$) cis δ 146.1, 137.8, 137.6, 128.7, 128.5 (2C), 128.3 (2C), 126.6, 126.2, 125.4, 124.6, 122.2, 120.3, 120.2, 120.0, 113.0, 109.5, 78.6, 77.8, 38.1, 32.9, 21.2

HRMS (ESI) m/z: $[M + H]^+$ Calcd for $C_{23}H_{23}NO$ 330.1859; Found 330.1852.

3-((*E*)-benzylidene)-5-((*E*)-hept-1-en-1-yl)-2-methyltetrahydrofuran (3ap)

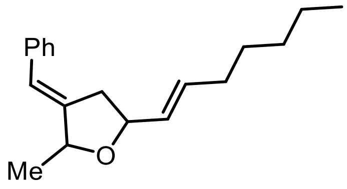

General scope procedure was followed. (*Z*)-3-bromo-4-phenylbut-3-en-2-ol **1a** (113.6 mg, 0.50 mmol, 1.0 equiv.), N-phenyl urea (6.8 mg, 0.050 mmol, 0.1 equiv.), (*E*)-nona-1,3-diene **2p** (93.2 mg, 0.75 mmol, 1.5 equiv.), K_2CO_3 (83 mg, 0.60 mmol, 1.2 equiv.), and 1 mL of stock solution was used. The crude material was purified as a yellow oil using flash chromatography (silica, 100% hexanes - 95/5 hexanes/EtOAc) cis/trans (56:44)

Run 1: (81.5 mg, 0.301 mmol, 60%)
Run 2: (80.9 mg, 0.299 mmol, 60%)
Run 3: (87.2 mg, 0.322 mmol, 64%)
Average: 62% yield

^{1}H NMR (400 MHz, $CDCl_3$) trans δ 7.38 – 7.28 (m, 4H), 7.24 – 7.18 (m, 1H), 6.21 (q, J = 2.8 Hz, 1H), 5.85 – 5.67 (m, 1H), 5.61 – 5.47 (m, 1H), 4.60 – 4.47 (m, 1H), 4.33 – 4.23 (m, 1H), 3.09 – 2.92 (m, 1H), 2.69 – 2.54 (m, 1H), 2.10 – 1.97 (m, 2H), 1.48 – 1.18 (m, 9H), 0.94 – 0.82 (m, 3H)

^{1}H NMR (400 MHz, $CDCl_3$) cis δ 7.38 – 7.28 (m, 4H), 7.24 – 7.18 (m, 1H), 6.25 (q, J = 2.4 Hz, 1H), 5.85 – 5.67 (m, 1H), 5.61 – 5.47 (m, 1H), 4.79 – 4.70 (m, 1H), 4.60 – 4.47 (m, 1H), 3.09 – 2.92 (m, 1H), 2.69 – 2.54 (m, 1H), 2.10 – 1.97 (m, 2H), 1.48 – 1.18 (m, 9H), 0.94 – 0.82 (m, 3H)

^{13}C NMR (100 MHz, $CDCl_3$) trans δ 146.3, 137.9, 135.0, 129.7, 128.5 (2C), 128.2 (2C), 126.7, 119.8, 79.8, 78.5, 38.6, 32.4, 31.6, 28.8, 22.7, 20.7, 14.2

^{13}C NMR (100 MHz, $CDCl_3$) cis δ 146.1, 137.9, 133.8, 130.0, 128.5 (2C), 128.3 (2C), 126.7, 120.1, 78.4, 77.7, 37.7, 32.3, 31.6, 28.9, 22.7, 21.2, 14.2

HRMS (ESI) m/z: $[M + H]^+$ Calcd for $C_{19}H_{26}O$ 271.2063; Found 271.2053.

3-((*E*)-benzylidene)-2-methyl-5-((*E*)-4-phenylbut-1-en-1-yl)tetrahydrofuran (3aq)

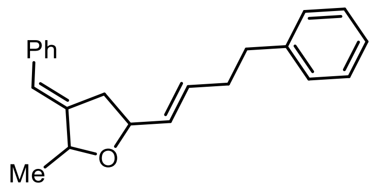

General scope procedure was followed. (*Z*)-3-bromo-4-phenylbut-3-en-2-ol **1a** (113.6 mg, 0.50 mmol, 1.0 equiv.), N-phenyl urea (6.8 mg, 0.050 mmol, 0.1 equiv.), (*E*)-hexa-3,5-dien-1-ylbenzene **2q** (118.7 mg, 0.75 mmol, 1.5 equiv.), K_2CO_3 (83 mg, 0.60 mmol, 1.2 equiv.), and 1 mL of stock solution was used. The crude material was purified as a yellow oil using flash chromatography (silica, 100% hexanes - 95/5 hexanes/EtOAc) cis/trans (47:53)

Run 1: (79.2 mg, 0.260 mmol, 52%)
Run 2: (79.8 mg, 0.262 mmol, 52%)
Run 3: (84.9 mg, 0.279 mmol, 56%)
Average: 53% yield

^{1}H NMR (400 MHz, $CDCl_3$) trans δ 7.41 – 7.14 (m, 10H), 6.23 (q, J = 2.9 Hz, 1H), 5.92 – 5.74 (m, 1H), 5.68 – 5.54 (m, 1H), 4.63 – 4.49 (m, 1H), 4.36 – 4.27 (m, 1H), 3.09 – 2.94 (m, 1H), 2.82 – 2.53 (m, 4H), 2.53 – 2.31 (m, 2H), 1.47 (d, J = 6.3 Hz, 3H)

^{1}H NMR (400 MHz, $CDCl_3$) cis δ 7.41 – 7.14 (m, 10H), 6.27 (q, J = 2.3 Hz, 1H), 5.92 – 5.74 (m, 1H), 5.68 – 5.54 (m, 1H), 4.80 – 4.72 (m, 1H), 4.63 – 4.49 (m, 1H), 3.09 – 2.94 (m, 1H), 2.82 – 2.53 (m, 4H), 2.53 – 2.31 (m, 2H), 1.41 (d, J = 6.4 Hz, 3H)
^{13}C NMR (100 MHz, $CDCl_3$) trans δ 146.0, 141.8, 137.8, 133.6, 130.5, 128.5 (3C), 128.4 (2C), 128.2 (2C), 126.6 (2C), 126.0, 119.8, 79.6, 78.5, 38.5, 35.5, 34.3, 20.6

^{13}C NMR (100 MHz, $CDCl_3$) cis δ 145.8, 141.8, 137.8, 132.3, 130.8, 128.5 (3C), 128.4 (2C), 128.2 (2C), 126.7 (2C), 125.9, 120.2, 78.2, 77.7, 37.5, 35.6, 34.2, 21.2

HRMS (ESI) m/z: $[M + H]^+$ Calcd for $C_{22}H_{25}O$ 305.1906; Found 305.1908.

(*E*)-3-benzylidene-2-methyl-5-(1-phenylvinyl)tetrahydrofuran (3ar)

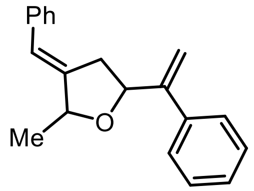

General scope procedure was followed. (*Z*)-3-bromo-4-phenylbut-3-en-2-ol **1a** (113.6 mg, 0.50 mmol, 1.0 equiv.), N-phenyl urea (6.8 mg, 0.050 mmol, 0.1 equiv.), buta-1,3-dien-2-ylbenzene **2r** (78.1 μL, 0.60 mmol, 1.2 equiv.), K_2CO_3 (83 mg, 0.60 mmol, 1.2 equiv.), and 1 mL of stock solution was used. The crude material was purified as a yellow oil using flash chromatography (silica, 100% hexanes - 95/5 hexanes/EtOAc) cis/trans (36:64)

Run 1: (86.5 mg, 0.313 mmol, 63%)
Run 2: (89.3 mg, 0.323 mmol, 65%)
Run 3: (91.2 mg, 0.330 mmol, 66%)
Average: 64% yield

^{1}H NMR (400 MHz, $CDCl_3$) trans δ 7.50 – 7.15 (m, 10H), 6.25 (s, 1H), 5.56 (d, J = 1.2 Hz, 1H), 5.43 (d, J = 1.2 Hz, 1H), 4.95 – 4.82 (m, 1H), 4.74 – 4.64 (m, 1H), 3.18 – 3.00 (m, 1H), 2.75 – 2.54 (m, 1H), 1.53 (dd, J = 6.0, 2.8 Hz, 3H)

^{1}H NMR (400 MHz, $CDCl_3$) cis δ 7.50 – 7.15 (m, 10H), 6.27 (s, 1H), 5.45 (d, J = 1.6 Hz, 1H), 5.38 (d, J = 1.6 Hz, 1H), 5.21 – 5.13 (m, 1H), 4.95 – 4.82 (m, 1H), 3.18 – 3.00 (m, 1H), 2.75 – 2.54 (m, 1H), 1.48 (dd, J = 6.4, 2.8 Hz, 3H)

^{13}C NMR (100 MHz, $CDCl_3$) trans δ 148.3, 145.7, 139.5, 137.6, 128.5 (4C), 128.2 (2C), 127.8, 126.9 (2C), 126.7, 120.1, 113.2, 79.8, 78.8, 38.3, 20.7

^{13}C NMR (100 MHz, $CDCl_3$) cis δ 148.7, 145.3, 139.6, 137.6, 128.5 (4C), 128.3 (2C), 127.8, 126.8 (2C), 126.7, 120.3, 112.4, 78.6, 78.3, 37.0, 21.2

HRMS (ESI) m/z: $[M + H]^+$ Calcd for $C_{20}H_{21}O$ 277.1593; Found 277.1586.

4-((*E*)-benzylidene)-2,5-dimethyl-2-((*E*)-styryl)tetrahydrofuran (3as)

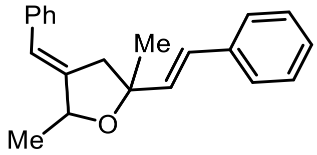

General scope procedure was followed. (*Z*)-3-bromo-4-phenylbut-3-en-2-ol **1a** (113.6 mg, 0.50 mmol, 1.0 equiv.), N-phenyl urea (6.8 mg, 0.050 mmol, 0.1 equiv.), (*E*)-(3-methylbuta-1,3-dien-1-yl)benzene **2s** (86.5 mg, 0.60 mmol, 1.2 equiv.), K_2CO_3 (83 mg, 0.60 mmol, 1.2 equiv.), and 1 mL of stock solution was used. The crude material was purified as a yellow oil using flash chromatography (silica, 100% hexanes - 95/5 hexanes/EtOAc) cis/trans (49:51)

Run 1: (112.4 mg, 0.387 mmol, 77%)
Run 2: (109.8 mg, 0.378 mmol, 76%)
Run 3: (104.1 mg, 0.372 mmol, 74%)
Average: 76% yield

^{1}H NMR (400 MHz, $CDCl_3$) trans δ 7.50 – 7.19 (m, 10H), 6.74 – 6.19 (m, 3H), 4.90 – 4.80 (m, 1H), 3.16 – 2.82 (m, 2H), 1.60 (s, 3H), 1.56 – 1.50 (m, 3H)

^{1}H NMR (400 MHz, $CDCl_3$) cis δ 7.50 – 7.19 (m, 10H), 6.74 – 6.19 (m, 3H), 4.80 – 4.70 (m, 1H), 3.16 – 2.82 (m, 2H), 1.56 – 1.50 (m, 3H), 1.45 (s, 3H)

^{13}C NMR (100 MHz, $CDCl_3$) trans δ 146.0, 137.7, 136.9, 135.4, 128.6 (2C), 128.4 (2C) 128.3 (2C), 127.7, 127.5, 126.6, 126.6 (2C), 120.6, 82.1, 81.9, 43.6, 28.0, 21.5

^{13}C NMR (100 MHz, $CDCl_3$) cis δ 146.2, 137.8, 137.0, 134.2, 128.6 (2C), 128.4 (2C), 128.2 (2C), 127.8, 127.5, 126.7, 126.5 (2C), 120.2, 77.5, 77.2, 43.2, 25.2, 21.8

HRMS (ESI) m/z: $[M + H]^+$ Calcd for $C_{21}H_{23}O$ 291.1750; Found 291.1744.

4-((*E*)-benzylidene)-2-((*E*)-hex-1-en-1-yl)-2,5-dimethyltetrahydrofuran (3at)

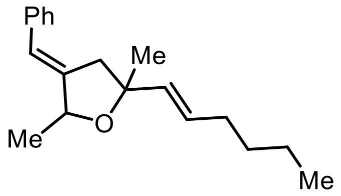

General scope procedure was followed. (*Z*)-3-bromo-4-phenylbut-3-en-2-ol **1a** (113.6 mg, 0.50 mmol, 1.0 equiv.), N-phenyl urea (6.8 mg, 0.050 mmol, 0.1 equiv.), (*E*)-2-methylocta-1,3-diene **2t** (93.2 mg, 0.75 mmol, 1.5 equiv.), K_2CO_3 (83 mg, 0.60 mmol, 1.2 equiv.), and 1 mL of stock solution was used. The crude material was purified as a yellow oil using flash chromatography (silica, 100% hexanes - 95/5 hexanes/EtOAc) cis/trans (47:53)

Run 1: (61.1 mg, 0.226 mmol, 45%)
Run 2: (66.0 mg, 0.244 mmol, 49%)
Run 3: (67.1 mg, 0.248 mmol, 50%)
Average: 48% yield

^{1}H NMR (400 MHz, $CDCl_3$) trans δ 7.38 – 7.27 (m, 4H), 7.26 – 7.18 (m, 1H), 6.20 (s, 1H), 5.75 – 5.39 (m, 2H), 4.76 – 4.66 (m, 1H), 2.99 – 2.64 (m, 2H), 2.10 – 1.94 (m, 2H), 1.47 – 1.19 (m, 10H), 0.94 – 0.83 (m, 3H)

^{1}H NMR (400 MHz, $CDCl_3$) cis δ 7.38 – 7.27 (m, 4H), 7.26 – 7.18 (m, 1H), 6.26 (s, 1H), 5.75 – 5.39 (m, 2H), 4.65 – 4.56 (m, 1H), 2.99 – 2.64 (m, 2H), 2.10 – 1.94 (m, 2H), 1.47 – 1.19 (m, 10H), 0.94 – 0.83 (m, 3H)

^{13}C NMR (100 MHz, $CDCl_3$) trans δ 147.0, 138.0, 135.6, 129.0, 128.4 (2C), 128.2 (2C), 126.5, 119.8, 81.7, 81.7, 43.0, 32.1, 31.5, 27.9, 22.2, 21.4, 14.0

^{13}C NMR (100 MHz, $CDCl_3$) cis δ 146.7, 137.9, 134.1, 129.3, 128.4 (2C), 128.2 (2C), 126.6, 120.4, 77.1, 76.9, 43.6, 32.0, 31.5, 24.6, 22.4, 21.9, 14.1

HRMS (ESI) m/z: $[M + H]^+$ Calcd for $C_{19}H_{27}O$ 271.2063; Found 271.2055.

3-((*E*)-benzylidene)-2-methyl-5-((*E*)-1-phenylprop-1-en-2-yl)tetrahydrofuran (3au)

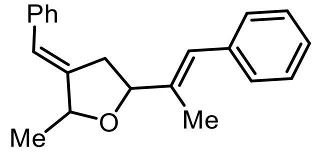

General scope procedure was followed. (*Z*)-3-bromo-4-phenylbut-3-en-2-ol **1a** (113.6 mg, 0.50 mmol, 1.0 equiv.), N-phenyl urea (6.8 mg, 0.050 mmol, 0.1 equiv.), (*E*)-(2-methylbuta-1,3-dien-1-yl)benzene **2u** (108.2 mg, 0.75 mmol, 1.5 equiv.), K_2CO_3 (83 mg, 0.60 mmol, 1.2 equiv.), and 1 mL of stock solution was used. The crude material was purified as a yellow oil using flash chromatography (silica, 100% hexanes - 95/5 hexanes/EtOAc) cis/trans (47:53)

<u>Run 1:</u> (67.7 mg, 0.233 mmol, 47%)
<u>Run 2:</u> (69.1 mg, 0.238 mmol, 48%)
<u>Run 3:</u> (73.5 mg, 0.253 mmol, 51%)
Average: 48% yield

^{1}H NMR (400 MHz, $CDCl_3$) trans δ 7.41 – 7.17 (m, 10H), 6.66 (s, 1H), 6.34 – 6.25 (m, 1H), 4.76 – 4.53 (m, 1H), 4.53 – 4.44 (m, 1H), 3.19 – 3.00 (m, 1H), 2.88 – 2.70 (m, 1H), 1.93 (s, 3H), 1.54 – 1.49 (m, 3H)

^{1}H NMR (400 MHz, $CDCl_3$) cis δ 7.41 – 7.17 (m, 10H), 6.61 (s, 1H), 6.34 – 6.25 (m, 1H), 4.99 – 4.85 (m, 1H), 4.76 – 4.53 (m, 1H), 3.19 – 3.00 (m, 1H), 2.88 – 2.70 (m, 1H), 1.90 (s, 3H), 1.49 – 1.43 (m, 3H)

^{13}C NMR (100 MHz, $CDCl_3$) trans δ 145.8, 137.8, 137.7, 137.2, 129.1 (2C), 128.5 (2C), 128.2 (2C), 128.2 (2C), 126.7, 126.6, 126.4, 120.0, 83.8, 78.8, 36.9, 20.6, 13.6

^{13}C NMR (100 MHz, $CDCl_3$) cis δ 145.7, 137.9, 137.7, 137.7, 129.1 (2C), 128.5 (2C), 128.3 (2C), 128.2 (2C), 126.7, 126.5, 125.5, 120.2, 82.6, 78.8, 36.1, 21.4, 13.9

HRMS (ESI) m/z: $[M + H]^+$ Calcd for $C_{21}H_{23}O$ 291.1750; Found 291.1739.

Methodology Limitations

Substrates depicted below did not produce any desired heteroannulation product under standard reaction conditions, affording a small amount of an inseparable mix of side products.

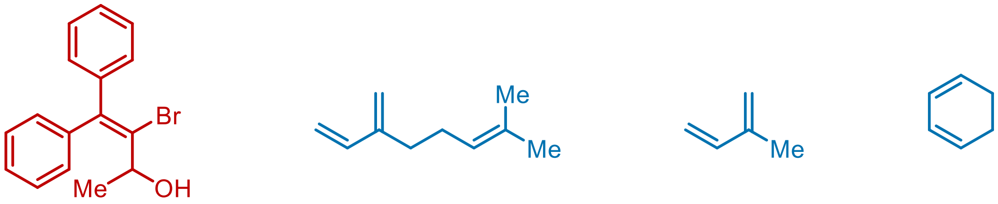

(Z) vs. (E) Diene

(Z)-1-phenylbutadiene (2a') synthesis

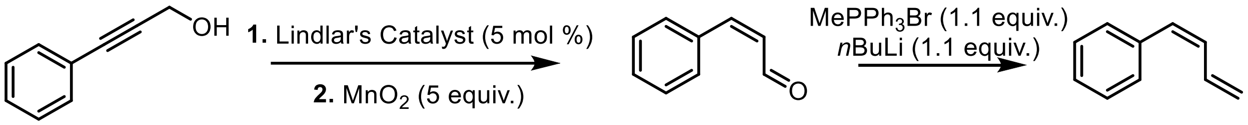

(*Z*)-3-phenylacrylaldehyde (S35)

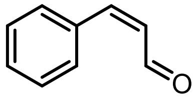

Prepared according to a modified literature procedure.[32] Quinoline (374 µL, 3.0 mmol, 0.30 equiv.) and Lindlar's Catalyst (120 mg, 10 wt%) was added to a degassed EtOAc (25 mL, 0.40 M) solution of 3-phenylprop-2-yn-1-ol (1.32 g, 10 mmol, 1.0 equiv.) in a round bottom flask equipped with a stir bar. A ballon filled with H_2 gas was then attached to flask. The reaction mixture was stirred at rt for 2 hours. After completion, the mixture was filtered through Celite. The collected organic layer was then washed with 1M HCl solution (3 X 15 mL). The organic layer was collected and washed with $MgSO_4$, then solvent was removed via low pressure. The crude colorless mixture was then diluted with hexanes (200 mL, 0.05 M). Subsequently, manganese (IV) dioxide (4.75 g, 60 mmol, 6 equiv.) was added to the reaction mixture. The reaction was stirred overnight in darkness at room temperature. Upon completion, the reaction mixture was filtered through a pad of celite and the filtrate was concentrated under reduced pressure. Crude material was purified via column chromatography on SiO_2 using 100% hexanes -> 20% EtOAc/Hex to afford (**S33**) as a slightly yellow oil (53%, 700 mg, 5.30 mmol). Material was stored at −20 °C and used promptly. Spectral data agree with that reported in the literature.[33]

^{1}H NMR (400 MHz, $CDCl_3$): δ 9.97 (d, J = 8.1 Hz, 1H), 7.63 (d, J = 11.7 Hz, 1H), 7.41 (bs, 5 H), 6.22 – 6.17 (m, 1H)

(*Z*)-buta-1,3-dien-1-ylbenzene (2a')

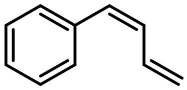

Prepared according to diene synthesis procedure A. Methyltriphenylphosphonium bromide (1.61 g, 4.50 mmol, 1.00 equiv.) was measured into an oven-dried round bottom flask equipped with a stir bar and septum. The flask was evacuated and refilled with N_2 three times before the addition of dry THF (30 mL, 0.2 M). The solution was then cooled to −78 °C. 2.5 M *n*BuLi in hexanes was then added (1.98 mL, 4.95 mmol, 1.1 equiv.). The solution was allowed to warm to rt (20 °C) over 1 h. (*Z*)-3-phenylacrylaldehyde (**S33**) (0.654 g, 4.95 mmol, 1.1 equiv.) was then added to the solution and the reaction was allowed to stir for 12 h. Upon completion, the solution was quenched with NH_4Cl (3 x 40 mL). The organic layer was extracted with Et_2O (100 mL) and washed with brine (3 X 40

mL), then dried over $MgSO_4$. Solvent was removed under reduced pressure. Crude material was purified via column chromatography on SiO_2 using 100% hexanes to afford (**2a**) as a colorless oil (78%, 456 mg, 3.51 mmol). Material was stored at −20 °C and used promptly. Spectral data agree with that reported in the literature.[34]

^{1}H NMR (400 MHz, $CDCl_3$): δ 7.39 – 7.34 (m, 4H), 7.30 – 7.25 (m, 1H), 6.92 (dt, J = 17.0, 11.2 Hz, 1H), 6.49 (d, J = 11.7 Hz, 1H), 6.30 (t, J = 11.4 Hz, 1H), 5.41 (d, J = 17.0 Hz, 1H), 5.25 (d, J = 10.1 Hz, 1H)

^{13}C NMR (100 MHz, $CDCl_3$): δ 137.5, 133.3, 130.9, 130.5, 129.1, 128.4, 127.2, 119.8

3-((*E*)-benzylidene)-2-methyl-5-((*E*)-styryl)tetrahydrofuran (3aa)

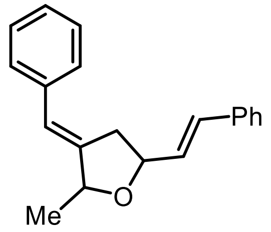

Bromoallyl alcohol **1a** (0.50 mmol, 1.0 equiv.), N-phenyl urea (6.8 mg, 0.050 mmol, 0.1 equiv.), and (*Z*)-buta-1,3-dien-1-ylbenzene **2a’** (84 µL, 0.60 mmol, 1.2 equiv.) were weighed/measured out in the above-mentioned order into a flame-dried 1-dram vial equipped with a small football-shaped stir bar and a cap with a silicone septum. 1.0 mL stock solution was prepared (in a flame-dried 1-dram vial equipped with a small football-shaped stir bar and a cap with a silicone septum) with solvent (0.2 mL DMF, 0.8 mL MIBK), palladium acetate (5.6 mg, 0.025 mmol, 0.05 equiv.), and 1,3,5-trimethoxybenzene (42.0 mg, 0.25 mmol, 0.5 equiv.) and left to stir for 30 min. Then, the stock solution was added to the first vial and left to stir for several minutes at room temperature. Potassium carbonate base (83 mg, 0.60 mmol, 1.2 equiv.) was added to the reaction mixture and a fresh septum was placed on the vial. The reaction mixture was then stirred at 110 °C in an aluminum block for 18 h at 650 rpm. After cooling to rt, the crude material was filtered through a plug of celite using ethyl acetate. The crude material was then analyzed via NMR to determine the d.r. and percent yield. cis/trans (47:53)

Run 1: (0.156 mmol, 31%)
Run 2: (0.153 mmol, 31%)
Run 3: (0.168 mmol, 34%)
Average: 32% yield

(*E*)-3-(cyclohexylmethylene)-2-methyl-5-((*E*)-styryl)tetrahydrofuran (3ma)

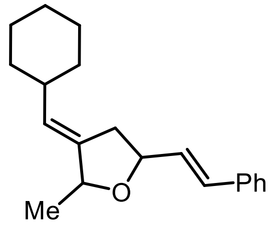

Bromoallyl alcohol **1m** (0.50 mmol, 1.0 equiv.), N-phenyl urea (6.8 mg, 0.050 mmol, 0.1 equiv.), and (*Z*)-buta-1,3-dien-1-ylbenzene **2a’** (84 µL, 0.60 mmol, 1.2 equiv.) were weighed/measured out in the above-mentioned order into a flame-dried 1-dram vial equipped with a small football-shaped stir bar and a cap with a silicone septum. 1.0 mL stock solution was prepared (in a flame-dried 1-dram vial equipped with a small football-shaped stir bar and a cap with a silicone septum) with solvent (0.2 mL DMF, 0.8 mL MIBK), palladium acetate (5.6 mg, 0.025 mmol, 0.05 equiv.), and 1,3,5-trimethoxybenzene (42.0 mg, 0.25 mmol, 0.5 equiv.) and left to stir for 30 min. Then, the stock solution was added to the first vial and left to stir for several minutes at room temperature. Potassium carbonate base (83 mg, 0.60 mmol, 1.2 equiv.) was added to the reaction mixture and a fresh septum was placed on the vial. The reaction mixture was then stirred at 110 °C in an aluminum block for 18 h at 650 rpm. After cooling to rt, the crude material was filtered through a plug of celite using ethyl acetate. The crude material was then analyzed via NMR to determine the d.r. and percent yield. cis/trans (47:53)

Run 1: (0.026 mmol, 5%)

Run 2: (0.019 mmol, 4%)
Run 3: (0.024 mmol, 5%)
Average: 5% yield

Derivatization of THF Products

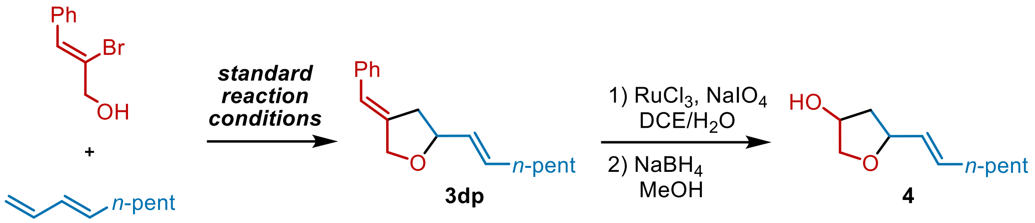

4-((*E*)-benzylidene)-2-((*E*)-hept-1-en-1-yl)tetrahydrofuran (3dp)

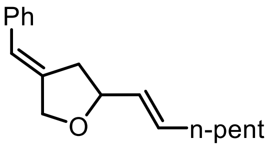

(*Z*)-2-bromo-3-phenylprop-2-en-ol (5.0 mmol, 1.07 g, 1.0 equiv.), N-phenyl urea (68.0 mg, 0.50 mmol, 0.1 equiv.), and (*E*)-nona-1,3-diene (7.5 mmol, 0.931 g, 1.5 equiv.) were weighed out in the above-mentioned order into a 25 mL round bottom flask with a stir bar and capped with a septum. 10.0 mL of prestirred (30 min) stock solution with solvent (2.0 mL DMF, 8.0 mL MIBK) and palladium acetate (56.0 mg, 0.25 mmol, 0.05 equiv.) was then added to the flask and left to stir for several minutes. Then, K_2CO_3 (830 mg, 6.00 mmol, 1.2 equiv.) was added to the reaction mixture and a fresh septum was placed on the flask. The reaction mixture was then stirred at 110 °C in an aluminum block for 18 h. After cooling to room temperature, the crude material filtered through a plug of celite using ethyl acetate. The crude material was then purified as a yellow oil using flash chromatography (silica, 100% hexanes - 95/5 hexanes/EtOAc).

Run 1: (410 mg, 1.60 mmol, 32%)
Run 2: (440 mg, 1.72 mmol, 34%)
Run 3: (424 mg, 1.65 mmol, 33%)
Average: 33% yield

^{1}H NMR (400 Hz) 7.35 – 7.28 (m, 4H), 7.22 – 7.18 (m, 1H), 6.32 – 6.30 (m, 1H), 5.76 (dt, J = 15.4, 6.6 Hz, 1H), 5.54 (dd, J = 15.4, 7.2 Hz, 1H), 4.59 (d, J = 13.2 Hz, 1H), 4.46 – 4.36 (m, 2H), 2.95 (dd, J = 16.3, 6.2 Hz, 1H), 2.55 (ddt, J = 15.7, 7.7, 3.6 Hz, 1H), 2.04 (q, J = 6.8 Hz, 1H), 1.39 – 1.25 (m, 6H), 0.87 (t, J = 6.9 Hz, 3H)

^{13}C NMR (100 Hz) 141.4, 137.7, 134.5, 129.5, 128.5, 128.2, 126.7, 119.7, 81.3, 72.7, 37.8, 32.4, 31.5, 28.8, 22.7, 14.2

HRMS (ESI) m/z: $[M + H]^+$ Calcd for $C_{18}H_{25}O$ 257.1906; Found 257.1901.

(*E*)-5-(hept-1-en-1-yl)tetrahydrofuran-3-ol (4)

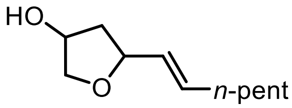

To a flame dried flask equipped with a stir bar was added 4-((*E*)-benzylidene)-2-((*E*)-hept-1-en-1-yl)tetrahydrofuran (500 mg, 1.95 mmol, 1eq) . The compound was dissolved in a 1:1 mixture of DCE:H_2O (40 mL, 0.049 M). Ruthenium trichloride hydrate (20.2 mg, 0.0975 mol, 0.05 equiv.) was added to the reaction mixture. While stirring heavily, sodium periodate (834 mg, 3.90

mmol, 2 equiv.) was added slowly. The reaction mixture was allowed to stir overnight. After consumption of starting material was detected via TLC, the reaction was quenched by added sat. sodium thiosulfate (20 mL). The reaction mixture was then extracted with EtOAc (3 X 40 mL) and solvents were removed via reduced pressure. The crude mixture was then dissolved in a 1:1 mixture of MeOH and DCM (40 mL, 0.049 M). The reaction was cooled to 0 °C via an ice bath. Sodium borohydride (73.8 mg, 1.95 mmol, 1 equiv.) was added slowly and the reaction was stirred for 2 h. The reaction was then quenched with sat. NH_4Cl (20 mL). The reaction mixture was then extracted with EtOAc (3 X 40 mL) and solvents were removed via reduced pressure. The crude material was then purified as a colorless oil using flash chromatography (silica, 100% hexanes - 98/2 hexanes/EtOAc). cis/trans (20:80) determined with help from similar literature products.[35]

Run 1: (259 mg, 1.40 mmol, 72%)
Run 2: (271 mg, 1.47 mmol, 76%)
Run 3: (263 mg, 1.42 mmol, 73%)
Average: 74% yield

^{1}H NMR (400 MHz, $CDCl_3$) trans δ 5.75 – 5.65 (m, 1H), 5.57 (ddt, J = 15.3, 7.1, 1.3 Hz, 1H), 4.52 – 4.41 (m, 1H), 4.26 (q, J = 7.2 Hz, 1H), 3.84 (dt, J = 9.8, 1.6 Hz, 1H), 3.75 – 3.67 (m, 1H), 2.34 (ddd, J = 13.5, 7.8, 6.4 Hz, 1H), 2.21 (s, 1H), 2.01 (m, 2H), 1.70 – 1.63 (m, 1H), 1.39 – 1.22 (m, 6H), 0.86 (t, J = 6.9 Hz, 3H)

^{1}H NMR (400 MHz, $CDCl_3$) cis δ 5.75 – 5.65 (m, 1H), 5.41 (ddt, J = 15.2, 7.4, 1.5 Hz, 1H), 4.52 – 4.41 (m, 2H), 4.02 (dd, J = 9.8, 4.4 Hz, 1H), 3.75 – 3.67 (m, 1H), 2.34 (ddd, J = 13.5, 7.8, 6.4 Hz, 1H), 2.27 (s, 1H), 2.01 (m, 2H), 1.77 – 1.70 (m, 1H), 1.39 – 1.22 (m, 6H), 0.86 (t, J = 6.9 Hz, 3H)

^{13}C NMR (100 MHz, $CDCl_3$) trans δ 133.6, 130.8, 79.8, 75.6, 72.9, 41.9, 32.2, 31.5, 28.8, 22.6, 14.1

^{13}C NMR (100 MHz, $CDCl_3$) cis δ 134.1, 129.7, 79.0, 75.6, 72.8, 42.3, 32.3, 31.5, 28.8, 22.6, 14.1

HRMS (ESI) m/z: $[M + H]^+$ Calcd $C_{11}H_{21}O_2$ 207.1362; Found 207.1359.

NMR Spectra of New Compounds

(*Z*)-2-bromo-1,3-diphenylprop-2-en-1-ol (1b)

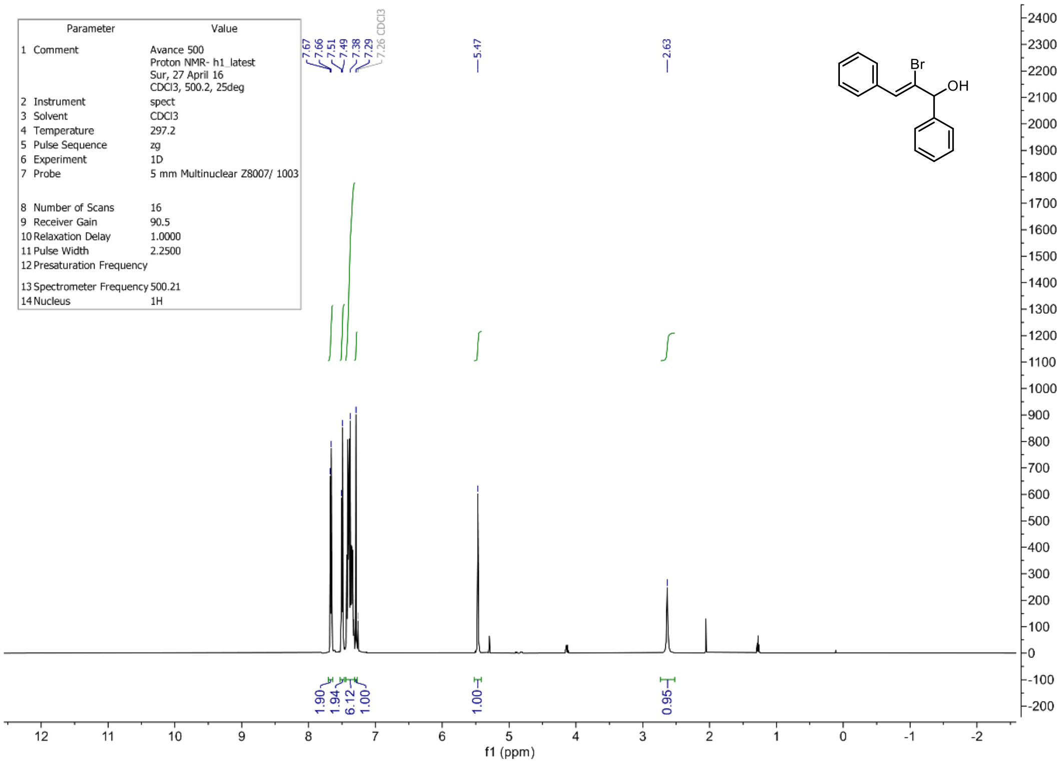

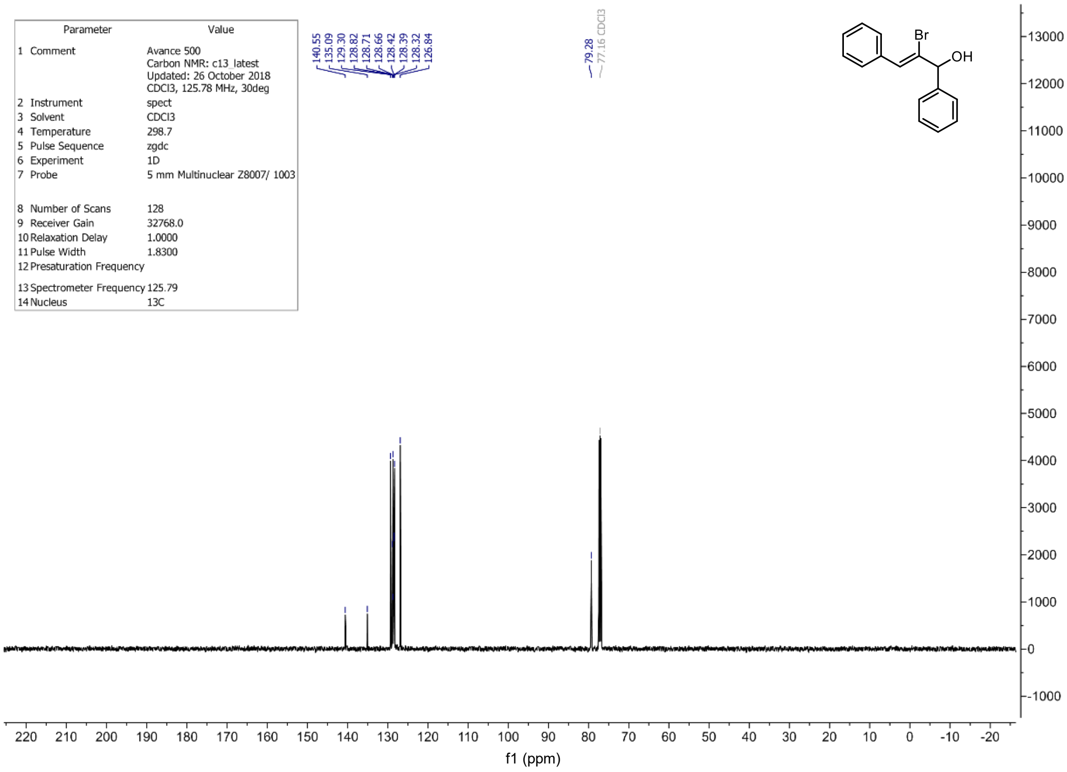

(*Z*)-2-bromo-4,4-dimethyl-1-phenylpent-1-en-3-ol (1c)

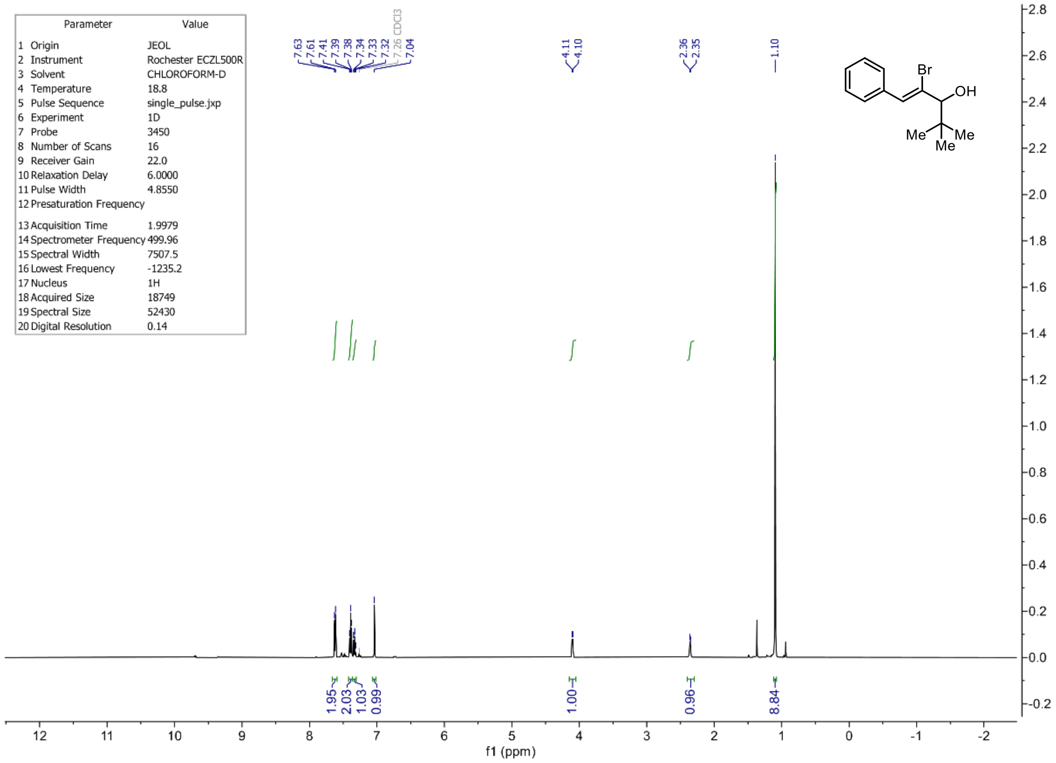

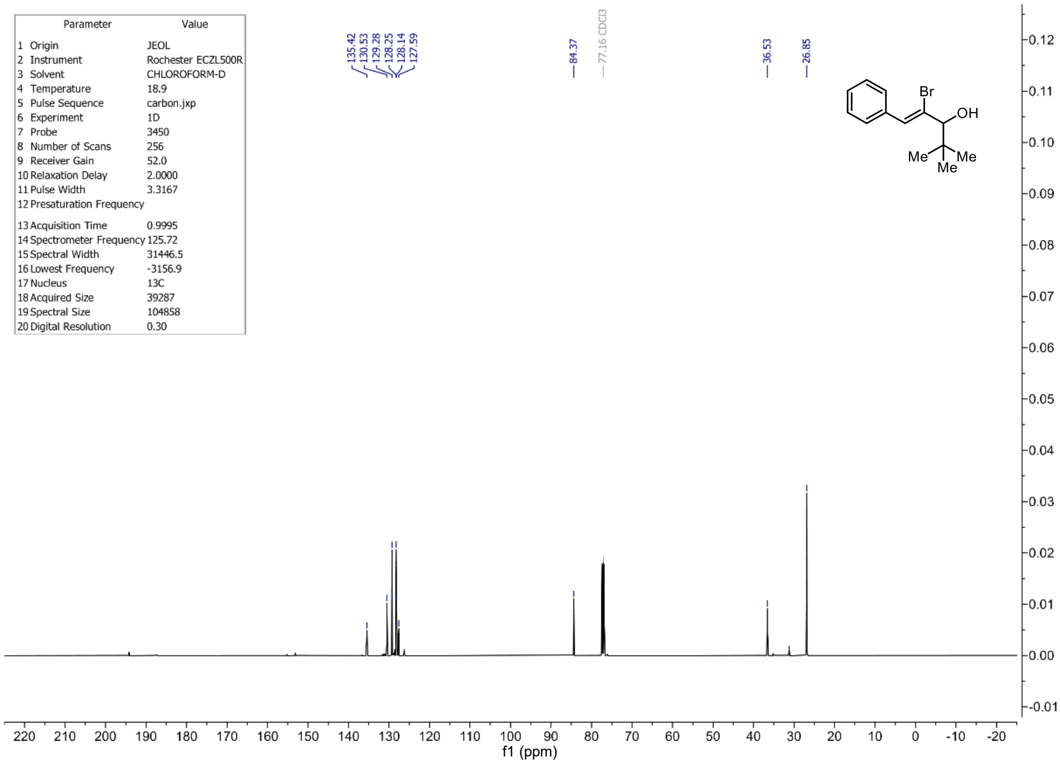

(*Z*)-3-bromo-2-methyl-4-phenylbut-3-en-2-ol (1e)

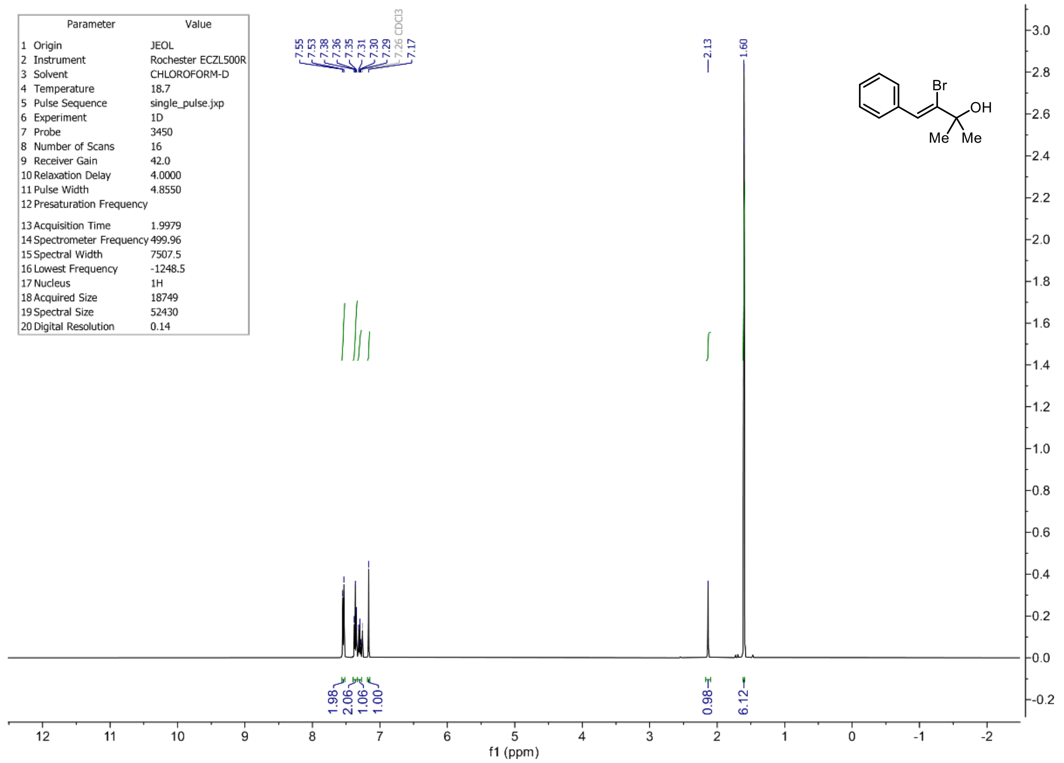

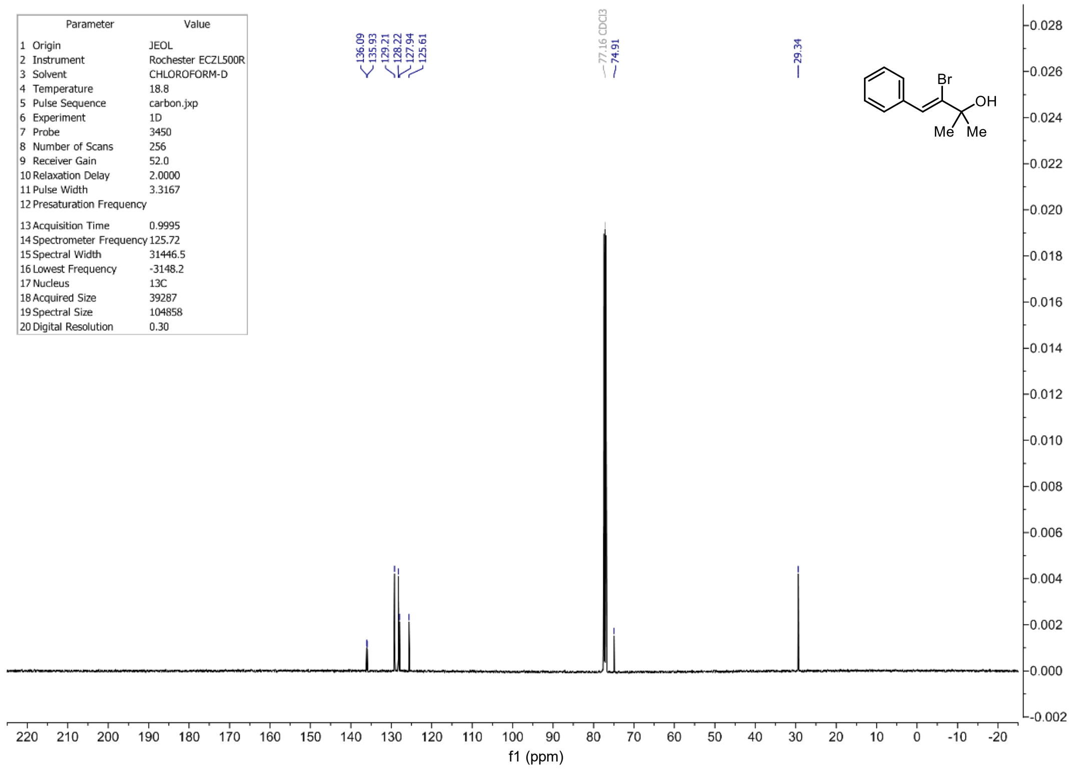

3-bromobut-3-en-2-ol (1g)

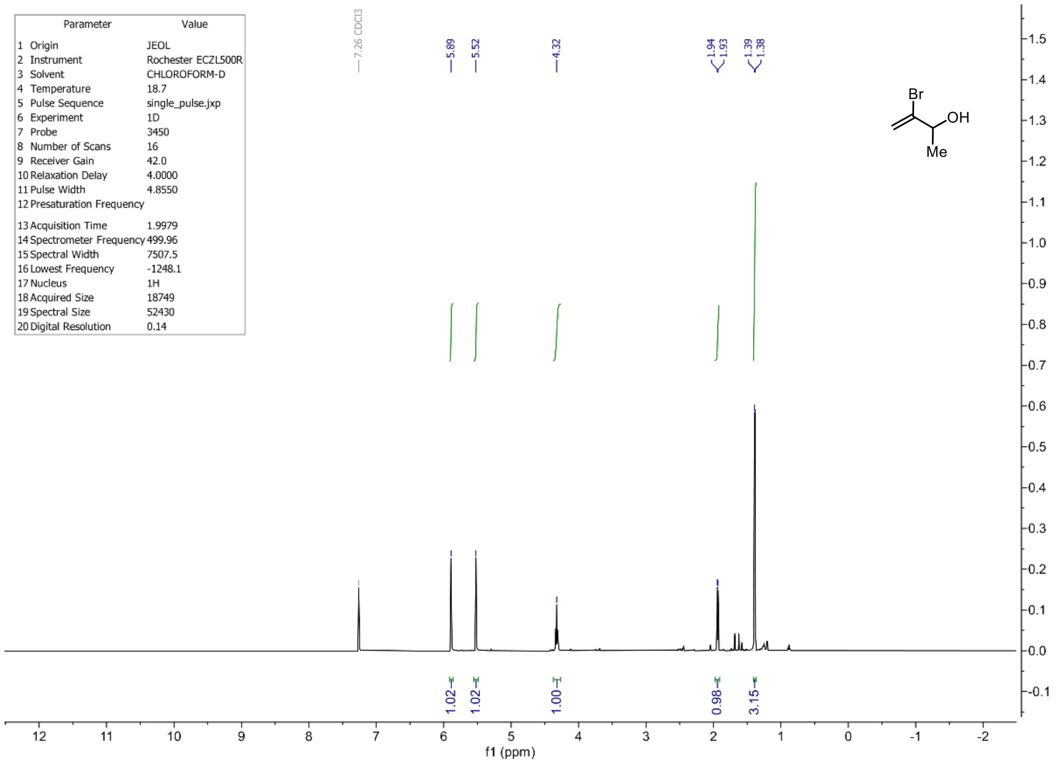

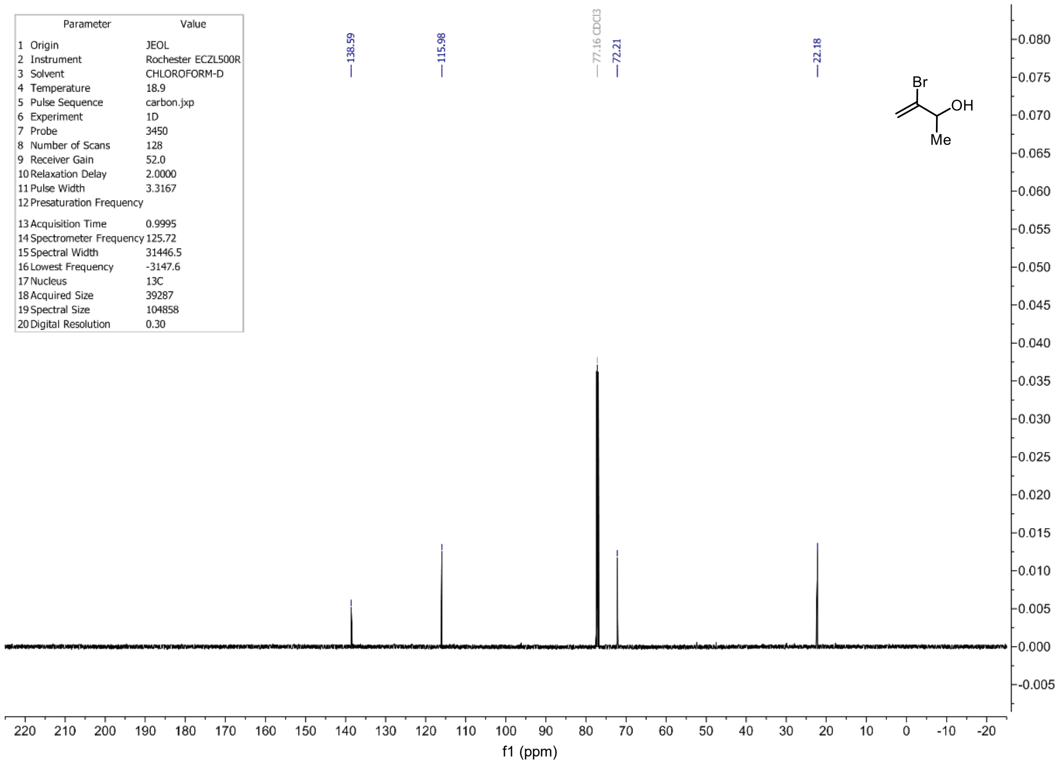

(*Z*)-1-(benzo[d][1,3]dioxol-5-yl)-2-bromo-3-phenylprop-2-en-1-ol (1h)

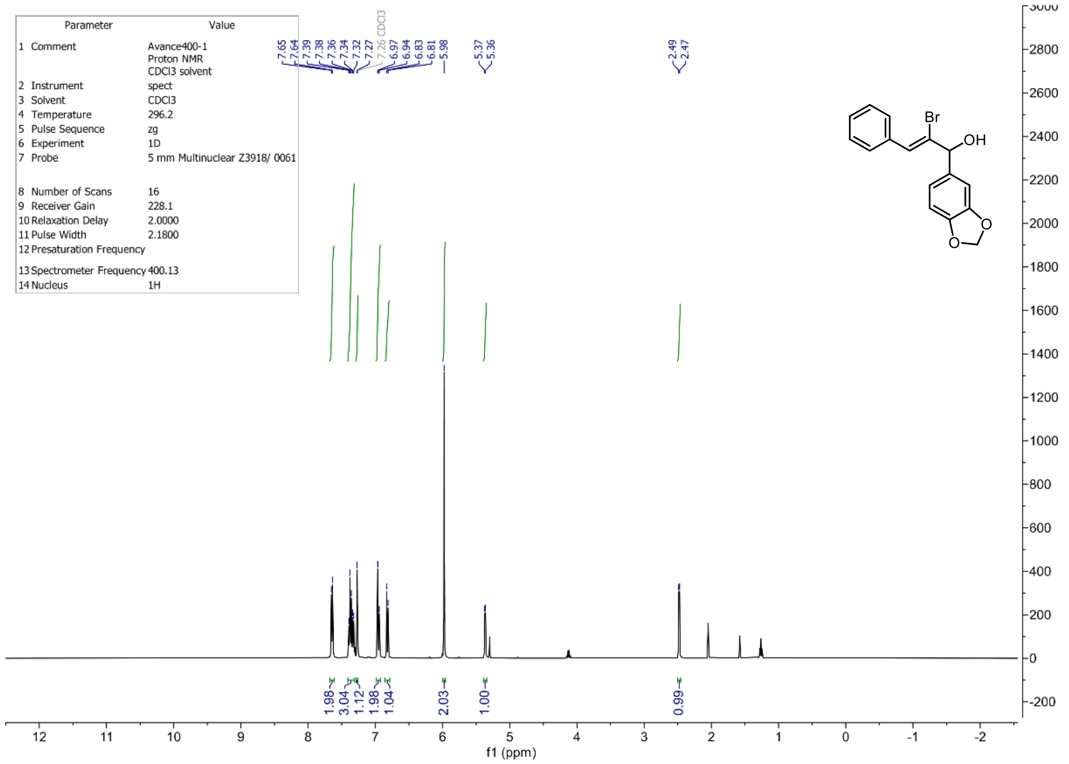

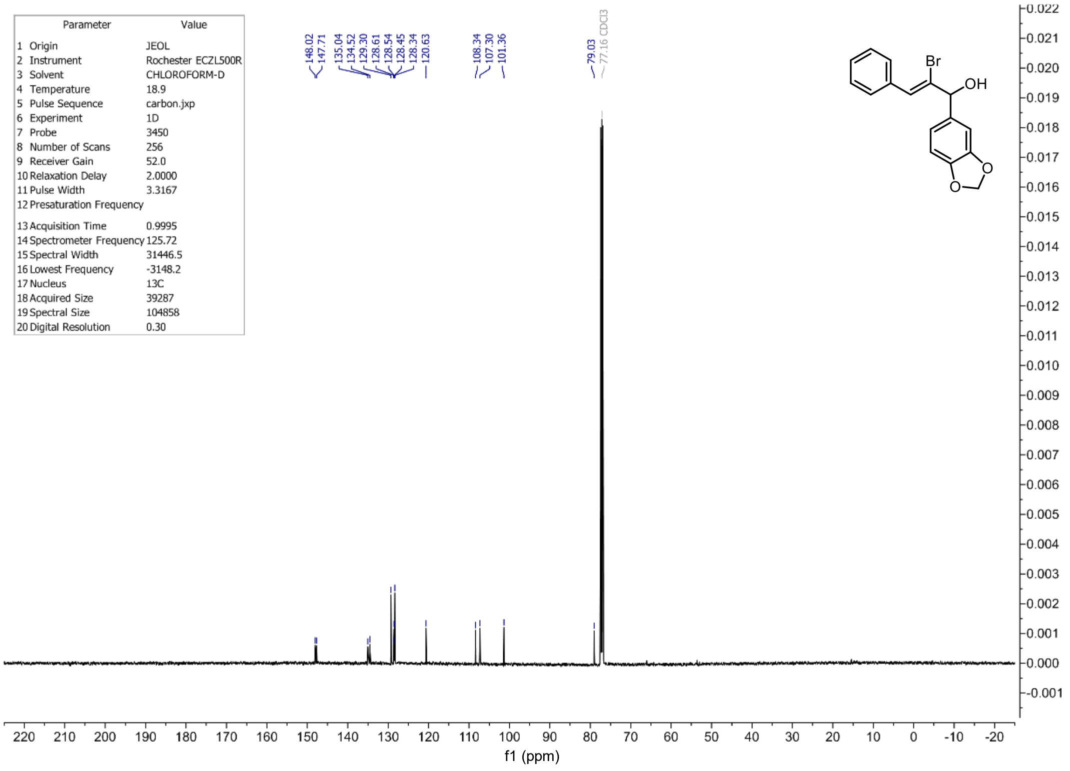

(*Z*)-2-bromo-1-cyclopropyl-3-phenylprop-2-en-1-ol (1i)

Parameter	Value
1 Origin	JEOL
2 Instrument	Rochester ECZL500R
3 Solvent	DMSO-D6
4 Temperature	18.7
5 Pulse Sequence	single_pulse.jxp
6 Experiment	1D
7 Probe	3450
8 Number of Scans	16
9 Receiver Gain	42.0
10 Relaxation Delay	4.0000
11 Pulse Width	4.8700
12 Presaturation Frequency	
13 Acquisition Time	1.9979
14 Spectrometer Frequency	499.96
15 Spectral Width	7507.5
16 Lowest Frequency	-1234.8
17 Nucleus	1H
18 Acquired Size	18749
19 Spectral Size	52430
20 Digital Resolution	0.14

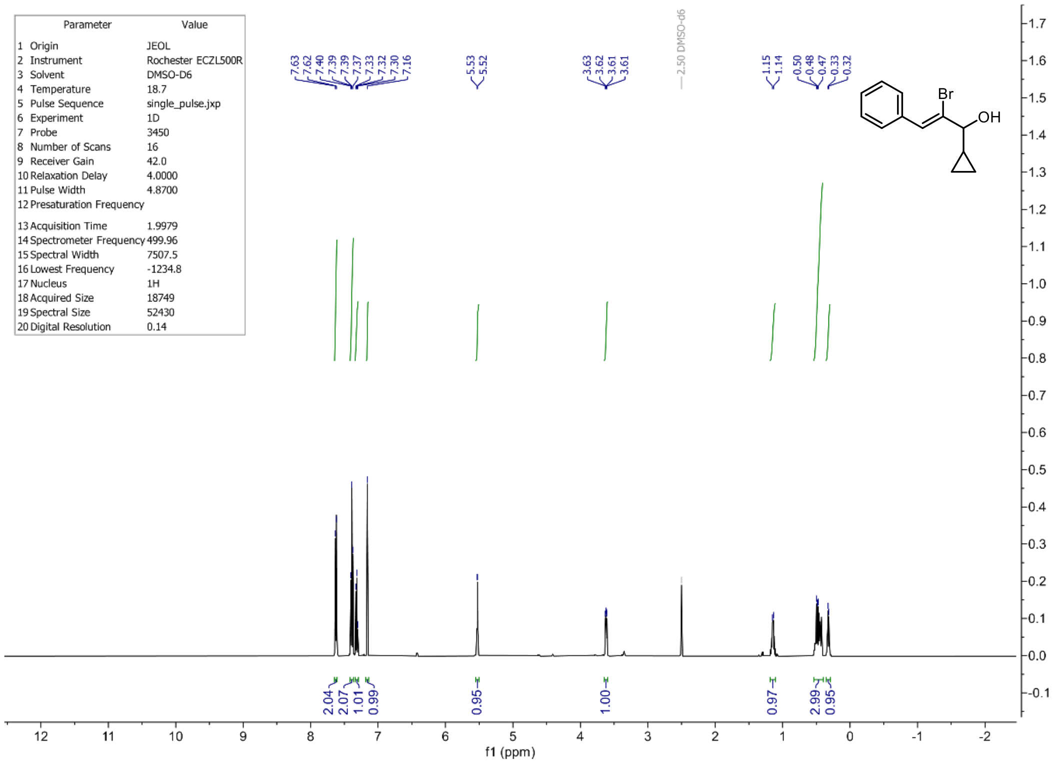

Parameter	Value
1 Origin	JEOL
2 Instrument	Rochester ECZL500R
3 Solvent	DMSO-D6
4 Temperature	18.9
5 Pulse Sequence	carbon.jxp
6 Experiment	1D
7 Probe	3450
8 Number of Scans	128
9 Receiver Gain	52.0
10 Relaxation Delay	2.0000
11 Pulse Width	3.2467
12 Presaturation Frequency	
13 Acquisition Time	0.9995
14 Spectrometer Frequency	125.72
15 Spectral Width	31446.5
16 Lowest Frequency	-3214.4
17 Nucleus	13C
18 Acquired Size	39287
19 Spectral Size	104858
20 Digital Resolution	0.30

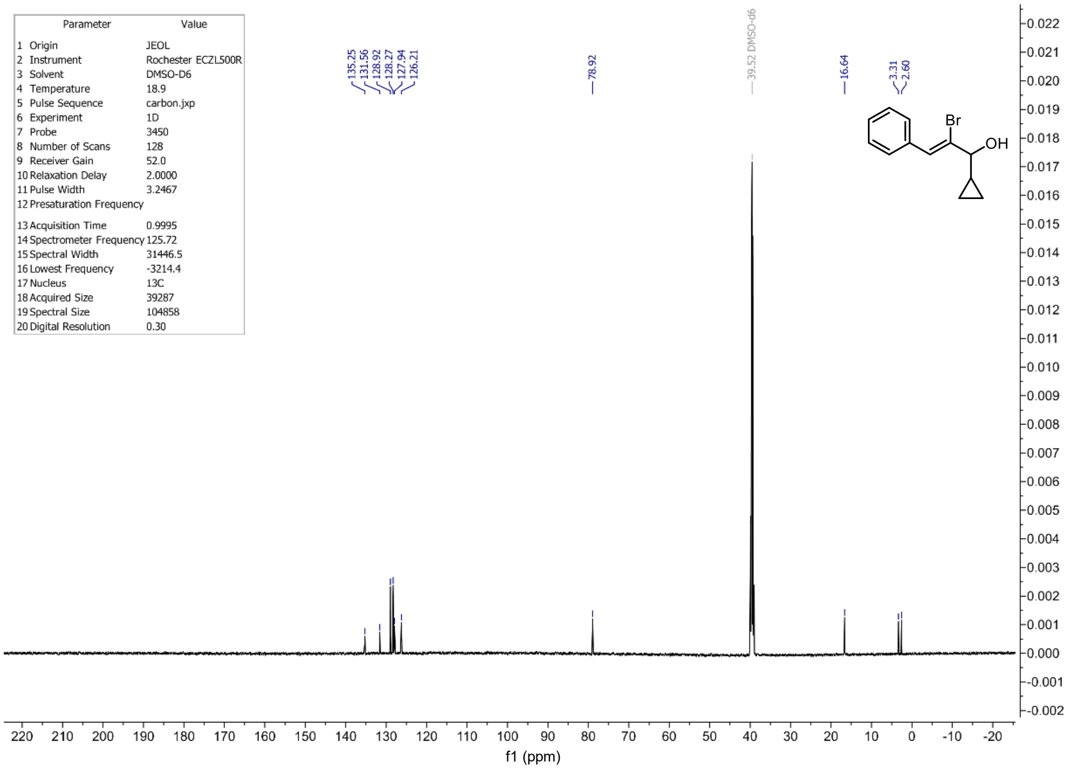

(*Z*)-2-bromo-1-phenylpenta-1,4-dien-3-ol (1j)

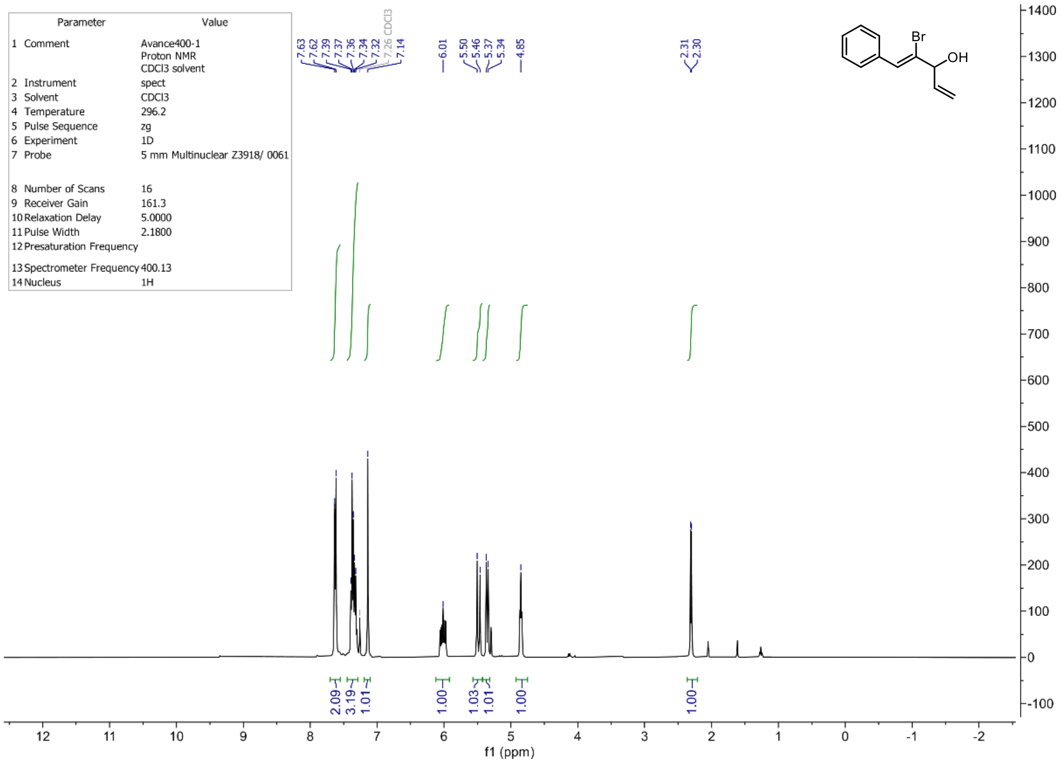

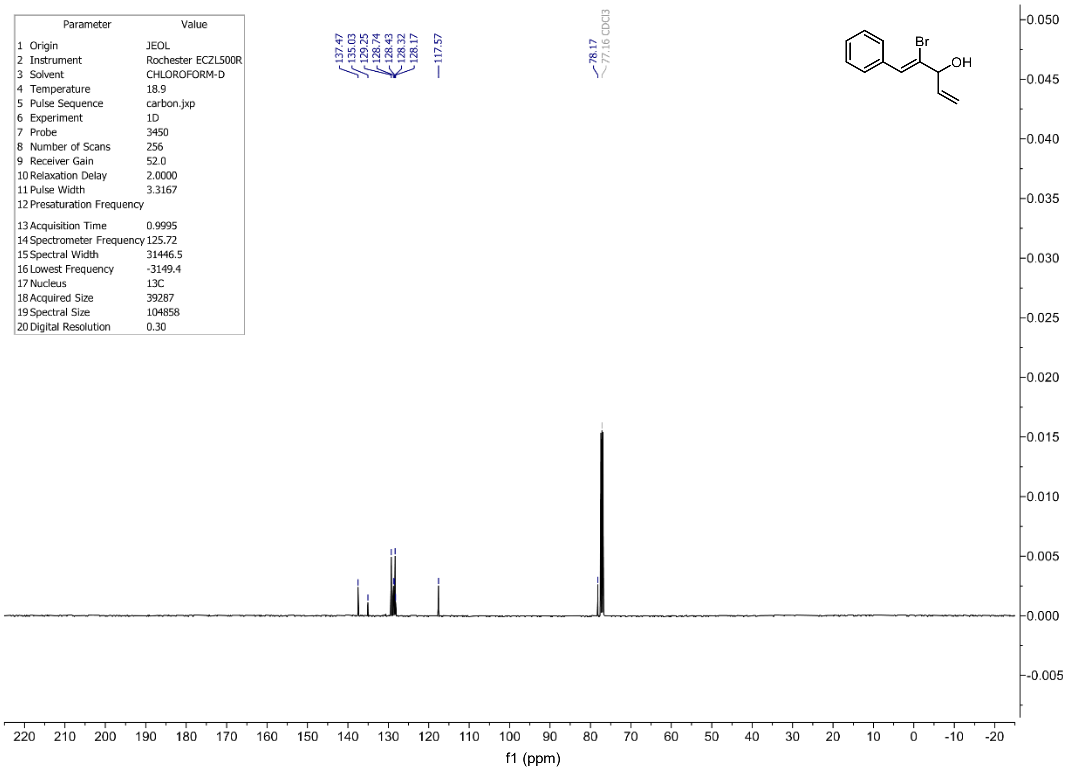

(*Z*)-3-bromo-6-phenylhex-3-en-2-ol (1l)

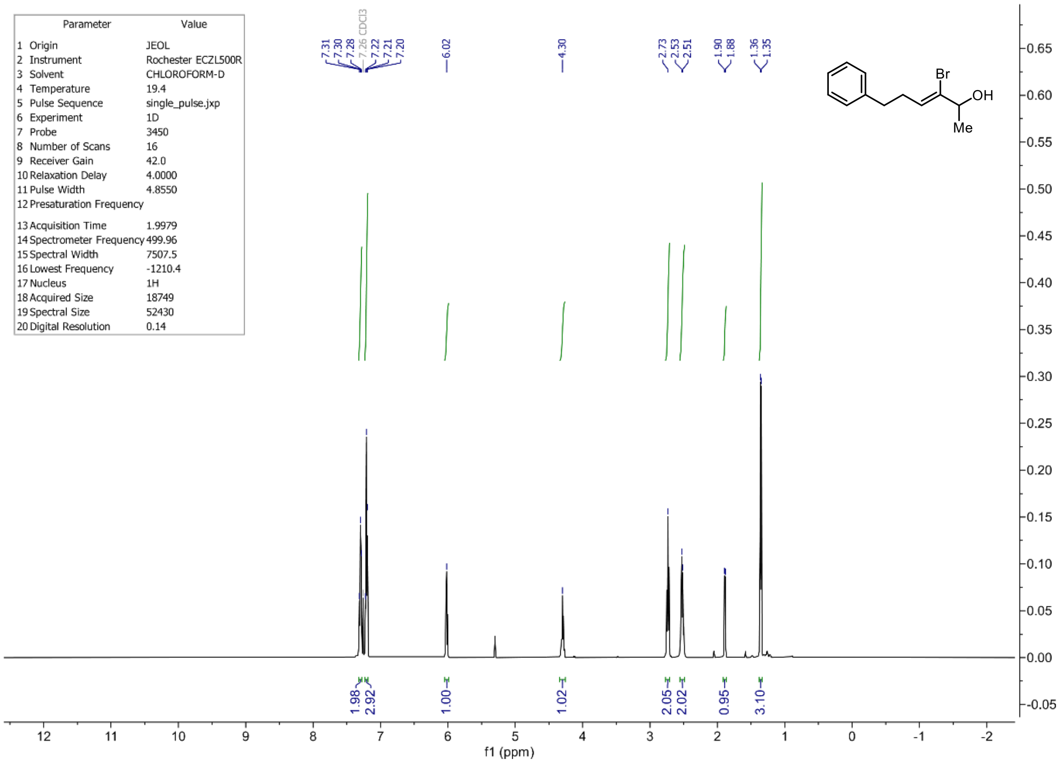

Parameter	Value
1 Origin	JEOL
2 Instrument	Rochester ECZL500R
3 Solvent	CHLOROFORM-D
4 Temperature	19.4
5 Pulse Sequence	single_pulse.jxp
6 Experiment	1D
7 Probe	3450
8 Number of Scans	16
9 Receiver Gain	42.0
10 Relaxation Delay	4.0000
11 Pulse Width	4.8550
12 Presaturation Frequency	
13 Acquisition Time	1.9979
14 Spectrometer Frequency	499.96
15 Spectral Width	7507.5
16 Lowest Frequency	-1210.4
17 Nucleus	1H
18 Acquired Size	18749
19 Spectral Size	52430
20 Digital Resolution	0.14

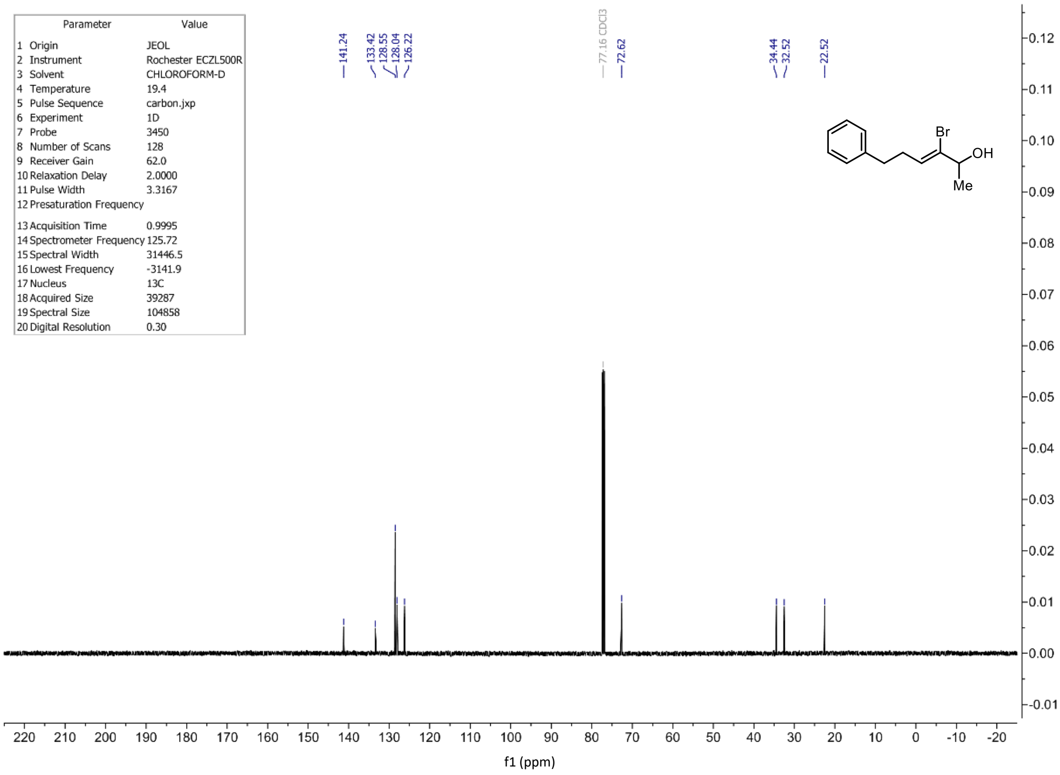

Parameter	Value
1 Origin	JEOL
2 Instrument	Rochester ECZL500R
3 Solvent	CHLOROFORM-D
4 Temperature	19.4
5 Pulse Sequence	carbon.jxp
6 Experiment	1D
7 Probe	3450
8 Number of Scans	128
9 Receiver Gain	62.0
10 Relaxation Delay	2.0000
11 Pulse Width	3.3167
12 Presaturation Frequency	
13 Acquisition Time	0.9995
14 Spectrometer Frequency	125.72
15 Spectral Width	31446.5
16 Lowest Frequency	-3141.9
17 Nucleus	13C
18 Acquired Size	39287
19 Spectral Size	104858
20 Digital Resolution	0.30

(*Z*)-3-bromo-4-cyclohexylbut-3-en-2-ol (1m)

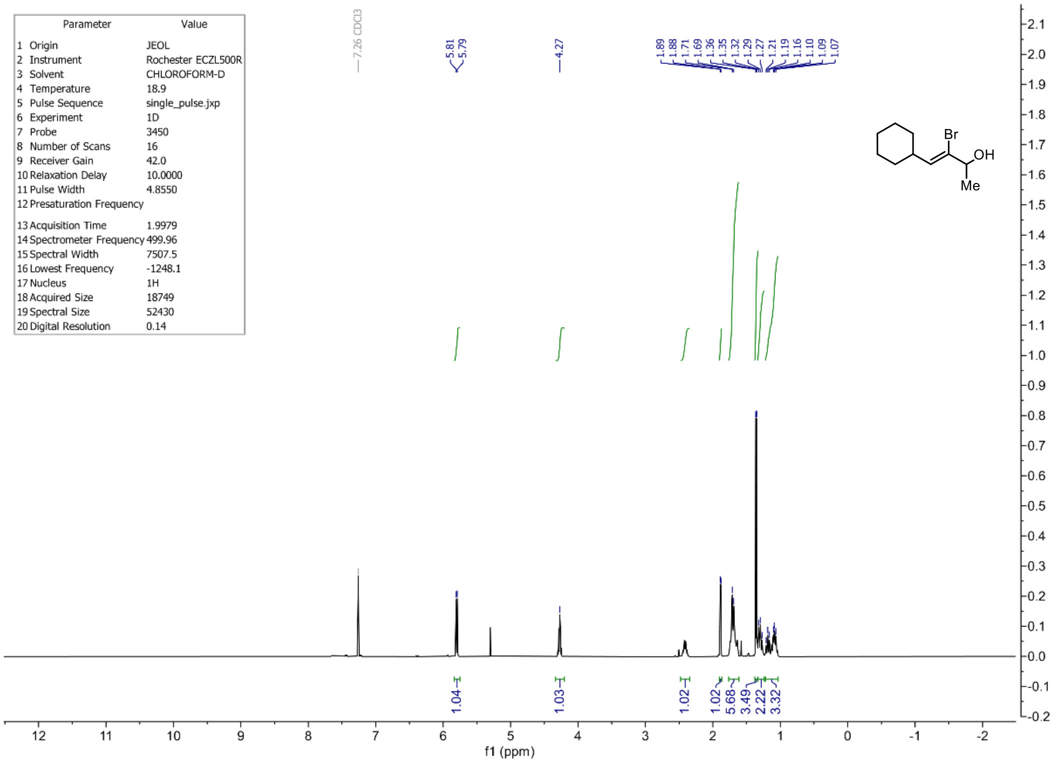

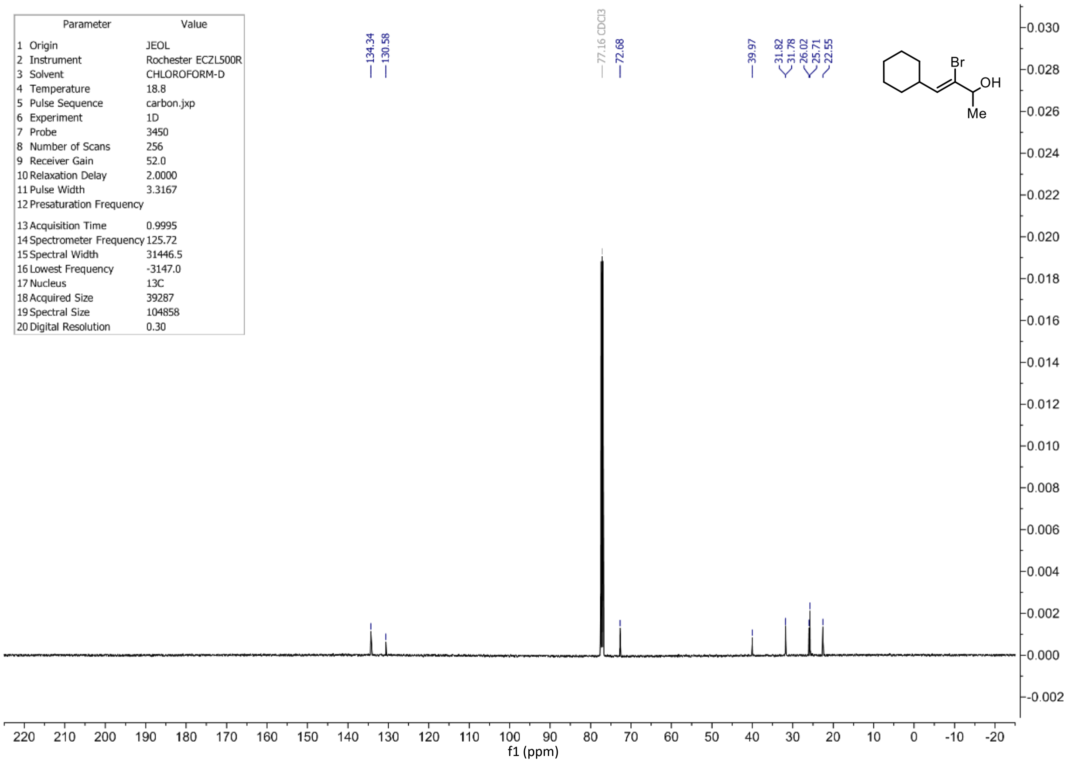

(*Z*)-3-bromo-4-(4-methoxyphenyl)but-3-en-2-ol (1n)

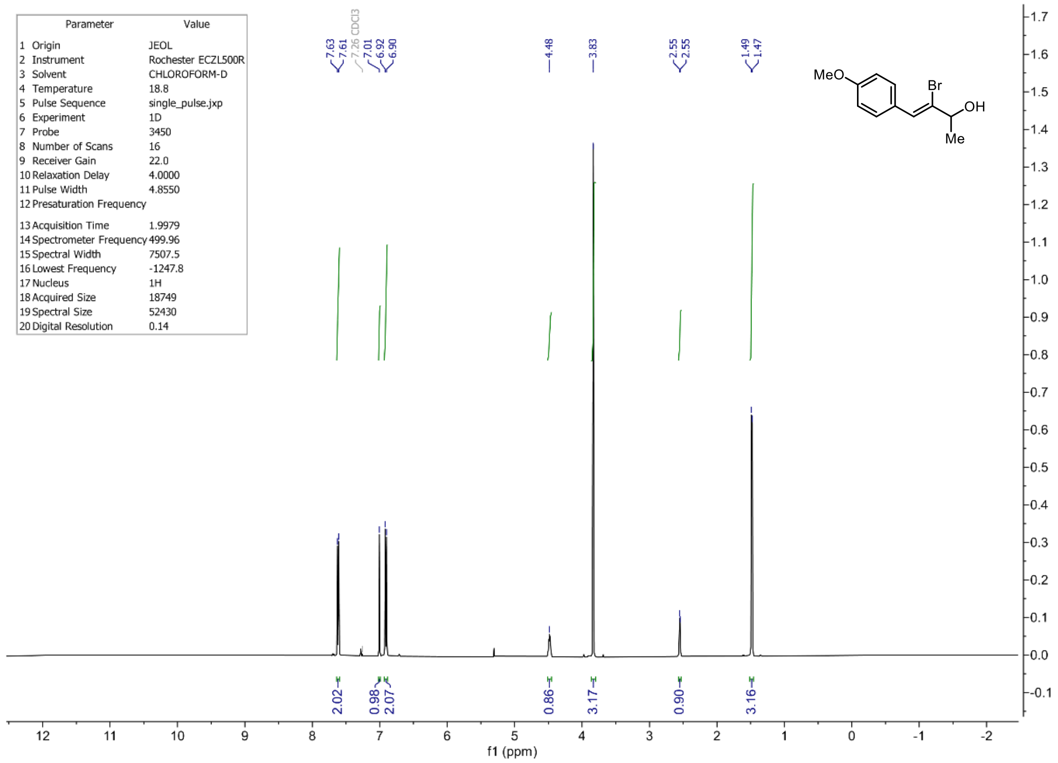

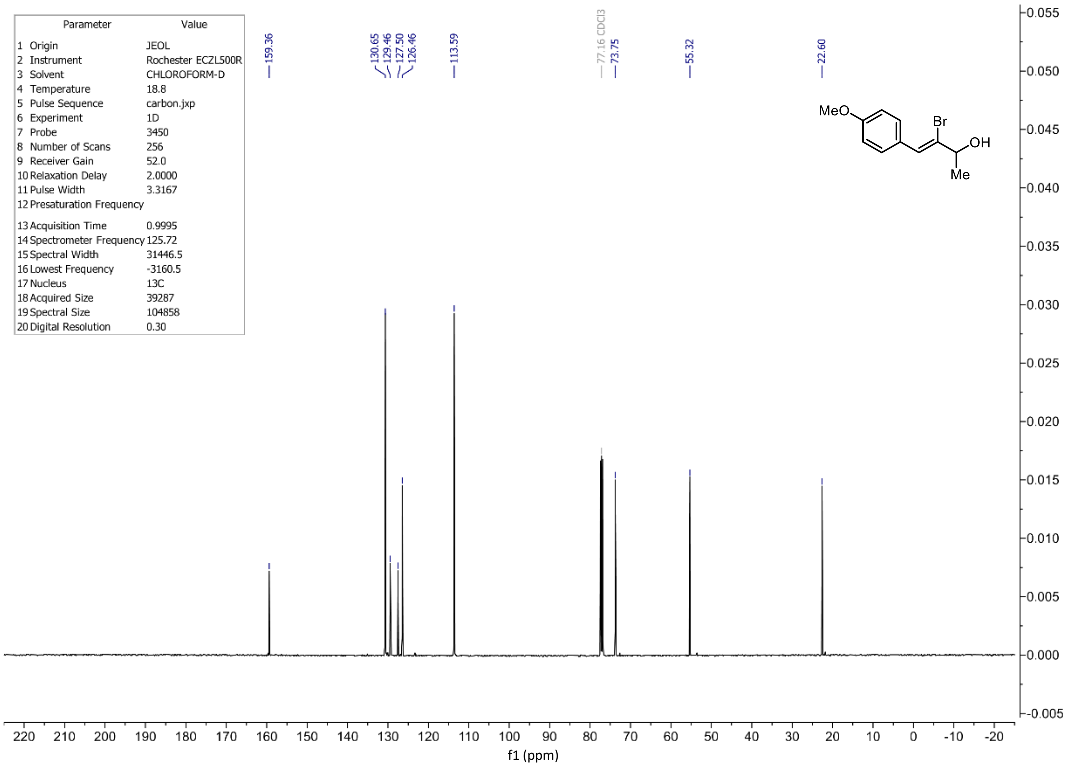

(*Z*)-3-bromo-4-(4-(trifluoromethyl)phenyl)but-3-en-2-ol (1o)

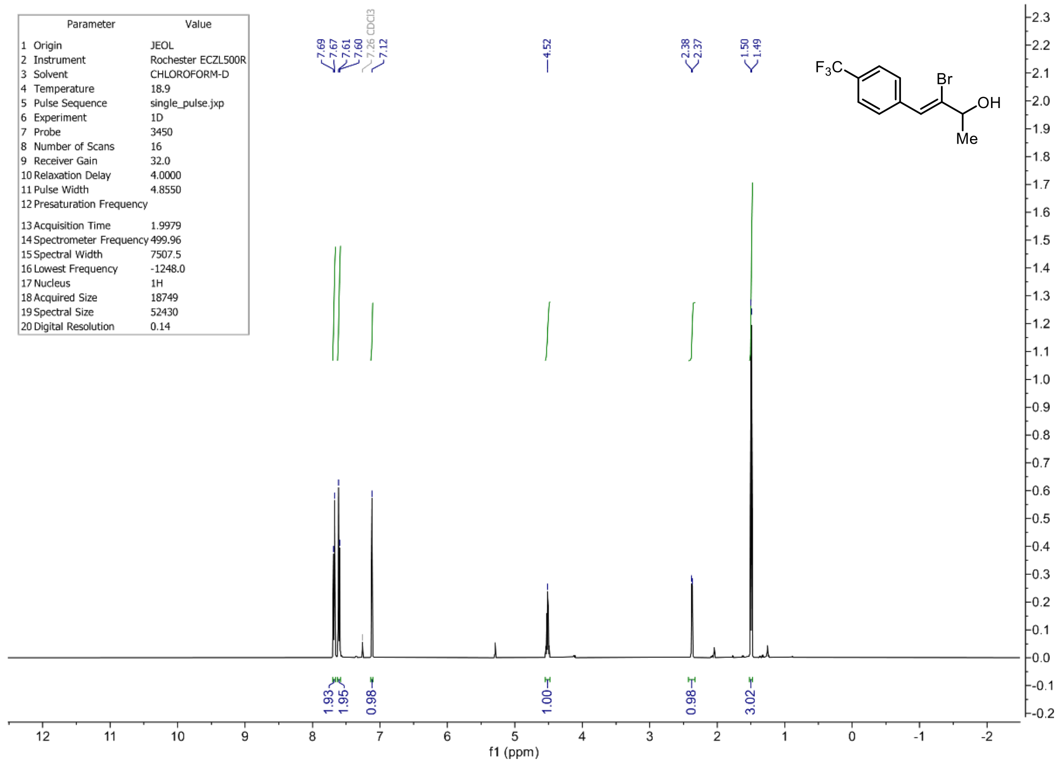

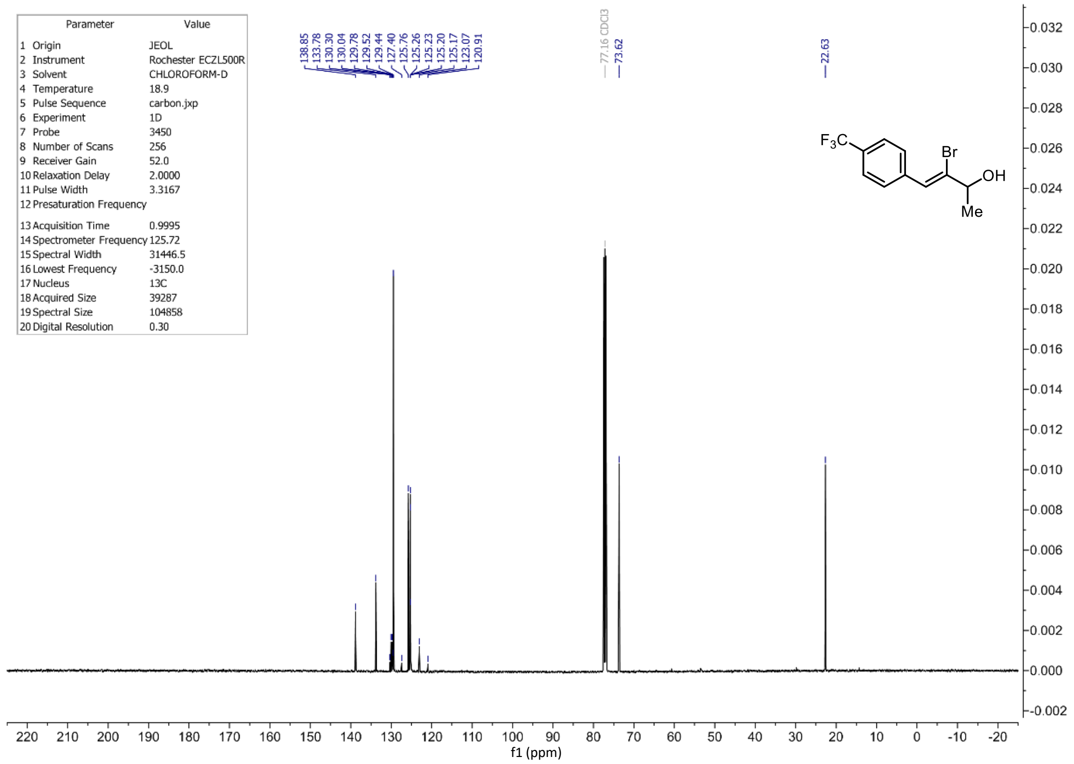

Parameter		Value
1	Origin	JEOL
2	Instrument	Rochester ECZL500R
3	Solvent	CHLOROFORM-D
4	Temperature	18.9
5	Pulse Sequence	single_pulse.jxp
6	Experiment	1D
7	Probe	3450
8	Number of Scans	16
9	Receiver Gain	42.0
10	Relaxation Delay	4.0000
11	Pulse Width	5.6350
12	Presaturation Frequency	
13	Acquisition Time	1.9993
14	Spectrometer Frequency	470.43
15	Spectral Width	94117.6
16	Lowest Frequency	-94102.1
17	Nucleus	Fluorine19
18	Acquired Size	235217
19	Spectral Size	419432
20	Digital Resolution	0.22

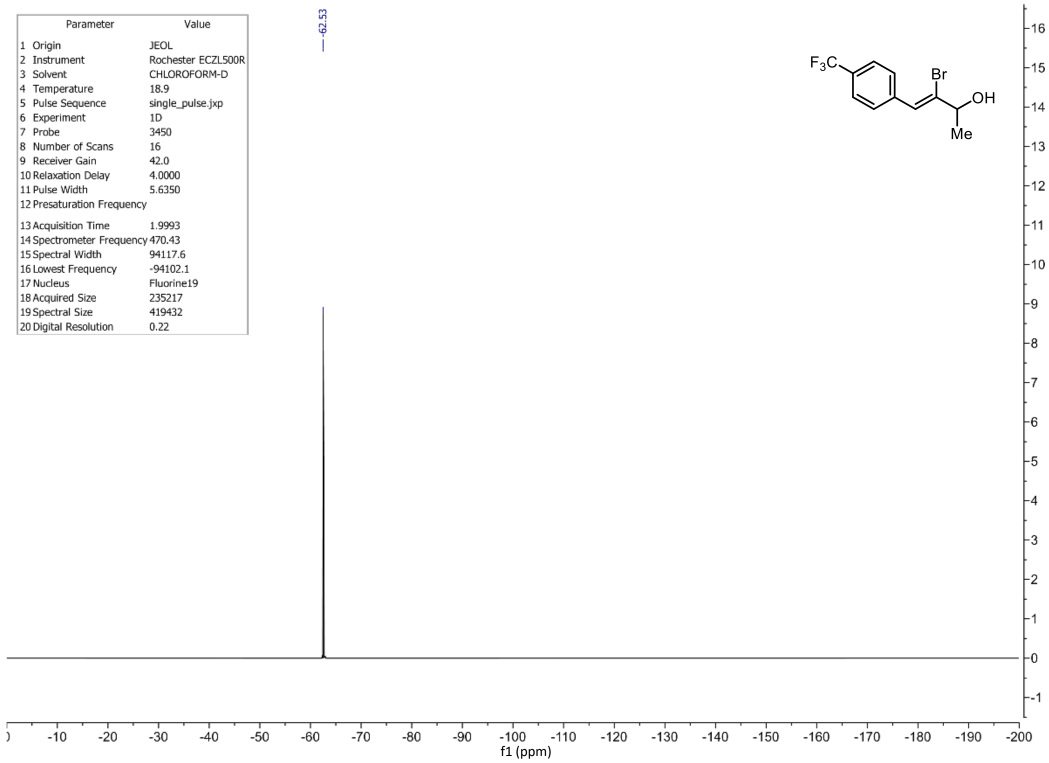

(*Z*)-3-bromo-4-(2,3-dihydrobenzofuran-5-yl)but-3-en-2-ol (1p)

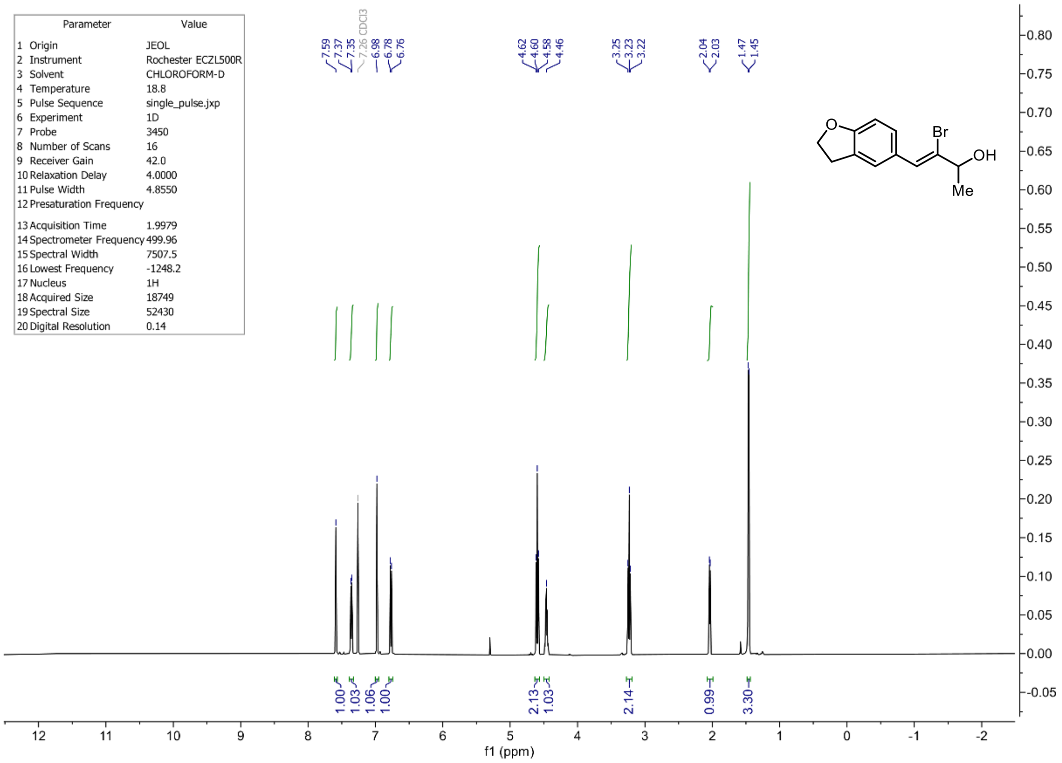

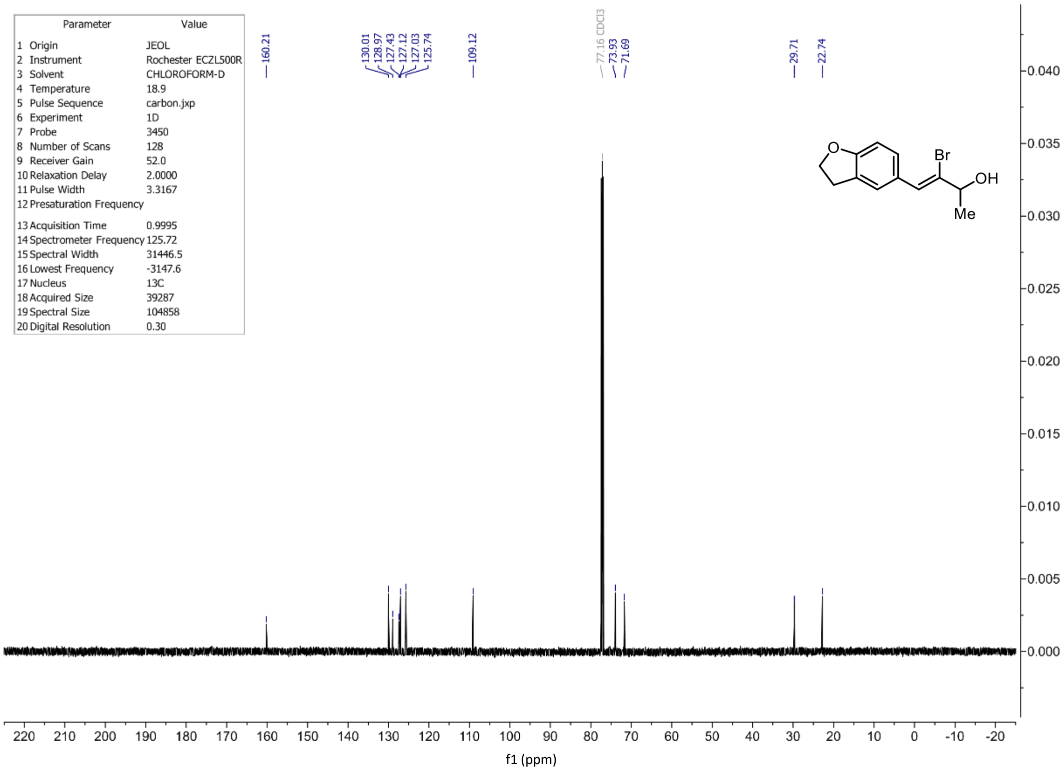

(*Z*)-3-bromo-4-(pyridin-3-yl)but-3-en-2-ol (1q)

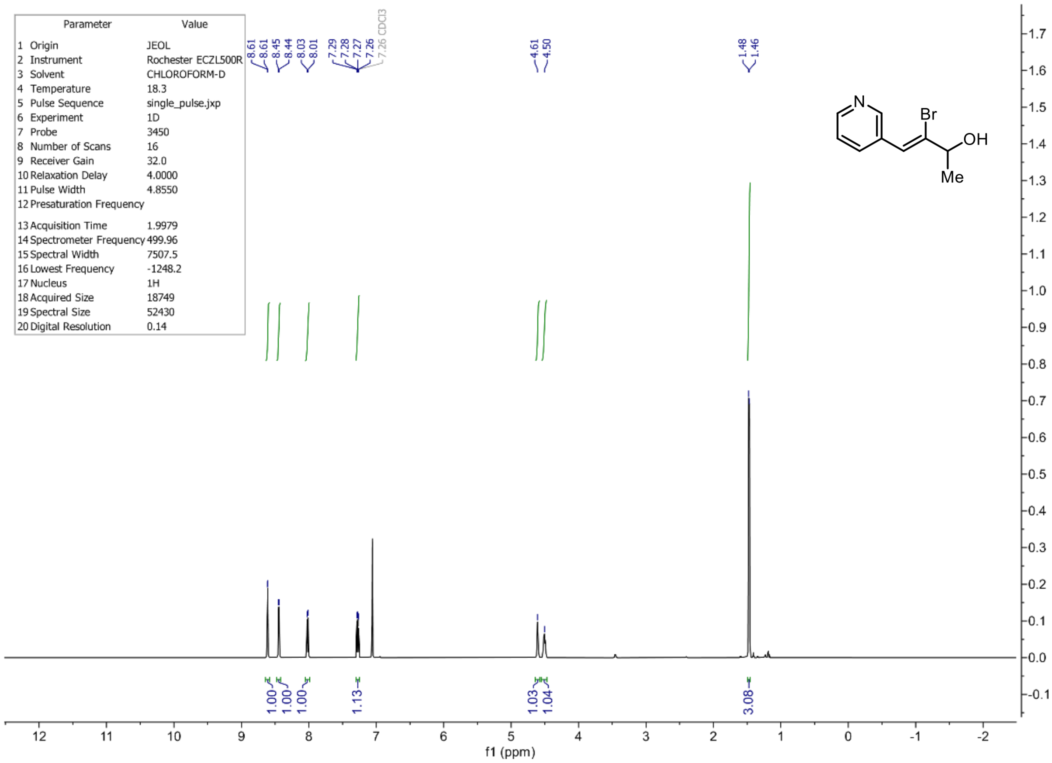

Parameter	Value
1 Origin	JEOL
2 Instrument	Rochester ECZL500R
3 Solvent	CHLOROFORM-D
4 Temperature	18.3
5 Pulse Sequence	single_pulse.jxp
6 Experiment	1D
7 Probe	3450
8 Number of Scans	16
9 Receiver Gain	32.0
10 Relaxation Delay	4.0000
11 Pulse Width	4.8550
12 Presaturation Frequency	
13 Acquisition Time	1.9979
14 Spectrometer Frequency	499.96
15 Spectral Width	7507.5
16 Lowest Frequency	-1248.2
17 Nucleus	1H
18 Acquired Size	18749
19 Spectral Size	52430
20 Digital Resolution	0.14

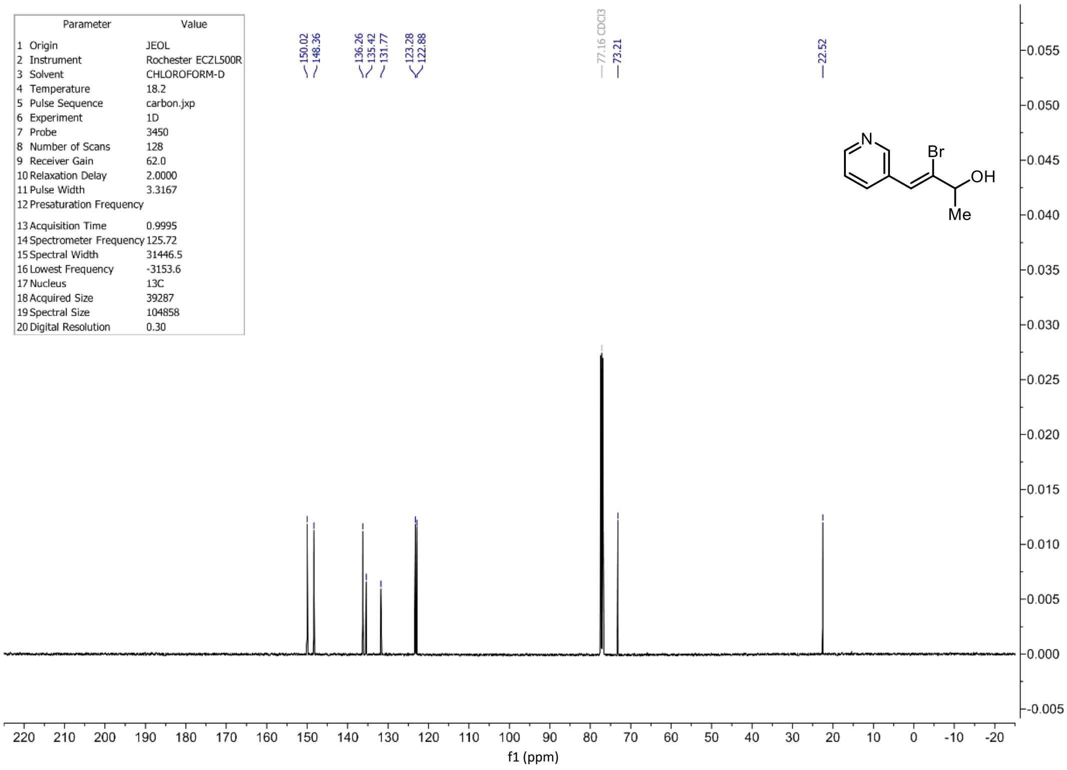

Parameter	Value
1 Origin	JEOL
2 Instrument	Rochester ECZL500R
3 Solvent	CHLOROFORM-D
4 Temperature	18.2
5 Pulse Sequence	carbon.jxp
6 Experiment	1D
7 Probe	3450
8 Number of Scans	128
9 Receiver Gain	62.0
10 Relaxation Delay	2.0000
11 Pulse Width	3.3167
12 Presaturation Frequency	
13 Acquisition Time	0.9995
14 Spectrometer Frequency	125.72
15 Spectral Width	31446.5
16 Lowest Frequency	-3153.6
17 Nucleus	13C
18 Acquired Size	39287
19 Spectral Size	104858
20 Digital Resolution	0.30

3-((*E*)-benzylidene)-2-methyl-5-((*E*)-styryl)tetrahydrofuran (3aa)

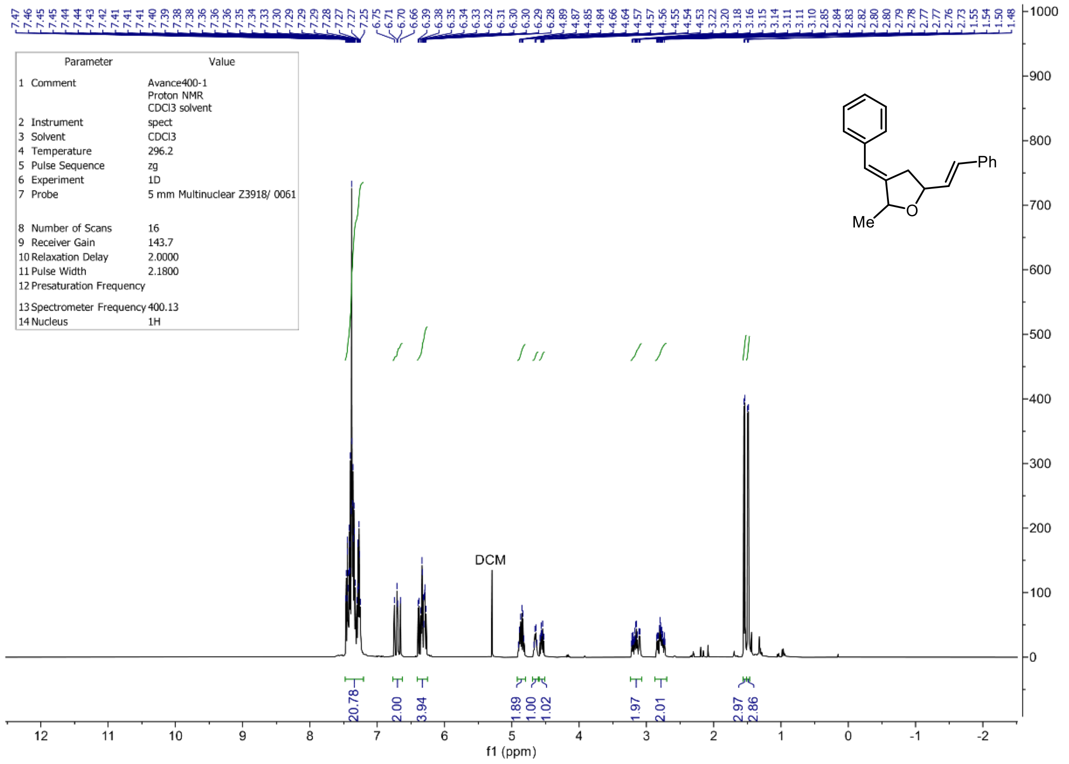

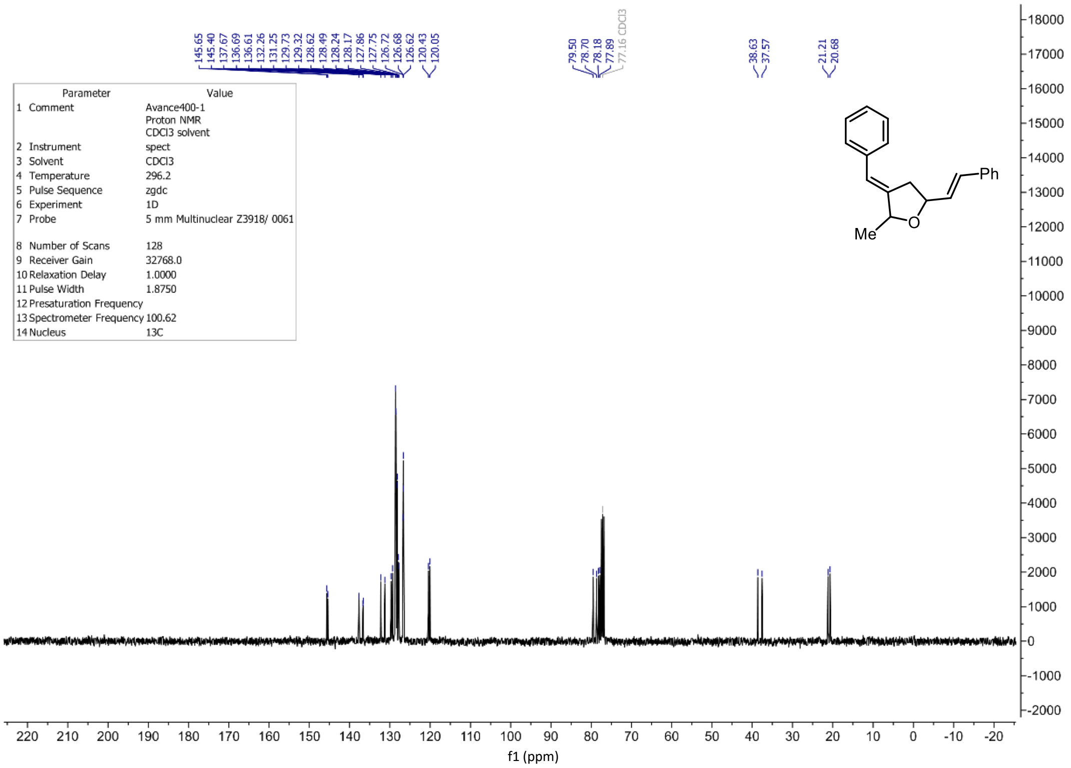

3-((*E*)-benzylidene)-2-methyl-5-((*E*)-styryl)tetrahydrofuran (3aa) 93:7 (cis:trans) mixture

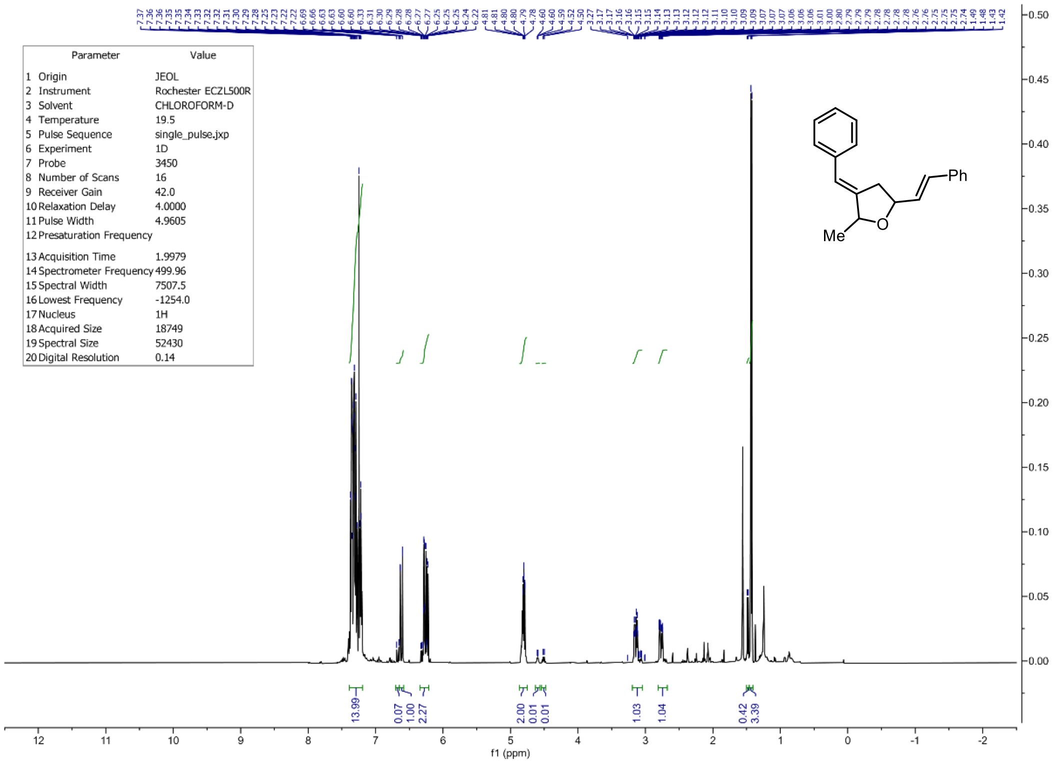

Parameter	Value
1 Origin	JEOL
2 Instrument	Rochester ECZL500R
3 Solvent	CHLOROFORM-D
4 Temperature	19.5
5 Pulse Sequence	single_pulse.jxp
6 Experiment	1D
7 Probe	3450
8 Number of Scans	16
9 Receiver Gain	42.0
10 Relaxation Delay	4.0000
11 Pulse Width	4.9605
12 Presaturation Frequency	
13 Acquisition Time	1.9979
14 Spectrometer Frequency	499.96
15 Spectral Width	7507.5
16 Lowest Frequency	-1254.0
17 Nucleus	1H
18 Acquired Size	18749
19 Spectral Size	52430
20 Digital Resolution	0.14

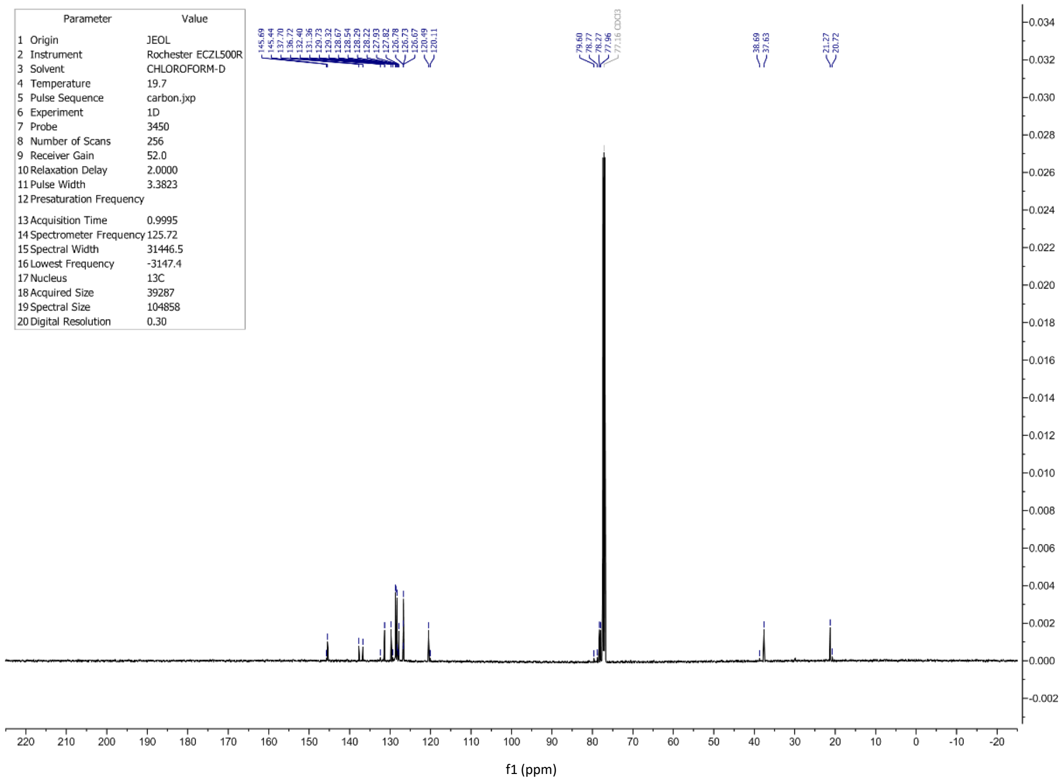

Parameter	Value
1 Origin	JEOL
2 Instrument	Rochester ECZL500R
3 Solvent	CHLOROFORM-D
4 Temperature	19.7
5 Pulse Sequence	carbon.jxp
6 Experiment	1D
7 Probe	3450
8 Number of Scans	256
9 Receiver Gain	52.0
10 Relaxation Delay	2.0000
11 Pulse Width	3.3823
12 Presaturation Frequency	
13 Acquisition Time	0.9995
14 Spectrometer Frequency	125.72
15 Spectral Width	31446.5
16 Lowest Frequency	-3147.4
17 Nucleus	13C
18 Acquired Size	39287
19 Spectral Size	104858
20 Digital Resolution	0.30

3-((*E*)-benzylidene)-2-phenyl-5-((*E*)-styryl)tetrahydrofuran (3ba)

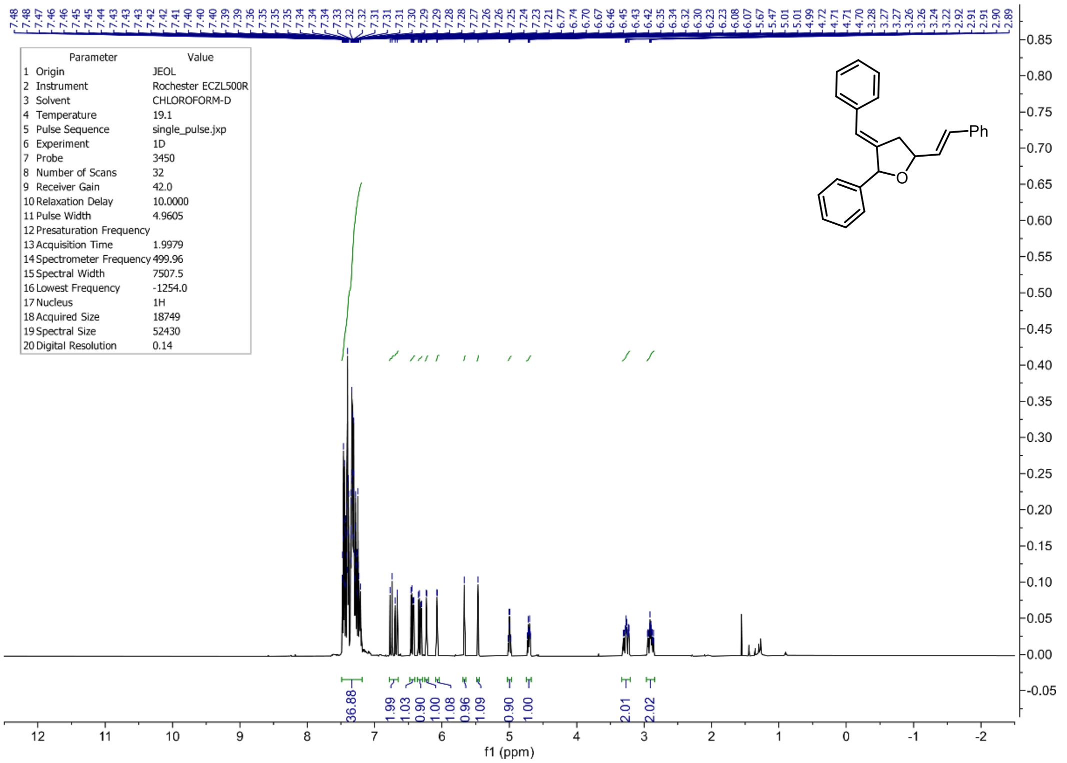

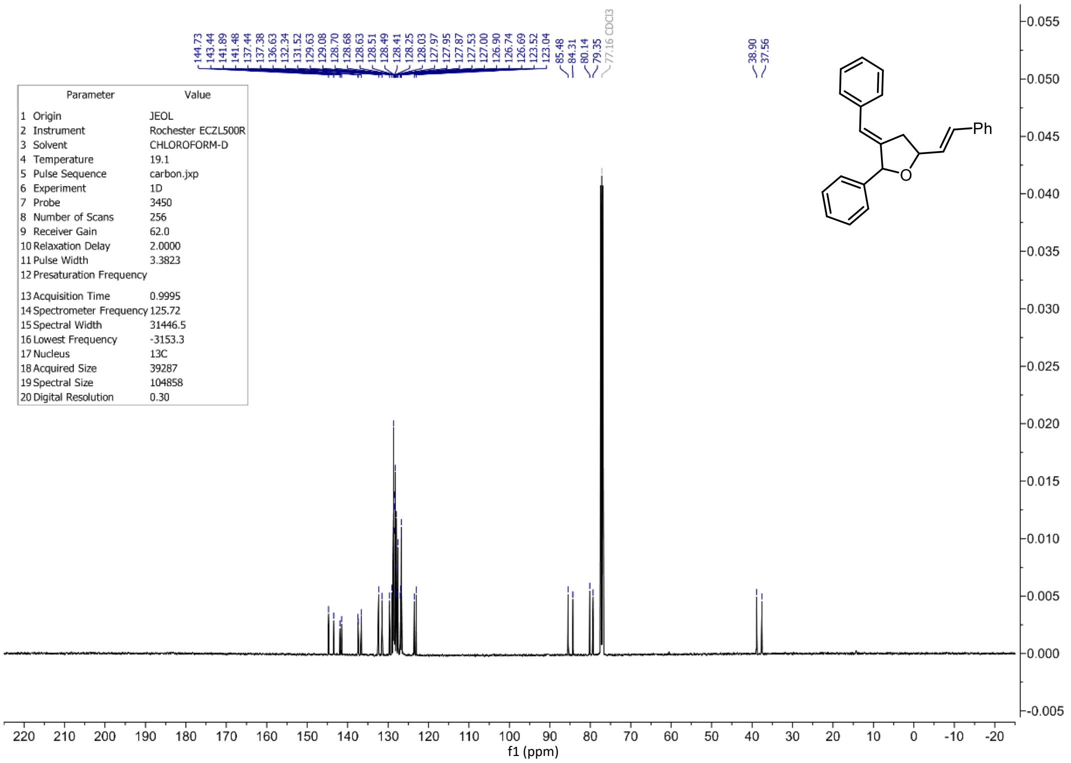

3-((*E*)-benzylidene)-2-(tert-butyl)-5-((*E*)-styryl)tetrahydrofuran (3ca)

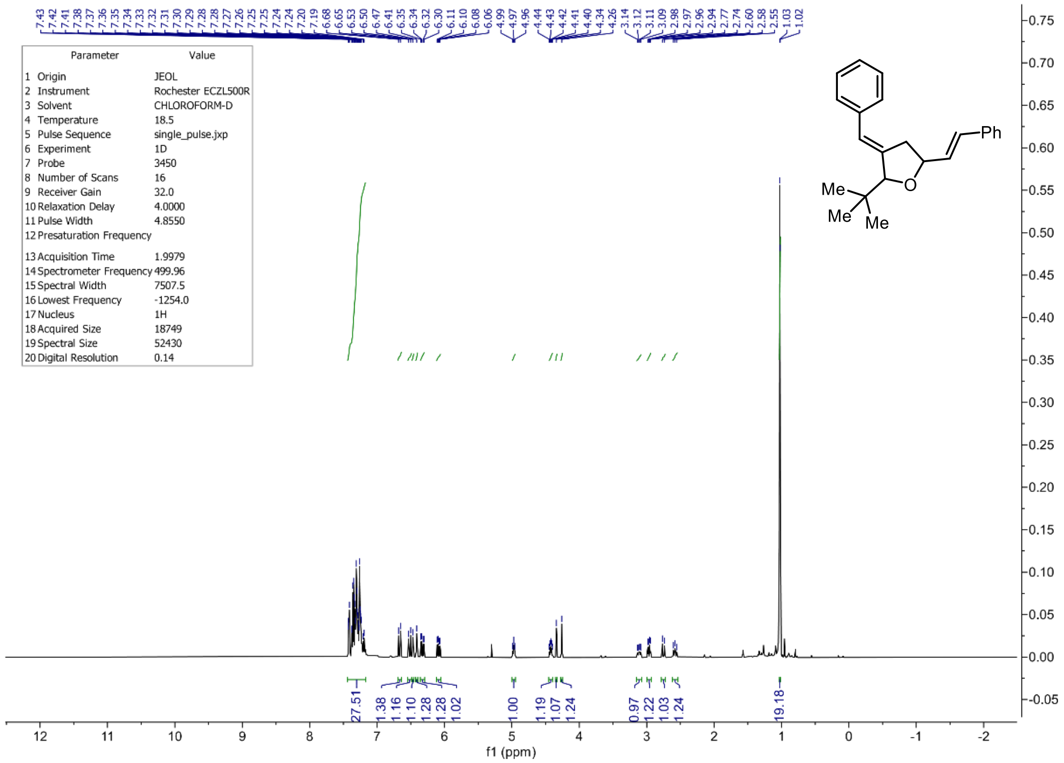

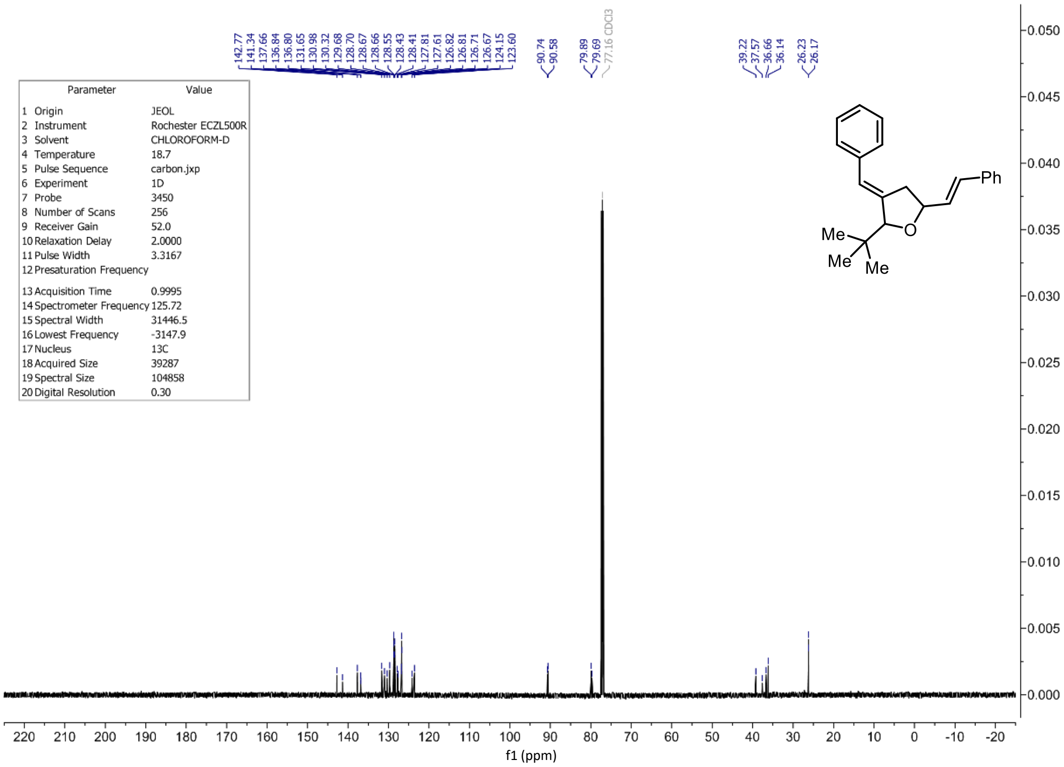

4-((*E*)-benzylidene)-2-((*E*)-styryl)tetrahydrofuran (3da)

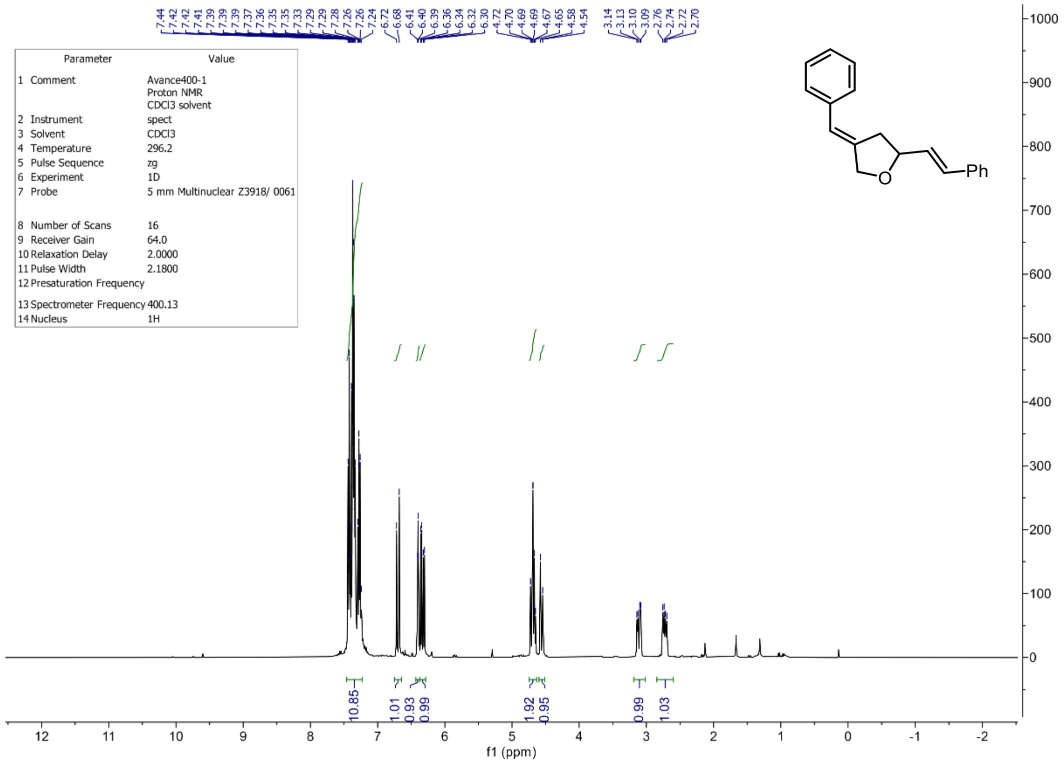

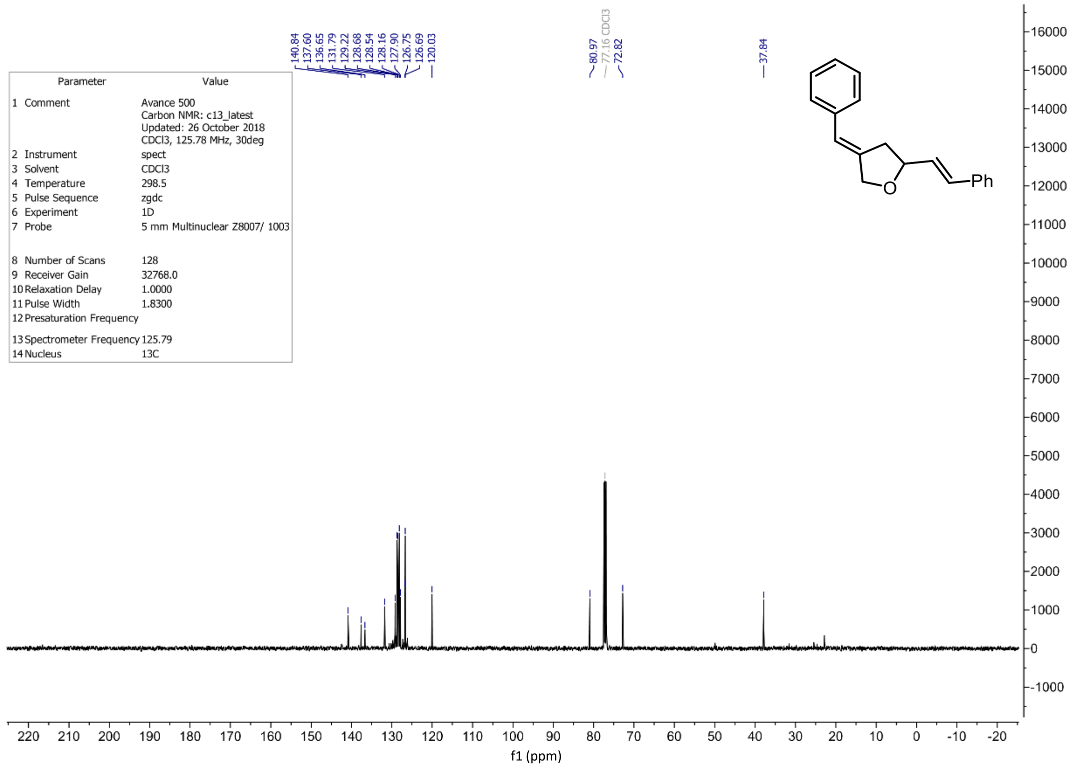

3-((*E*)-benzylidene)-2,2-dimethyl-5-((*E*)-styryl)tetrahydrofuran (3ea)

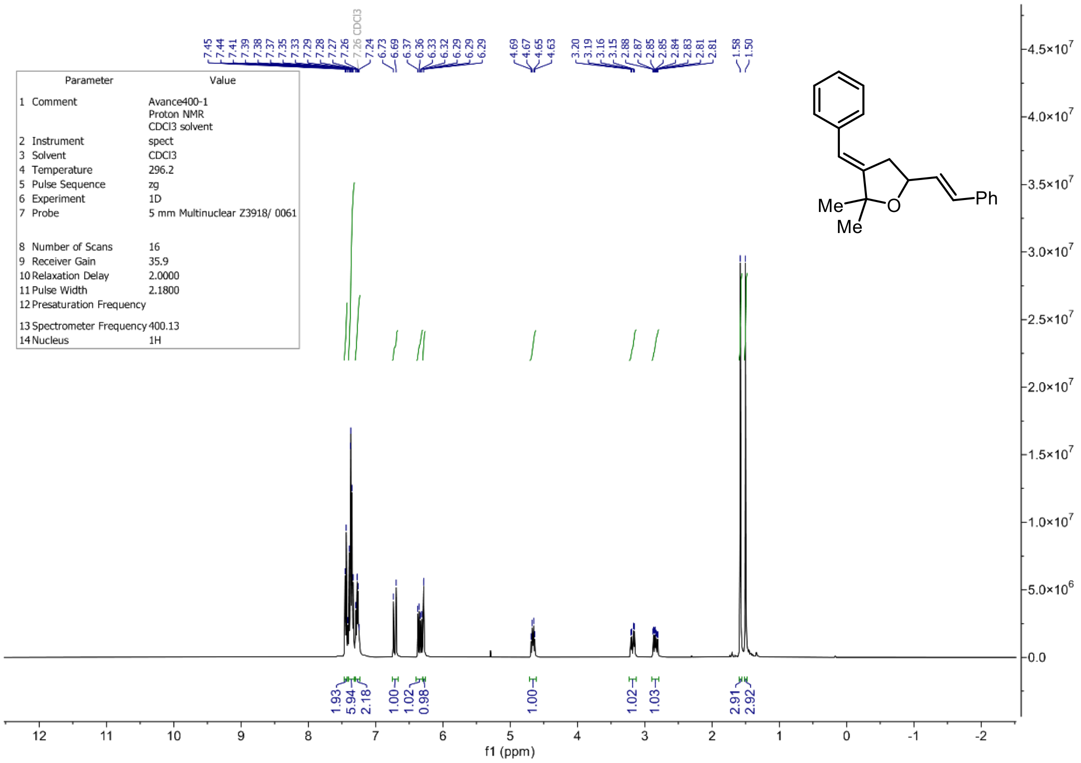

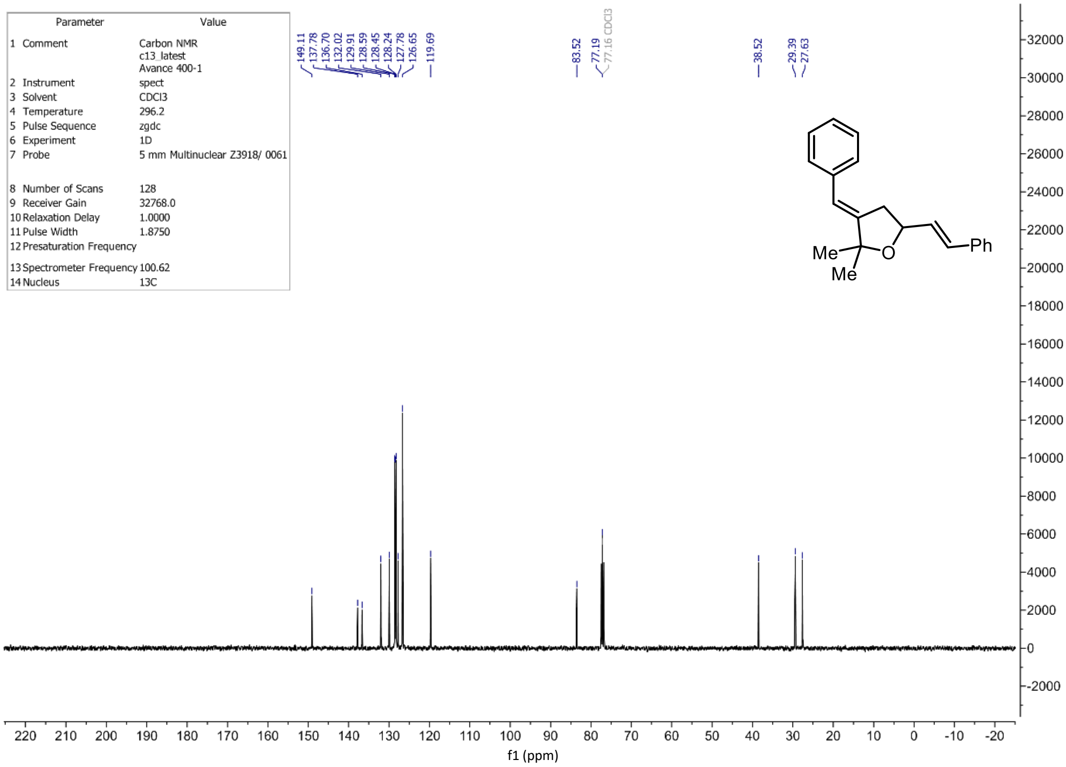

(*E*)-4-methylene-2-styryltetrahydrofuran (3fa)

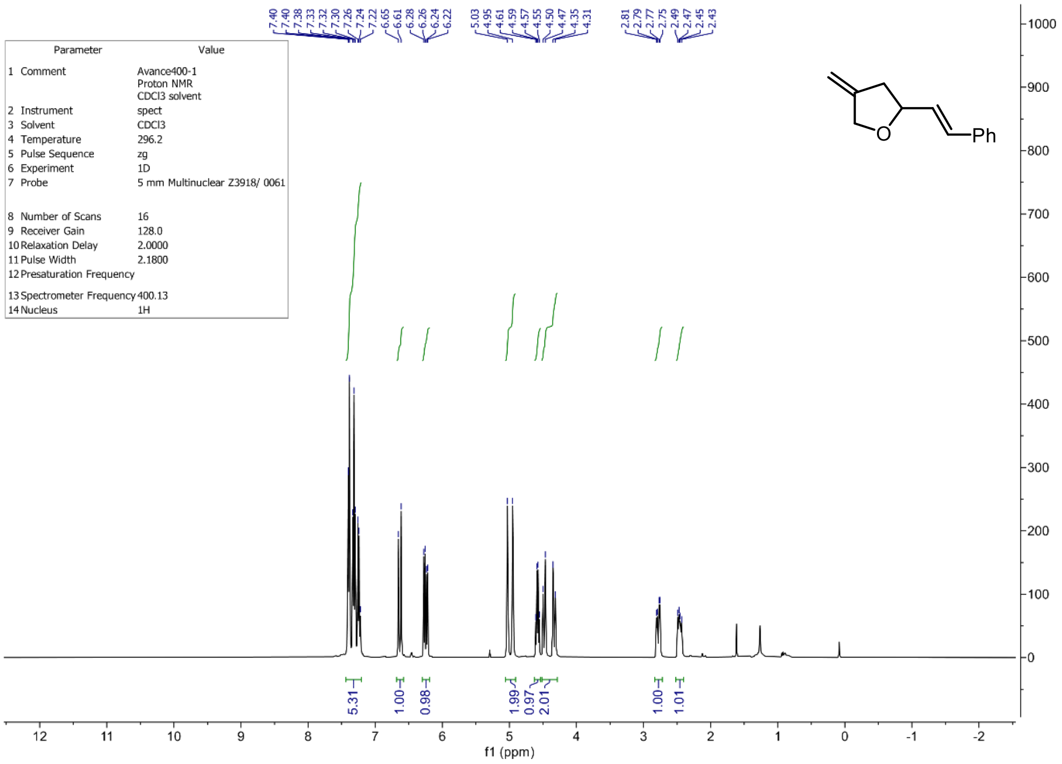

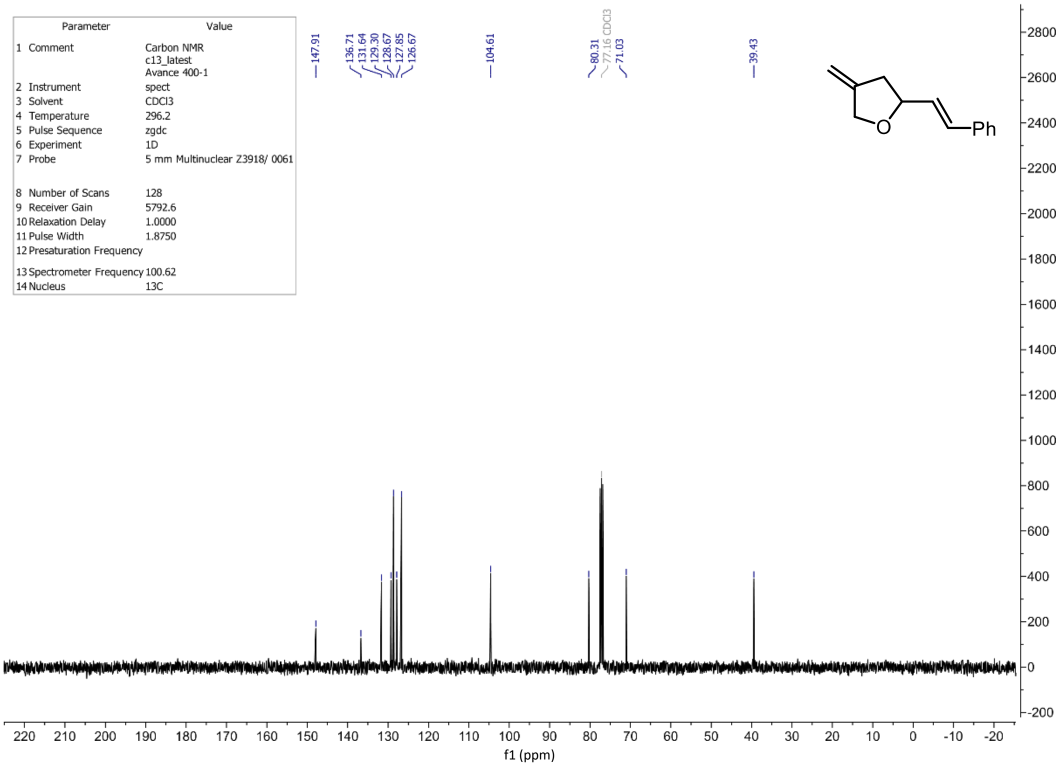

(*E*)-2-methyl-3-methylene-5-styryltetrahydrofuran (3ga)

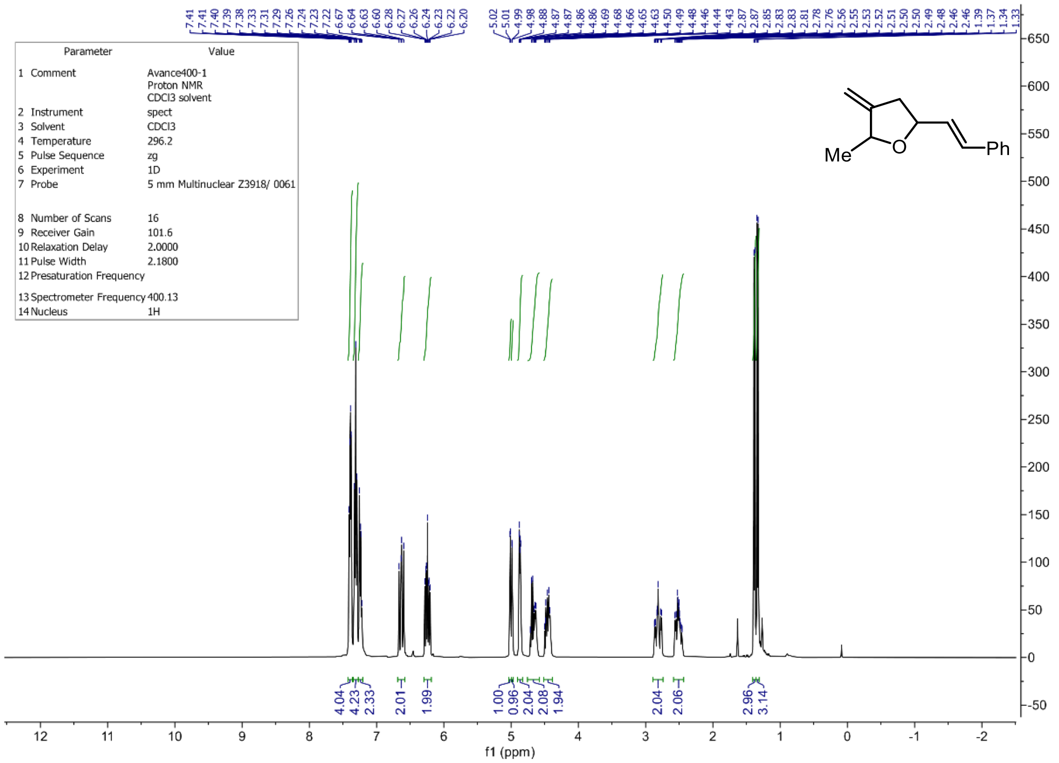

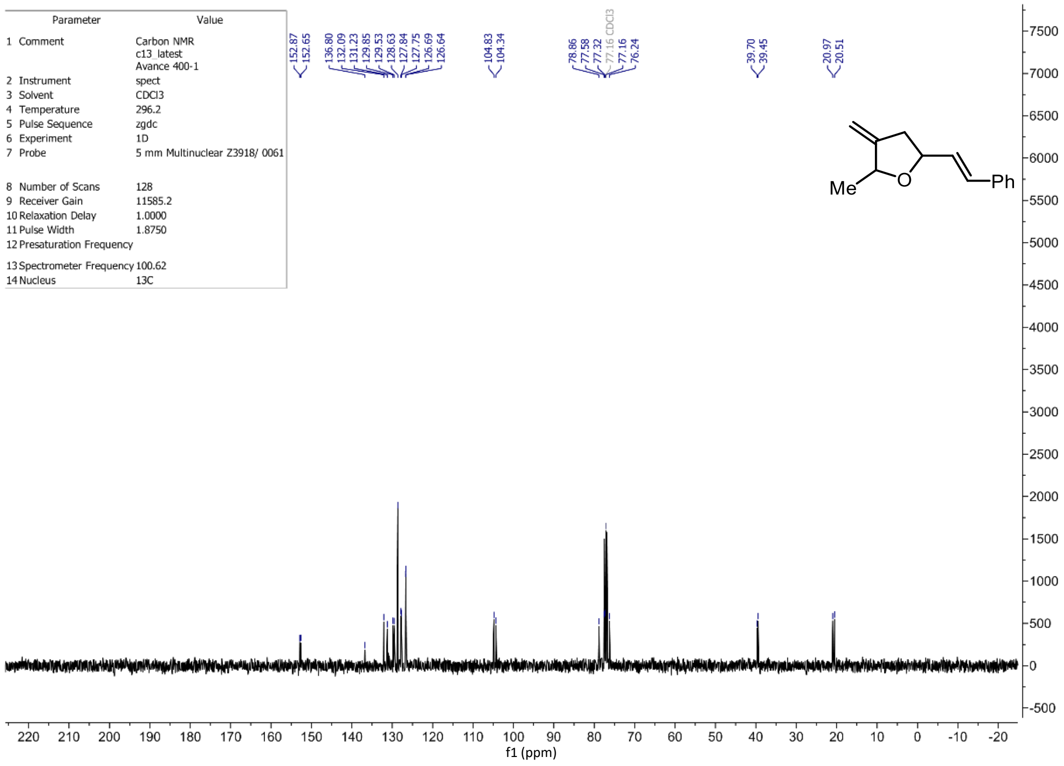

5-(3-((*E*)-benzylidene)-5-styryltetrahydrofuran-2-yl)benzo[d][1,3]dioxole (3ha)

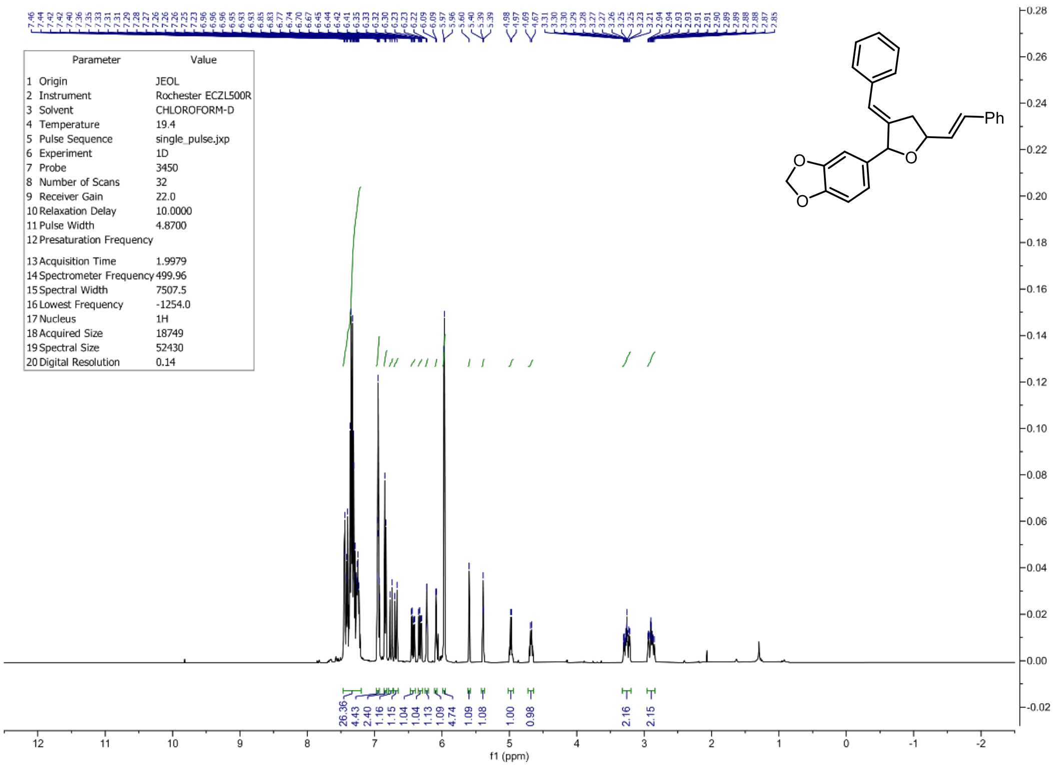

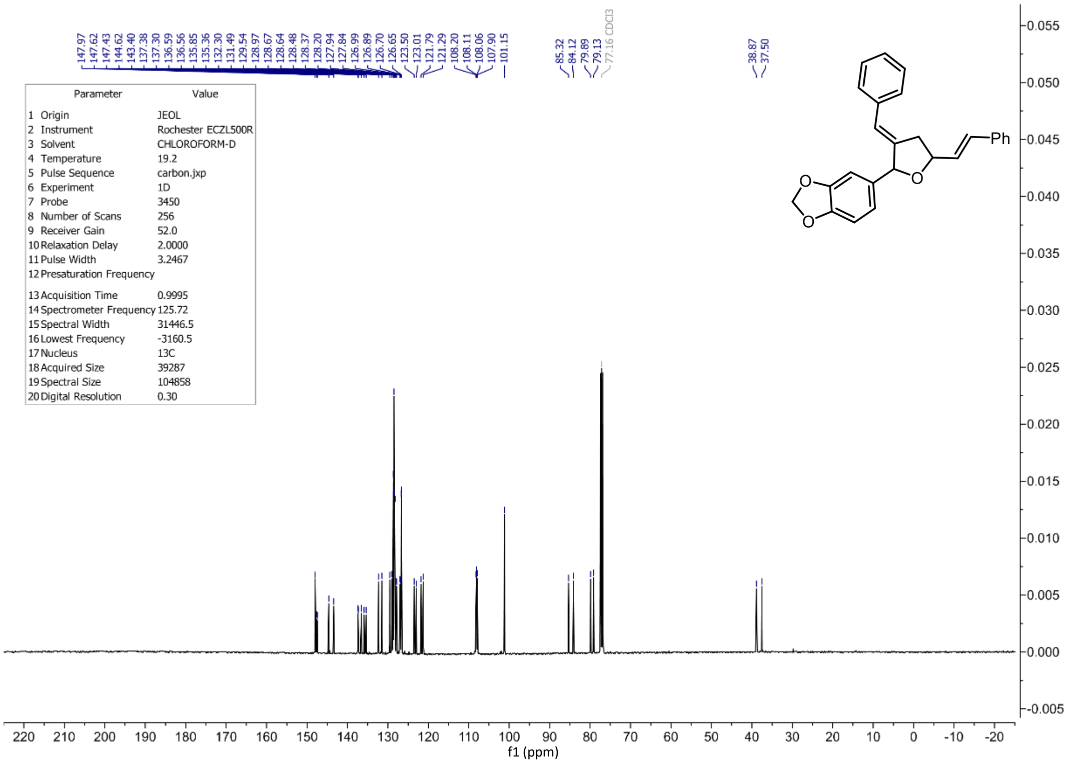

3-((*E*)-benzylidene)-2-cyclopropyl-5-((*E*)-styryl)tetrahydrofuran (3ia)

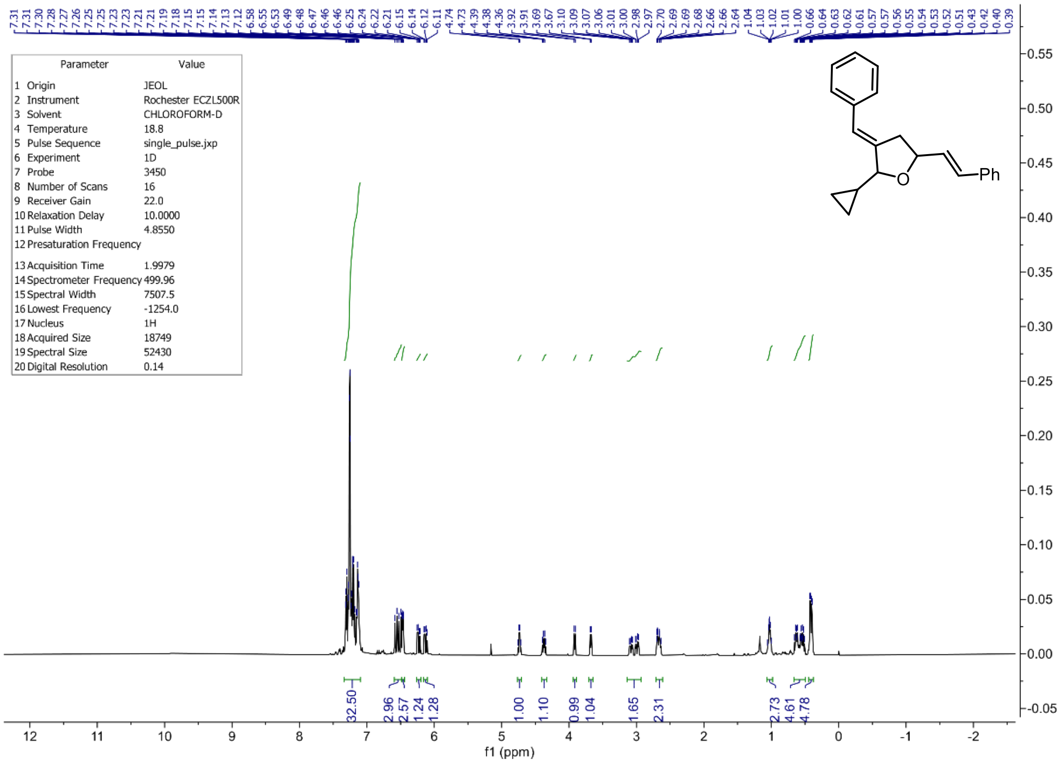

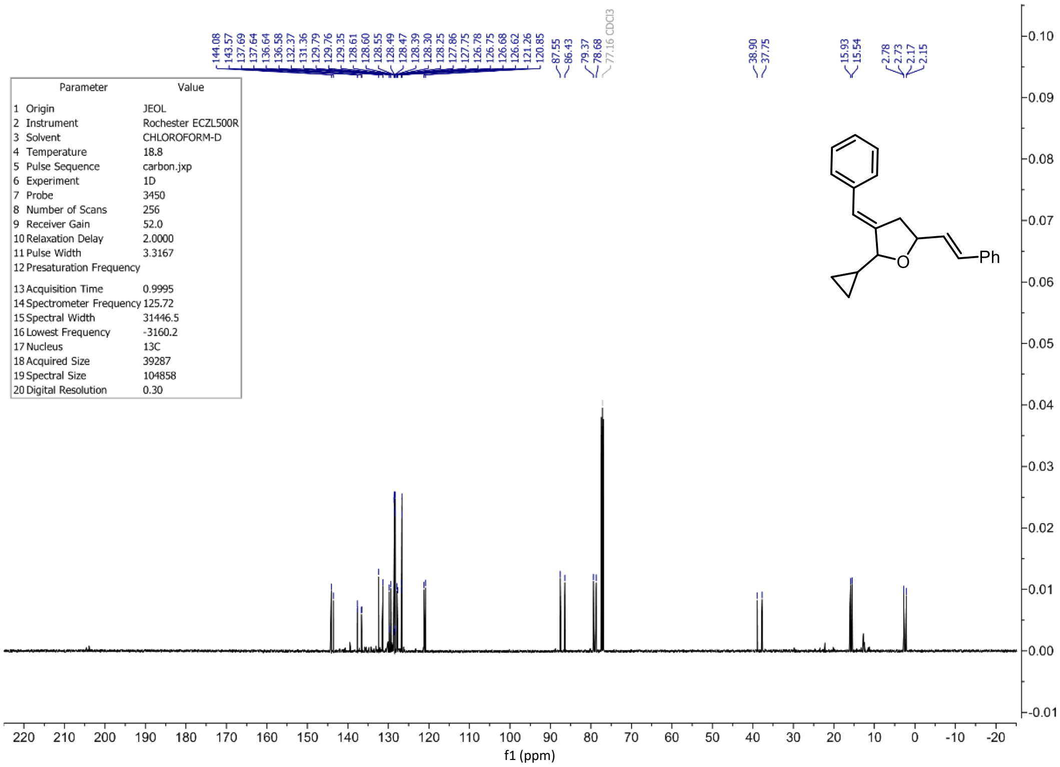

3-((*E*)-benzylidene)-5-((*E*)-styryl)-2-vinyltetrahydrofuran (3ja)

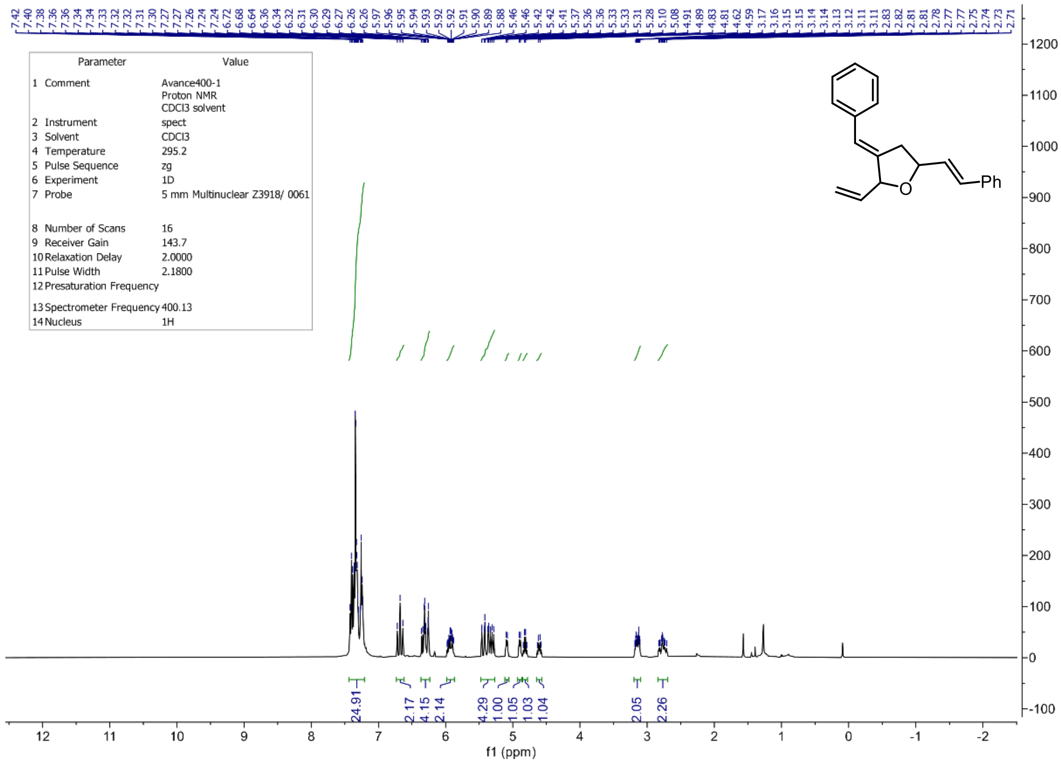

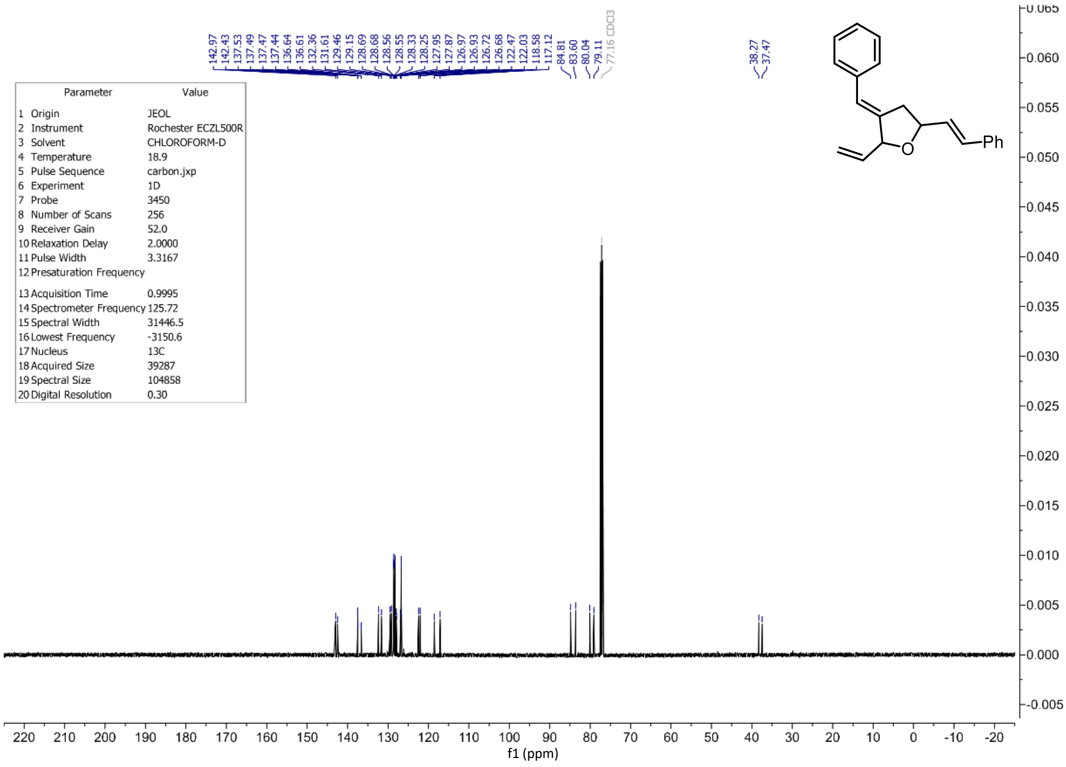

(*E*)-2-styryl-2,3,5,6,7,7a-hexahydrobenzofuran (3ka)

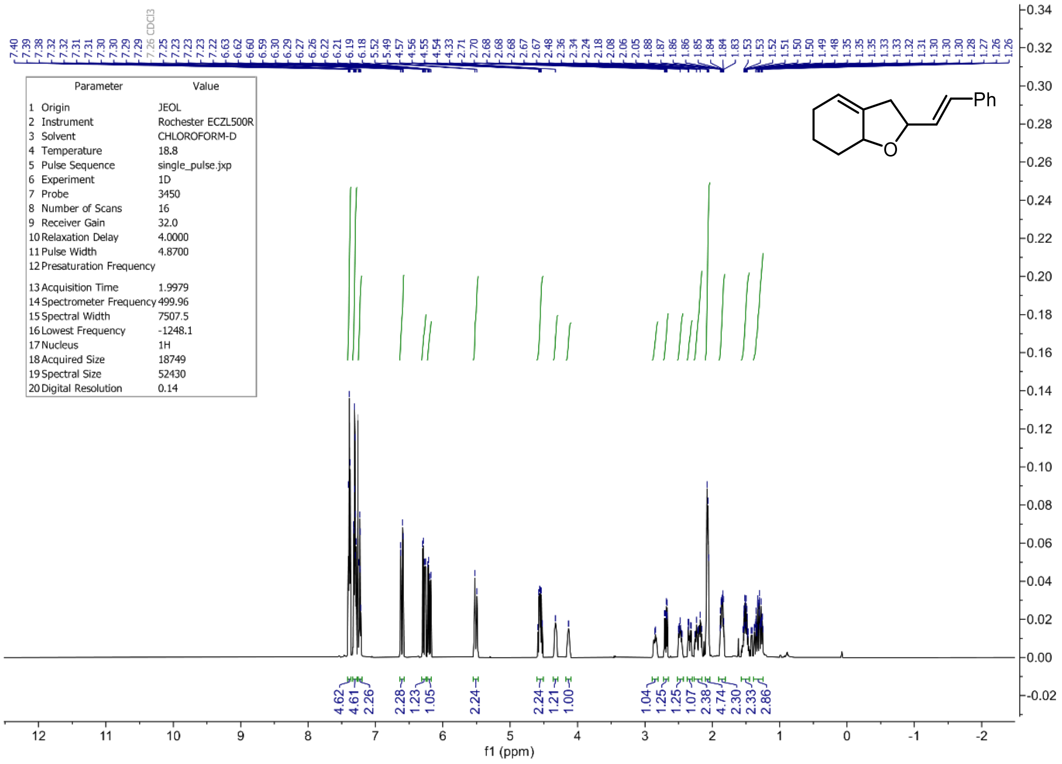

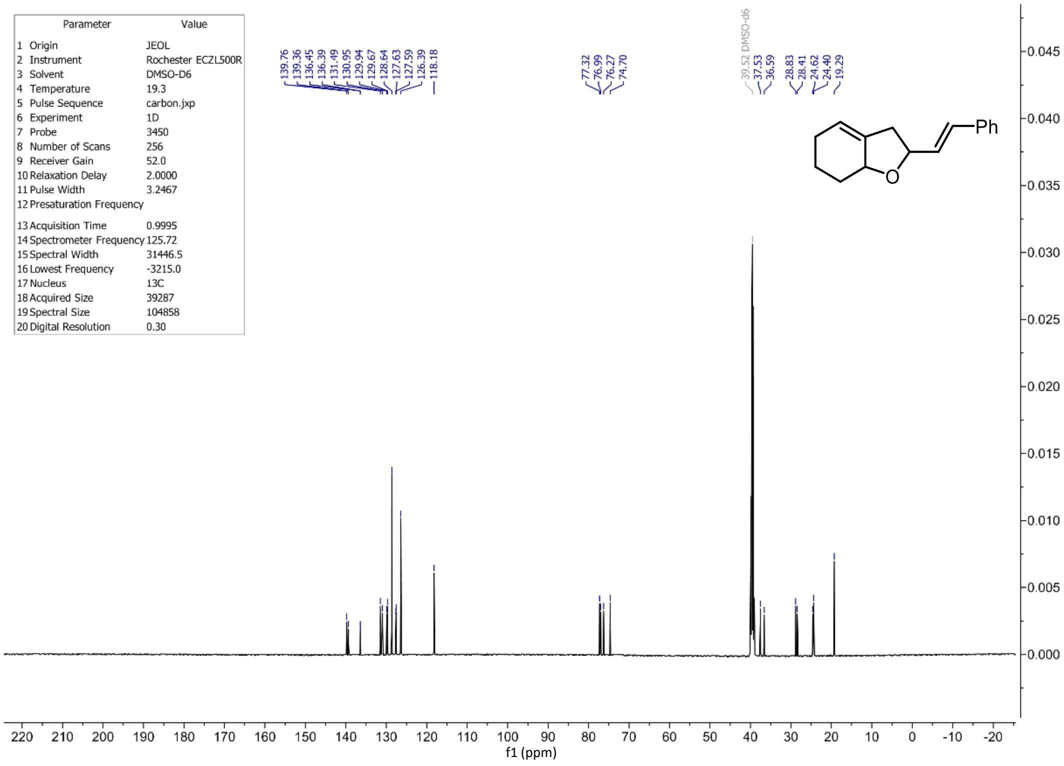

(*E*)-2-methyl-3-(3-phenylpropylidene)-5-((*E*)-styryl)tetrahydrofuran (3la)

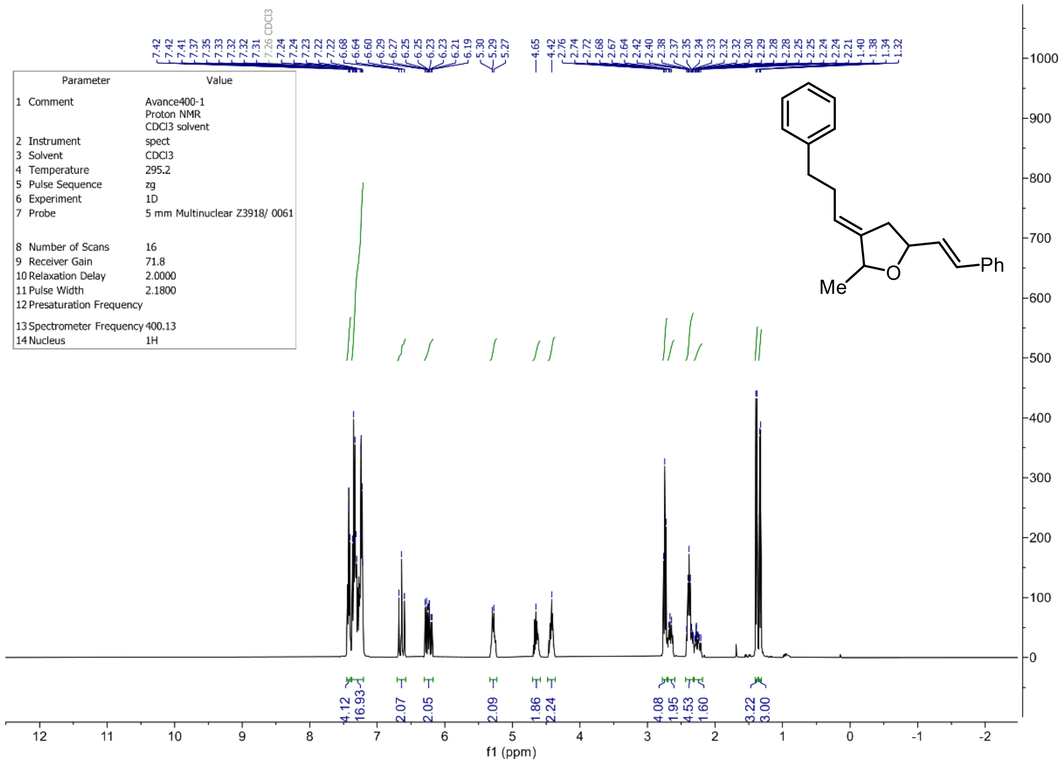

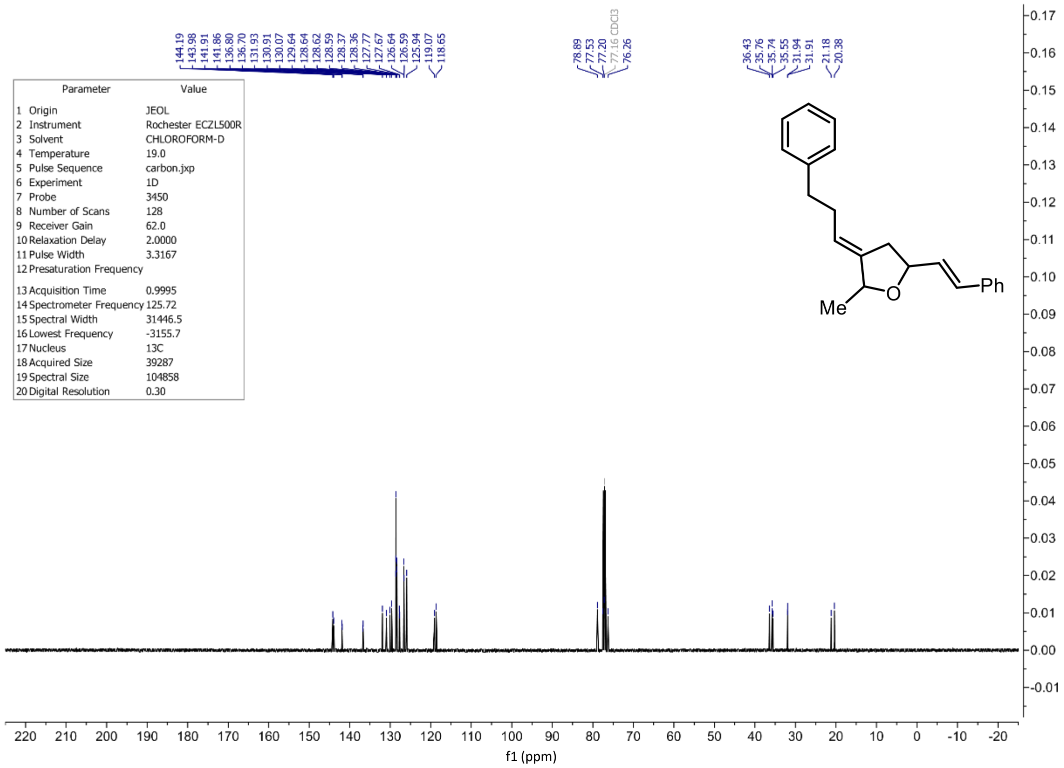

(*E*)-3-(cyclohexylmethylene)-2-methyl-5-((*E*)-styryl)tetrahydrofuran (3ma)

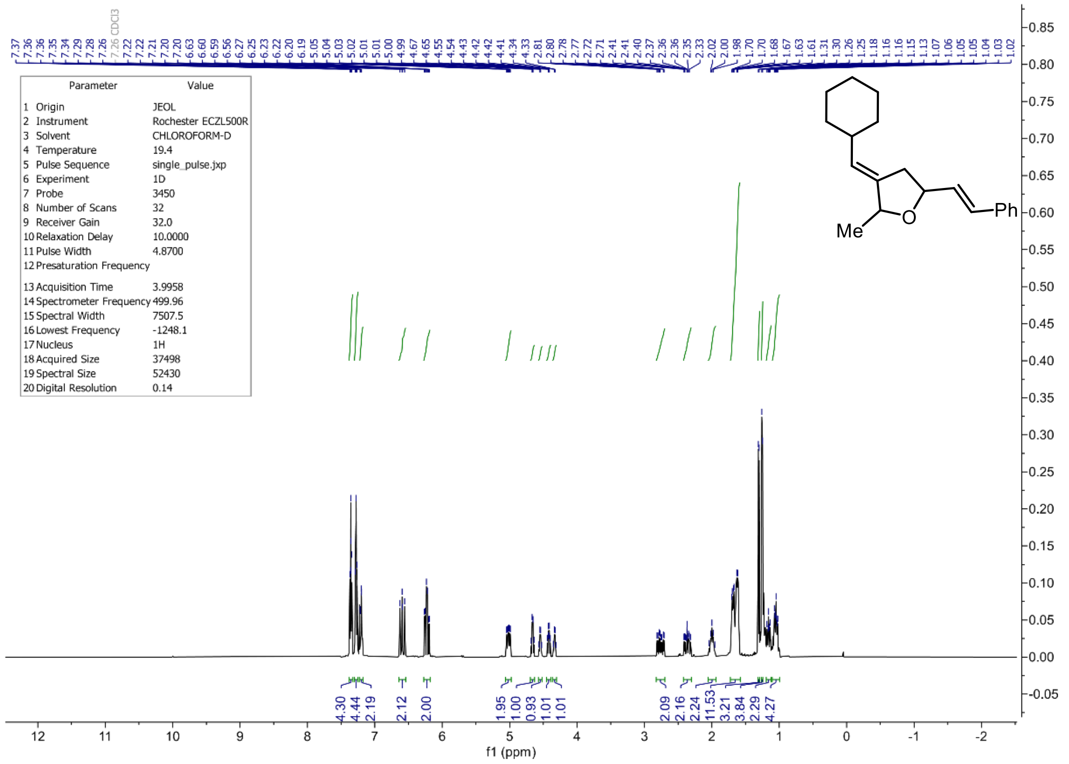

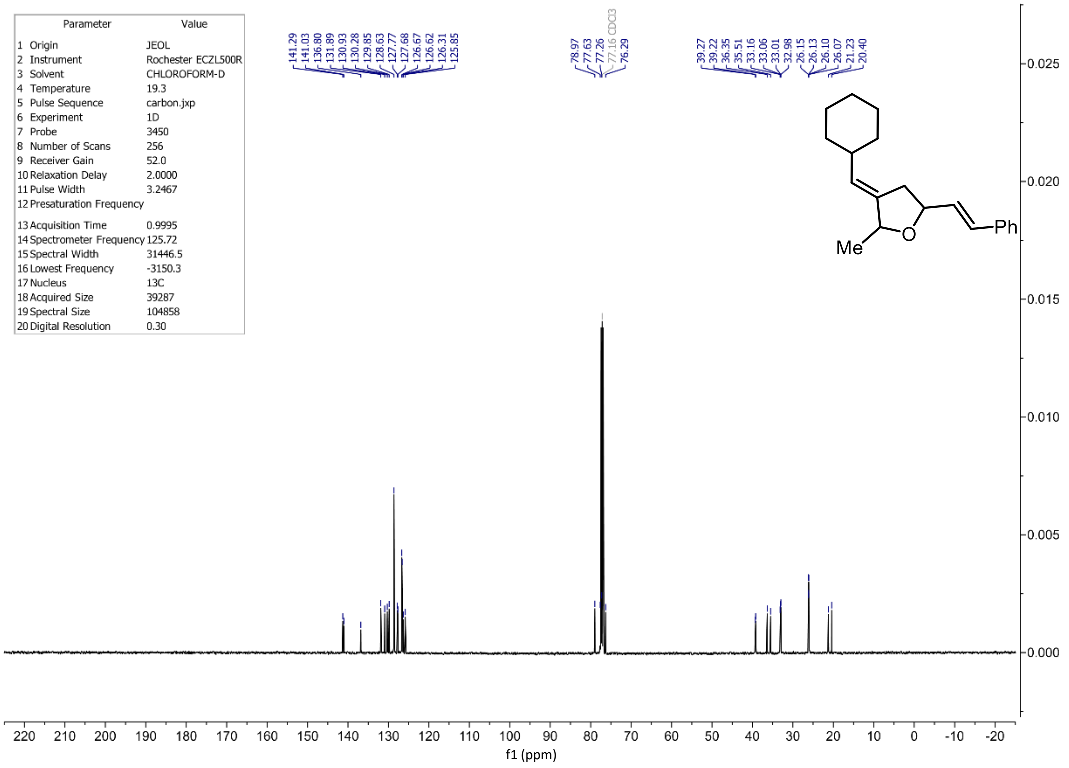

3-((*E*)-4-methoxybenzylidene)-2-methyl-5-((*E*)-styryl)tetrahydrofuran (3na)

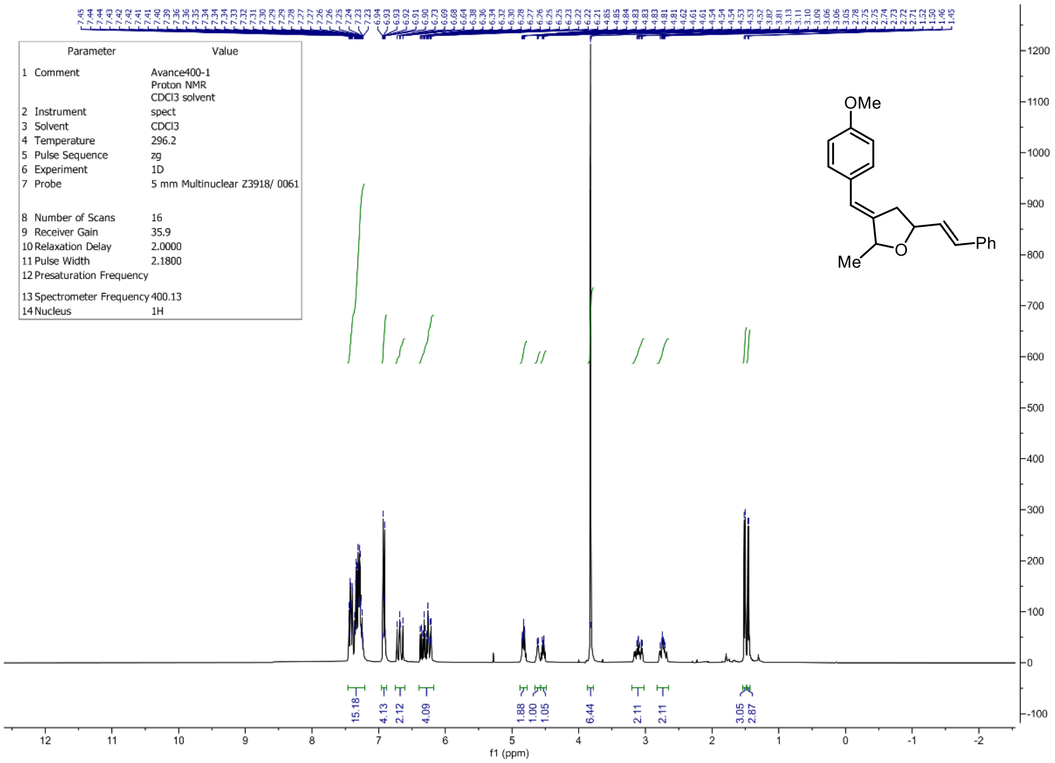

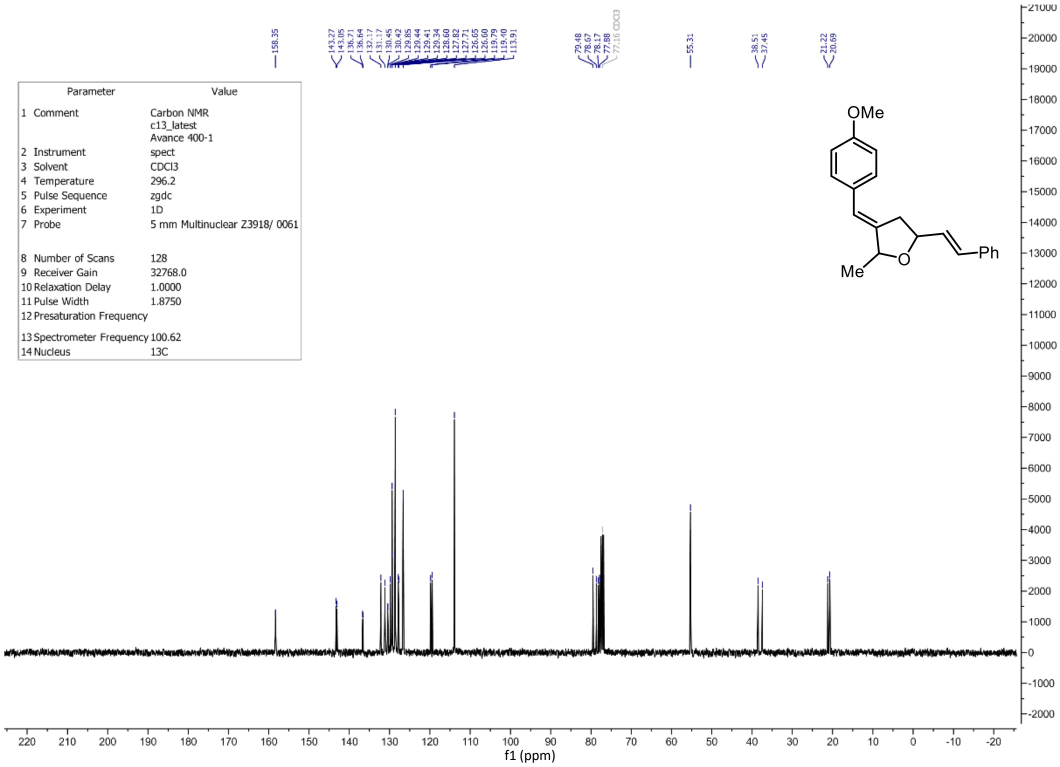

2-methyl-5-((*E*)-styryl)-3-((*E*)-4-(trifluoromethyl)benzylidene)tetrahydrofuran (3oa)

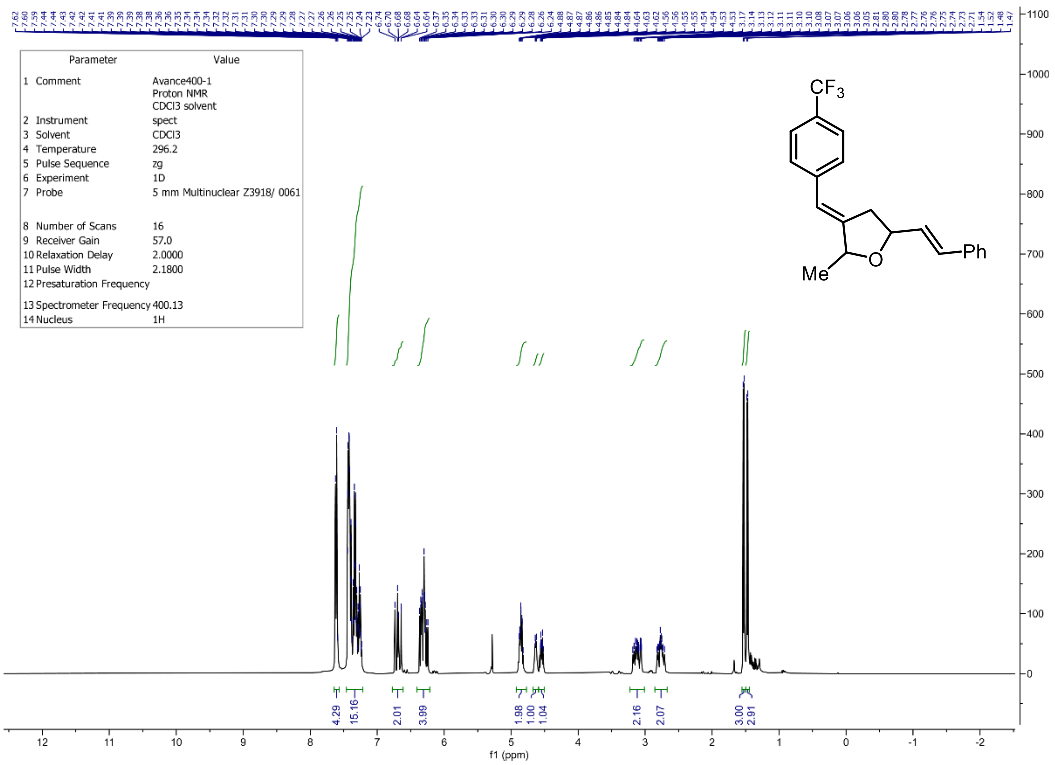

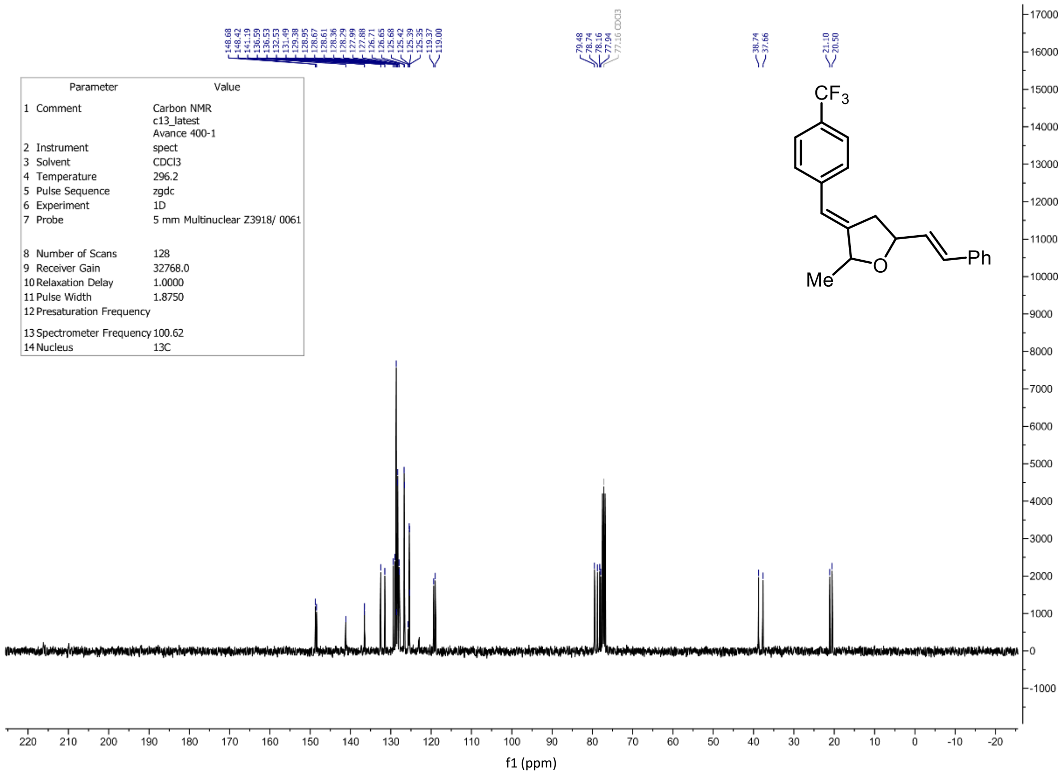

Parameter	Value
1 Origin	JEOL
2 Instrument	Rochester ECZL500R
3 Solvent	CHLOROFORM-D
4 Temperature	18.7
5 Pulse Sequence	single_pulse.jxp
6 Experiment	1D
7 Probe	3450
8 Number of Scans	16
9 Receiver Gain	42.0
10 Relaxation Delay	4.0000
11 Pulse Width	5.6350
12 Presaturation Frequency	
13 Acquisition Time	2.9849
14 Spectrometer Frequency	470.43
15 Spectral Width	94562.6
16 Lowest Frequency	-94324.6
17 Nucleus	Fluorine19
18 Acquired Size	352825
19 Spectral Size	419432
20 Digital Resolution	0.23

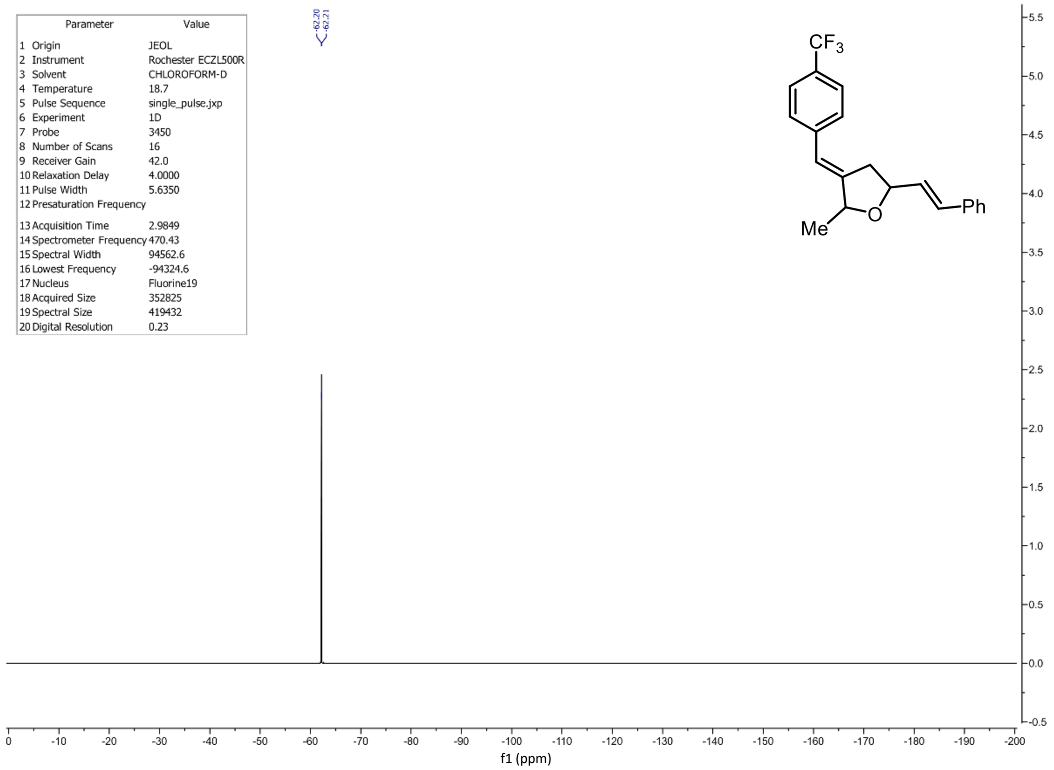

5-((*E*)-(2-methyl-5-((*E*)-styryl)dihydrofuran-3(2H)-ylidene)methyl)-2,3-dihydrobenzofuran (3pa)

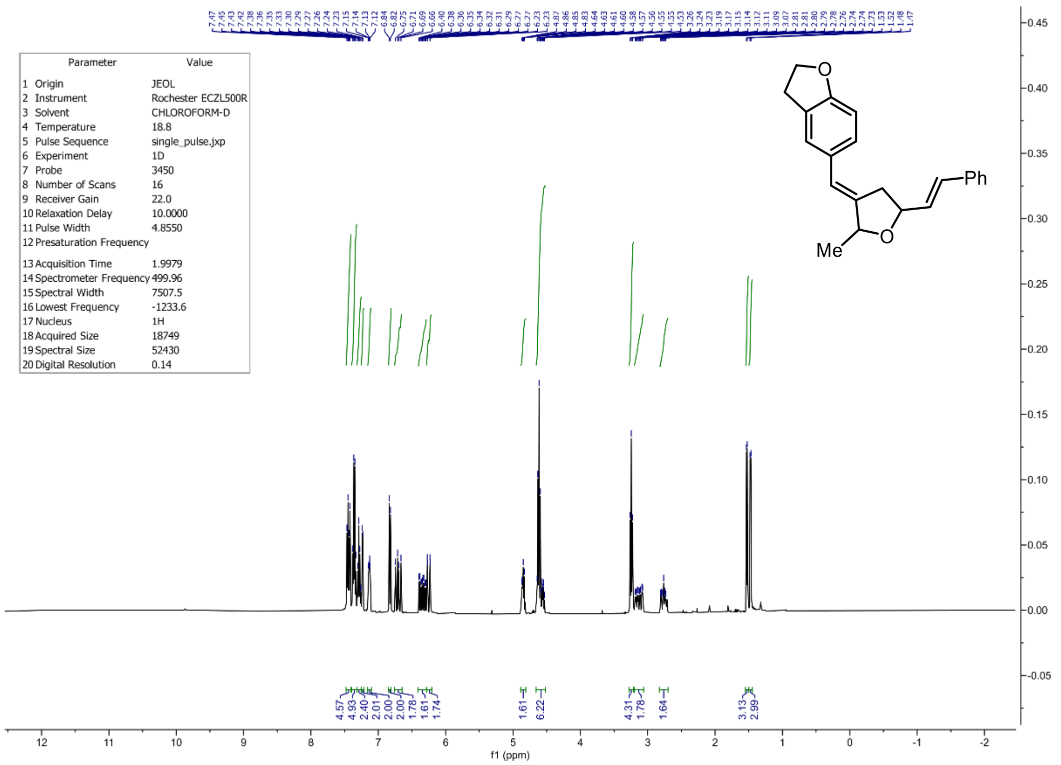

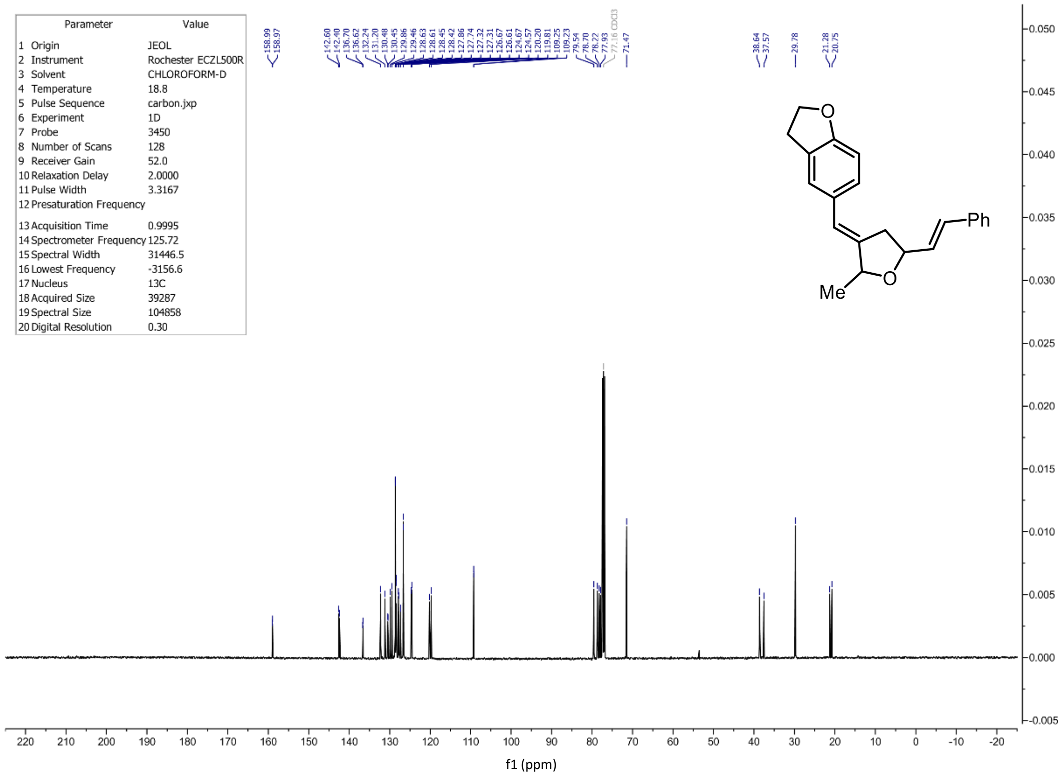

3-((1*E*)-(2-methyl-5-styryldihydrofuran-3(2H)-ylidene)methyl)pyridine (3qa)

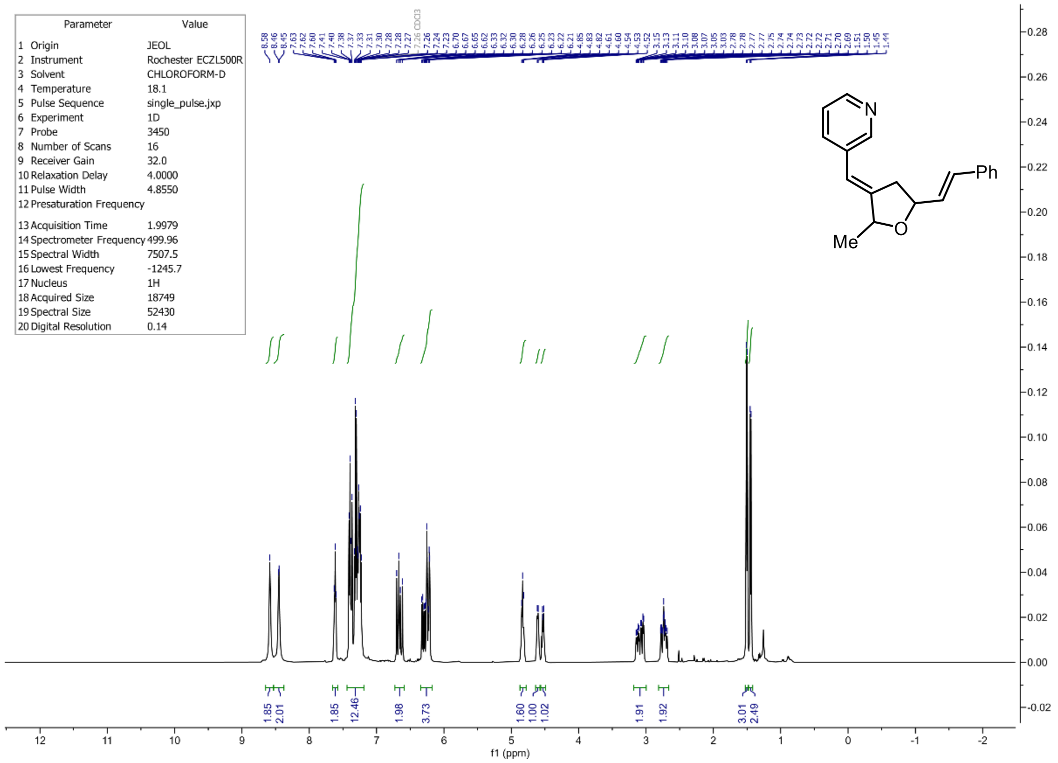

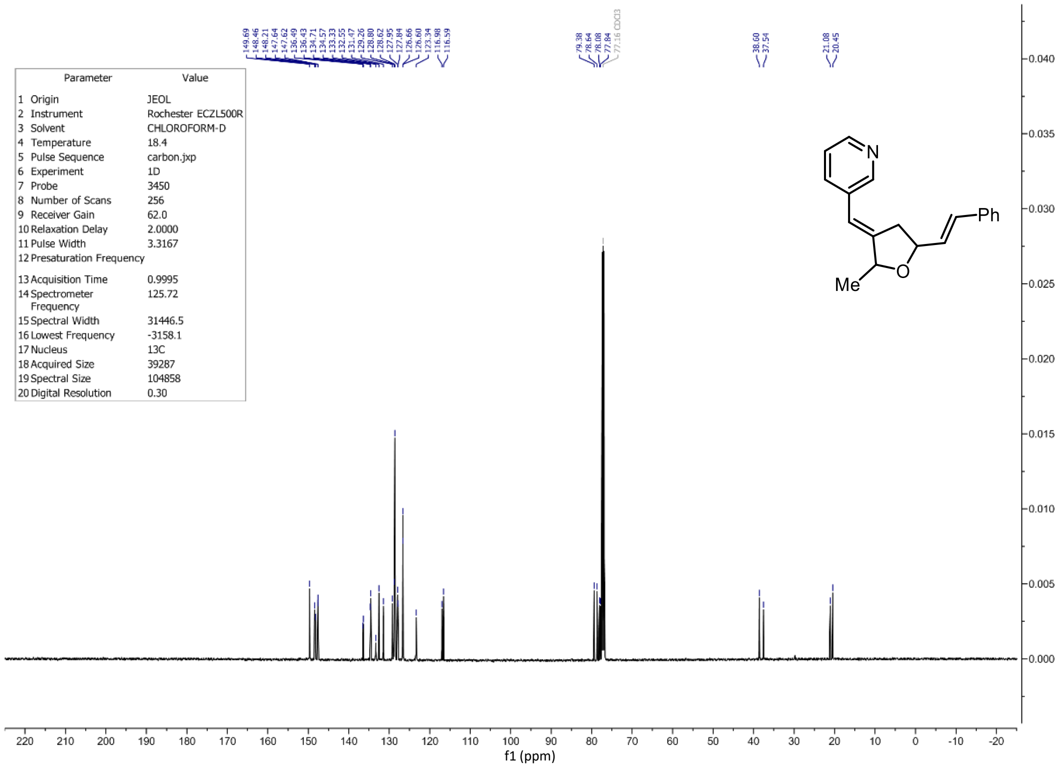

4-((*E*)-2-(4-((*E*)-benzylidene)-5-methyltetrahydrofuran-2-yl)vinyl)-N,N-dimethylaniline (3ab)

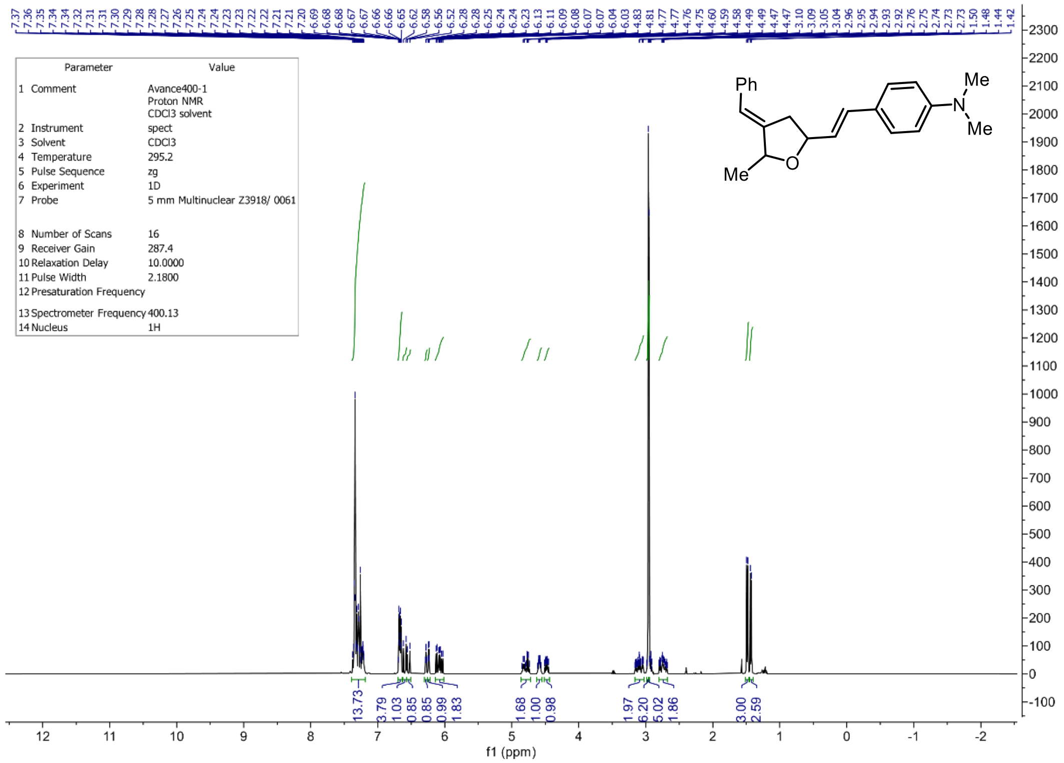

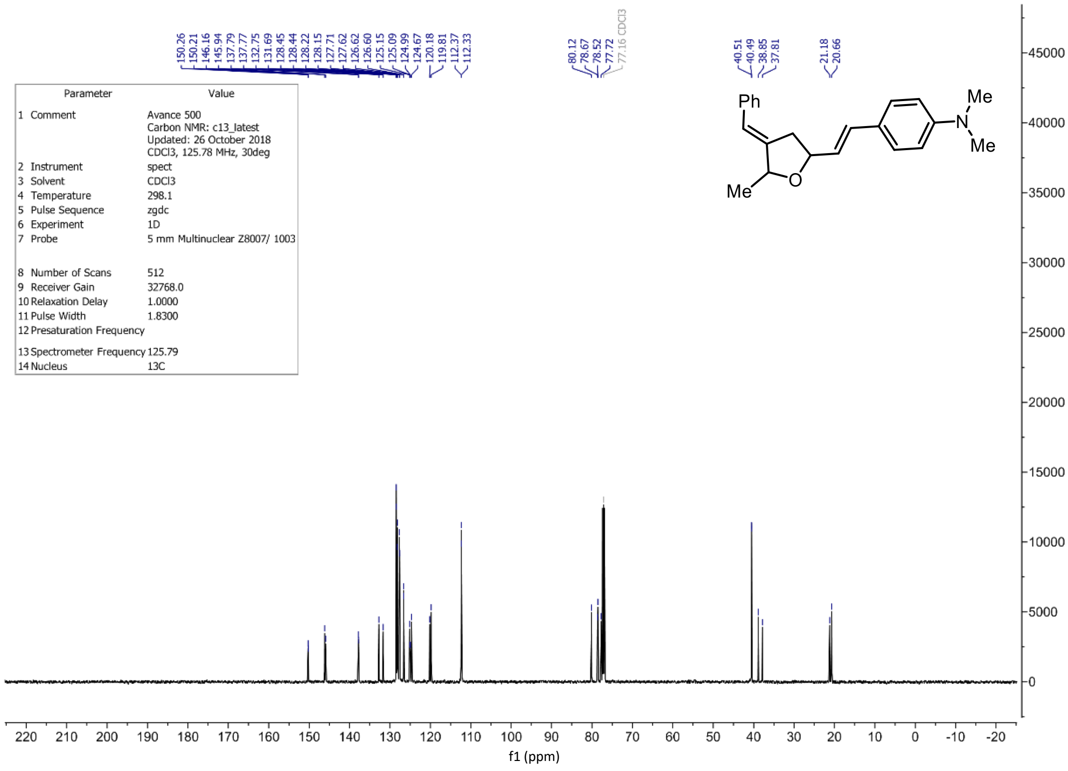

3-((*E*)-benzylidene)-5-((*E*)-4-methoxystyryl)-2-methyltetrahydrofuran (3ac)

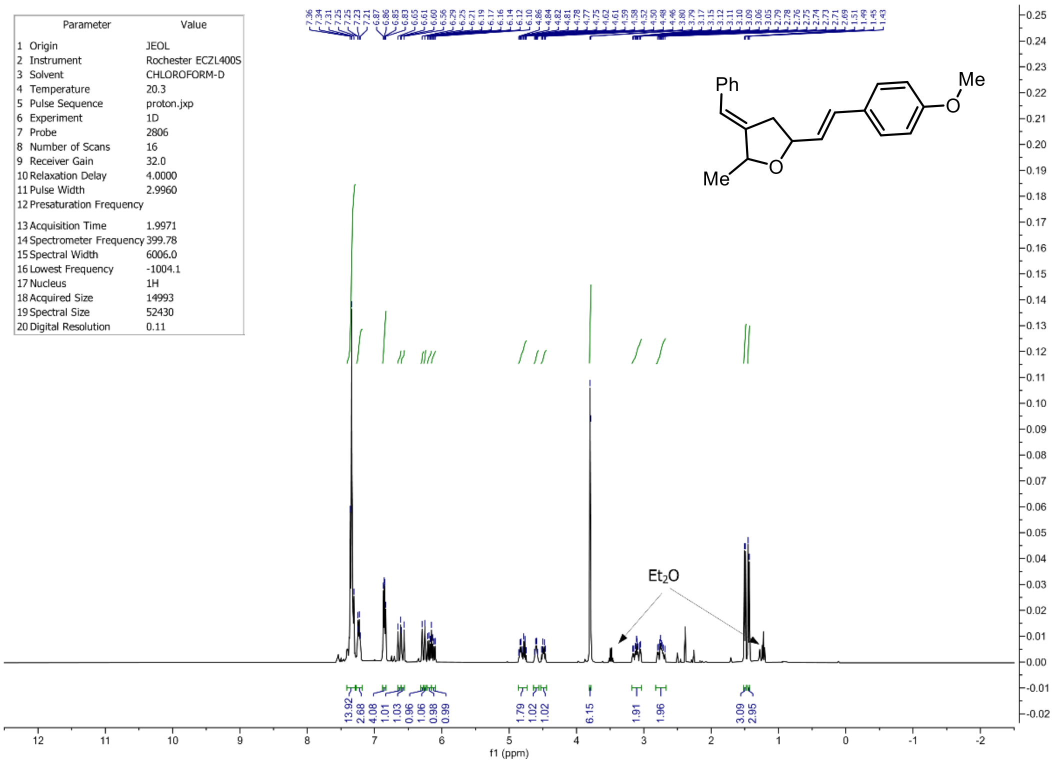

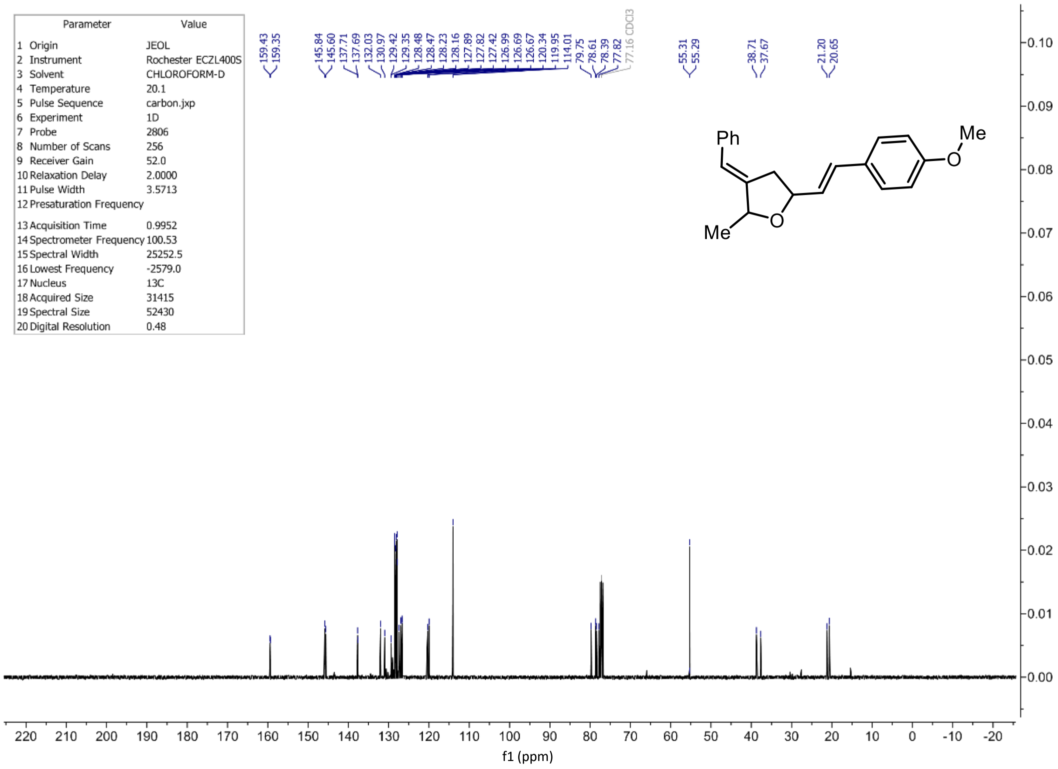

3-((*E*)-benzylidene)-2-methyl-5-((*E*)-4-(methylthio)styryl)tetrahydrofuran (3ad)

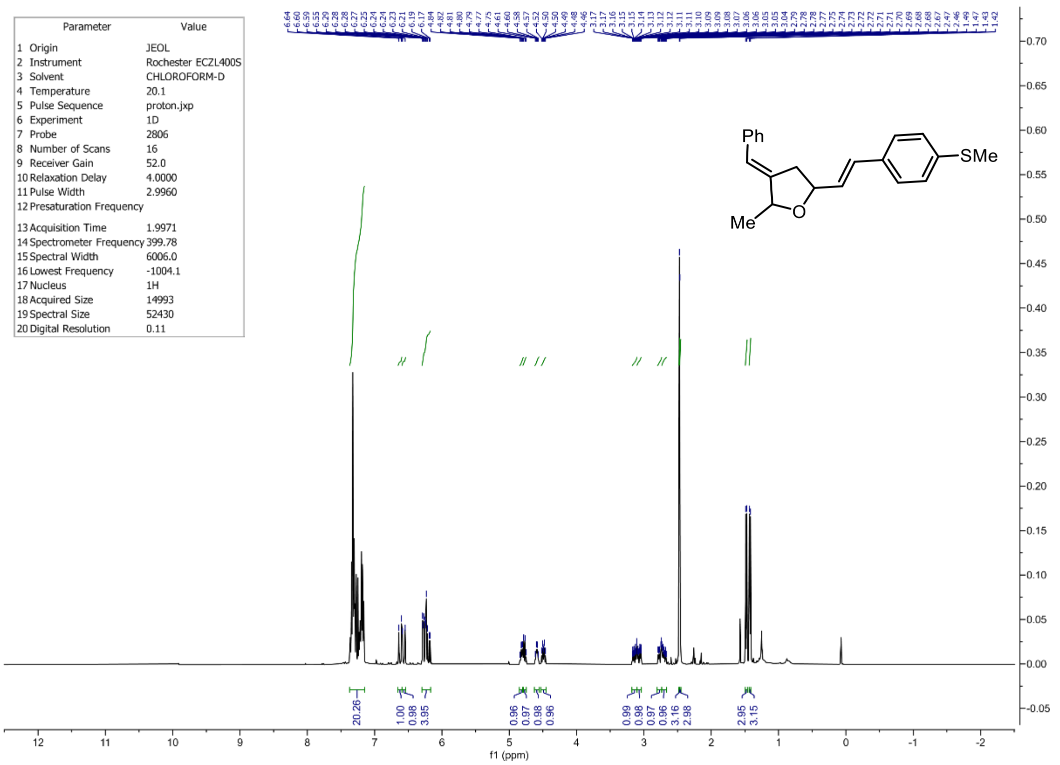

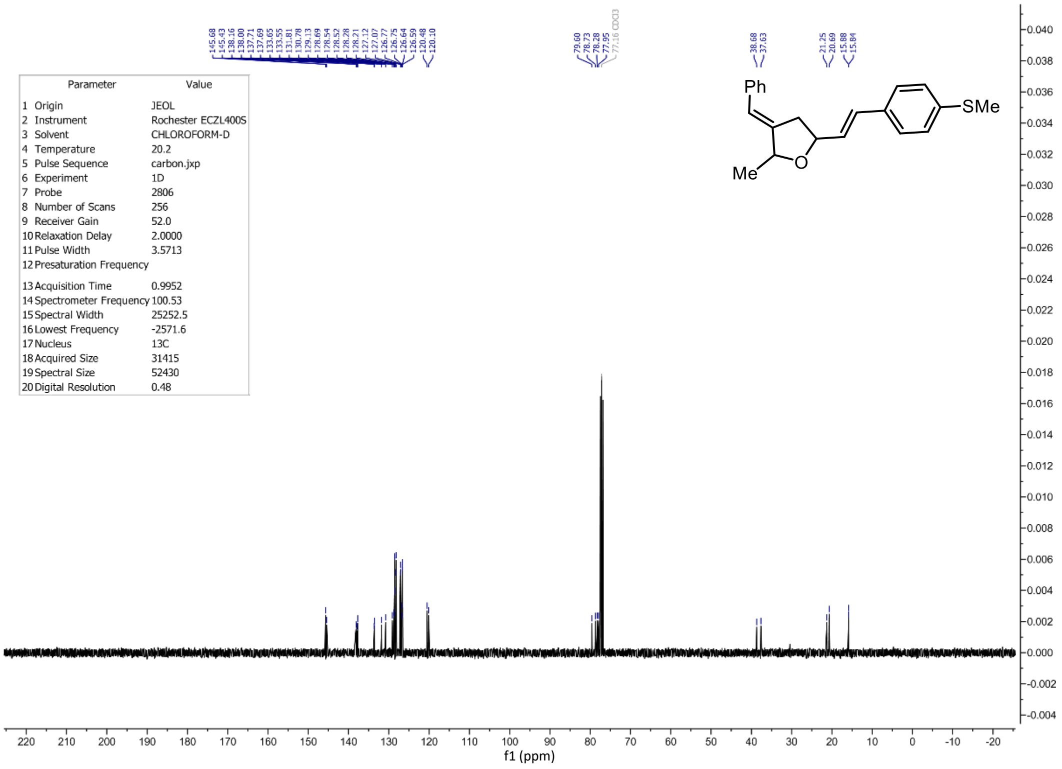

3-((*E*)-benzylidene)-5-((*E*)-4-fluorostyryl)-2-methyltetrahydrofuran (3ae)

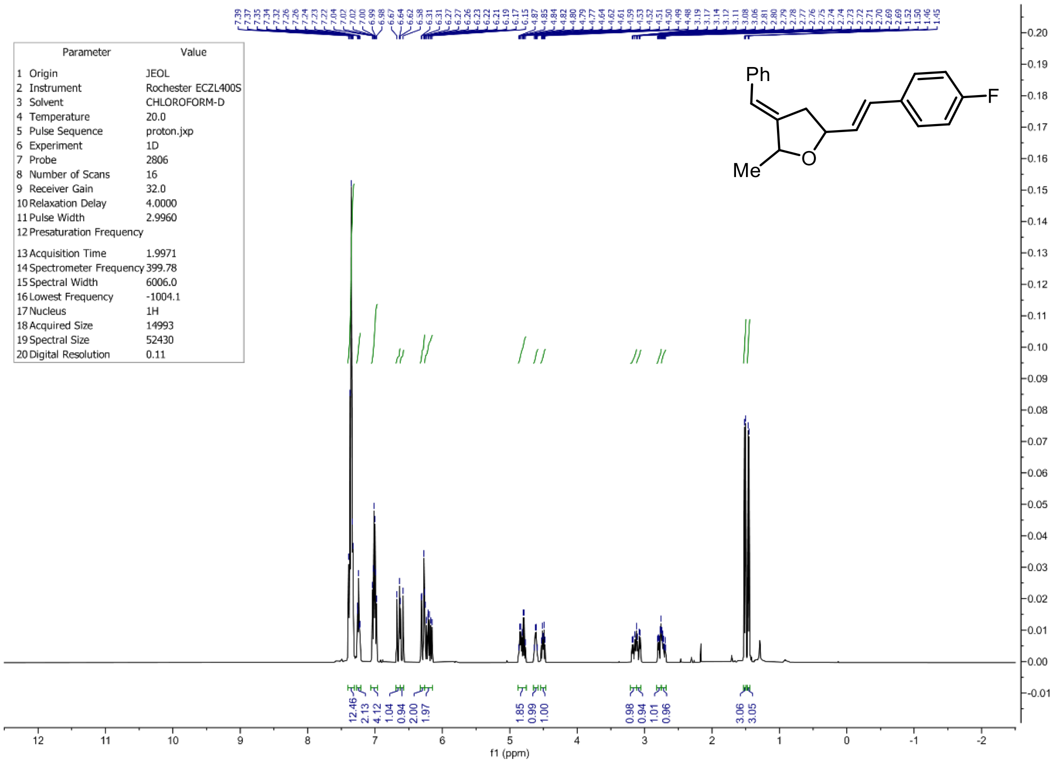

Parameter	Value
1 Origin	JEOL
2 Instrument	Rochester ECZL400S
3 Solvent	CHLOROFORM-D
4 Temperature	20.0
5 Pulse Sequence	proton.jxp
6 Experiment	1D
7 Probe	2806
8 Number of Scans	16
9 Receiver Gain	32.0
10 Relaxation Delay	4.0000
11 Pulse Width	2.9960
12 Presaturation Frequency	
13 Acquisition Time	1.9971
14 Spectrometer Frequency	399.78
15 Spectral Width	6006.0
16 Lowest Frequency	-1004.1
17 Nucleus	1H
18 Acquired Size	14993
19 Spectral Size	52430
20 Digital Resolution	0.11

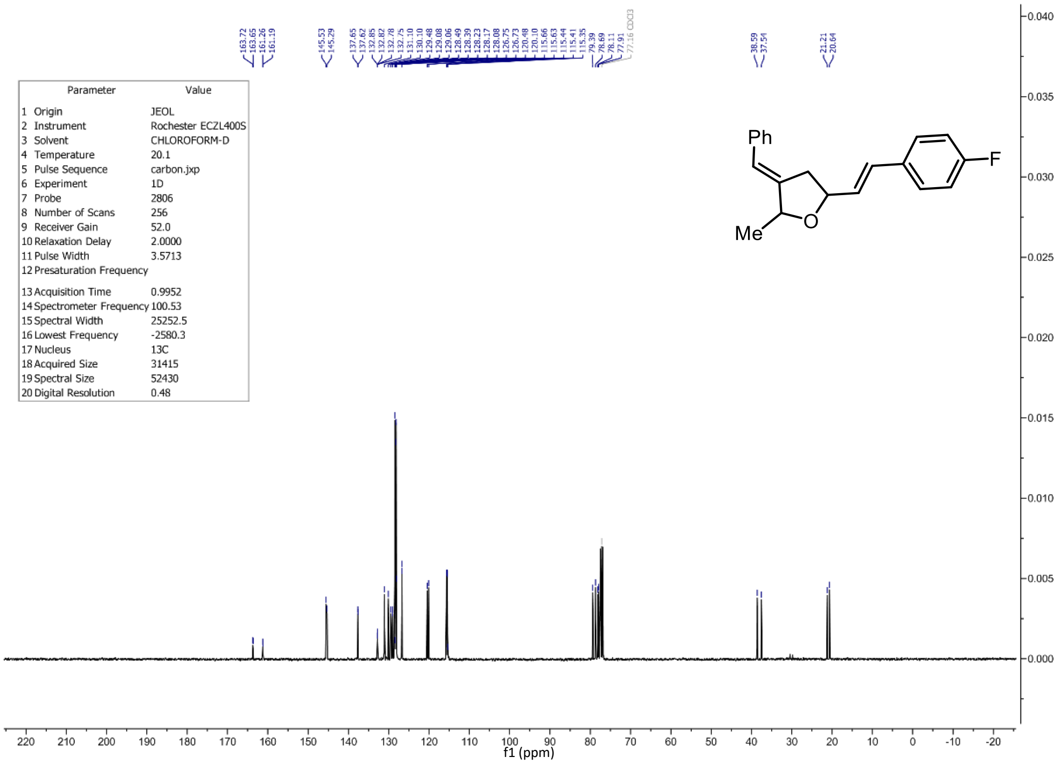

Parameter	Value
1 Origin	JEOL
2 Instrument	Rochester ECZL400S
3 Solvent	CHLOROFORM-D
4 Temperature	20.1
5 Pulse Sequence	carbon.jxp
6 Experiment	1D
7 Probe	2806
8 Number of Scans	256
9 Receiver Gain	52.0
10 Relaxation Delay	2.0000
11 Pulse Width	3.5713
12 Presaturation Frequency	
13 Acquisition Time	0.9952
14 Spectrometer Frequency	100.53
15 Spectral Width	25252.5
16 Lowest Frequency	-2580.3
17 Nucleus	13C
18 Acquired Size	31415
19 Spectral Size	52430
20 Digital Resolution	0.48

	Parameter	Value
1	Origin	JEOL
2	Instrument	Rochester ECZL400S
3	Solvent	CHLOROFORM-D
4	Temperature	19.8
5	Pulse Sequence	single_pulse.jxp
6	Experiment	1D
7	Probe	2806
8	Number of Scans	16
9	Receiver Gain	62.0
10	Relaxation Delay	4.0000
11	Pulse Width	3.7390
12	Presaturation Frequency	
13	Acquisition Time	1.9862
14	Spectrometer Frequency	376.17
15	Spectral Width	75757.6
16	Lowest Frequency	-75495.9
17	Nucleus	Fluorine19
18	Acquired Size	188087
19	Spectral Size	419432
20	Digital Resolution	0.18

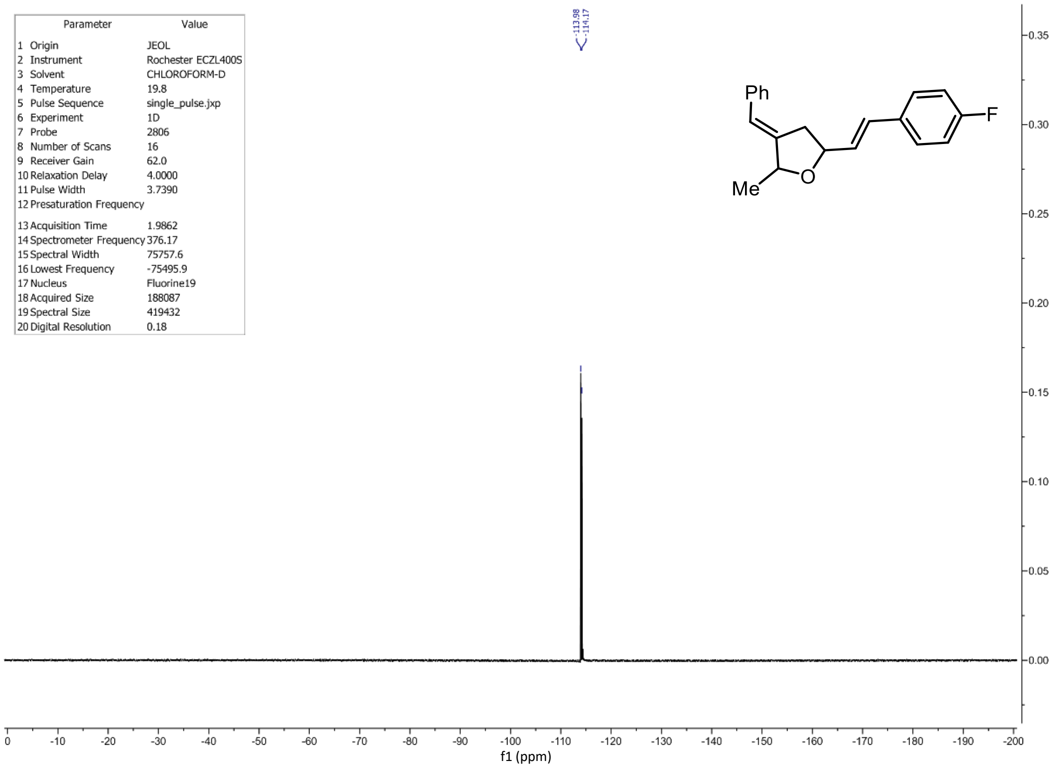

Methyl 4-((*E*)-2-(4-((*E*)-benzylidene)-5-methyltetrahydrofuran-2-yl)vinyl)benzoate (3af)

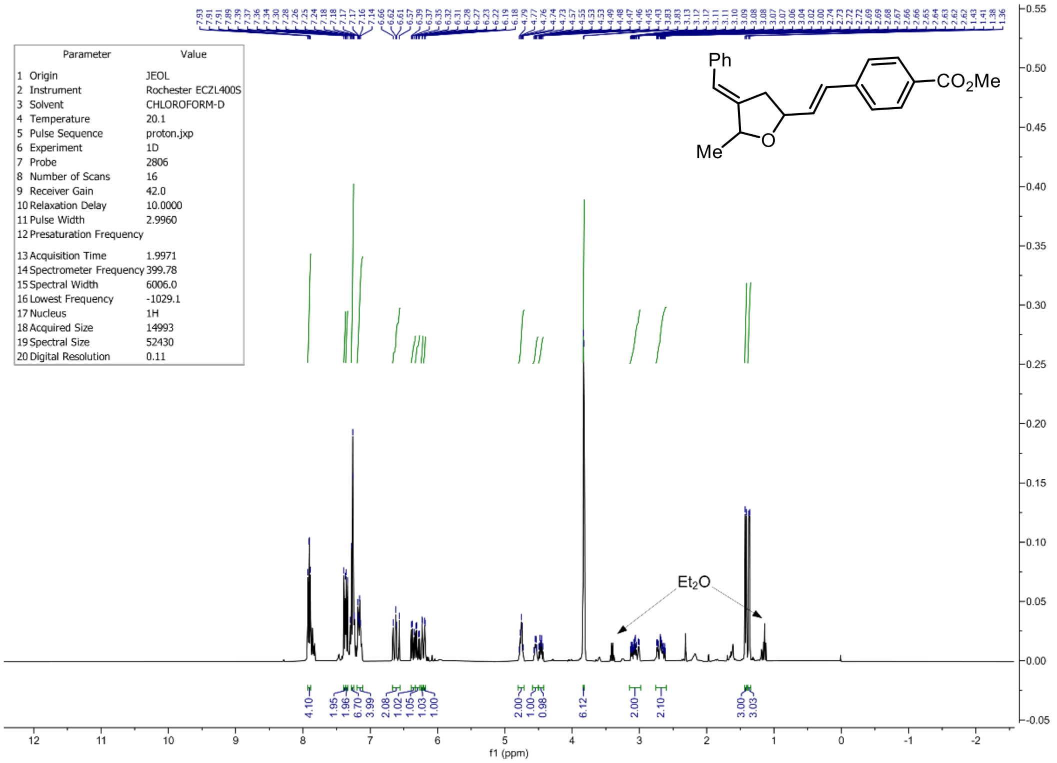

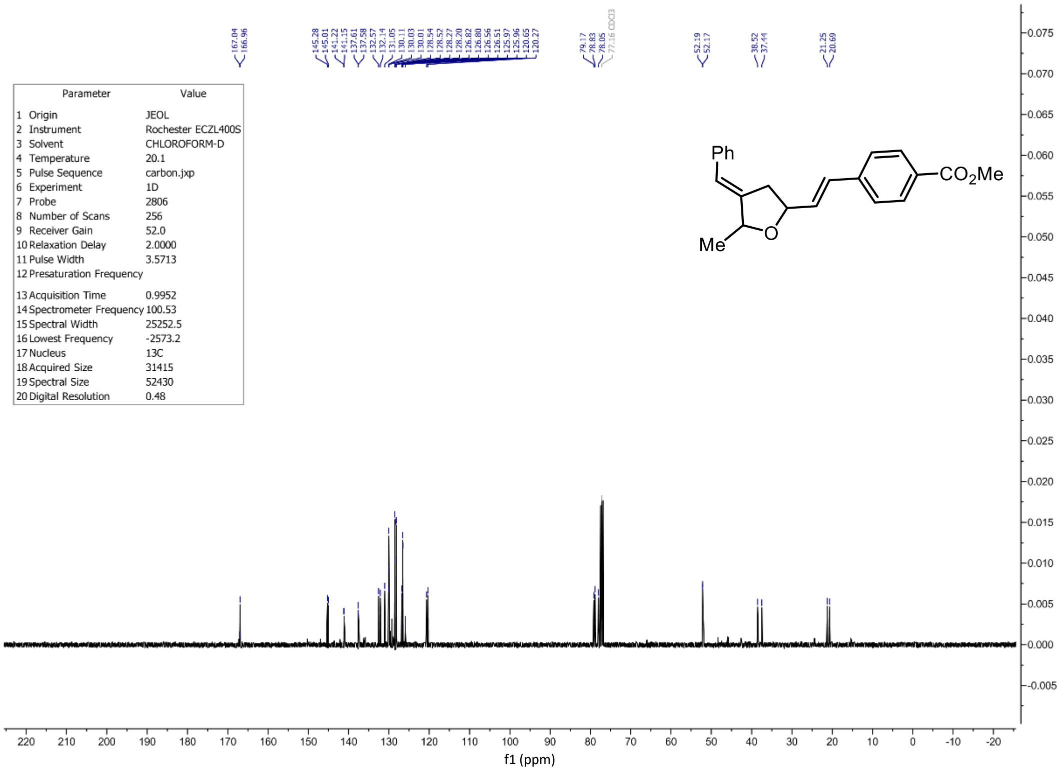

4-((*E*)-2-(4-((*E*)-benzylidene)-5-methyltetrahydrofuran-2-yl)vinyl)benzonitrile (3ag)

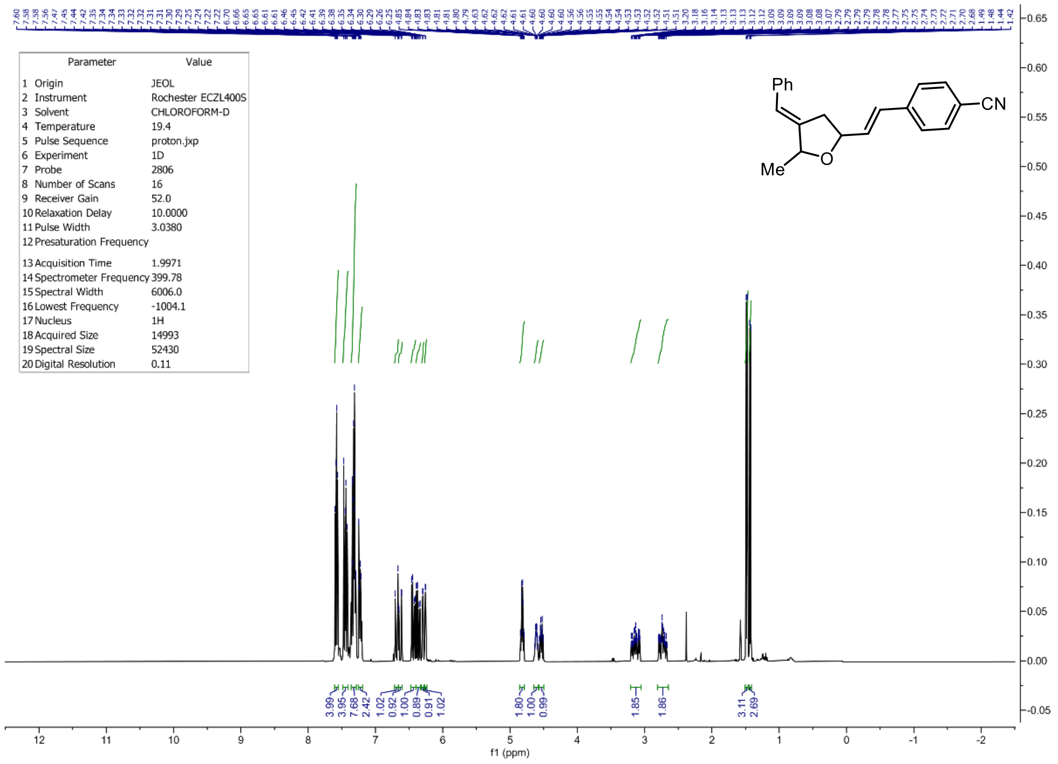

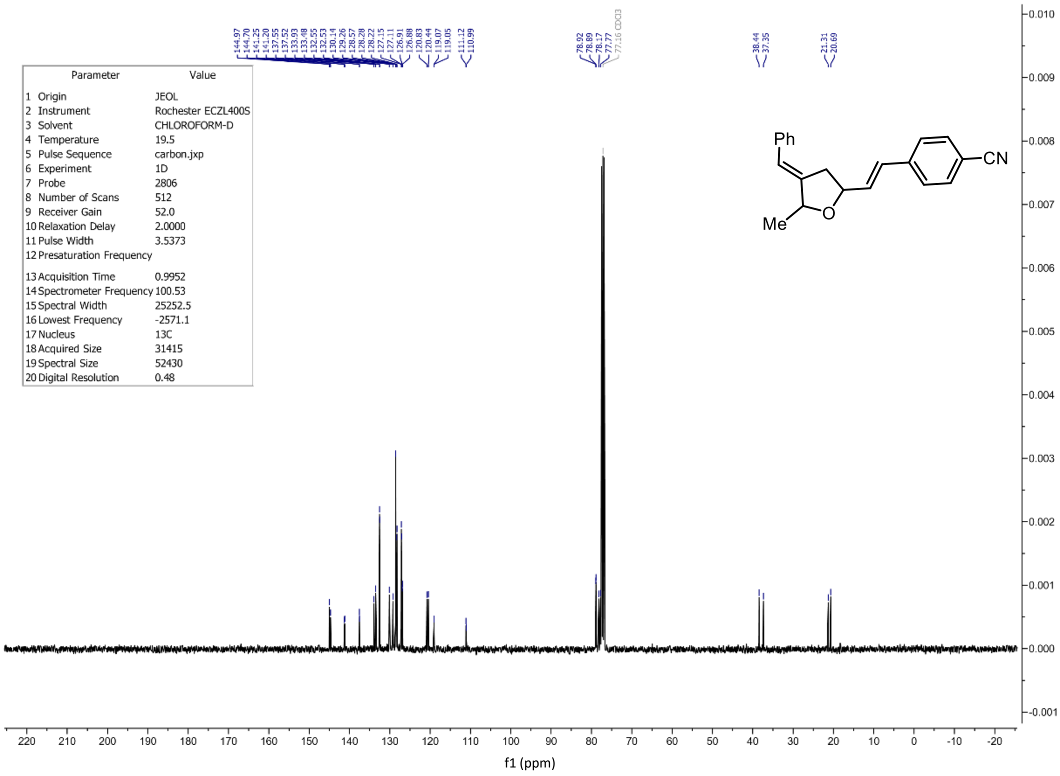

3-((*E*)-benzylidene)-2-methyl-5-((*E*)-3-(trifluoromethyl)styryl)tetrahydrofuran (3ah)

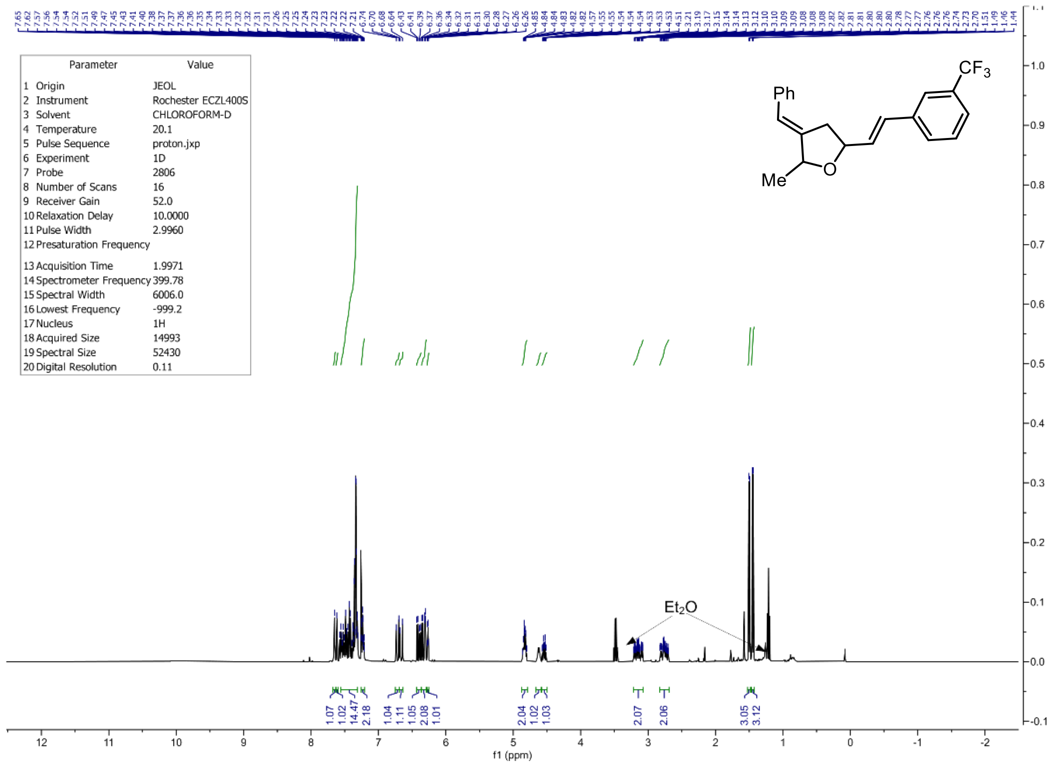

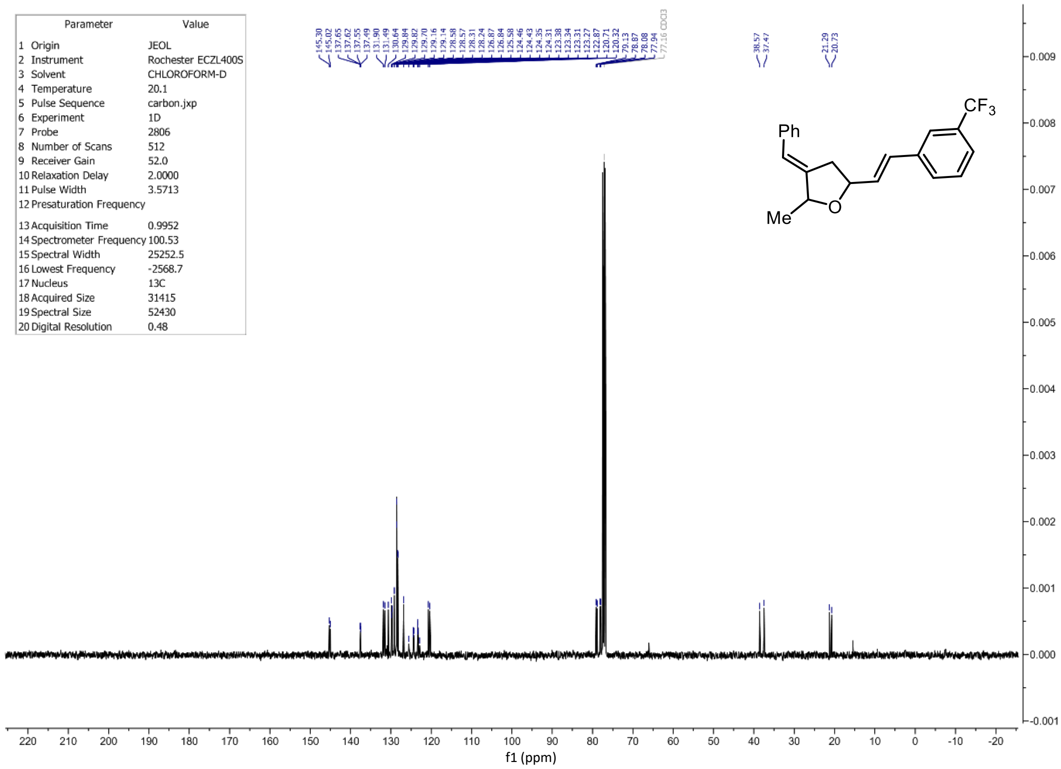

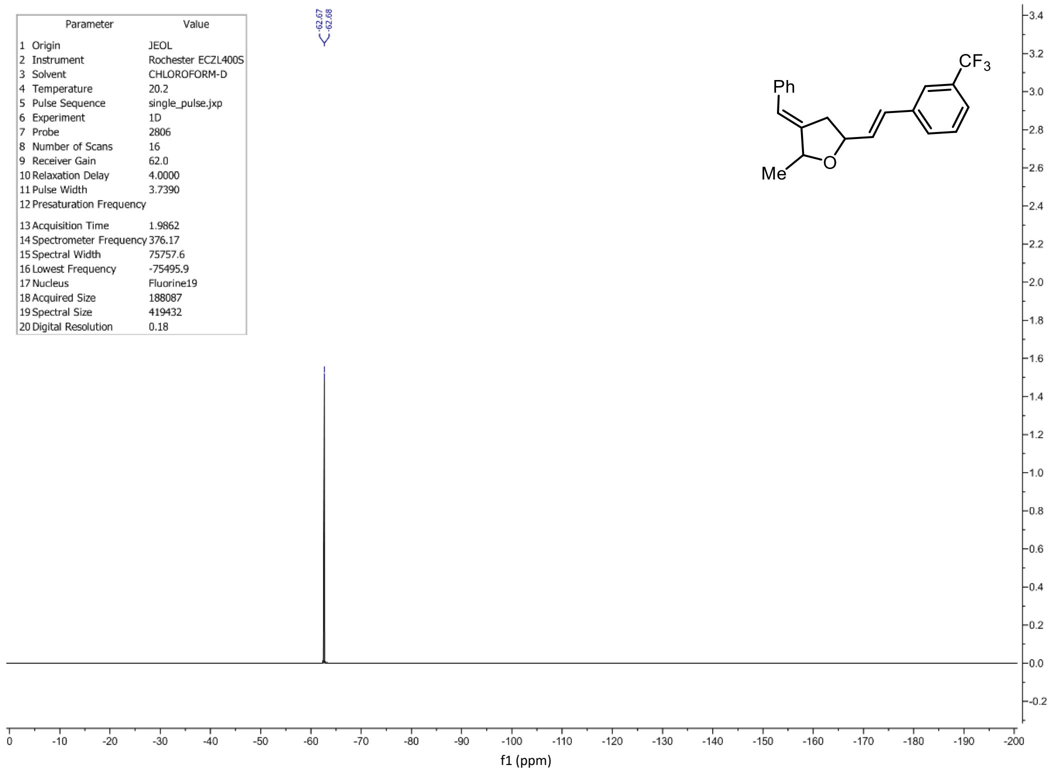

Parameter Value
1 Origin JEOL
2 Instrument Rochester ECZL400S
3 Solvent CHLOROFORM-D
4 Temperature 20.2
5 Pulse Sequence single_pulse.jxp
6 Experiment 1D
7 Probe 2806
8 Number of Scans 16
9 Receiver Gain 62.0
10 Relaxation Delay 4.0000
11 Pulse Width 3.7390
12 Presaturation Frequency
13 Acquisition Time 1.9862
14 Spectrometer Frequency 376.17
15 Spectral Width 75757.6
16 Lowest Frequency -75495.9
17 Nucleus Fluorine19
18 Acquired Size 188087
19 Spectral Size 419432
20 Digital Resolution 0.18
-62.67
-62.68
Ph
Me
O
CF3
f1 (ppm)

3-((*E*)-benzylidene)-5-((*E*)-2-ethylstyryl)-2-methyltetrahydrofuran (3ai)

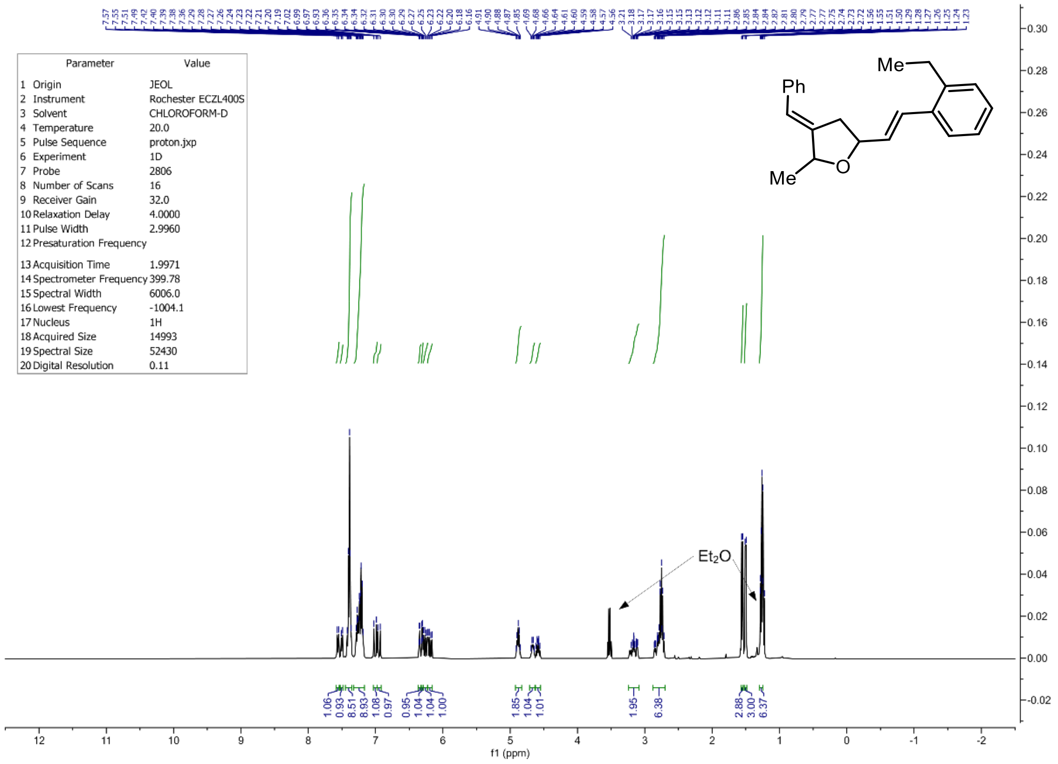

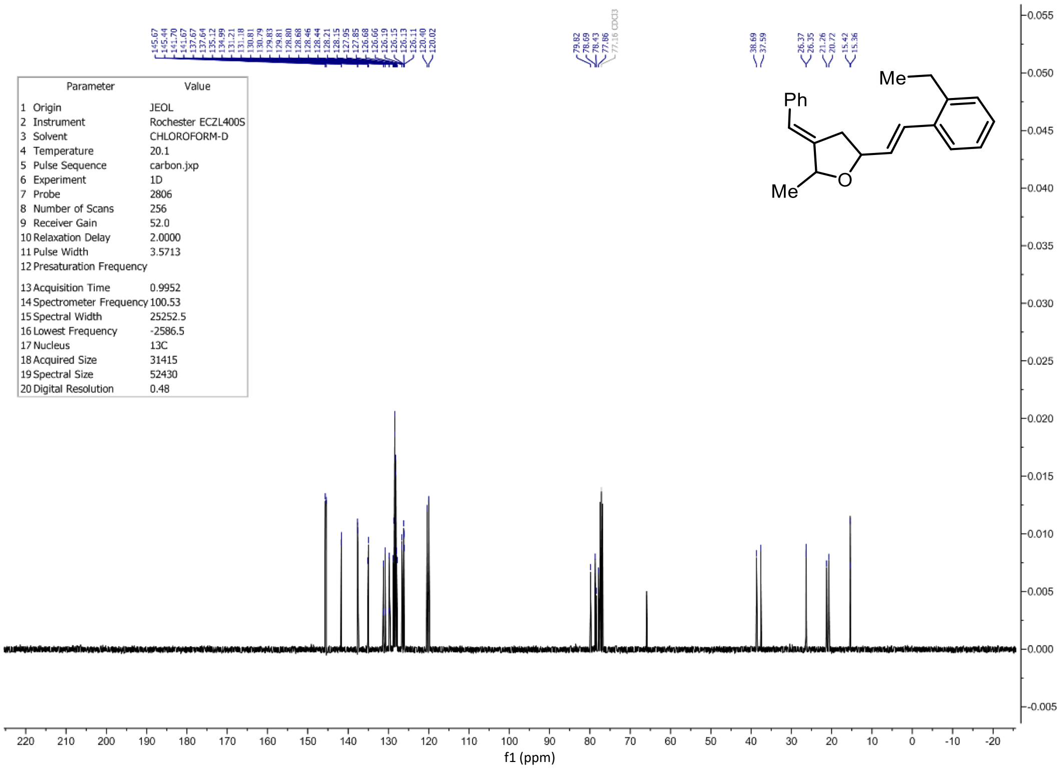

2-((*E*)-2-(4-((*E*)-benzylidene)-5-methyltetrahydrofuran-2-yl)vinyl)furan (3aj)

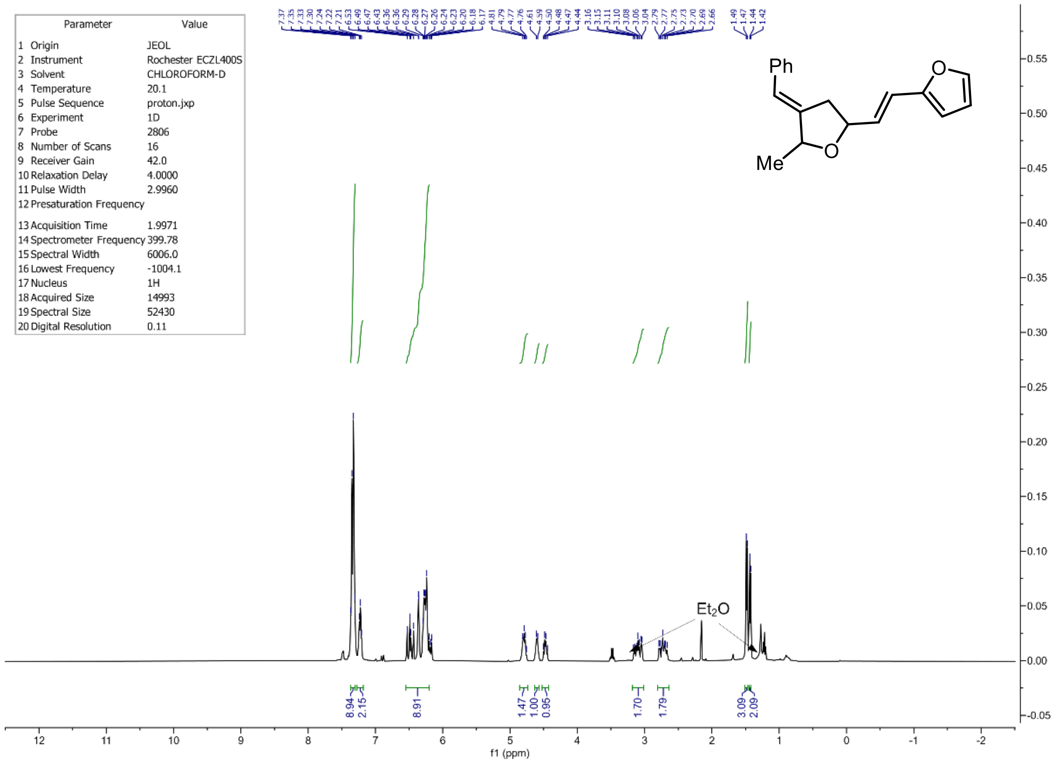

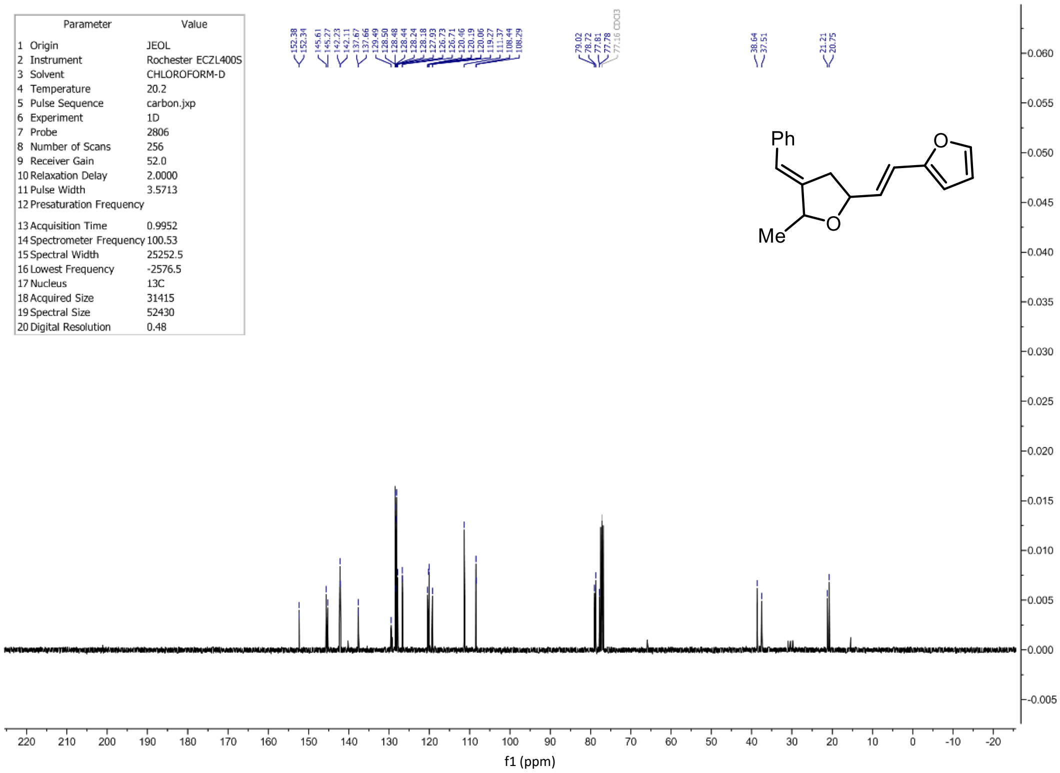

3-((*E*)-benzylidene)-2-methyl-5-((*E*)-2-(thiophen-2-yl)vinyl)tetrahydrofuran (3ak)

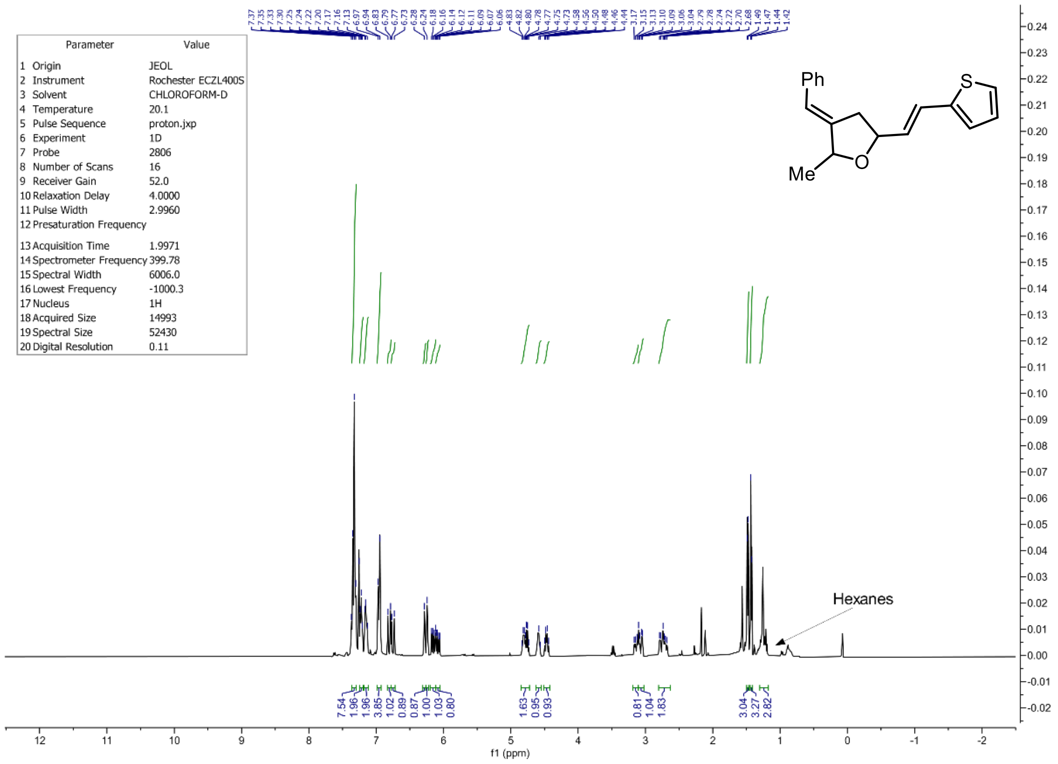

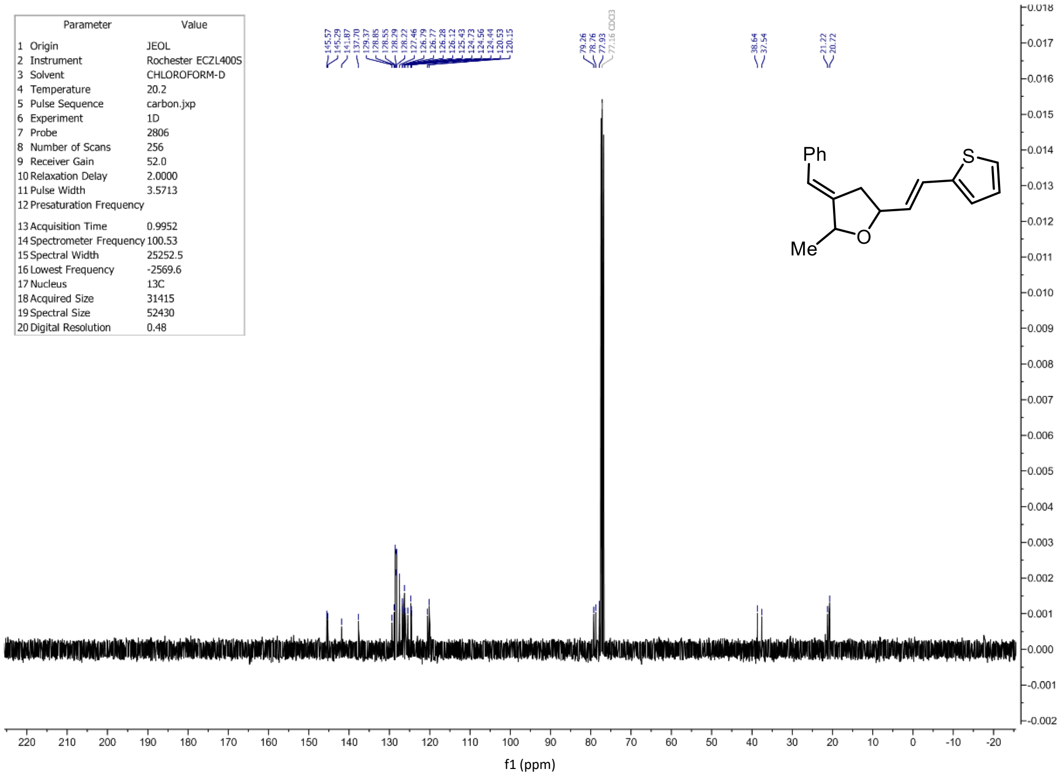

3-((*E*)-benzylidene)-2-methyl-5-((*E*)-2-(thiophen-3-yl)vinyl)tetrahydrofuran (3al)

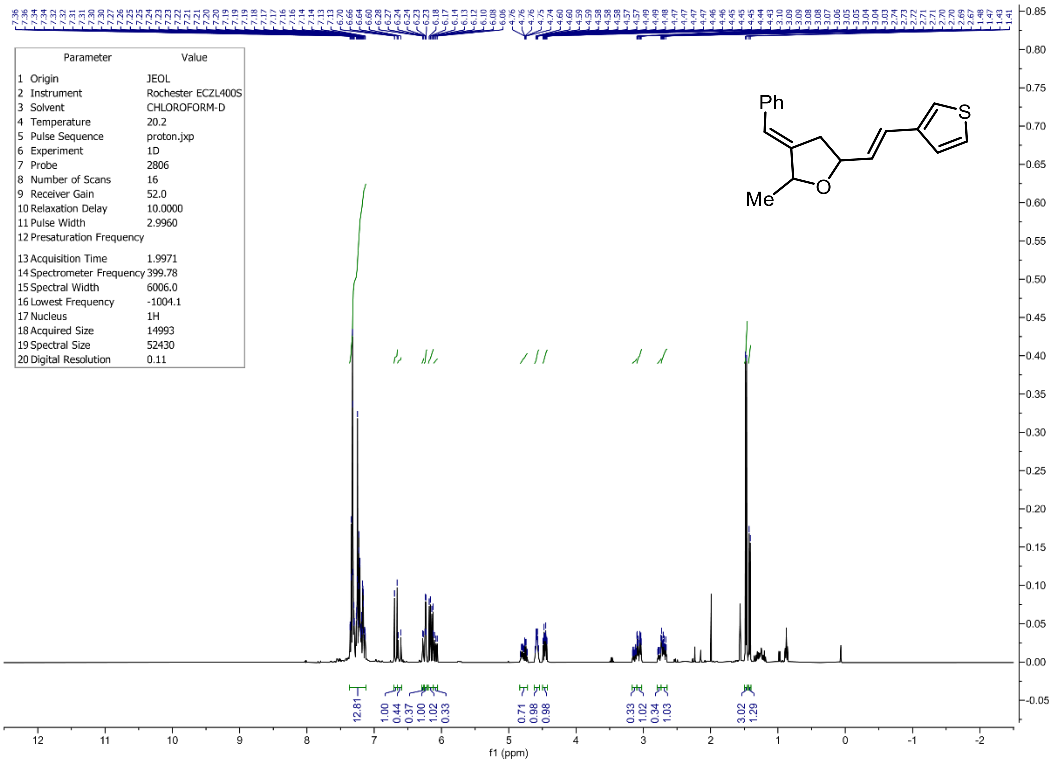

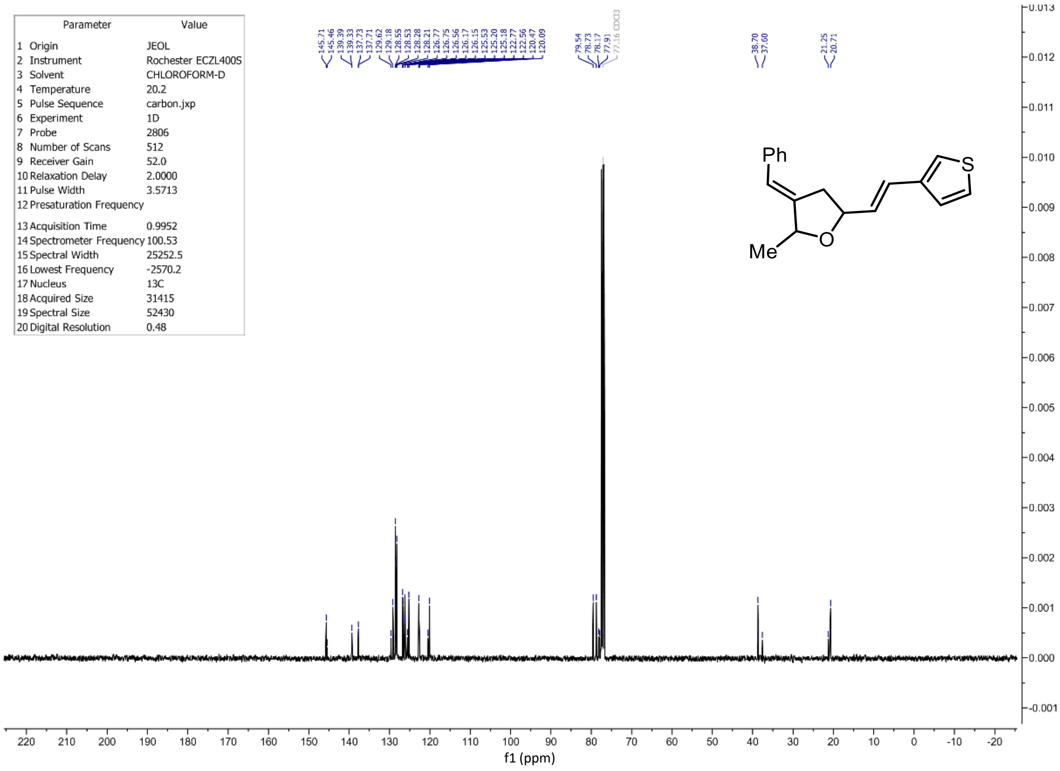

5-((*E*)-2-(4-((*E*)-benzylidene)-5-methyltetrahydrofuran-2-yl)vinyl)benzo[d][1,3]dioxole (3am)

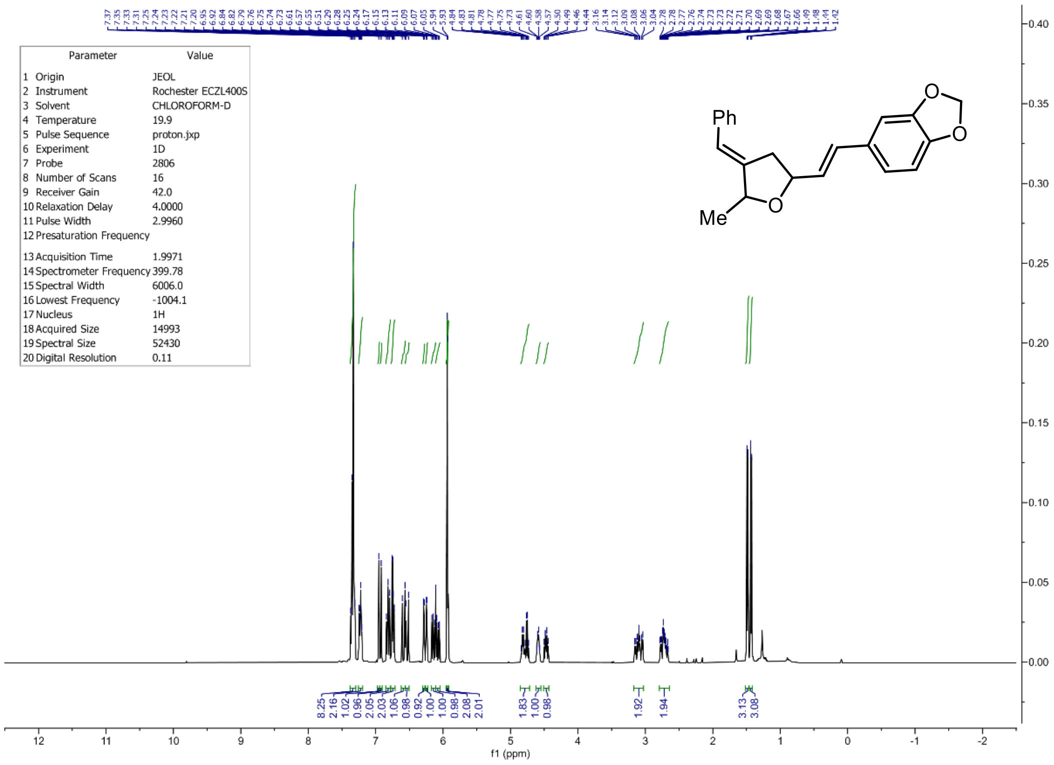

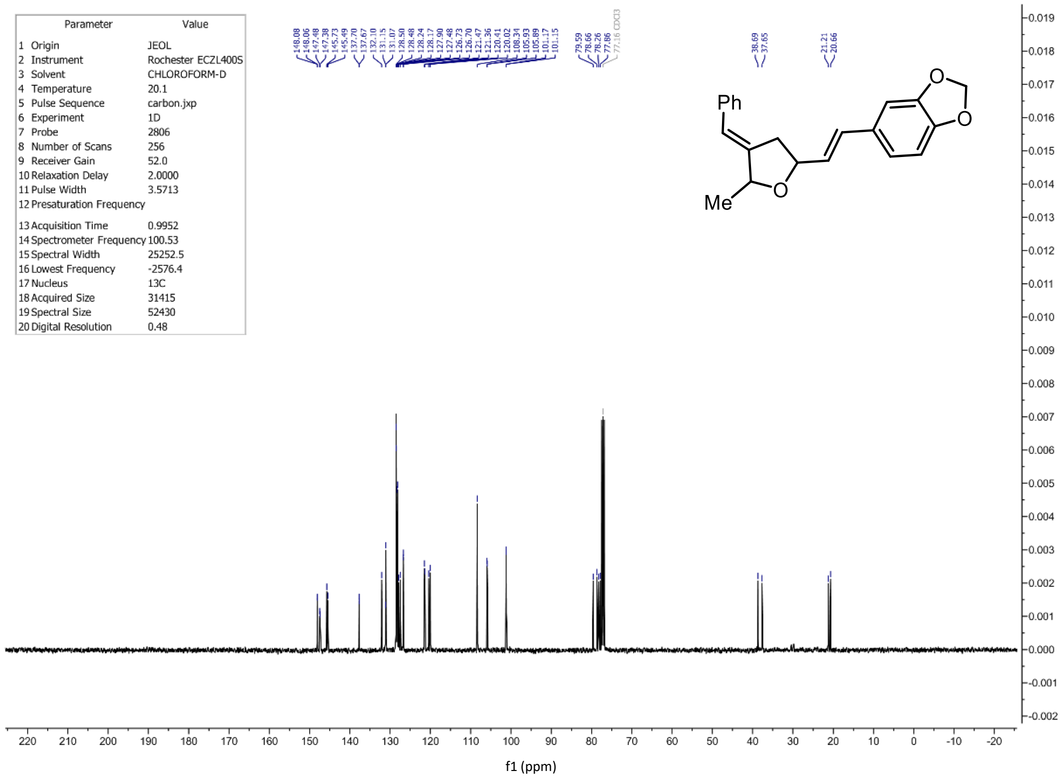

3-((*E*)-2-(4-((*E*)-benzylidene)-5-methyltetrahydrofuran-2-yl)vinyl)pyridine (3an)

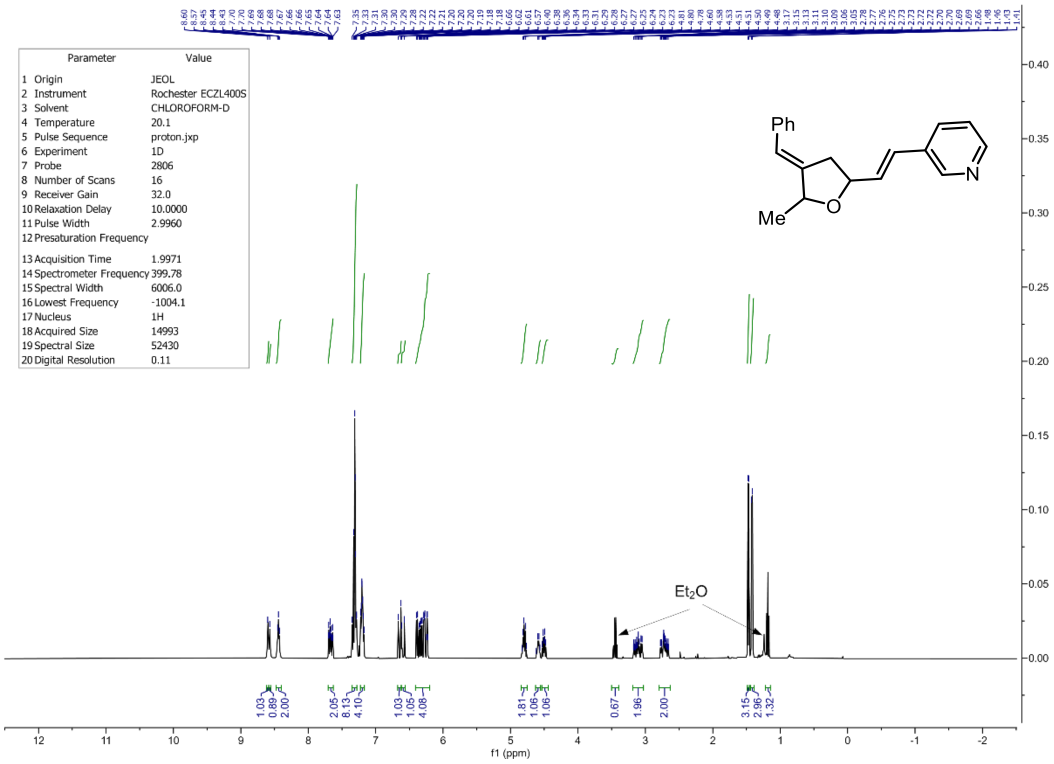

Parameter	Value
1 Origin	JEOL
2 Instrument	Rochester ECZL400S
3 Solvent	CHLOROFORM-D
4 Temperature	20.1
5 Pulse Sequence	proton.jxp
6 Experiment	1D
7 Probe	2806
8 Number of Scans	16
9 Receiver Gain	32.0
10 Relaxation Delay	10.0000
11 Pulse Width	2.9960
12 Presaturation Frequency	
13 Acquisition Time	1.9971
14 Spectrometer Frequency	399.78
15 Spectral Width	6006.0
16 Lowest Frequency	-1004.1
17 Nucleus	1H
18 Acquired Size	14993
19 Spectral Size	52430
20 Digital Resolution	0.11

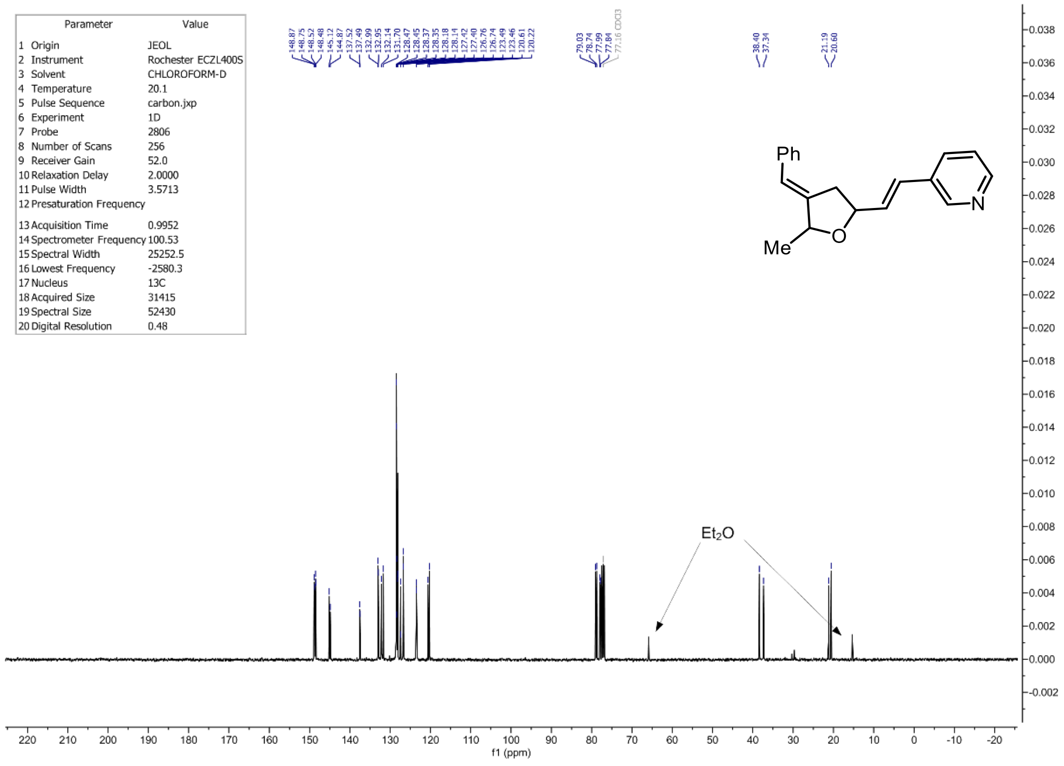

Parameter	Value
1 Origin	JEOL
2 Instrument	Rochester ECZL400S
3 Solvent	CHLOROFORM-D
4 Temperature	20.1
5 Pulse Sequence	carbon.jxp
6 Experiment	1D
7 Probe	2806
8 Number of Scans	256
9 Receiver Gain	52.0
10 Relaxation Delay	2.0000
11 Pulse Width	3.5713
12 Presaturation Frequency	
13 Acquisition Time	0.9952
14 Spectrometer Frequency	100.53
15 Spectral Width	25252.5
16 Lowest Frequency	-2580.3
17 Nucleus	13C
18 Acquired Size	31415
19 Spectral Size	52430
20 Digital Resolution	0.48

3-((*E*)-2-(4-((*E*)-benzylidene)-5-methyltetrahydrofuran-2-yl)vinyl)-1-methyl-1H-indole (3ao)

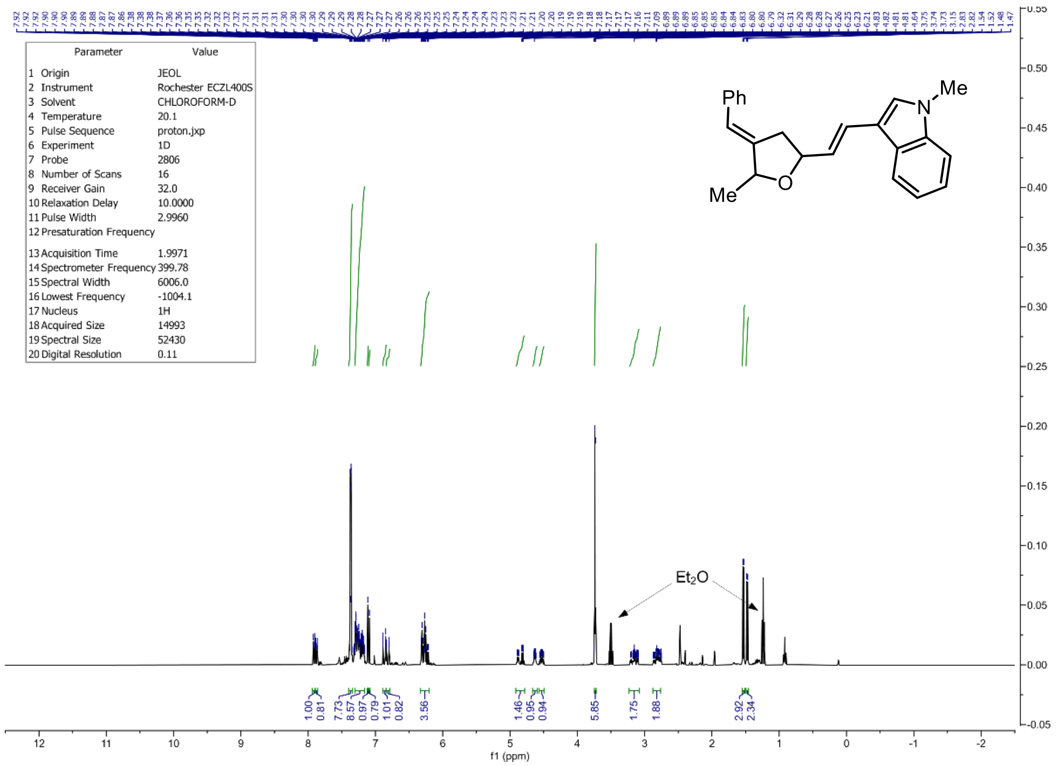

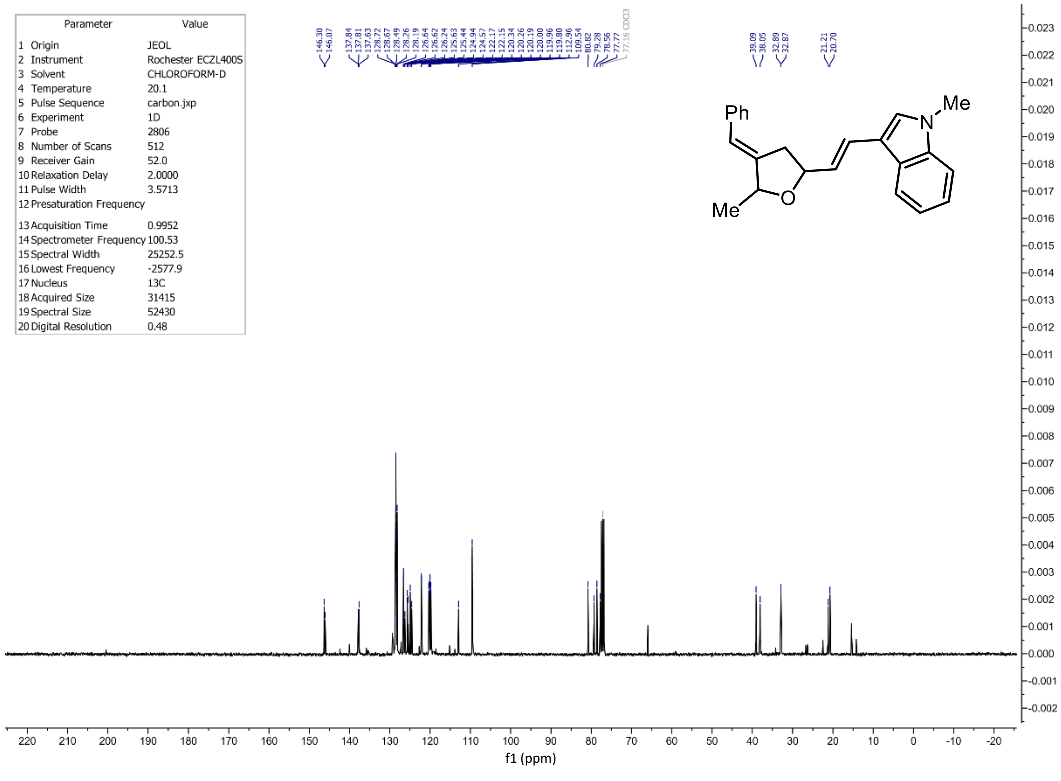

3-((*E*)-benzylidene)-5-((*E*)-hept-1-en-1-yl)-2-methyltetrahydrofuran (3ap)

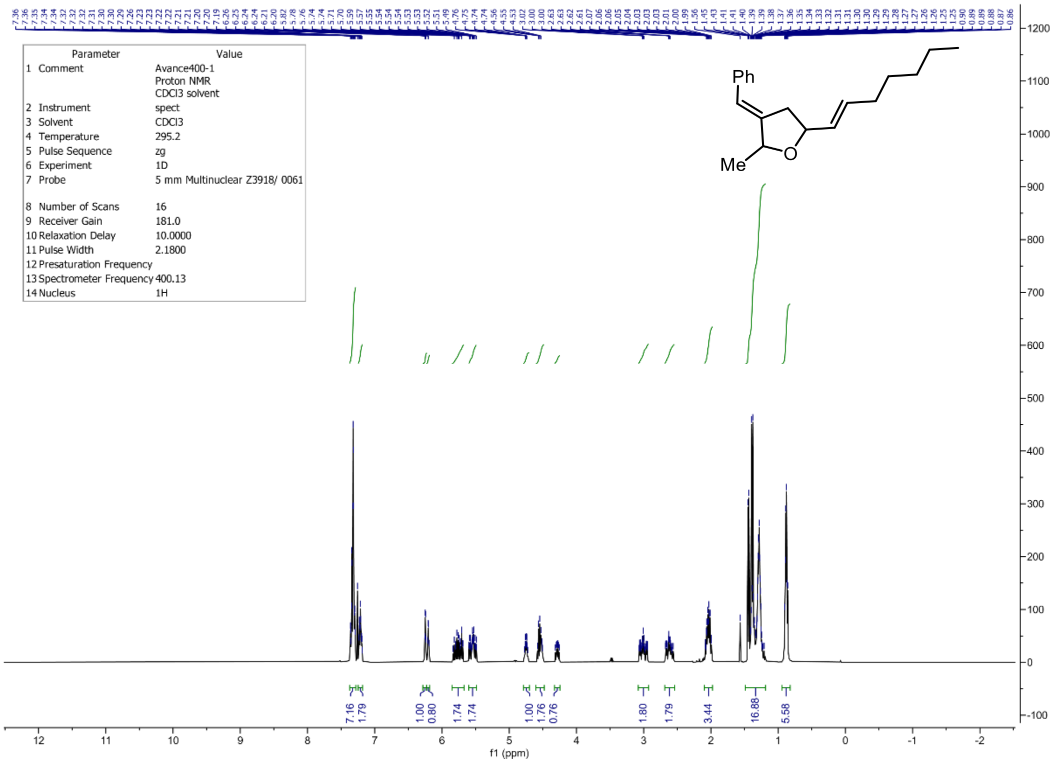

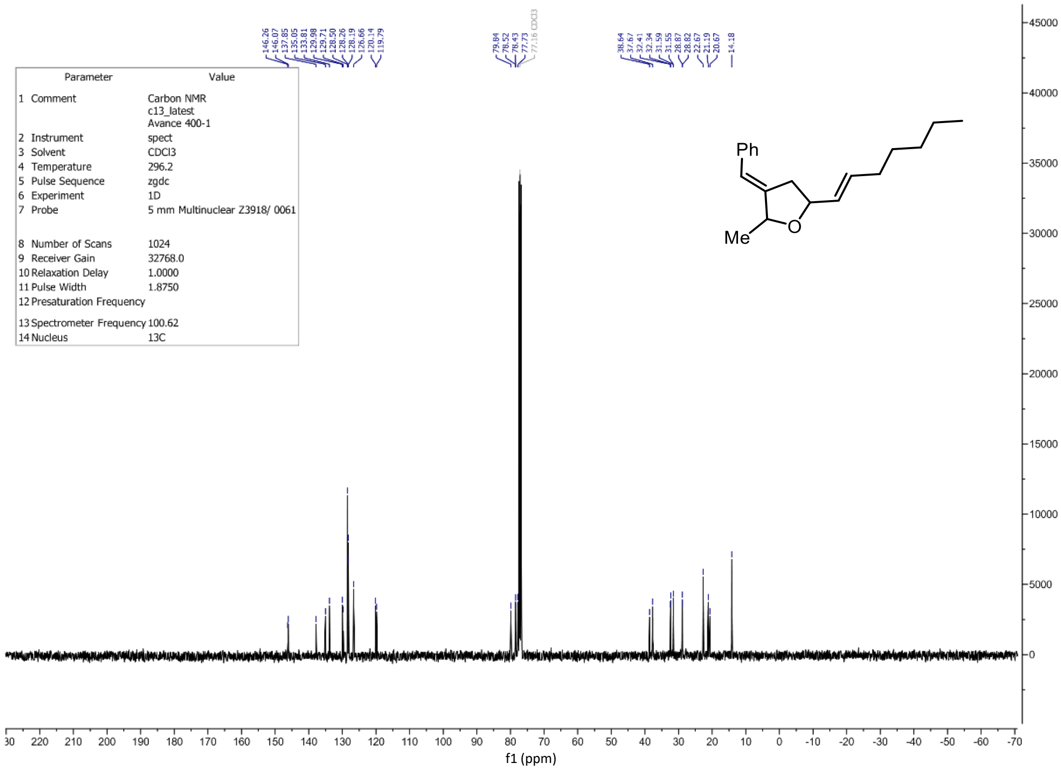

3-((*E*)-benzylidene)-2-methyl-5-((*E*)-4-phenylbut-1-en-1-yl)tetrahydrofuran (3aq)

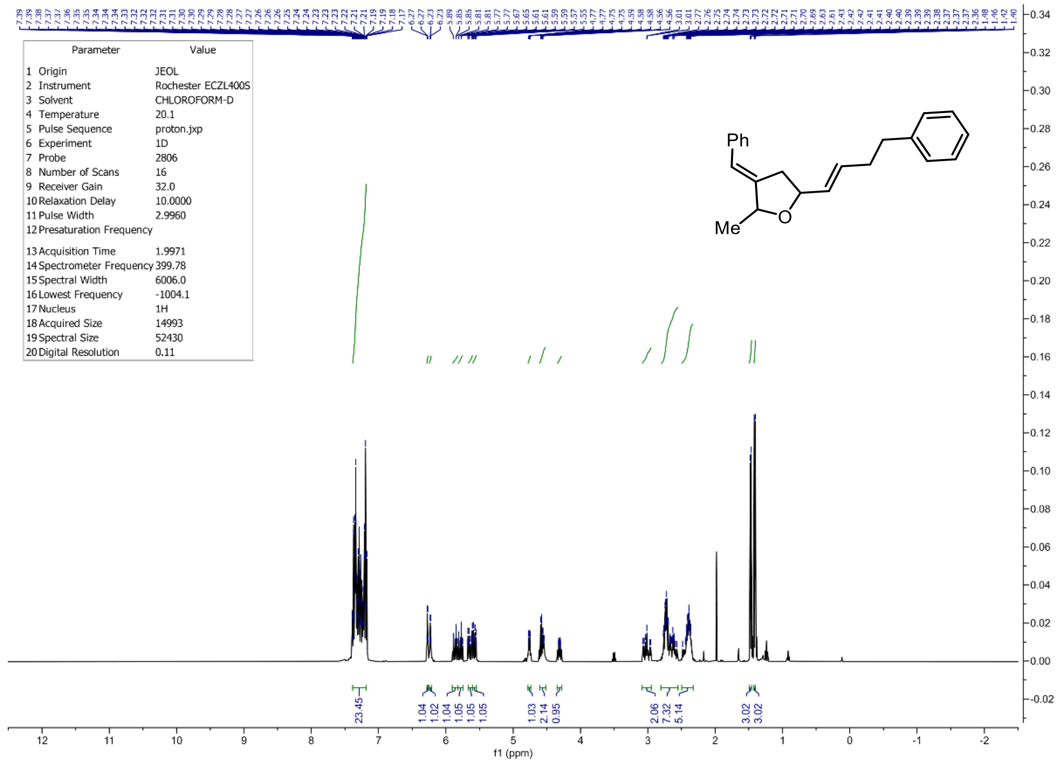

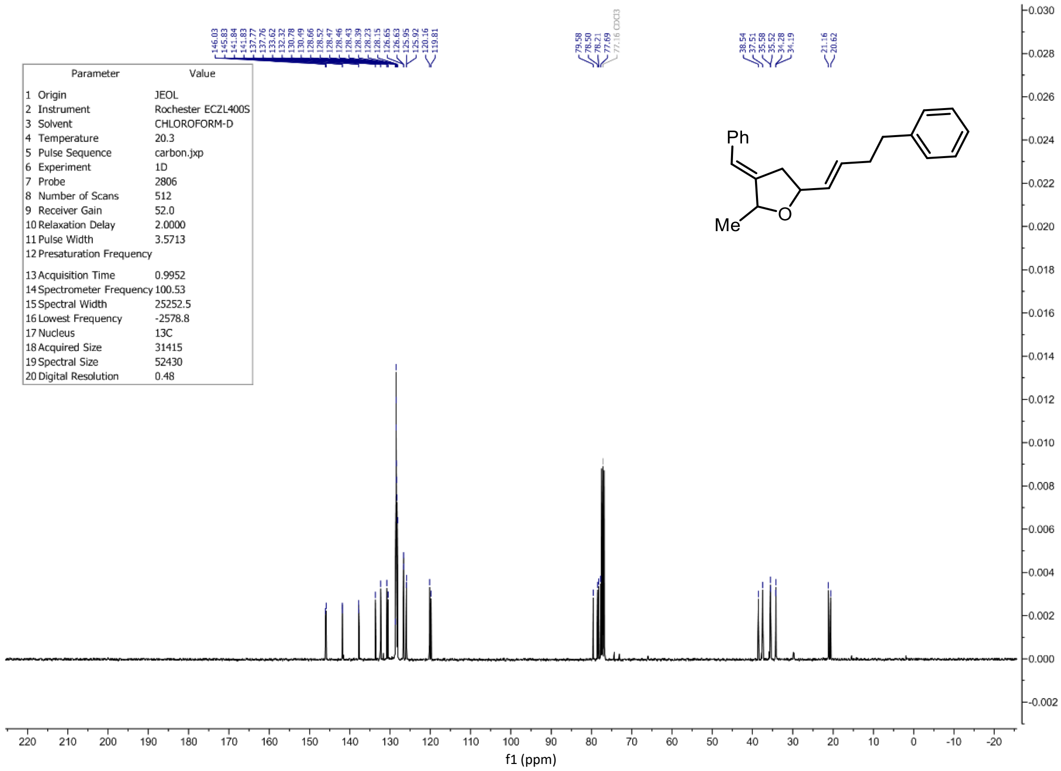

(*E*)-3-benzylidene-2-methyl-5-(1-phenylvinyl)tetrahydrofuran (3ar)

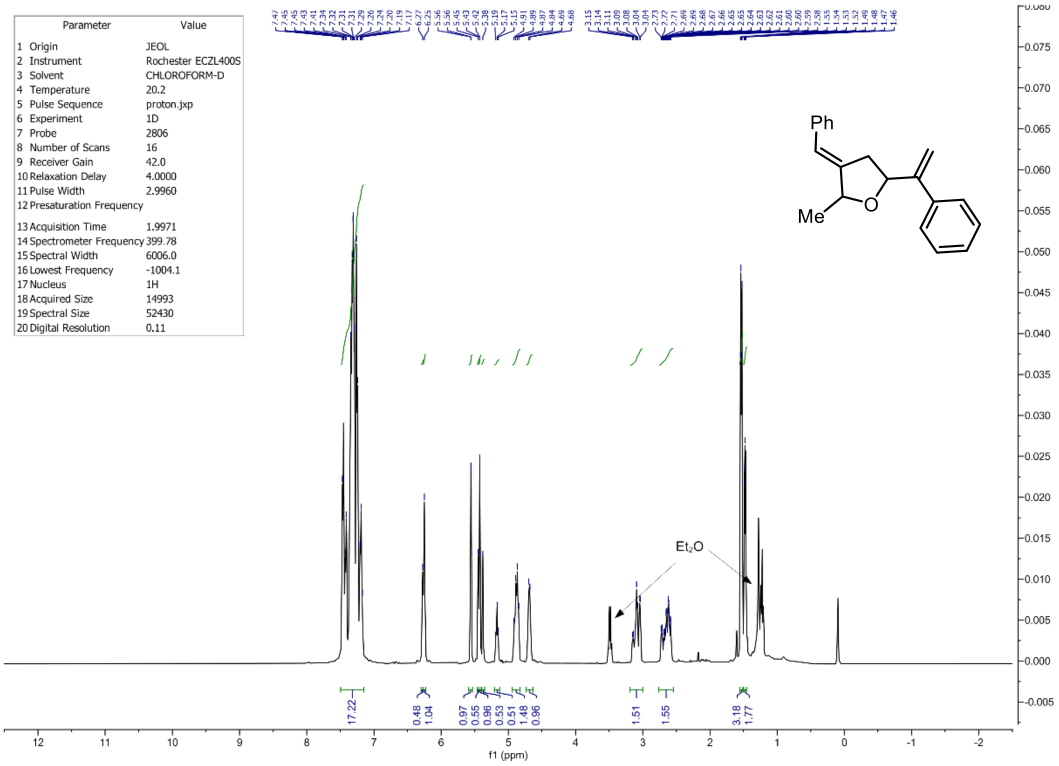

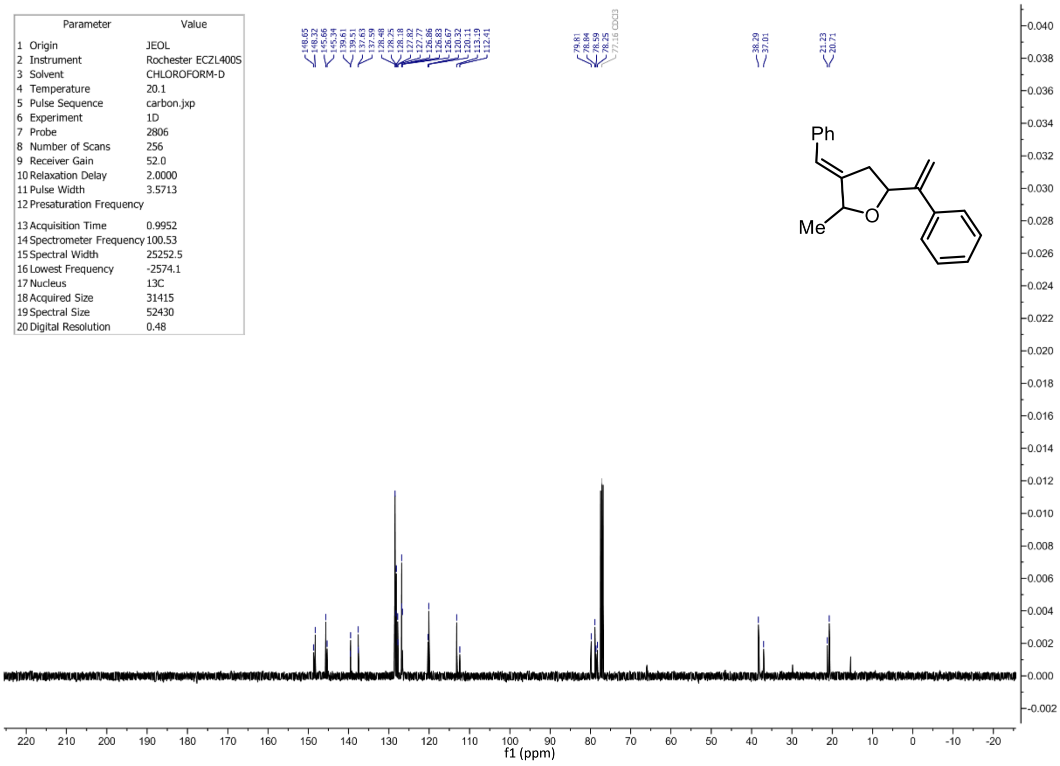

4-((*E*)-benzylidene)-2,5-dimethyl-2-((*E*)-styryl)tetrahydrofuran (3as)

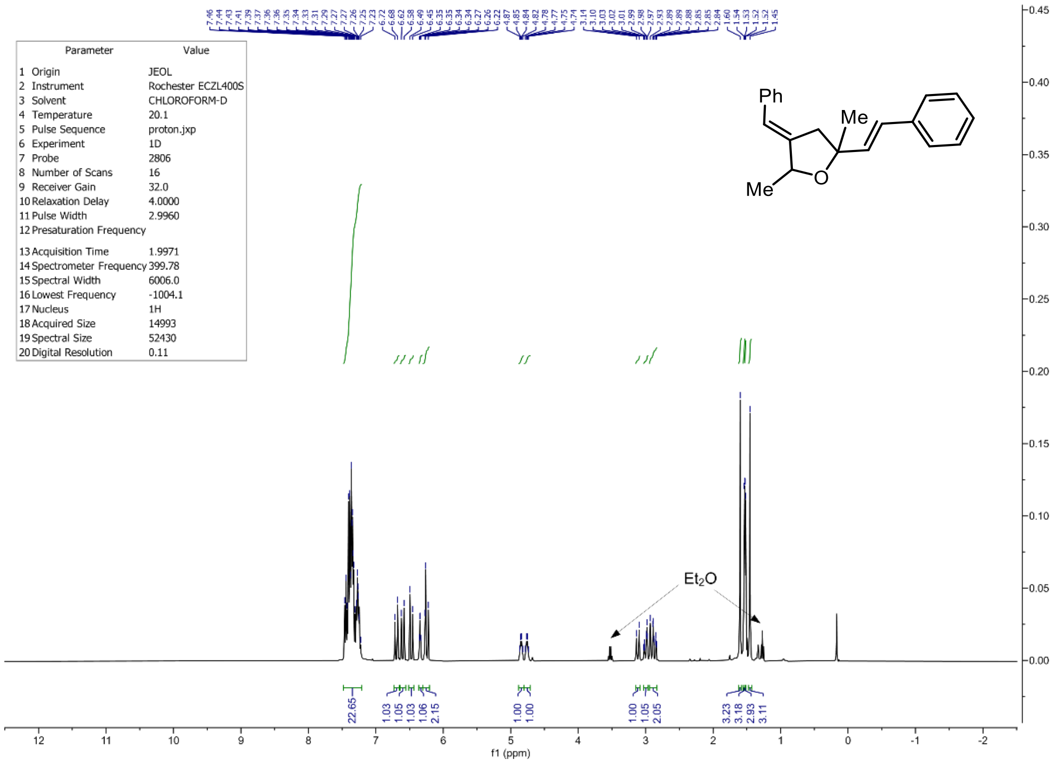

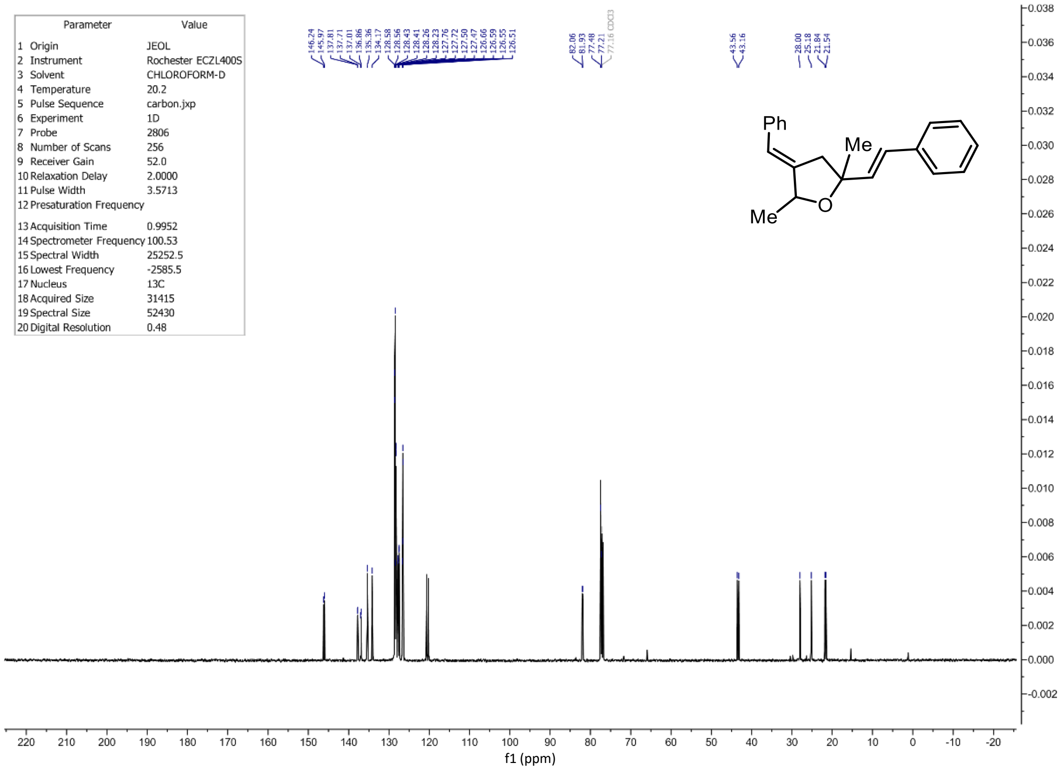

4-((*E*)-benzylidene)-2-((*E*)-hex-1-en-1-yl)-2,5-dimethyltetrahydrofuran (3at)

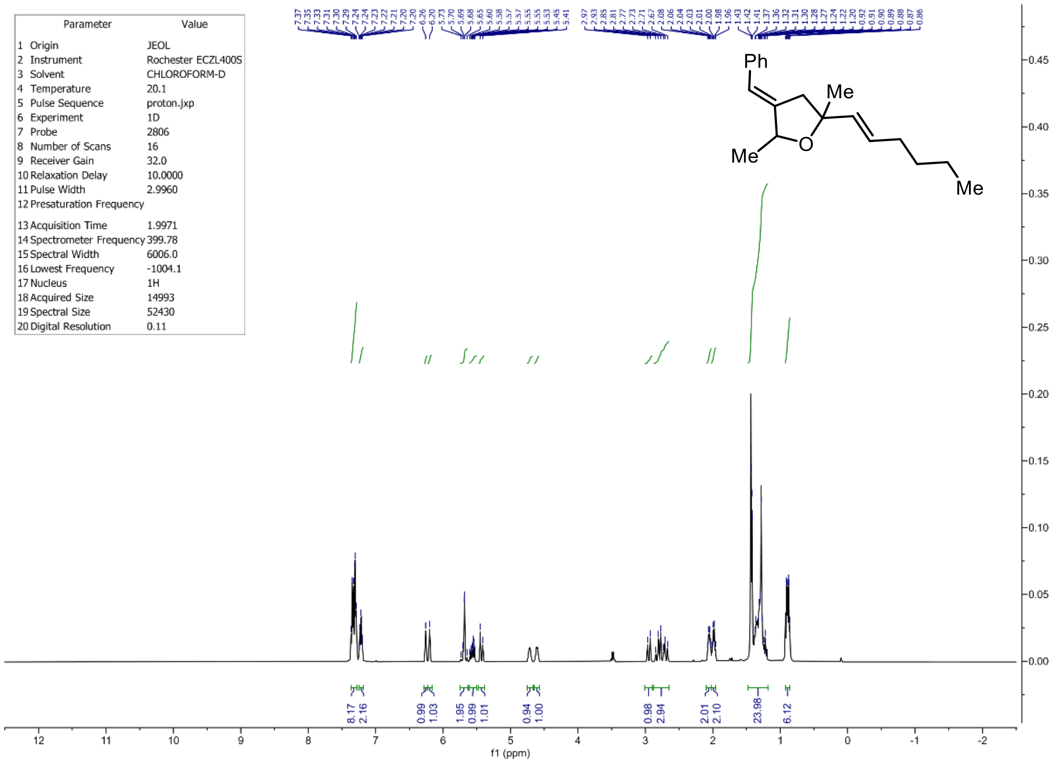

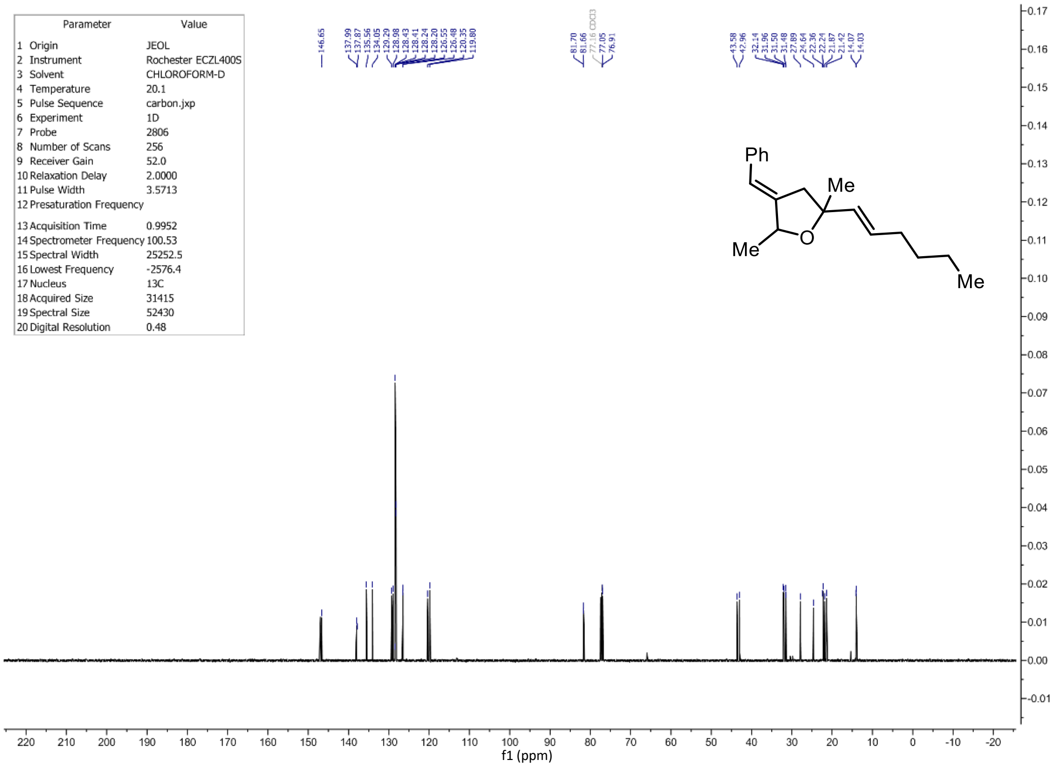

3-((*E*)-benzylidene)-2-methyl-5-((*E*)-1-phenylprop-1-en-2-yl)tetrahydrofuran (3au)

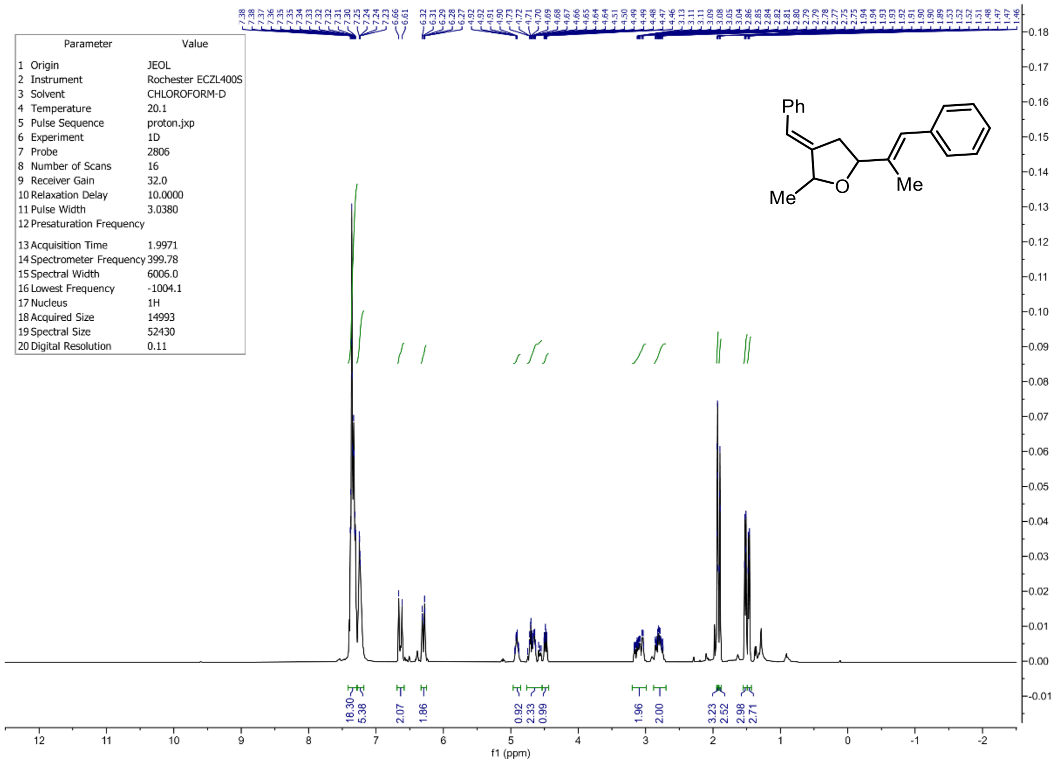

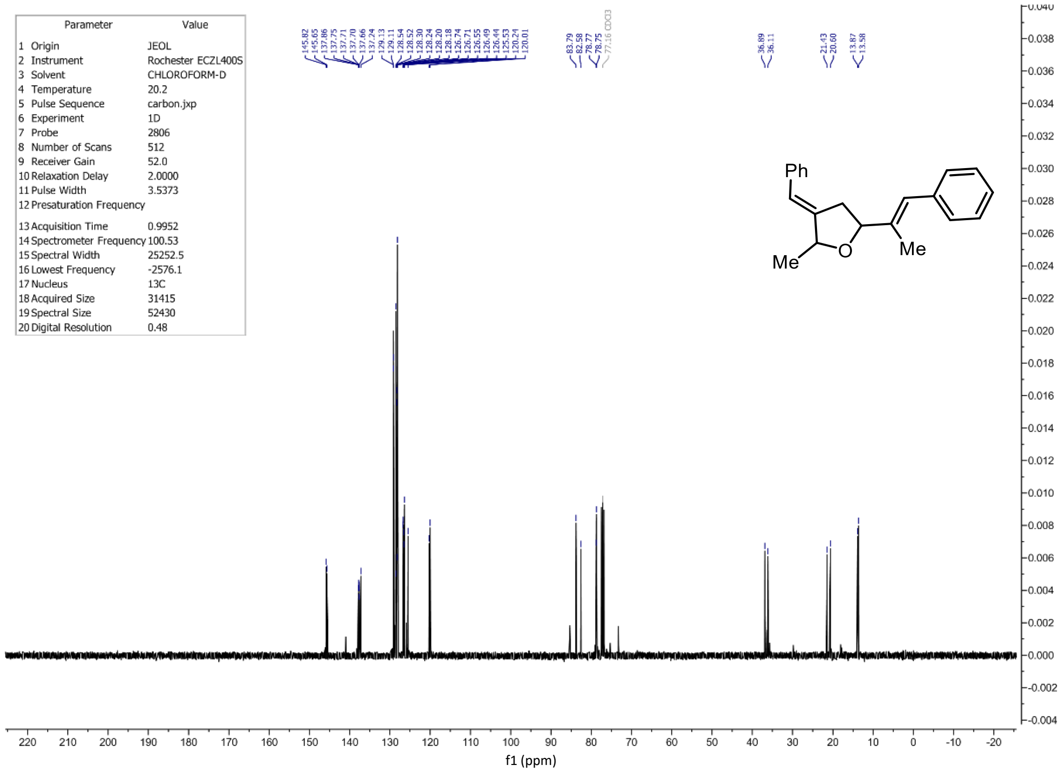

4-((*E*)-benzylidene)-2-((*E*)-hept-1-en-1-yl)tetrahydrofuran (3dp)

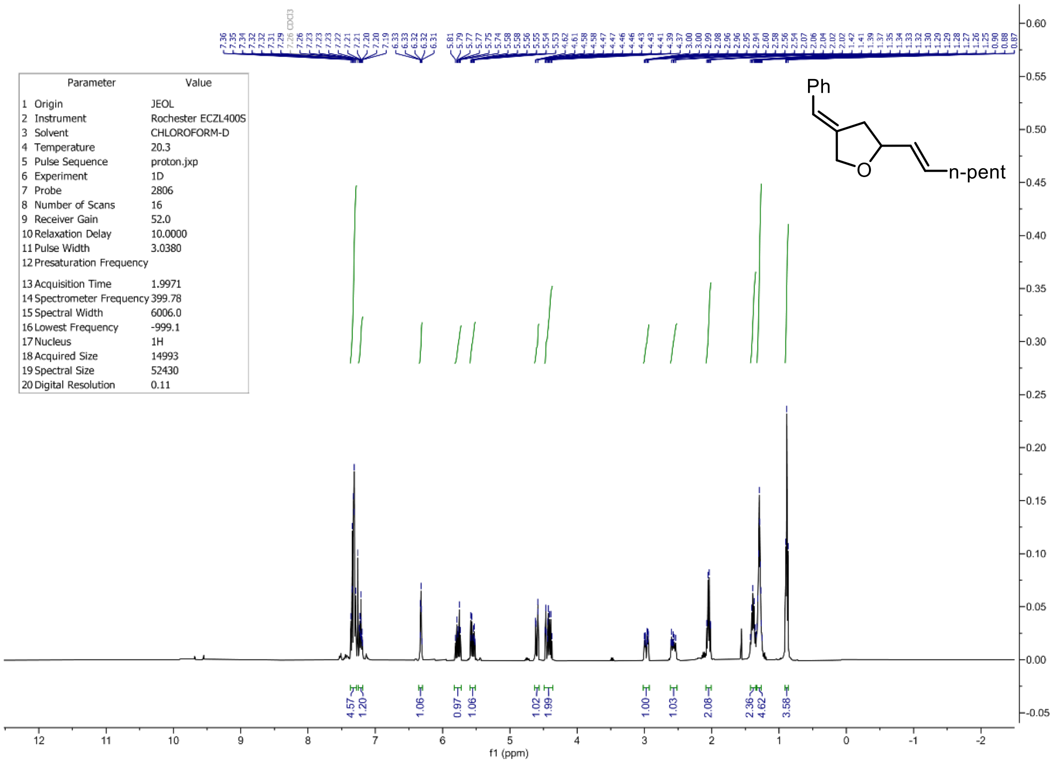

	Parameter	Value
1	Origin	JEOL
2	Instrument	Rochester ECZL400S
3	Solvent	CHLOROFORM-D
4	Temperature	20.3
5	Pulse Sequence	proton.jxp
6	Experiment	1D
7	Probe	2806
8	Number of Scans	16
9	Receiver Gain	52.0
10	Relaxation Delay	10.0000
11	Pulse Width	3.0380
12	Presaturation Frequency	
13	Acquisition Time	1.9971
14	Spectrometer Frequency	399.78
15	Spectral Width	6006.0
16	Lowest Frequency	-999.1
17	Nucleus	1H
18	Acquired Size	14993
19	Spectral Size	52430
20	Digital Resolution	0.11

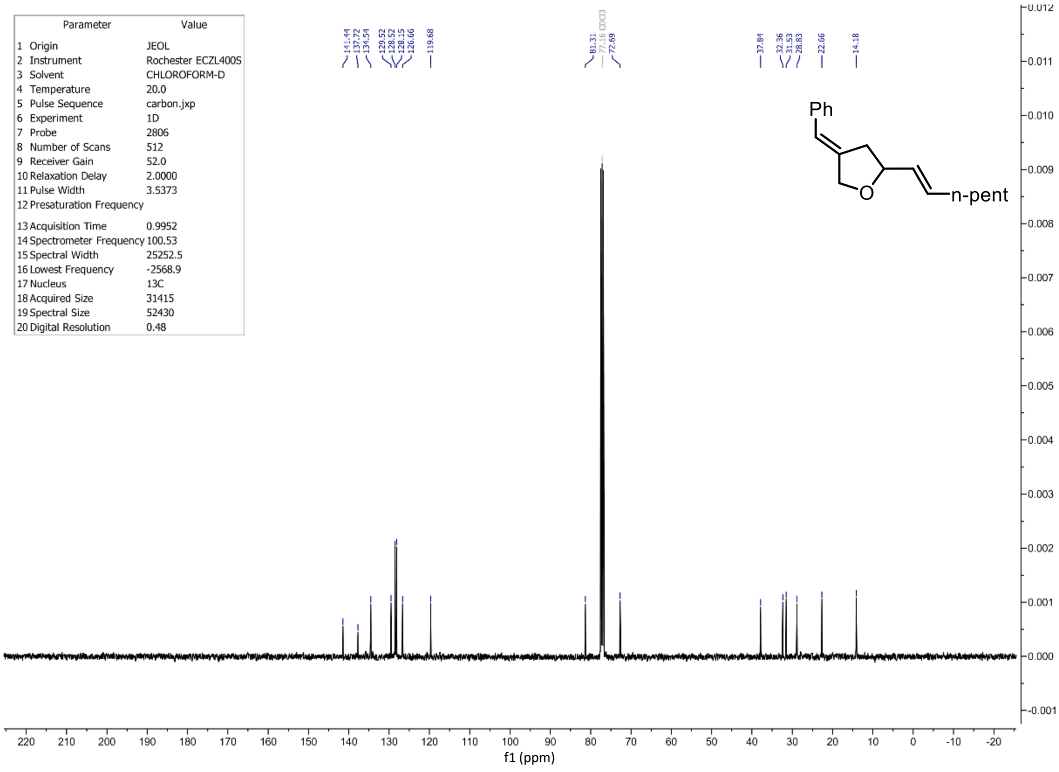

	Parameter	Value
1	Origin	JEOL
2	Instrument	Rochester ECZL400S
3	Solvent	CHLOROFORM-D
4	Temperature	20.0
5	Pulse Sequence	carbon.jxp
6	Experiment	1D
7	Probe	2806
8	Number of Scans	512
9	Receiver Gain	52.0
10	Relaxation Delay	2.0000
11	Pulse Width	3.5373
12	Presaturation Frequency	
13	Acquisition Time	0.9952
14	Spectrometer Frequency	100.53
15	Spectral Width	25252.5
16	Lowest Frequency	-2568.9
17	Nucleus	13C
18	Acquired Size	31415
19	Spectral Size	52430
20	Digital Resolution	0.48

(*E*)-5-(hept-1-en-1-yl)tetrahydrofuran-3-ol (4)

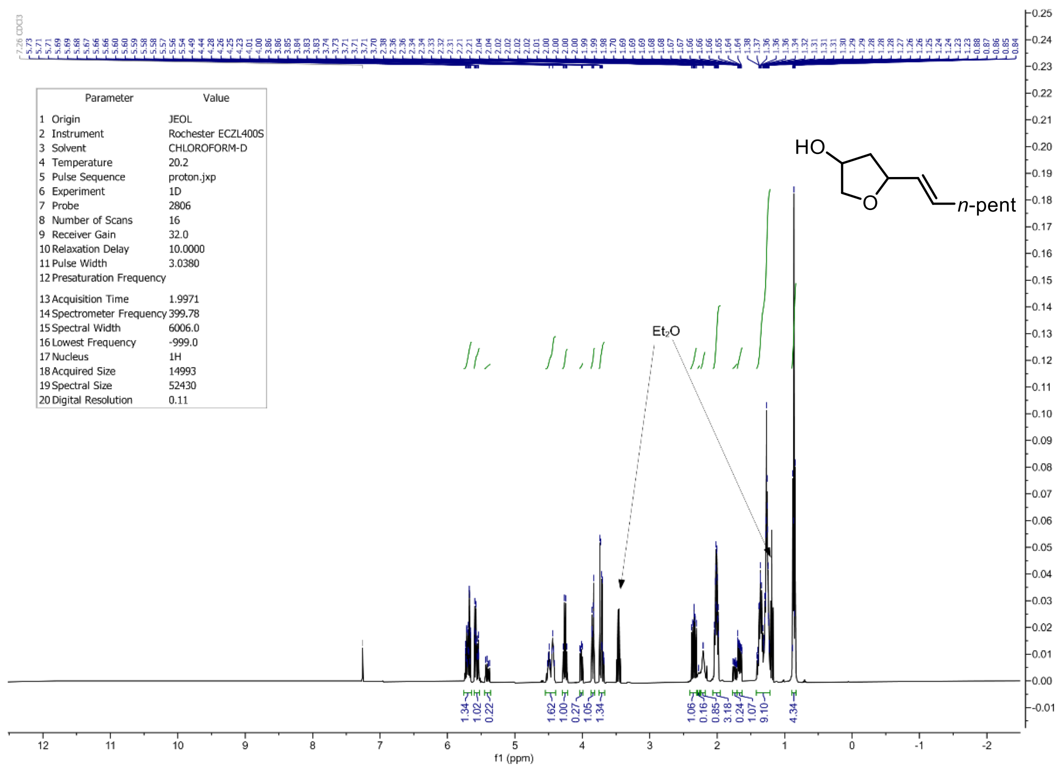

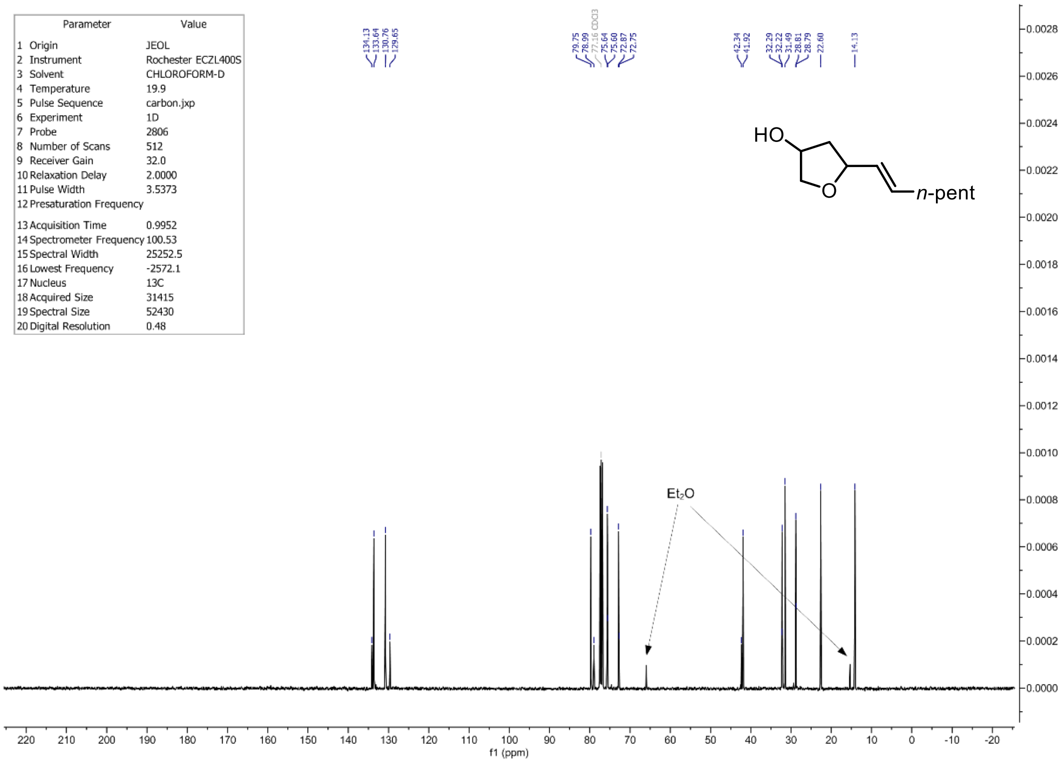

References

1 D. Kobus-Bartoszewicz and S. Stecko, Synthesis of Chiral a-Amino Ketones via Transition Metal Catalyzed or Photoredox Cross-Coupling and Olefin Photo-Cleavage Reaction Sequence, *Adv. Synth. Catal.*, **2023**, *365*, 1224-1237.

2 R. Moser, Z. V. Boskovic, C. S. Crowe and B. H. Lipshutz, CuH-catalyzed enantioselective 1,2-reductions of alpha,beta-unsaturated ketones, *J. Am. Chem. Soc.*, **2010**, *132*, 7852-7853.

3 S. Liu, P. Cui, J. Wang, H. Zhou, Q. Liu and J. Lv, Asymmetric transfer hydrogenation of cycloalkyl vinyl ketones to allylic alcohols catalyzed by ruthenium amido complexes, *Org. Biomol. Chem.*, **2019**, *17*, 264-267.

4 V. M. Muzalevskiy, Y. A. Ustynyuk, I. P. Gloriozov, V. A. Chertkov, A. Y. Rulev, E. V. Kondrashov, I. A. Ushakov, A. R. Romanov and V. G. Nenajdenko, Experimental and Theoretical Study of an Intramolecular CF3-Group Shift in the Reactions of alpha-Bromoenones with 1,2-Diamines, *Chem. Eur. J.*, **2015**, *21*, 16982-16989.

5 D. Wang, X. Ye and X. Shi, Efficient Synthesis of E-α-Haloenones Through Chemoselective Alkyne Activation Over Allene with Triazole−Au Catalysts, *Org. Lett.*, **2010**, *12*, 2088-2091.

6 W. R. Bowman, C. F. Bridge, P. Brookes, M. O. Cloonan and D. C. Leach, Cascade radical synthesis of heteroarenes iminyl radicals, *J. Chem. Soc., Perkin Trans. 1*, **2002**, DOI: 10.1039/b108323f, 58-68.

7 J. Singh, V. Saini, A. Kumar and R. Bansal, Synthesis, molecular docking and biological evaluation of some newer 2-substituted-4-(benzo[d][1,3]dioxol-5-yl)-6-phenylpyridazin-3(2H)-ones as potential anti-inflammatory and analgesic agents, *Bioorg. Chem.*, **2017**, *71*, 201-210.

8 D. Wang, Y. Zhang, A. Harris, L. N. S. Gautam, Y. Chen and X. Shi, Triazole-Gold-Promoted, Effective Synthesis of Enones from Propargylic Esters and Alcohols: A Catalyst Offering Chemoselectivity, Acidity and Ligand Economy, *Adv. Synth. Catal.*, **2011**, *353*, 2584-2588.

9 M. Vuagnoux-d'Augustin and A. Alexakis, Influence of the Double-Bond Geometry of the Michael Acceptor on Copper-Catalyzed Asymmetric Conjugate Addition, *Eur. J. Org. Chem.*, **2007**, 5852-5860.

10 S.-L. Zhang and Z.-Q. Deng, Copper-catalyzed retro-aldol reaction of β-hydroxy ketones or nitriles with aldehydes: chemo- and stereoselective access to (E)-enones and (E)-acrylonitriles, *Org. Biomol. Chem.*, **2016**, *14*, 7282-7294.

11 H. Li, M.-L. Wang, Y.-W. Liu, L.-J. Li, H. Xu and H.-X. Dai, Enones as Alkenyl Reagents via Ligand-Promoted C–C Bond Activation, *ACS Catal.*, **2022**, *12*, 82-88.

12 M. Banwell and C. Cowden, Convergent Routes to the [1,3]Dioxolo[4,5-j]phenanthridin-6(5H)-one and 2,3,4,4a-Tetrahydro[1,3]dioxolo[4,5-j]phenanthridin-6(5H)-one Nuclei. Application to Syntheses of the Amaryllidaceae Alkaloids Crinasiadine, N-Methylcrinasiadine and Trisphaeridine, *Aust. J. Chem.*, **1994**, *47*, 2235-2254.

13 M. H. Gieuw, S. Chen, Z. Ke, K. N. Houk and Y.-Y. Yeung, Boron tribromide as a reagent for anti-Markovnikov addition of HBr to cyclopropanes, *Chem. Sci.*, **2020**, *11*, 9426-9433.

14 T. W. Bentley, S. J. Norman, R. Kemmer and M. Christl, Synthesis and Solvolysis of Bicyclo[3.1.1]Hept-3-En-2-Yl, Bicyclo[3.1.1]Hept-2-Yl, and 2-Halogenocyclohex-2-En-1-Yl Methanesulfonates and P-Nitrobenzoates, *Liebigs Ann. Chem.*, **1995**, 599-608.

15 A. R. White, R. A. Kozlowski, S.-C. Tsai and C. D. Vanderwal, A Direct Synthesis of Highly Substituted π-Rich Aromatic Heterocycles from Oxetanes, *Angew. Chem. Int. Ed.*, **2017**, *56*, 10525-10529.

16 M. E. Krafft and T. F. N. Haxell, Organomediated Morita−Baylis−Hillman Cyclization Reactions, *J. Am. Chem. Soc.*, **2005**, *127*, 10168-10169.

17 J. Vaith, D. Rodina, G. C. Spaulding and S. M. Paradine, Pd-Catalyzed Heteroannulation Using N-Arylureas as a Sterically Undemanding Ligand Platform, *J. Am. Chem. Soc.*, **2022**, *144*, 6667-6673.

18 N. Yasukawa, H. Yokoyama, M. Masuda, Y. Monguchi, H. Sajiki and Y. Sawama, Highly-functionalized arene synthesis based on palladium on carbon-catalyzed aqueous dehydrogenation of cyclohexadienes and cyclohexenes, *Green Chem.*, **2018**, *20*, 1213-1217.

19 R. Riveiros, L. Saya, J. P. Sestelo and L. A. Sarandeses, Palladium-catalysed cross-coupling reactions of triorganoindium reagents with alkenyl halides, *Eur. J. Org. Chem.*, **2008**, 1959-1966.

20 R. A. Fernandes, N. Chandra, A. J. Gangani and G. N. Khatun, Palladium-Catalyzed Regioselective Intermolecular Hydroalkoxylation of 1-Arylbutadienes, *J. Org. Chem.*, **2023**, *88*, 10339-10354.

21 W. Wang, S. He, Y. Zhong, J. Chen, C. Cai, Y. Luo and Y. Xia, Cobalt-Catalyzed Z to E Geometrical Isomerization of 1,3-Dienes, *J. Org. Chem.*, **2022**, *87*, 4712-4723.

22 H. Liu, H. Yuan and X. Shi, Synthesis of nickel and palladium complexes with diarylamido-based unsymmetrical pincer ligands and application for norbornene polymerization, *Dalton Trans.*, **2019**, *48*, 609-617.

23 V. T. Nguyen, H. T. Dang, H. H. Pham, V. D. Nguyen, C. Flores-Hansen, H. D. Arman and O. V. Larionov, Highly Regio- and Stereoselective Catalytic Synthesis of Conjugated Dienes and Polyenes, *J. Am. Chem. Soc.*, **2018**, *140*, 8434-8438.

24 A. Zhang, H. Zhang, T. Jin, L. Ge, X. Ma, J. Tang, J. Liu, C. H. Tan, R. Lee and Y. Ge, Stereoconvergent Access to Z-Allylborons from E/Z-Mixed 1,3-Dienes via Cu-Guanidine Catalysis, *Adv. Synth. Catal.*, **2024**, *367*, e202401322.

25 K. S. Madden, S. David, J. P. Knowles and A. Whiting, Heck-Mizoroki coupling of vinyliodide and applications in the synthesis of dienes and trienes, *Chem. Commun.*, **2015**, *51*, 11409-11412.

26 A. Yadav, S. Sandha and C. B. Tripathi, Organocatalytic photoinduced carboamination of dienes, *Chem. Commun.*, **2023**, *59*, 5579-5582.

27 E. Davenport and E. Fernandez, Transition-metal-free synthesis of vicinal triborated compounds and selective functionalisation of the internal C-B bond, *Chem. Commun.*, **2018**, *54*, 10104-10107.

28 P. Fourgeaud, C. Midrier, J. P. Vors, J. N. Volle, J. L. Pirat and D. Virieux, Oxaphospholene and oxaphosphinene heterocycles via RCM using unsymmetrical phosphonates or functional phosphinates, *Tetrahedron*, **2010**, *66*, 758-764.

29 B. Liu, T. Y. Liu, S. W. Luo and L. Z. Gong, Asymmetric hetero-Diels-Alder reaction of diazenes catalyzed by chiral silver phosphate: water participates in the catalysis and stereocontrol, *Org. Lett.*, **2014**, *16*, 6164-6167.

30 D. Rodina, J. Vaith and S. M. Paradine, Ligand control of regioselectivity in palladium-catalyzed heteroannulation reactions of 1,3-Dienes, *Nat. Commun.*, **2024**, *15*, 5433.

31 K. E. Houghtling, A. M. Canfield and S. M. Paradine, Convergent Synthesis of Dihydrobenzofurans via Urea Ligand-Enabled Heteroannulation of 2-Bromophenols with 1,3-Dienes, *Org. Lett.*, **2022**, *24*, 5787-5790.

32 H. Saito, R. Kato, K. Ikeuchi, T. Suzuki and K. Tanino, 8π Electrocyclic Reaction of Phosphonate Derivatives: Access to Seven-Membered Cross-Conjugated Cyclic Trienes, *Org Lett*, **2021**, *23*, 9606-9610.

33 H. J. Bestmann, K. Roth and M. Ettlinger, Kumulierte Ylide, XII. Eine stereoselektive Synthesemethode für (Z)-α,β-ungesättigte Aldehyde2), *Chem. Ber.*, **1982**, *115*, 161-171.

34 D. J. Shyh Tsai and D. S. Matteson, A stereocontrolled synthesis of Z and E terminal dienes from pinacol E-1-trimethylsilyl-1-propene-3-boronate, *Tetrahedron Lett*, **1981**, *22*, 2751-2752.

35 L. W. Erickson, E. L. Lucas, E. J. Tollefson and E. R. Jarvo, Nickel-Catalyzed Cross-Electrophile Coupling of Alkyl Fluorides: Stereospecific Synthesis of Vinylcyclopropanes, *J. Am. Chem. Soc.*, **2016**, *138*, 14006-14011.
